# Genome-wide survey and expression analysis of GRAS transcription factor family in sweetpotato provides insights into their potential roles in stress response

**DOI:** 10.1186/s12870-022-03618-5

**Published:** 2022-05-06

**Authors:** Chengbin Zhang, Siyuan Liu, Delong Liu, Fen Guo, Yiyu Yang, Tingting Dong, Yi Zhang, Chen Ma, Zixuan Tang, Feifan Li, Xiaoqing Meng, Mingku Zhu

**Affiliations:** 1 Institute of Integrative Plant Biology, School of Life Sciences, Jiangsu Normal University, Xuzhou, 221116 Jiangsu Province China; 2 Jiangsu Key Laboratory of Phylogenomics & Comparative Genomics, School of Life Sciences, Jiangsu Normal University, Xuzhou, 221116 Jiangsu Province China

**Keywords:** Abiotic stress, GRAS transcription factor, Expression analysis, Molecular characterization, Sweetpotato

## Abstract

**Background:**

The plant-specific GRAS transcription factors play pivotal roles in various adverse environmental conditions. Numerous GRAS genes have been explored and characterized in different plants, however, comprehensive survey on GRASs in sweetpotato is lagging.

**Results:**

In this study, 72 putative sweetpotato *IbGRAS* genes with uneven distribution were isolated on 15 chromosomes and classified into 12 subfamilies supported by gene structures and motif compositions. Moreover, both tandem duplication and segmental duplication events played critical roles in the expansion of sweetpotato GRAS genes, and the collinearity between *IbGRAS* genes and the related orthologs from nine other plants further depicted evolutionary insights into GRAS gene family. RNA-seq analysis under salt stress and qRT-PCR detection of 12 selected *IbGRAS* genes demonstrated their significant and varying inductions under multiple abiotic stresses (salt, drought, heat and cold) and hormone treatments (ABA, ACC and JA). Consistently, the promoter regions of *IbGRAS* genes harbored a series of stress- and hormone-associated cis-acting elements. Among them, IbGRAS71, the potential candidate for breeding tolerant plants, was characterized as having transactivation activity in yeasts, while IbGRAS-2/-4/-9 did not. Moreover, a complex interaction relationship between IbGRASs was observed through the interaction network analysis and yeast two-hybrid assays.

**Conclusions:**

Our results laid a foundation for further functional identifications of *IbGRAS* genes, and multiple members may serve as potential regulators for molecular breeding of tolerant sweetpotato.

**Supplementary Information:**

The online version contains supplementary material available at 10.1186/s12870-022-03618-5.

## Background

Sweetpotato (*Ipomoea batatas* L.) is a pivotal food crop, ranking seventh in the world, and is the only crop with starch storage roots in the Convolvulaceae [1, 2]. Asia is the largest sweetpotato-planting region, accounting for more than 50% of the world's planted area, and the production accounts for about 80% (approximately 68% in China) [2]. Not only can sweetpotato be applied in human diet (which has long been considered a food security source against famine), animal feed and starch processing, this crop can also be employed as an important alternative source of bioenergy. Owing to its inherent tolerance to stressful conditions, sweetpotato can tolerate various edaphic and climatic conditions, and can grow under limited input requirements, while its productivity and quality are widely threatened by biotic and abiotic stresses. Gene engineering has been increasingly applied to enhance its stress tolerance and quality, to date, multiple genes associated with abiotic stress tolerance and disease resistance have been identified from sweetpotato [2]. Among them, transcription factors (TFs) are extraordinary components that participate in the modulation of signal transductions and the regulation of stress-related target genes via binding their specific cis-elements, such as bHLH, bZIP, AP2/ERF, NAC, WRKY and GRAS TF family [3–8]. For instance, overexpression of bZIP TF IbABF4 increases the drought and salt stress tolerance of transgenic *Arabidopsis* and sweetpotato [9].

The name of plant-specific GRAS TFs was derived from its first three-member, including Gibberellic Acid Intensive (GAI), Repressor of GAI-3 mutant (RGA), and Scarecrow (SCR). They appeared in land plants through the lateral transfer from bacteria, and radiated in the ancestors of bryophytes, lycophytes and higher plants [10]. Typically, GRAS proteins consist of 360 ~ 850 amino acid residues, including a hypervariable N-terminus and a highly-conserved C-terminus [7]. The C-terminus is composed of five conserved motifs in the order: leucine heptad repeat I (LHR I), VHIID, LHR II, PFYRE and SAW, which are pivotal for the dimerization of GRAS and other proteins including TFs [7, 11, 12]. For example, the *Arabidopsis* GRAS protein SCL14 can interact with TGA TFs and is necessary for activating the stress-inducible promoters [13]. Previously, eight subfamilies: DELLA, HAM, SCL4/7, PAT1, LS, SCR, SHR and SCL9 were generally identified based on the report from woad, tomato and Chinese cabbage [14]. Subsequently, 13 and 16 branches were classified in *Brassica napus* and *Medicago truncatula* [15], respectively, suggesting the complexity of GRAS gene classification. Presently, the genome-wide isolation of GRASs have been extensively conducted in many plants, a total of 57, 62, 81, and 48 GRASs were found in monocots such as *Oryza sativa* [16]*, Hordeum vulgare* [17]*, Sorghum bicolor* [18] and *Brachypodium distachyon* [19]*,* respectively. In addition, 32, 35, 117, 87, 53, 52, 88, 150 members were found in eudicots including *Arabidopsis thaliana* [16]*, Cucumis sativus* [20]*, Glycine max* [21]*, Brassica napus* [14], *Solanum lycopersicum* [22]*, Camellia sinensis* [23]*, Brassica juncea* [24]*, Gossypium hirsutum* [25], respectively.

GRAS proteins have been increasingly demonstrated to play diverse and important roles in a variety of biological processes, including radial organization of roots [26], phytochrome and gibberellin signaling [11], chlorophyll biosynthesis [27], anther microsporogenesis [28] and meristem maintenance [11]. Our previous findings also exhibited that the GRAS protein SlFSR participated in the regulation of tomato fruit shelf-life [29]. Moreover, GRASs also function as the principal regulators in the signal transduction networks that modulate multiple adverse environmental conditions, including salt, drought and cold stress [7, 30]. For example, the transcription of *NtGRAS1* was significantly enhanced by H_2_O_2_ and SA, and it may functioned as an important regulator involved in plant stress response [31]. Rice OsGRAS23 was revealed as a positive regulator of drought tolerance via inducting a series of stress-related genes [32]. Overexpression of the GRAS gene *PeSCL7* from poplar and *VaPAT1* from *Vitis amurensis* both confers drought and salt resistance in *Arabidopsis* [33, 34], and overexpression of *VaPAT1* improves cold tolerance by regulating JA biosynthesis in grape calli [30]. Likewise, the GRAS TFs BrLAS from *Brassica rapa* and HcSCL13 from *Halostachys caspic*a are involved in drought or salt stress tolerance in transgenic *Arabidopsis* [35, 36]. Nevertheless, although GRAS proteins function as vital integrator in plant growth and development and in response to abiotic stress, the specific roles and regulatory mechanisms of most GRASs in many plants remain unknown.

The recently completed sweetpotato genome sequencing has created sufficient conditions for the exploration of specific TF families in the whole genome [37]. However, until recently, information about the GRAS genes in sweetpotato was almost inaccessible. Previously, although 70 *ItfGRASs* were isolated in *Ipomoea trifida*, which is the most likely diploid wild relative of sweetpotato [38], its genome information could not be served as plenitudinous representations of the genome sequence of cultivated sweetpotato. The identification of molecular characterization of the important GRAS TF family will provide clues for understanding the adaptive mechanisms of plants to environmental stresses. Here, the genome- and transcriptome-wide characterization of GRAS proteins in sweetpotato were carried out, and the possible IbGRASs associated with stress tolerance were screened. The present systematic research provided insights into the evolutionary relationships of *IbGRAS* genes in sweetpotato and further functional exploration of their potential roles in response to abiotic stress.

## Results

### Identification and characterization of the GRAS gene family members in sweetpotato

In this study, all the possible GRAS TFs were screened using the known GRAS proteins from *Arabidopsis* and rice as inquire sequences by the BLASTP program. Ultimately, a total of 77 possible non-redundant *IbGRAS* genes were identified, and five genes were excluded because their GRAS domains contain too few amino acids than the typical GRAS proteins (Table 1 and Additional file 1). Whereafter, the remaining 72 genes were named *IbGRAS1* ~ *IbGRAS72* based on the positions of 15 sweetpotato chromosomes from top to bottom (Additional file 2). Afterwards, the protein length (aa), molecular weight (Mw), theoretical isoelectric point (pI), subcellular location, and potential phosphorylation site of 72 IbGRAS proteins were analyzed. The length and Mw of IbGRASs varied greatly, with lengths ranging from 258 aa (IbGRAS33) to 1400 aa (IbGRAS16), correspondingly, their Mw varies from 28,885.31 to 157,318.47 Da, and the theoretical pI distributes from 4.7 (IbGRAS43) to 9.63 (IbGRAS40). The predicted subcellular localizations suggested that all IbGRAS proteins were located in the nucleus. Besides, predictions of potential phosphorylation sites suggested that IbGRASs contain 25 (IbGRAS10) to 152 (IbGRAS16) phosphorylation sites, of which all IbGRAS proteins contain more Ser sites than Tyr and Thr sites, over 80% of the IbGRAS proteins contain at least 40 phosphorylation sites (Table 1).

**Table 1:** Characteristics of IbGRAS proteins in *Ipomoea batatas*

Gene name	Gene ID	Amino acids	MW (Da)	PI	Subcellular location	No. of phosphorylation cite			
						Ser site	Tyr cite	Thr cite	Total
*IbGRAS1*	g37.t1	440	49,553.23	7.03	Nucleus	35	0	5	40
*IbGRAS2*	g255.t1	580	64,349.79	6.64	Nucleus	44	8	14	66
*IbGRAS3*	g728.t1	550	62,148.77	4.8	Nucleus	40	6	16	62
*IbGRAS4*	g1041.t1	438	48,706.35	5.2	Nucleus	31	2	13	46
*IbGRAS5*	g1240.t1	404	45,861.05	6.71	Nucleus	18	1	6	25
*IbGRAS6*	g1843.t1	476	53,812.33	7.69	Nucleus	31	4	13	48
*IbGRAS7*	g3693.t1	619	69,881.79	5.71	Nucleus	35	8	21	64
*IbGRAS8*	g3694.t1	663	74,448.02	5.58	Nucleus	41	8	23	72
*IbGRAS9*	g3695.t1	668	75,276.89	5.8	Nucleus	38	9	17	64
*IbGRAS10*	g4401.t1	399	44,313.72	6.68	Nucleus	14	0	11	25
*IbGRAS11*	g4931.t1	700	78,864.66	5.39	Nucleus	48	7	17	72
*IbGRAS12*	g4932.t1	510	57,949.54	5.8	Nucleus	34	8	16	58
*IbGRAS13*	g4933.t1	651	72,735.21	8.94	Nucleus	33	12	16	61
*IbGRAS14*	g4934.t1	669	74,709.41	8.88	Nucleus	52	6	21	79
*IbGRAS15*	g5283.t1	472	51,656.59	6.07	Nucleus	29	2	8	39
*IbGRAS16*	g5532.t1	1400	157,318.47	8.52	Nucleus	98	10	44	152
*IbGRAS17*	g7395.t1	468	53,007.31	5.37	Nucleus	22	2	12	36
*IbGRAS18*	g8862.t1	475	53,674.71	5.84	Nucleus	38	0	5	43
*IbGRAS19*	g9153.t1	678	73,651.07	5.85	Nucleus	47	1	9	57
*IbGRAS20*	g9443.t1	543	61,322.88	6	Nucleus	22	7	15	44
*IbGRAS21*	g9997.t1	619	69,725.66	6.07	Nucleus	33	11	13	57
*IbGRAS22*	g9998.t1	627	69,776.03	5.28	Nucleus	50	8	9	67
*IbGRAS23*	g13787.t1	424	47,740.17	5.45	Nucleus	35	3	7	45
*IbGRAS24*	g13824.t1	493	54,143.89	4.99	Nucleus	38	6	13	57
*IbGRAS25*	g15537.t1	704	77,064.19	5.81	Nucleus	55	3	11	69
*IbGRAS26*	g15890.t1	649	71,865.88	5.59	Nucleus	53	3	10	66
*IbGRAS27*	g17048.t1	536	58,471.78	5.07	Nucleus	43	2	11	56
*IbGRAS28*	g17059.t1	401	44,391.65	5.87	Nucleus	22	7	0	29
*IbGRAS29*	g17892.t1	719	81,278.09	5.76	Nucleus	41	14	14	69
*IbGRAS30*	g17993.t1	397	44,570.96	5.03	Nucleus	24	5	12	41
*IbGRAS31*	g20285.t1	815	91,263.15	6.01	Nucleus	56	6	14	76
*IbGRAS32*	g20603.t1	655	70,851.81	5.9	Nucleus	44	2	14	60
*IbGRAS33*	g22885.t1	258	28,885.31	8.81	Nucleus	19	1	5	25
*IbGRAS34*	g22892.t1	491	53,837.29	5.16	Nucleus	43	6	10	59
*IbGRAS35*	g24498.t1	380	42,592.07	4.75	Nucleus	25	3	14	42
*IbGRAS36*	g25605.t1	759	83,820.78	5.21	Nucleus	56	7	18	81
*IbGRAS37*	g26040.t1	520	56,465	5.36	Nucleus	36	6	10	52
*IbGRAS38*	g29038.t1	455	50,675.74	5.56	Nucleus	21	3	13	37
*IbGRAS39*	g29056.t1	514	57,766.06	5.73	Nucleus	45	3	20	68
*IbGRAS40*	g29244.t1	403	44,053.33	9.63	Nucleus	40	1	14	55
*IbGRAS41*	g29248.t1	543	59,709.71	5.71	Nucleus	41	3	14	58
*IbGRAS42*	g29289.t1	543	59,709.71	5.71	Nucleus	42	3	14	59
*IbGRAS43*	g29317.t1	575	62,273.67	4.7	Nucleus	31	3	15	49
*IbGRAS44*	g29775.t1	549	59,958.16	5.43	Nucleus	34	3	15	52
*IbGRAS45*	g30418.t1	515	56,666.08	5.22	Nucleus	44	2	11	57
*IbGRAS46*	g30921.t1	443	49,108.02	5.27	Nucleus	23	2	13	38
*IbGRAS47*	g30985.t1	545	61,029.21	6.36	Nucleus	41	11	8	60
*IbGRAS48*	g30993.t1	398	43,424.43	5.61	Nucleus	26	4	5	35
*IbGRAS49*	g33366.t1	430	46,238.56	5.24	Nucleus	35	1	11	47
*IbGRAS50*	g37852.t1	389	42,543.22	4.81	Nucleus	22	2	10	34
*IbGRAS51*	g38267.t1	765	84,510.55	5.44	Nucleus	45	2	13	60
*IbGRAS52*	g39630.t1	417	46,288.47	5.68	Nucleus	28	1	14	43
*IbGRAS53*	g41663.t1	492	55,569.44	5.68	Nucleus	26	2	10	38
*IbGRAS54*	g41664.t1	592	67,759.91	5.46	Nucleus	36	2	12	50
*IbGRAS55*	g42253.t1	575	63,759.94	5.18	Nucleus	42	6	14	62
*IbGRAS56*	g42381.t1	433	47,696.89	5.16	Nucleus	29	4	13	46
*IbGRAS57*	g43994.t1	440	48,607.53	5.67	Nucleus	27	3	8	38
*IbGRAS58*	g44030.t1	470	52,666.62	5.66	Nucleus	29	4	9	42
*IbGRAS59*	g46988.t1	527	59,379.06	7.16	Nucleus	21	1	11	33
*IbGRAS60*	g49861.t1	476	53,651.93	6.07	Nucleus	30	1	10	41
*IbGRAS61*	g49862.t1	508	57,225.82	5.97	Nucleus	25	4	13	42
*IbGRAS62*	g50211.t1	578	62,587.2	5.1	Nucleus	44	5	16	65
*IbGRAS63*	g50932.t1	463	51,524.79	5.75	Nucleus	35	2	8	45
*IbGRAS64*	g51820.t1	385	42,500.97	5.91	Nucleus	33	1	16	50
*IbGRAS65*	g53996.t1	537	60,241.04	5.24	Nucleus	32	4	11	47
*IbGRAS66*	g54776.t1	514	57,548.38	6.36	Nucleus	39	10	14	63
*IbGRAS67*	g58760.t1	745	80,641.02	6.07	Nucleus	51	3	13	67
*IbGRAS68*	g58849.t1	473	53,864.39	4.87	Nucleus	34	9	8	51
*IbGRAS69*	g59046.t1	529	58,934.89	5.74	Nucleus	40	9	11	60
*IbGRAS70*	g60968.t1	530	58,961.24	5.64	Nucleus	23	2	14	39
*IbGRAS71*	g61121.t1	577	63,925.13	5.62	Nucleus	45	10	18	73
*IbGRAS72*	g64099.t1	726	80,342.49	5.7	Nucleus	55	9	15	79

### Chromosome distribution of sweetpotato IbGRAS genes

**Fig. 1:**
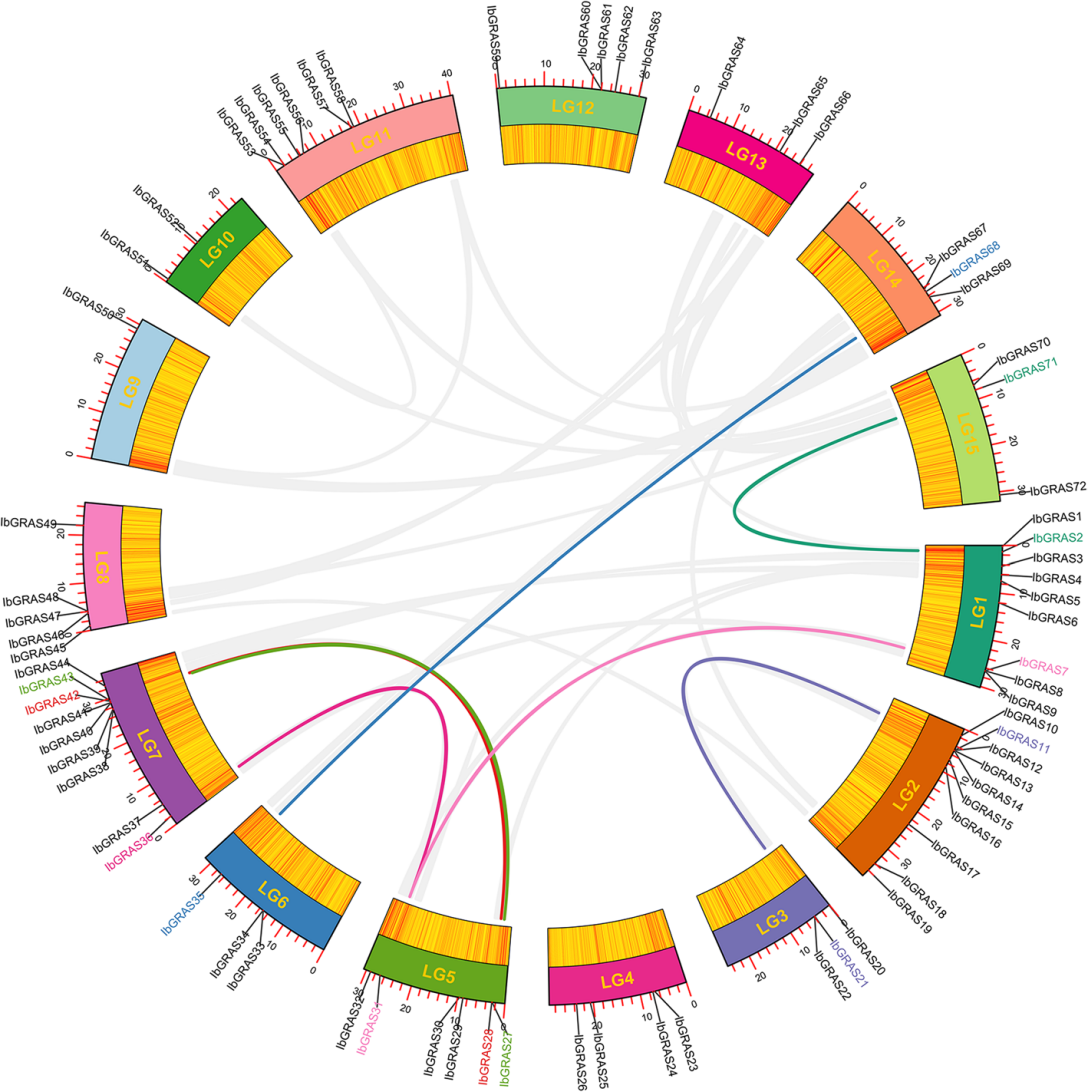
Inter-chromosomal relations of *IbGRAS* genes in sweetpotato chromosomes. Chromosomes LG1-LG15 are indicated by colored rectangles. The gene density on each chromosome is depicted by the heatmap along each rectangle. The colored curves represent duplicated *IbGRAS* gene pairs. The corresponding *IbGRAS* genes located in segmental duplications are marked with colors

The detection of physical position based on the GFF3 genome annotations displayed that 72 *IbGRAS* genes were mapped on all 15 chromosomes. Among them, Chr 1 and Chr 2 contain the most abundant *IbGRAS* genes, with nine and 10 members, respectively. However, Chr 9 and Chr 10 contain only one and two *IbGRAS* genes, respectively. The number of *IbGRAS* genes located in the remaining chromosomes ranges from three to seven (Fig. 1 and Additional file 2). These results revealed that the distribution of *IbGRAS* genes is highly variable and disproportionate to chromosome length. For example, the large chromosome (Chr 9) contains only one *IbGRAS* gene, while the small chromosome (Chr 3) contains three *IbGRAS* genes.

### Phylogenetic relationships of IbGRAS proteins in sweetpotato

**Fig. 2:**
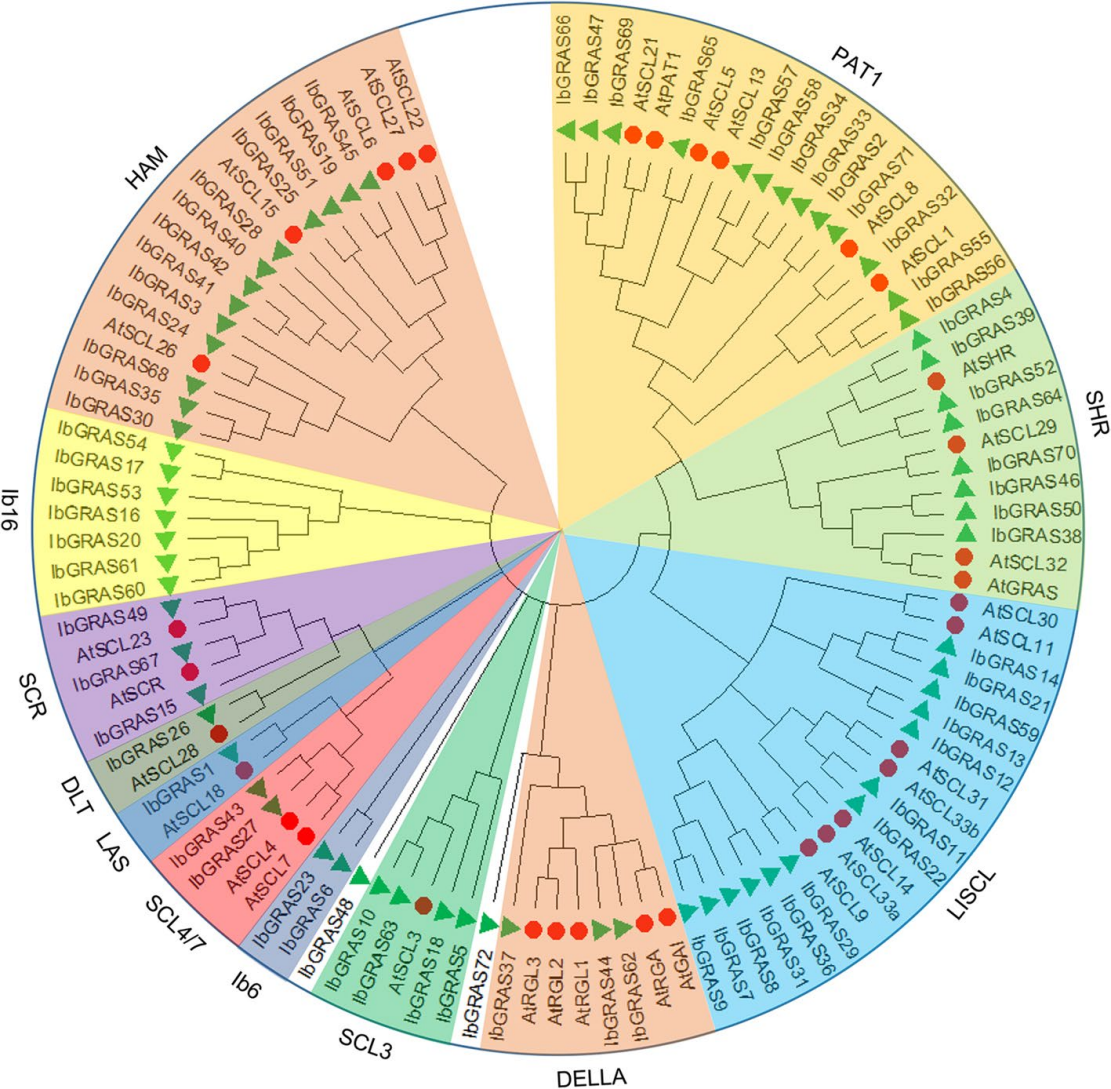
Unrooted phylogenetic tree of sweetpotato IbGRASs and *Arabidopsis* AtGRASs. The phylogenetic relationships were derived through the Maximum Likelihood method and the best evolutionary model JTT + G + F calculated through MEGA X was selected with the bootstrap value of 1000. Different subgroups are named based on the reports in *Arabidopsis* and are distinguished with different colors. The gene names are marked at the end of the branch, the red circle and green triangle represent the sweetpotato IbGRASs and *Arabidopsis* AtGRASs, respectively

To investigate the evolutionary relations and classifications of IbGRASs in sweetpotato, the unrooted phylogenetic tree was constructed using the entire amino acid sequences of sweetpotato IbGRASs and known classified AtGRASs in *Arabidopsis* (Additional file 3) using MEGA-X software. According to the previous classification of *Arabidopsis* AtGRASs [39], 72 IbGRAS proteins are divided into 12 subfamilies (except the IbGRAS48 and IbGRAS72), of which there are eight known subfamilies and two newly identified subfamilies, Ib6 and Ib16. The distributions of IbGRAS proteins in different subgroups were widely dispersed and unevenly. The three largest subgroups (LISCL, PAT1 and HAM) have 18–19 members, and all contain 13 sweetpotato IbGRAS proteins. However, relatively small ones were obtained in the SCR, DLT, LAS, Ib6, SCL3 and SCL4/7 subgroups with only 2–5 GRAS members. Interestingly, IbGRAS48 and IbGRAS72 do not belong to any of the 12 subgroups mentioned above, implying their possible unique functions (Fig. 2).

### Gene duplication survey of sweetpotato IbGRAS genes

Genome duplication events have promoted the evolution and expansion of many plant gene families [40]. To deduce the possible relationships among the 72 *IbGRAS* genes, a collinear analysis was conducted. The results suggested that six tandem duplication events were found among the 72 *IbGRAS* genes, including *IbGRAS8-IbGRAS7/9*, *IbGRAS11-IbGRAS12*, *IbGRAS13-IbGRAS14*, *IbGRAS53-IbGRAS54*, and *IbGRAS60-IbGRAS61* (Additional file 2). The genes exhibiting tandem repeat events are members of the same subgroup (Fig. 2). Furthermore, segmental duplications were found using the BlastP and MCScanX programs and seven gene pairs with segmental duplications were observed on eight of the 15 chromosomes as follows: *IbGRAS7/36-IbGRAS31*, *IbGRAS2-IbGRAS71*, *IbGRAS11-IbGRAS21*, *IbGRAS27- IbGRAS43*, *IbGRAS28-IbGRAS42,* and *IbGRAS68-IbGRAS35* (Fig. 1 and Additional file 4). Visibly, some chromosomes (LG1, LG5 and LG7) had more linkage groups than others. Similarly, all of these linked genes were linked within their subgroups. The results suggest that gene duplications have a potential contribution to the expansion of *IbGRAS* genes.

### Collinearity analysis of GRAS genes between sweetpotato and other plants

**Fig. 3:**
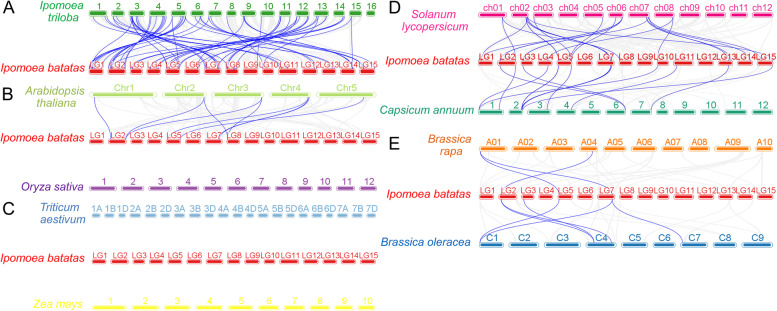
Synteny analyses of GRAS genes between sweetpotato and nine representative plant species from *Ipomoea triloba* (**A**), *Arabidopsis thaliana* and *Oryza sativa* (**B**), *Triticum aestivum* and *Zea mays* (**C**), *Solanum lycopersicum* and *Capsicum annuum* (**D**), and *Brassica rapa* and *Brassica oleracea* (**E**). The chromosomes of different plants are distinguished with differential colors. The blue lines connecting two different chromosomes highlight the syntenic GRAS gene pairs within sweetpotato and other plant genomes, respectively

To further infer the origin and evolutionary mechanisms of sweetpotato *IbGRAS* gene*s*, the comparative syntenic relationships between 72 *IbGRAS* genes and the related genes from nine representative species were explored, including the likely diploid wild relative of sweetpotato (*Ipomoea triloba*), the two most representative model plants (*Arabidopsis thaliana* and *Oryza sativa*), two Solanaceae plants (*Solanum lycopersicum* and *Capsicum annuum*), two Brassica plants (*Brassica rapa* and *Brassica oleracea*) and two cereal plants (*Triticum aestivum* and *Zea mays*). A total of 53 (73.6%) *IbGRAS* genes displayed syntenic relationships with those in *Ipomoea triloba*, followed by *Solanum lycopersicum* (23), *Capsicum annuum* (15), *Arabidopsis thaliana* (7), *Brassica oleracea* (4) and *Brassica rapa* (3). However, no such orthologous genes were observed between sweetpotato and three cereal plants *Oryza sativa*, *Triticum aestivum* and *Zea mays* (Fig. 3). It is worth mentioning that the collinearity between *IbGRAS* genes and *Ipomoea triloba* genes is greater than that identified with the other eight species, which may be related to the fact that *Ipomoea triloba* is the likely diploid wild relative of sweetpotato.

Moreover, we found that 8 *Ipomoea triloba* genes had a collinearity relationship with two sweetpotato *IbGRAS* genes, such as *itb03g09330.t2*-*IbGRAS-4/-39*, *itb03g16290.t1/itb12g22970.t1* -*IbGRAS-31/-36*, and *itb05g26310.t3/itb06g15290.t1-IbGRAS-2*/-*71* (Additional file 5). Interestingly, we found that some collinear gene pairs (with four *IbGRAS* genes: *IbGRAS-19/-32/-39/-45*) identified between sweetpotato and *Ipomoea triloba*/*Arabidopsis thaliana*/*Solanum lycopersicum*/*Capsicum annuum* were not found between sweetpotato and the two Brassica plants. Differently, three *IbGRAS* genes (*IbGRAS-11/-28/-42*) were found to be collinear with at least one syntenic gene among all the detected species with orthologous genes (Additional file 6), suggesting that they might be derived from a common ancestor of these plants.

### Gene structure and conserved motif analysis of IbGRAS genes

**Fig. 4:**
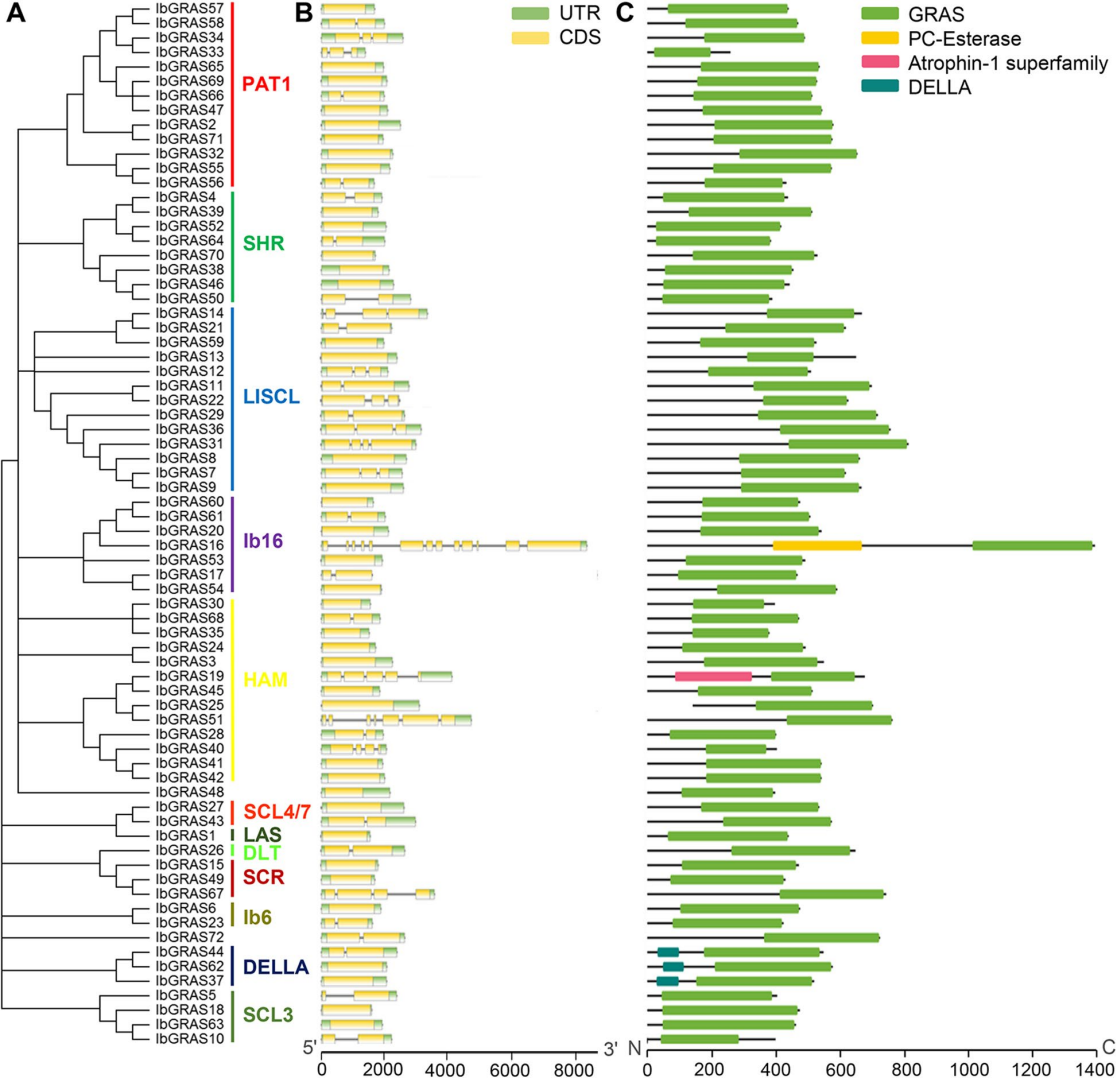
Phylogenetic relationships, gene structures and conserved domain distributions of 72 *IbGRAS* genes in sweetpotato. **A**. The phylogenetic tree of 72 IbGRASs was constructed by MEGA X based on the consistent parameters used in Fig. 2. **B**. Gene structures of 72 *IbGRAS* genes. Exons and UTR are marked using yellow and green bars, respectively, black lines indicate introns. **C**. Distributions of conserved domains detected by CD-search in the IbGRAS members. The colorful boxes present different conserved domains

To evaluate the sequence diversity of sweetpotato *IbGRAS* genes, the exon–intron structures and conserved domains of each *IbGRAS* were detected. The data exhibited that 39 *IbGRAS* genes (54.2%) were mono-exonic and 20 *IbGRAS* genes (27.8%) only contain one intron, which was similar to the previous results [18, 19, 38]. Moreover, previous data suggested that members of the same subgroup had similar gene structures and sequence compositions [18]. Similarly, our findings displayed that the majority of *IbGRAS* genes in the same subgroups generally possessed similar gene structures. However, some *IbGRAS* genes showed obvious exceptions in the same subgroups with differential gene structures, such as *IbGRAS16* in the subgroup Ib16 and *IbGRAS51* in the subgroup HAM (Fig. 4A and B). The results verified by Pfam, CD-search and Prosite analysis suggested that the amino acid sequences of 72 IbGRAS proteins all shared a highly conserved GRAS domain, which is consistent with our expectations. Additionally, three IbGRAS members from the DELLA subgroup all contain an exclusive DELLA domain, and IbGRAS16 and IbGRAS19 include a PC-Esterase and Atrophin-1 superfamily domain, respectively (Fig. 4 C).

To further survey the sequence characteristics of IbGRAS proteins, the motif composition was explored using the MEME tool. The results displayed that a total of 19 distinct motifs were found based on the previous settings in rice and *Arabidopsis* [16]. Consequentially, the IbGRASs within the same subgroups generally share similar motif compositions, which further support the subgroup classification. Similar to their homologs in many other plants including, *Arabidopsis*, rice, *Brassica napus*, and tomato [14, 16, 22], each IbGRAS possesses a GRAS domain consisting of LHRI, VHIID, LHRII, PFYRE and SAW at their C-terminus, and most motifs were located in the conserved GRAS domains. Despite this, many domain-loss events were observed in multiple IbGRAS members. Moreover, the N-terminus of IbGRASs varies substantially, while some members of the same subgroups possess certain conserved motifs, especially the LISCL subgroup (Additional file 7). For instance, motifs 13 and 15 were specifically found in almost all LlSCL members, and they might contain molecular recognition features required for the protein interaction [19]. Besides, although LHRI-A1, -A2 and -B were all units of the LHRI domain, they displayed different amino acids and were distributed in different subgroups. For example, the entire LHRI domain was mainly observed in LlSCL and PAT1 subfamilies, and missing or incomplete ones were found in other subfamilies. And the complete LHRII domain was prominently found in LlSCL subfamily, LHRII-A1, -A2, -B were mainly identified in PAT1, DELLA and SCL3 subfamilies, and other subfamilies only contained two or less units of LHRII domain, indicating the structure complexity of the members in different subgroups. Differently, the majority of IbGRASs contained the conserved VHIID domain, as well as the complete PFYRE and SAW domains, except for the HAM subfamily. The data suggest that the motif compositions and distributions vary remarkably among different GRAS subgroups, and specific motifs may imply distinct and diverse roles of *IbGRAS* genes in sweetpotato.

### Transcriptome‑wide identification of salt-responsive IbGRAS genes and their expression profiles in response to multiple abiotic stress and hormone treatments

**Fig. 5:**
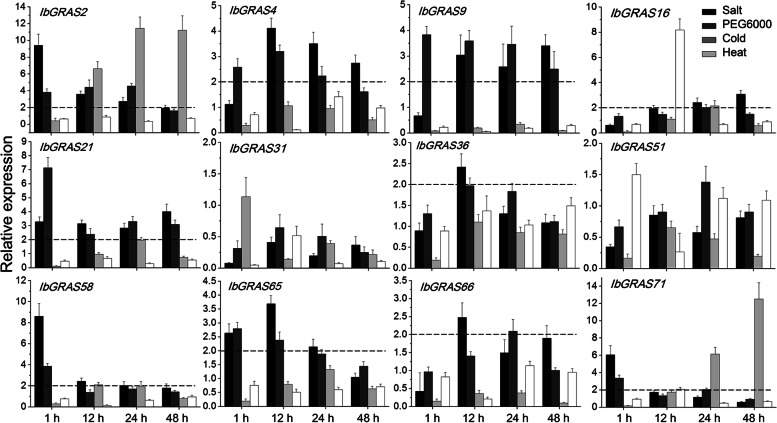
Relative expression levels of 12 *IbGRAS* genes in response to abiotic stresses detected by qRT-PCR. The abiotic stress treatments include salt (150 mM NaCl), drought (20% PEG6000), cold (4 °C) and heat (42 °C). The expression levels at 0 h were normalized to 1, and the Y-axis delineates the fold changes of relative expression comparing with 0 h. Bars represent the mean of three biological replicates ± SE. The two-fold threshold is presented by a dotted line

Increasing evidence demonstrated that GRAS TFs played diverse and critical roles in response to multiple abiotic stresses, such as salt, drought and cold. To determine the potential biological functions of *IbGRAS* genes in stress tolerance, their expression profiles under salt stress were first explored in salt-tolerant and salt-sensitive sweetpotato cultivars according to our previous RNA-seq data [41]. The results showed that about half of the screened *IbGRAS* genes was salt stress-responsive or genotype-specific (Additional file 8). Subsequently, the expression patterns of 12 *IbGRAS* genes (*IbGRAS-/-4*/-9/-16/-21/-31/-36/-51/-5*8*/-65/-66/-71) that displayed substantial change in the RNA-data was further examined under four abiotic stresses: salt, drought, heat and cold by qRT-PCR assay, and a two-fold cut-off value was explored [42]. The results revealed that most of these genes (10 out of 12, except *IbGRAS-31*/-*51*) exhibited significant and varied transcriptional abundance post four abiotic stress treatments. Among them, the expression of *IbGRAS-16/-71* could be induced by all the four stresses, the transcription of *IbGRAS-2*/-*58* was upregulated by three of the treatments, and nine *IbGRAS* genes (*IbGRAS-2*/-*4*/-*9*/-*16/-21*/-*58 /-65/-66/-71*) could be induced by both salt and drought stresses. Contrarily, the inhibited profiles of *IbGRAS31* and *IbGRAS51* expression were observed under all four abiotic stresses. Notably, *IbGRAS-2/-58/-71* exhibited the highest induction level under salt stress with about 6.1–9.4-fold changes, and a relative low induction level (2.4–4.1-fold) was detected in the expression of other *IbGRAS* genes (Fig. 5). The data are in good agreement with the RNA-seq data (Additional file 9). Similarly, the transcription of *IbGRAS21* was remarkably increased with about sevenfold changes under drought stress, and relative low upregulations (2.0–4.6-fold) were detected in the transcription of other *IbGRAS* genes. For cold and heat treatments, the expression of four *IbGRAS* genes (*IbGRAS-2/-16/-58*/-*71*) could be enhanced by cold stress with 2.1–12.5-fold, and only *IbGRAS-16/-71* expression was increased by heat stress with 2.1–8.2-fold (Fig. 5).

**Fig. 6:**
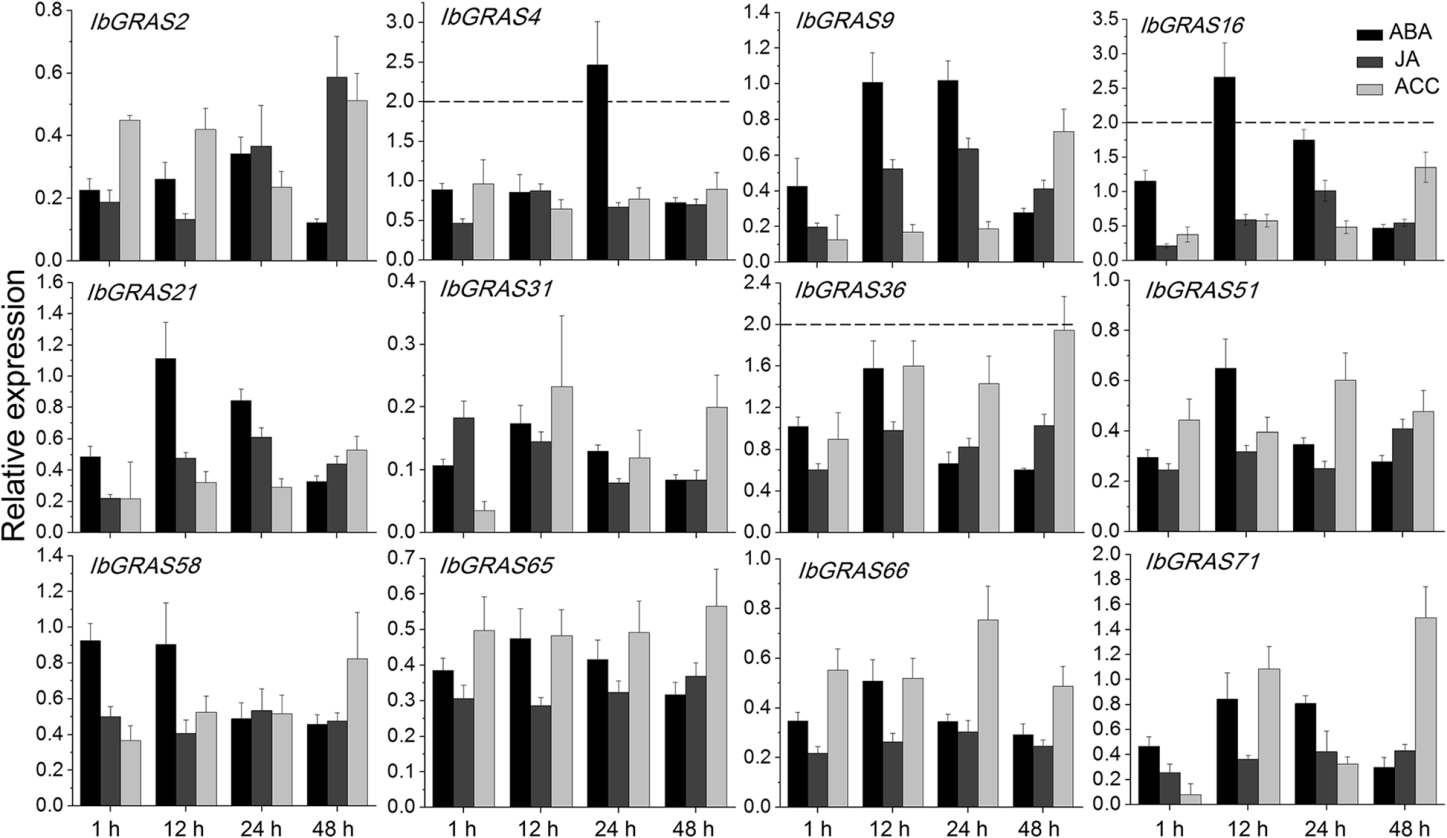
Relative expression levels detected by qRT-PCR under different hormone treatments including ABA, ACC and JA. The expression levels at 0 h were normalized to 1, and the Y-axis delineates the fold changes of relative expression comparing with 0 h. Bars represent the mean of three biological replicates ± SE. The two-fold threshold is presented by a dotted line

Additionally, the transcription profiles of 12 *IbGRAS* gene*s* were further detected under different hormone treatments by qRT-PCR including ABA, JA and ACC. It is reported that they function as vital messengers in the response of plants to multiple environmental conditions [43]. Unexpectedly, only the stress hormone ABA could induce the expression of *IbGRAS4* and *IbGRAS16* when we adopted a cut-off value of two-fold for differential gene expression. And the expression levels of most *IbGRAS* genes were downregulated at some time points post hormone treatments (Fig. 6). Similarly, previous report also showed that the transcription of most *BnGRAS* genes was not obviously induced by hormone treatments in *Brassica napus* [14]. Collectively, the data suggest that multiple IbGRAS members may function as important participants in response to hormones and/or abiotic stresses.

### Cis-element prediction in the promoters of IbGRAS genes

**Fig. 7:**
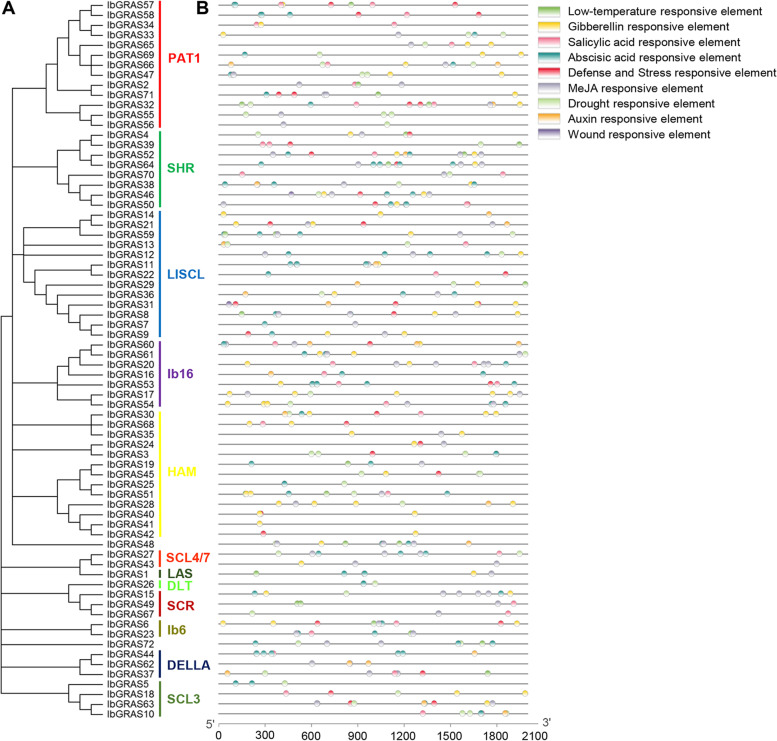
Phylogenetic clustering and predicted stress- and hormone-related cis-elements in the promoters of *IbGRAS* genes. **A**. The phylogenetic tree of 72 IbGRASs was constructed by MEGA X based on the consistent parameters used in Fig. 2. **B**. Predicted cis-elements in the *IbGRAS* promoters. 2000 bp promoter regions of each *IbGRAS* gene were detected by PlantCARE database. Different colored rectangles represent different cis-elements that are potentially involved in stress or hormone regulation

To explore the possible regulatory mechanism of IbGRASs in response to abiotic stresses and hormones, the cis-elements in the 2 kb upstream promoter sequences of each *IbGRAS* gene were scanned by the plantCARE database. The results revealed that the promoter regions of each *IbGRAS* have multiple stress- and/or hormone-related cis-elements. Among them, about 85% of the *IbGRAS* promoters contained multiple stress-related cis-elements, such as defense and stress responsive elements (TC-rich repeats), drought responsive elements (MBS), low temperature responsive elements (LTR), and wound responsive elements (WUN-motif). These cis-elements might be related to the expression profiles. For instance, the expression of multiple *IbGRAS* genes including *IbGRAS-2/-4/-9/-21/-58/-71* was improved by different stresses, accordingly, the MBS, TC-rich repeats or LTR cis-elements associated with stress response, are enriched in their promoter regions. However, exceptions are observed, for instance, although TC-rich repeats, MBS and LTR elements were observed in the promoters of *IbGRAS31* and *IbGRAS51* genes, their expressions were not enhanced by salt, drought or cold stress (Figs. 5 and 7, Additional file 9). Additionally, all *IbGRAS* promoters contain multiple hormone-responsive elements, such as abscisic acid responsive elements (ABRE), salicylic acid responsive elements (TCA-element), MeJA responsive elements (CGTCA-motif and TGACG-motif), gibberellin responsive elements (P-box, GARE-motif and TATC-box), or auxin responsive elements (TGA-box and AuxRR-core). Nevertheless, the transcription levels of most *IbGRAS* genes were not induced by the hormone treatments tested (Figs. 6 and 7, Additional file 9). Among them, 62.5% of the promoters contain abscisic acid response element. For instance, two ABRE sites were observed in the promoters of ABA-responsive *IbGRAS16* gene. The data indicate that these cis-elements may be involved in the abiotic stress and hormone responses.

### Interaction network analysis of the IbGRAS proteins in sweetpotato

**Fig. 8:**
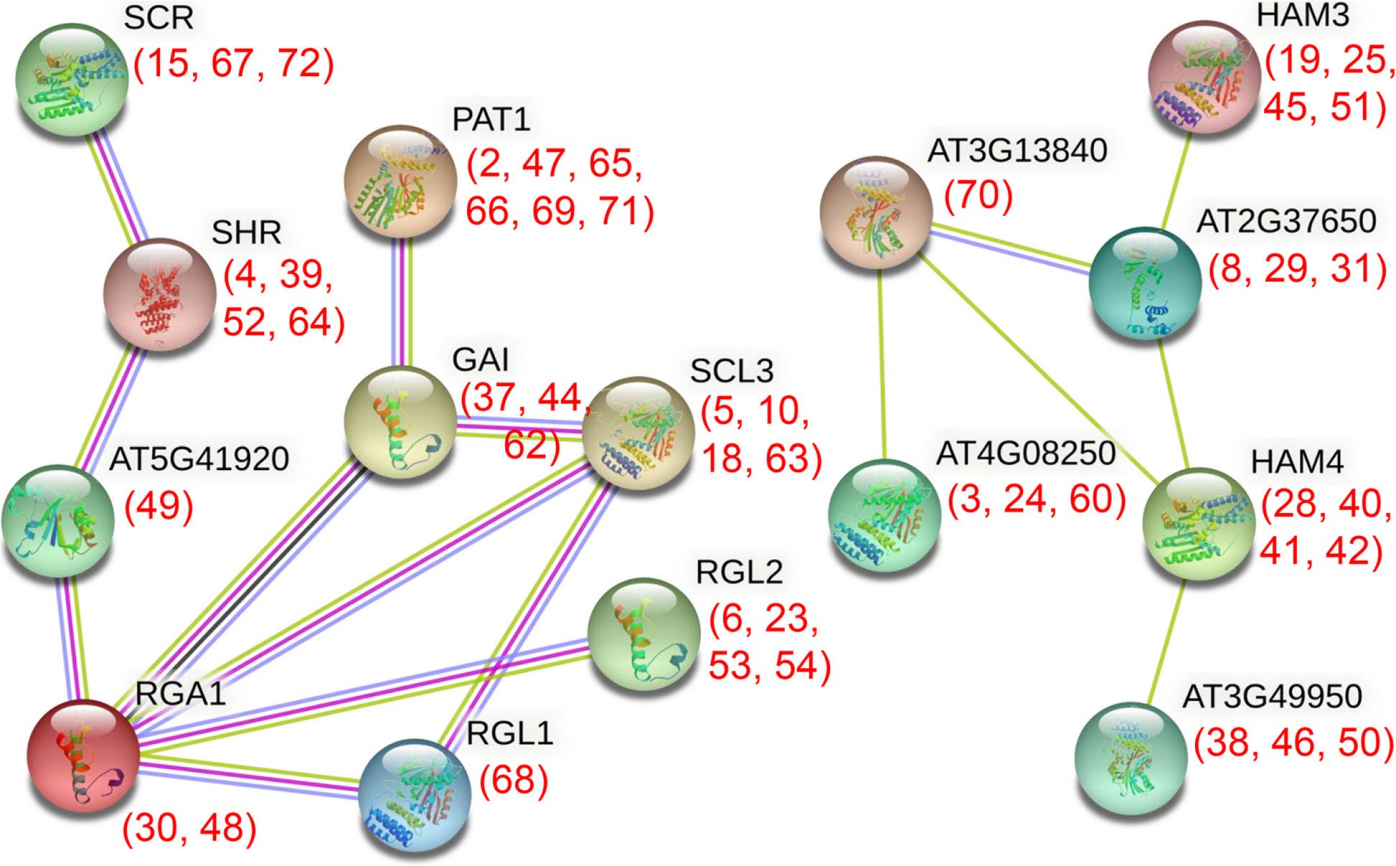
Interaction networks of IbGRAS proteins in sweetpotato according to the orthologues in *Arabidopsis*. The amino acid sequences of IbGRASs were employed to search the STRING database, network node represents proteins, and edge represents protein–protein associations. The colored lines between the nodes indicate the different kinds of interactions. The numbers (*IbGRAS* gene number) in brackets represent the corresponding orthologues in sweetpotato

The LHRI motif in GRAS domain was known to be necessary for protein interaction [44], indicating that IbGRASs may also function by forming homologous or heterologous protein complexes. Therefore, the protein interaction network for IbGRAS was constructed based on the orthologous analysis with *Arabidopsis* GRASs by STRING software (Fig. 8). Among these proteins, GAI (IbGRAS-37/-44/-62) was involved in reducing ROS accumulations in response to stress by upregulating the expression of superoxide dismutases. Additionally, IbGRASs that serve hormone signalling and growth and development were also observed. For instance, GAI (IbGRAS-37/-44/-62), RGA1 (IbGRAS-30/-48) and RGL2 (IbGRAS-6/-23/-53/-54) all act as GA signal repressors, and RGL2 could regulate seed germination and promote ABA biosynthesis. RGL1 (IbGRAS68) participated in floral development, seed germination and anther development. For SHR (IbGRAS-4/-39/-52/-64), it was required for the radial organization of the shoot axial organs and normal shoot gravitropism (https://string-db.org/). Thus, the results indicate that multiple IbGRAS members tend to form protein complexes, suggesting a potential way for *IbGRAS* genes to regulate the response to environmental stresses and plant growth and development.

### Detection of transactivation activity and protein interaction of selected IbGRASs

**Fig. 9:**
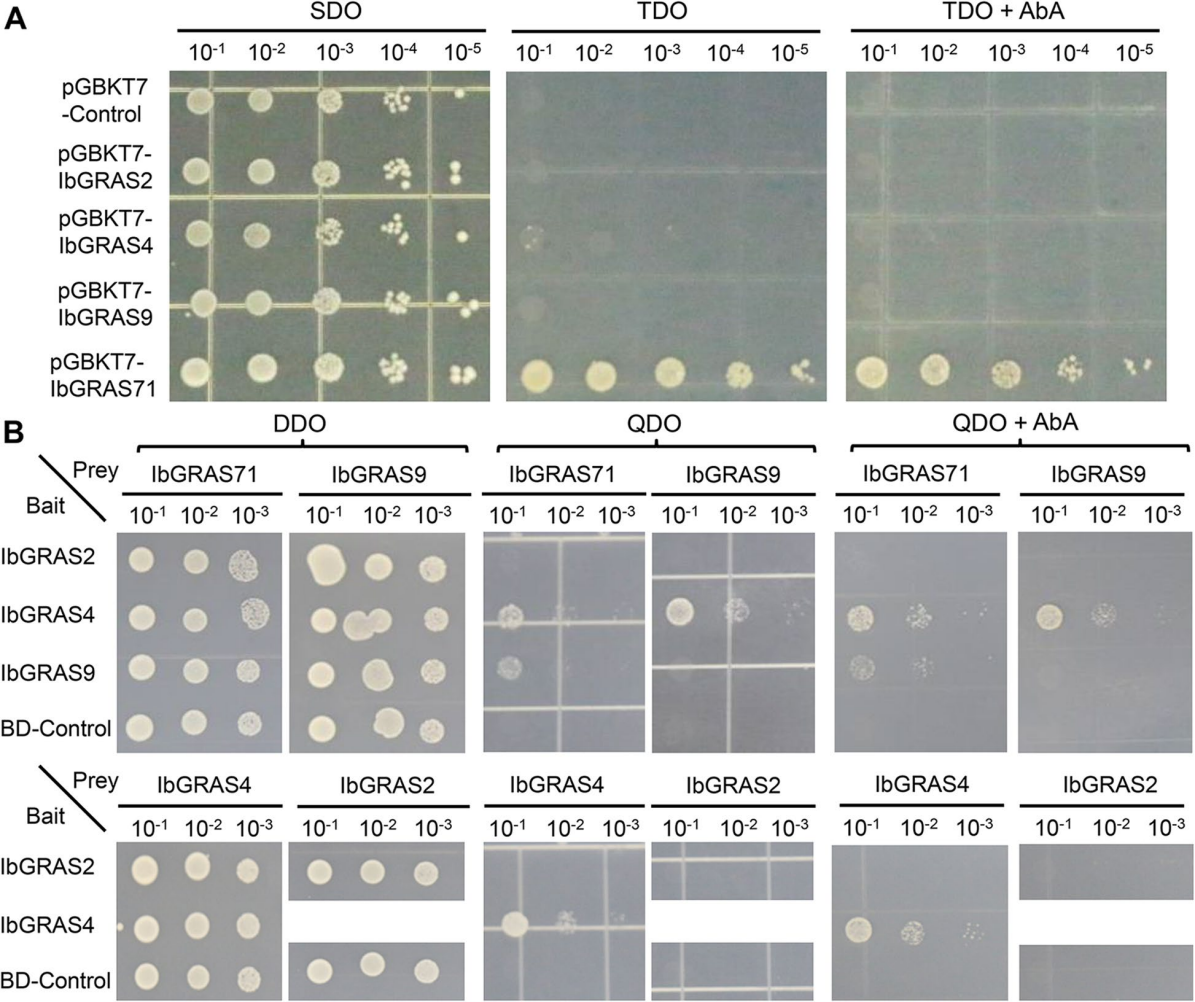
Analysis of transactivation activity and protein interaction of IbGRAS-2/-4/-9/-71 proteins. **A.** Yeasts containing pGBKT7-IbGRAS-2/-4/-9/-71 or pGBKT7 empty vector were streaked on the SDO (SD medium lacking Trp); TDO (SD medium lacking Trp, His, Ade) and TDO medium with 200 ng/mL AbA. **B.** Yeasts containing both the indicated recombined pGBKT7 and pGADT7 plasmids were streaked on DDO (SD/-Trp-Leu) medium, QDO (SD/-Trp-Leu-His-Ade) medium with or without 200 ng/mL AbA. All the plates were recorded 3 d after 30° of incubation

Considering that the expression of *IbGRAS-2/-4/-9/-71* genes was remarkably induced by various abiotic stresses, they were selected to detect possible interactions between them. First, the transactivation activity of four IbGRASs was detected by constructing recombinant pGBKT7 plasmids. The results demonstrated that all transformed yeasts could grow well on control SDO medium. Nevertheless, only transformed yeasts harboring IbGRAS71 could grow on the TDO and TDO with AbA (Aureobasidin A) medium, while the transformants containing the control pGBKT7 vector and recombinant pGBKT7-*IbGRAS-2/-4/-9* could not (Fig. 9A). The data suggest that IbGRAS71 protein has transactivation activity in yeasts, while IbGRAS-2/-4/-9 did not. Therefore, the interaction between any two of these four IbGRAS proteins was further tested by yeast two-hybrid assay (Y2H), except that pGBKT7-IbGRAS71 was not involved because of its self-activating activity. The results showed that all transformed yeasts could grow well on control QDO medium. And the results displayed that IbGRAS71 could interact with IbGRAS4 and IbGRAS9, and IbGRAS4 could also interact with IbGRAS9 and itself. IbGRAS2 could not interact with any IbGRAS detected including itself, and no interaction was observed in other combinations (Fig. 9B).

## Discussion

Plant-specific GRAS TFs represent a set of critical and diverse regulatory molecules in plant growth and development and in the response to multiple adverse environmental inputs have been increasingly elucidated. Their functional roles range from maintaining meristem to modulating hormone, light and stress signal transduction [7, 11]. Sweetpotato is an important crop widely used in food, animal feed, and industrial raw material. It has the advantages of wide adaptability, high yield and strong resistance to various environmental conditions [2, 45]. However, GRAS TFs in sweetpotato have not been comprehensively surveyed. This study systematically identified the GRAS TFs in sweetpotato**,** and the isolation of stress-responsive *IbGRAS* genes provide worthy foundation for further functional explorations of IbGRASs in stress tolerance.

A total of 72 *IbGRAS* genes were identified from the sweetpotato genomes, their protein lengths vary from 258 to 1400, and the theoretical pI distributes from 4.7 to 9.63. The significant differences and variabilities suggest the high degrees of complexity, which may be associated with gene-duplication events or genome sizes [18]. Previous finding showed that allopolyploidization was the major cause for the rapid expansion of the GRAS genes in *Brassica napus* [14]. However, a similar number of GRAS genes (70 *ItfGRASs*) were isolated from *Ipomoea trifida*, which is the most likely diploid wild relative of sweetpotato [38]. Such inconsistency may be due to the limitations of half-haplotype-resolved hexaploid genome sequencing of sweetpotato Taizhong6 [37]. Additionally, the number of 72 *IbGRAS* genes is more than that in *Arabidopsis* (32) and rice (57) [16]*,* barley (62) [17]*,* cucumber (35) [20], tomato (53) [22]*,* and tea (52) [23], but less than in sorghum (81) [18], soybean (117) [21]*, Brassica napus* (87) [14], and cotton (150) [25], suggesting the significant divergence of GRAS genes among the plants of monocot and dicot. Moreover, although 72 *IbGRAS* genes were mapped on all 15 chromosomes, the numbers of GRAS genes are irrelevant to the chromosome size (ranging from 1 to 10). Similar disproportionate distributions have also been found in *Arabidopsis* and rice [16], soybean [46], *Ipomoea trifida* [38], and tomato [22]. Differently, no *SbGRAS* genes were observed on Chr7 and no MeGRAS members were distributed on Chr16, which may be due to fragment loss or chromosome translocation during evolution [18, 47].

Gene structure analysis showed that about 82% of *IbGRAS* genes were intronless or contained only one intron, which was similar to the GRAS genes in *Arabidopsis*, rice, sorghum, *Ipomoea trifida*, soybean, and *Populus* [18, 38, 39, 46]. Intron-less genes have also been observed in several other gene families, such as the DEAD-box RNA helicases [48] and SAUR genes [49]. Previous report suggested that the plant GRAS gene family might have originated from prokaryotes mainly through horizontal gene transfers and duplication events in evolution [50]. Nonetheless, several *IbGRAS* genes showed obvious exceptions with more than 5 introns, suggesting that the high degrees of divergence among the *IbGRAS* genes. These gains or losses might be the results of chromosomal rearrangement and fusion, and might result in the functional diversifications of gene families [51]. Introns can elevate the length of genes and the frequency of gene recombinations, although intron-less genes have no such advantages in species evolutions or gene recombinations, they tend to respond quickly to stress [18, 52]. Therefore, many *IbGRAS* genes may respond quickly to environmental conditions.

Genome duplication events are critical driving forces for the evolution and expansion of many plant gene families, which can promote the emergence of new functional genes and species, so that plants can more tolerate adverse environmental conditions during evolution [40, 53]. Previous reports in *Arabidopsis* and rice [16], tomato [22], soybean [46], sorghum [18], and *Populus* [39] exhibited that segmental duplications or genome duplication events might explain the expansion of plant GRAS gene family. Similarly, many *IbGRAS* genes were identified as tandem duplications and segmental duplications by collinear analysis, indicating that some *IbGRAS* genes may be emerged by gene duplications in sweetpotato, further supporting this common mechanism that leads to GRAS gene expansion. And the contributions of tandem duplications to the increase of *IbGRAS* genes are similar to that of segmental duplications. Additionally, the *IbGRAS* genes exhibiting tandem repeat and segmental duplication events are members of the same subgroup, specially, most *IbGRAS* gene pairs were from the LISCL subgroup. The results are similar to the GRAS genes in grapevine and sorghum [18, 54], suggesting its critical evolutionary roles in gene expansions. Therefore, this indicates that the retentions of gene copies are somewhat biased, and there are differences in the retentions and losses of different subgenomes. Previous findings showed that if some proteins interact with other products encoded by genes, the genes will be biased post a replication event [55].

Besides, the synteny analysis assessing the relationship between *IbGRAS* genes and the counterparts from nine plants was analyzed, including *Ipomoea triloba*, model plants *Arabidopsis* and *rice*, representative Solanaceae, Brassica and cereal plants. Among them, the number of orthologous genes identified between sweetpotato and *Ipomoea triloba* was the largest, supporting their close evolutionary relationships, followed by tomato, pepper and *Arabidopsis*. These genes may be derived from the common ancestor [18]. Moreover, the complicated relationships such as single *Ipomoea triloba*-to-several *IbGRAS* genes were observed, implying that these members in *Ipomoea triloba* might play important roles in the evolution of *IbGRAS* genes. No orthologous gene pairs were found between sweetpotato and the detected cereal plants, probably because of enormous chromosomal rearrangements or fusions in their genomes [56]. Further, we found that multiple GRAS genes were only retained in several plants, similar result was also found in sorghum [18]. These findings might be associated with the phylogenetic relationships between sweetpotato and the nine plant species. And large-scale duplication events predate the divergence of some plant species and play important roles in the expansions of GRAS gene family.

Phylogenetic analysis showed that sweetpotato GRAS TFs were classified into 12 subfamilies, and at least one IbGRAS protein was identified in each subfamily of *Arabidopsis*, suggesting that the divergences of GRAS genes might be earlier than that of monocots and dicots [18], while several new subgroups and members were produced as evolution proceeded. The classifications of IbGRASs were similar to the reports in *Sorghum bicolor*, *Brassica napus* and *Medicago truncatula* [15, 18], but were different from the reports of eight subgroups in woad, tomato and Chinese cabbage [14]. Interestingly, IbGRAS48 and IbGRAS72 did not belong to any of the 12 subfamilies, indicating that they might have unique functions. Among them, LISCL had the most GRAS members, which was similar to the reports in many other plants, including *Arabidopsis*, rice, and *Populus* [39], sorghum [18], *Ipomoea trifida* [38] and soybean [46], suggesting that the gene family may have strong partial differentiation abilities in the long-term evolution processes. The classifications of IbGRASs were also supported by their conserved motifs, especially the close IbGRASs from the same subfamilies generally contain similar motif compositions. It is worth mentioning that multiple motifs exist in specific subgroups, implying that they may have specific functions, because GRAS TFs performing varied functions have been widely reported [7, 11], and many domain-loss events were detected in multiple IbGRAS members. For instance, the N-terminus of the members from DELLA subfamily contains the DELLA domain that may interact with the GA receptors to sense GA signals [57], which may lead to the diversifications of GRAS gene family and affect their functional differentiations.

The function of GRAS TFs as key participants in modulating the response of plants to multiple adverse environmental inputs has been increasingly documented [7, 30], illustrating that GRASs are promising candidates for enhancing crop stress tolerance by molecular breeding. For instance, overexpression of *OsGRAS23* enhanced drought and oxidative stress tolerance of rice via regulating stress-responsive genes [32], and *PeSCL7*-overexpressing *Arabidopsis* exhibited drought and salt tolerance [33]. *SlGRAS6*-silenced tomato displayed decreased tolerance to drought stress [58]. Presently, the roles of sweetpotato GRAS genes in regulating stress response are still poorly understood. In this study, our transcriptome data and qRT-PCR results showed that most of the detected *IbGRAS* genes displayed obvious differential expression under a variety of abiotic stresses, indicating that sweetpotato *IbGRAS* genes may also play critical and diverse functions in response to environmental stresses. For example, the expression of multiple *IbGRAS* genes, particularly *IbGRAS2*, *IbGRAS58* and *IbGRAS71*, were remarkably induced under various abiotic stresses. And stress hormone ABA could significantly induce the transcription of *IbGRAS4* and *IbGRAS16*, the results suggest that these *IbGRAS* genes may function as promising participants in stress/hormone response. Previously, *Brassica rapa* GRAS TF BrLAS was found to participate in drought stress tolerance via an ABA-dependent signaling pathway [36]. Additionally, the transcription of several *IbGRAS* genes could be simultaneously upregulated by at least two abiotic stresses, implying that they might play conserved functions in response to these stresses, while further experimental verifications are required. Furthermore, the potential roles of *IbGRAS* genes in stress tolerance were further supported by phylogenetic tree and cis-element analysis. Functional characterizations of GRAS genes have suggested the conserved functions of putative orthologues in each subgroup [14]. For example, the LISCL subgroup member SCL14 of *Arabidopsis* can interact with TGA TFs and is necessary for activating the stress-inducible promoters [13]. Therefore, the *IbGRAS9*, *IbGRAS21* and *IbGRAS31* genes belonging to the LISCL subgroup were also significantly induced by multiple stresses, and therefore may be involved in the regulation of stress response pathways. Besides, many stress- and hormone-associated cis-elements including the MBS, LTR, ABRE, TCA-element were found in the promoters of most *IbGRAS* genes. The findings were consistent with the previous reports of the GRASs in *Ipomoea trifida* [38], *Brassica juncea* [24], *Cucumis sativus* [20] and *Glycine max* [46]. Particularly, our data suggest that IbGRAS71 protein has transactivation activity in yeasts, which were also consistent with multiple previous results in the GRASs from rice and *Brachypodium distachyon* [19, 59]. However, the biological roles of most sweetpotato IbGRASs remain to be undefined.

The conserved GRAS domain is pivotal for the dimerizations of GRAS members and other proteins [7], the STRING database predictions indicated that sweetpotato *IbGRAS* genes might take part in stress tolerance or growth and development through a complex protein interaction network. The homologous gene *GAI* of sweetpotato *IbGRAS*-*37*/-*44*/-*62* in *Arabidopsis* was reported to be involved in reducing ROS accumulations in response to stress, and GAI could interact with multiple GRAS proteins including PAT1 (IbGRAS-2/-47/-65/-66/-69/-71), SCL3 (IbGRAS-5/-10/-18/-63) and RGA1 (IbGRAS-30/-48), indicating that the counterparts in sweetpotato may tend to form similar protein complexes. Further Y2H experiments confirmed that IbGRAS71 could interact with IbGRAS4 and IbGRAS9, and IbGRAS4 could also interact with IbGRAS9 and itself, suggesting a complex interaction relationship between sweetpotato IbGRAS proteins. Besides, protein phosphorylations are critical post-translational modifications in modulating TF activities. For instance, reversible phosphorylations are required for the stress-induced expression of *NtGRAS1* by employing the inhibitor of protein kinases and phosphatase actions [31]. Our results exhibited that the IbGRAS proteins had 25 to 152 phosphorylation sites, indicating that they might act through potential post-translational phosphorylation modifications. Collectively, these results suggest that multiple stress-responsive *IbGRAS* genes may play diverse and pivotal roles in regulating abiotic stress signaling cascades via a potential complex interaction network.

## Conclusions

In this study, 72 *IbGRAS* genes were identified in cultivated sweetpotato and were unevenly distributed on all 15 chromosomes. Most *IbGRAS* genes were intron-less, and phylogenetic analysis suggested that these IbGRASs were classified into 12 subgroups. Gene duplication survey showed that both tandem duplication and segmental duplication events contributed to the expansion of GRAS gene family in sweetpotato, and collinearity analysis of orthologous genes from nine typical plants provided important clues to the evolutionary characteristics of sweetpotato GRAS genes. The stress-responsive *IbGRAS* genes were screened through RNA-seq analysis, and the diverse and significant expression profiles of *IbGRAS* genes were detected under various abiotic stress and hormone treatments by qRT-PCR assays. Particularly, multiple IbGRAS members, such as *IbGRAS2*, *IbGRAS58* and *IbGRAS71*, may hold crucial roles in stress response. In addition, IbGRAS71 protein was tested to have transactivation activity, and a complex interaction relationship between IbGRASs was detected. These results will facilitate to understand the complexity of GRAS gene family and their promising roles in sweetpotato response to environmental stresses.

## Methods

### Genome-wide identification of IbGRAS genes in sweetpotato

The full genome sequence and annotation data of *Ipomoea batatas* were obtained from *Ipomoea* Genome Hub (https://ipomoea-genome.org) [37]. And all the GRAS gene information in *Arabidopsis* and rice was downloaded from TAIR (https://www.arabidopsis.org/) and Rice Genome Annotation Project (http://rice.plantbiology.msu.edu/) based on the previous report [16]. To single out all the possible GRAS genes in sweetpotato, all the *Arabidopsis* and rice GRAS sequences were used as inquires to perform the BLASTP search against all the protein sequences of *Ipomoea batatas*. Afterwards, 77 candidate protein sequences were screened, and the Pfam database (http://pfam.xfam.org/), online batch CD-search program (https://www.ncbi.nlm.nih.gov/cdd/Structure/cdd/wrpsb.cgi) and PROSITE database (https://prosite.expasy.org/) were employed to verify each candidate non-redundant GRAS member to exclude those lacking a typical conserved GRAS domain. The sequence information of putative IbGRAS proteins can be found in Additional file 10.

### Protein property, exon–intron structure and cis-element analyses of IbGRAS gene promotors

The online ExPASy tool (http://expasy.org/) was used to investigate the suppositional molecular weight (Mw) and theoretical isoelectric point (pI) of 72 IbGRAS proteins. The Plant-mPLoc software (http://www.csbio.sjtu.edu.cn/bioinf/plant-multi/) and NetPhos 3.1 Server (http://www.cbs.dtu.dk/services/NetPhos/) were employed to predict their subcellular locations and phosphorylation sites, respectively. The intron–exon structures of *IbGRAS* gene*s* were generated by comparing their coding sequences and genomic sequences, and the result was illustrated by Tbtools [60]. To determine the potential hormone- and/or stress-related cis-elements in the promoters of 72 *IbGRAS* genes, the 2.0 kb promoter regions of each *IbGRAS* were extracted from *Ipomoea* Genome Hub and then submitted to the plantCARE database (http://bioinformatics.psb.ugent.be/webtools/plantcare/html/).

### Chromosomal location and collinearity analysis of sweetpotato IbGRAS genes

The physical position information of 72 *IbGRAS* genes on sweetpotato chromosome was identified according to the GFF annotation information obtained from *Ipomoea* Genome Hub. For the synteny analysis between *IbGRAS* genes and the genes from other plant species, the genome sequence and annotation information of *Ipomoea batatas*, *Ipomoea triloba*, *Arabidopsis thaliana*, *Oryza sativa*, *Solanum lycopersicum*, *Capsicum annuum*, *Brassica rapa*, *Brassica oleracea*, *Triticum aestivum* and *Zea mays* were downloaded from multiple databases including *Ipomoea* Genome Hub, TAIR, Ensembl (http://plants.ensembl.org/index.html) and Phytozome (https://phytozome.jgi.doe.gov/pz/portal.html). The gene duplications and collinearity relationships were generated using the Multiple Collinearity Scan toolkit (MCScanX) through the default parameters [61], and circos and TBtools softwares were applied to visualize the results, and the minimum block size was set to 30 [60, 62].

### Analysis of phylogenetic relationships, conserved domains and protein interacting networks

For phylogenetic analysis, well-classified AtGRAS proteins in *Arabidopsis* [16] and all IbGRAS proteins in sweetpotato were used to construct the un-rooted phylogenetic tree through MEGA-X software using Maximum Likelihood method [63]. The parameters were as as follows: the best evolutionary model JTT + G + F with bootstrap value of 1000, and the phylogenetic relationships of 72 IbGRAS proteins were also constructed by the same parameter. MEME 5.3.3 (https://meme-suite.org/meme/tools/meme) was applied to generate the conserved domains [64] with maximum number of 19 based on the previous settings in *Arabidopsis* and rice [16]. Subsequently, the potential protein interacting network was performed via STRING 11.0 (https://string-db.org/).

### Salt-responsive IbGRAS genes were identified by transcriptome analysis

The salt-tolerant sweetpotato cultivar XuShu 22 and salt-sensitive sweetpotato cultivar XuShu 32 were obtained from the Xuzhou Sweetpotato Research Center, China. No permissions were necessary to collect the plants. The adventitious roots of the two cultivars were treated with salt stress, and then collected for RNA-seq detection by Illumina HiSeq 2500. The RNA-seq data displayed a high expression correlation (R^2^ ≥ 0.897) except Xu22-CR2, thus the assembled sequences (except Xu22-CR2) were used for downstream analysis. Whereafter, gene expression levels were calculated by read counts using false discovery rate (FDR) and Log2 (fold change) as descripted before [41]. The annotations of genes according to several databases such as *Ipomoea* Genome Hub, Nr, Pfam, and SwissProt, etc. [65].

### Abiotic stress and hormone treatments of sweetpotato and qRT-PCR detection

The treatments of XuShu 22 seedlings by abiotic stress and hormone as descripted in our previous publication [66]. Simply, the seedlings were cultivated in a growth chamber under sodium lights timed for 16 h days (25 °C) and 8 h nights (20 °C). Uniform plants with fibrous roots about 9 cm long were employed, salt and drought treatments were conducted by submerging the roots in 150 mM NaCl and 20% PEG6000, respectively, then roots were collected. Cold and heat treatments were imposed by transferred plants into an incubator at 4 °C and 42 °C, respectively, then leaves were harvested. The hormone treatment was carried out by spraying 0.1 mM ABA, ACC and JA solutions on the seedlings, then leaves were collected. Untreated seedlings were used as controls, and the samples were collected at 1, 12, 24 and 48 h post each treatment with three independent biological replicates.

To validate the RNA-seq data, total RNA of all the collected samples was extracted by using an RNA Extraction Kit (TianGen, Beijing, China) based on the manufacturers’ instructions. 1 μg RNA of each sample was reverse-transcribed using TransScript® one-step gDNA removal and cDNA synthesis supermix (TransGen, Beijing, China). qRT-PCR assay was conducted by a CFX96™ Real-Time System (Bio-Rad, USA) as descripted before [6]. The sweetpotato *ARF* gene (JX177359) was applied as the internal control [67]. All the specific primers for qRT-PCR detection are listed in Additional file 11.

### Analysis of transactivation activity and protein interaction of IbGRAS proteins in yeast

The open reading frame sequences of *IbGRAS-2/-4/-9/-71* genes were separately cloned into the pDONR207 vector through BP clonase (Invitrogen), then were fused into the pGADT7 and pGBKT7 vectors, respectively, by the LR reaction (Invitrogen). Then the pGBKT7 control, recombined pGBKT7-*IbGRAS* plasmids, and both recombined pGBKT7-*IbGRAS* and pGADT7-*IbGRAS* vectors were transformed into Y2HGold yeasts as descripted before [68]. For transactivation detection, the yeast dilution was dropped on SD/-Trp (SDO), SD/-Trp-His-Ade (TDO) medium with or without 200 ng/mL AbA (Aureobasidin A). For protein interaction detection, the dilutions were dropped on SD/-Trp-Leu (DDO), SD/-Trp-Leu-His-Ade (QDO) medium with or without 200 ng/mL AbA. All the plates were cultivated at 30 °C for 3 d to check their transactivation activities or protein interaction. The primers applied for gene cloning and vector construction are presented in Additional file 11.

### Statistical analysis

Considering the biological significance, a cut-off value of two-fold for differential gene expression was adopted [42]. OriginPro 8 software (SAS Institute) was used to generate graphs.

## Supplementary Information

**Additional file 1: **Characteristics of five excluded* Ipomoea batatas* proteins obtained by BLASTP search using the GRAS sequence information in *Arabidopsis* and rice.

**Additional file 2: **Schematic representations of the chromosomal distribution of the 72 *IbGRAS* genes on 15 sweetpotato chromosomes.

**Additional file 3: **Accession numbers of GRAS genes in sweetpotato and *Arabidopsi*s.

**Additional file 4: **Chromosomal locations and segmental duplications of *IbGRAS* genes in sweetpotato.

**Additional file 5: **Orthologous genes between sweetpotato and *Ipomoea trilob*a.

**Additional file 6: **Venn diagrams among the detected species with orthologous genes of sweetpotato *IbGRAS* genes.

**Additional file 7: **Phylogenetic relationships and distributions of amino acid motif compositions within the IbGRAS proteins identified by MEME.

**Additional file 8: **Differentially expressed *IbGRAS* genes in sweetpotato transcriptome analysis under salt stress. CR, Control roots; SR, Salt-treated roots.

**Additional file 9: **Cis-elements associated with hormone and abiotic stress within the *IbGRAS* gene promoters.

**Additional file 10: **The nucleotide sequences and amino acid sequences of 72* IbGRASs* identified in sweetpotato genomes.

**Additional file 11: **Specific primer sequences used for gene cloning, qRT-PCR analysis and vector construction.

## Data Availability

Most data generated or analysed during this study are included in this published article and its supplementary information files. The open RNA-seq data (accession numbers SAMN14884352-SAMN14884363) used and analyzed during this study are available in the NCBI database.

## Abbreviations

Aa: Amino acids
ABA: Abscisic acid
ABRE: Abscisic acid responsive element
bZIP: Basic leucine zipper
ACC: 1-Aminocyclopropane-1-carboxylate
JA: Jasmonic acid
Mw: Molecular weight
pI: Isoelectric point
qRT-PCR: Quantitative reverse transcription-PCR
TF: Transcription factor
