# Supplementary material for: Genome-wide survey and expression analysis of GRAS transcription factor family in sweetpotato provides insights into their potential roles in stress response
Source: BMC Plant Biol. 2022 May 6;22:232. doi: 10.1186/s12870-022-03618-5 (PMC9074257; doi:10.1186/s12870-022-03618-5)
Supplement: Supplementary file 1 — Additional file 1: Characteristics of five excluded Ipomoea batatas proteins obtained by BLASTP search using the GRAS sequence information in Arabidopsis and rice. [file 12870_2022_3618_MOESM1_ESM.docx]

Additional file 1. Characteristics of five excluded *Ipomoea batatas* proteins obtained by BLASTP search using the GRAS sequence information in *Arabidopsis* and rice.

| Gene ID | Amino acids | MW (Da) | PI | Subcellular  location | No.of phosphorylation cite | | | |
| --- | --- | --- | --- | --- | --- | --- | --- | --- |
|  |  |  |  |  | Ser site | Tyr cite | Thr cite | Total |
| g28735.t1 | 152 | 17426.12 | 10.5 | Nucleus | 9 | 0 | 3 | 12 |
| g28745.t1 | 56 | 6707.69 | 4.94 | Nucleus | 2 | 0 | 2 | 4 |
| g37660.t1 | 1219 | 134746.51 | 6.74 | Nucleus | 69 | 17 | 29 | 115 |
| g51195.t1 | 141 | 15538.17 | 6.59 | Nucleus | 7 | 0 | 3 | 10 |
| g55184.t1 | 117 | 13738.43 | 6.18 | Nucleus | 2 | 0 | 1 | 3 |
