# Supplementary material for: Genome-wide survey and expression analysis of GRAS transcription factor family in sweetpotato provides insights into their potential roles in stress response
Source: BMC Plant Biol. 2022 May 6;22:232. doi: 10.1186/s12870-022-03618-5 (PMC9074257; doi:10.1186/s12870-022-03618-5)
Supplement: Supplementary file 2 — Additional file 2: Schematic representations of the chromosomal distribution of the 72 IbGRAS genes on 15 sweetpotato chromosomes. [file 12870_2022_3618_MOESM2_ESM.docx]

## Additional file 10. The nucleotide sequences and amino acid sequences of 72 IbGRASs identified in sweetpotato genomes.

72 nucleotide sequences

>IbGRAS1

ATGTTAGGCTCATTTGGTTCTTCATCCTCTTCCAGATCCCTCGACGAACAGGATACTACTAATAATCATAATAATAATGAGCCGCCTTCATCTGTGGTGGCTCCGTTGCCGCCGTTTCAGTTCCACCAGATTCAACGCCGGAGGACTGTCTTCGCTTCTTCTTCTTCTTCTCCTCCGGCGATTCAAATGCGGCAATTCCTCATTAGCTGCGCTGAGTTAATCTCGAGATCTGATTTCTCCGCCGCTCATCGTCTCATCTCATTTTTGGCGTCTAATACTTCCCCTTTTGGGGATTCCAGTGAGAGATTGGCGCATCAGTTCACTCGCGCCCTTTCTCTCCGCCTCCACCGGAGCATCTCCGCCTCCGCCGCTTCGGCCGTTGCCCCGCTACCGTTTCCTGAGCTTCAGATGGAACAACCGGGGCTTGTTCAGTCCTCCTATTTGTCTCTCAACCAAATCACGCCGTTCATAAGGTTTACTCATCTCACCGCGAATCAGGCGATCCTGGAAGCGATCGACGACGGTCAACGAAATGCCGTTCACATCCTTGACTTCGACGTCATGCACGGCGTTCAGTGGCCGCCCTTAATGCAAGCCCTAGCGGATCGCTACCCTCCCCCGACGCTCCGGATCACCGGCACCGGAAATGACCTGGACATCCTACGGAGAACCGGCGACCGTCTCGCCAAATTCGCTCATTCCTTAGGGTTGAGGTTCCAATTTCACCCTCTCCTCCTCAGCAACAACGAAGATGACGAAGACGTCGACCCTCCTCTATCCTCCGTCGTTCTACTCCCGGACGAAACCCTAGCCGTGAACTGCGTGCTTTACCTCCACAGACTCCTGAAGAATCGCGAGAAGCTCAACCGTTTTCTTCACAGAATCAAAAGCATGAGGCCCAGGGTGGTCACCGTGGCGGAAAGGGAAGCGAGCCACAACAGCTCTTGCTTCCTCCAGCGGTTCGTGGAGGCGCTGGATCACTACGCCGTGGTGTTCGACTCCCTGGAAGCAACGCTGCCGCCCAGCAGCCGGGAGAGGGTGGCGGTGGAGCAAGTCTGGTTCGGGAGAGAGATTGTGGACATTGTGGCGGCGGAGGGAGAGAAGAGGAAAGAGCGGCACGAGAAATTCCGAACATGGGAGATGTTGTTGAGGAGCACCGGTTTTAGCAATATCGGTTTAAGCCCTTTTGCTCTGTCGCAGGCAAAACTTCTTCTCAGACTCCATTACCCTTCAGAAGGTTACCAGCTCCGTGTCATCGCCGATTCTTTCTTCCTCGGCTGGCAAAACCATCCACTTTTCTCTGTCTCTTCTTGGCACTGA

>IbGRAS2

ATGGACTCCCATCAGTTTTTTGGATATGGTGTAACTGGAGCGGATTTTGCATACTCTTCGTATCCCGATATTCCTTCAATACCTATTAGGCATATTGGATCCTTAAAATTTGACTCAAGAACTTCTCCGAATTCGCCTTTTGCACCTCACTTCGATCCTCAGACCCCGACCTCTCTAAGCGACAGCCAGGAGCAACAGAGCTCGACTGATAATCTCTCGGGAGTCAGTGCTTCGAGTAATTCTCTGCTGGATTATAGCAGTTACTTCCAACAGCGTAGCTCCCCGTTGGATTGCGGTCCGGAAAGTCTGCTAATTCCTTCTGGTGCGACTTCTTTTCATCGGAATCAGAAGGCGAAACATGTTTTCTGGCAGGTGGAGCCTGCTTTGATTGTGCCAGATCAAACGGGGGCCAAAACATCAGATCTGTCCTTGAGTGAAAATAAGCACCAACAACCGTTAGGCCAGAAGTCTAGGTCTTGGAGCCAGGAAGGCCAGATTTCGGGCCTGATTGAATTTCAGCCGTCTCGTGTCTCGTCAATTGGAAAACCCGACGGGAGTGTTCATAGCCTAAAACGCTACAAAACTGTGCAGGACTTTCCCTTGCAGGGCGTTCCTCAGGGTAATATAAAACAGCTTCTCATTTCTTGTGCTCGAGCTCTACTTGCTGAAAATAAAGATAATTTCGATAGACTGGTTGAAGAAGCGCGCGTTTCTGTGTCCATCACCGGGGATCCTATTCAGCGCCTCGGAGCTTACATGATAGAAGGGCTAGTTGCAAAGAAAGAGGCATCGGGTGCAAACTTTTATCGGGGTTTGAAGGGTAAAGAACCGGCAGGGAAGGACTTACTCTCCTACATGCACATCCTGTATGAATTATGCCCTTATCTCAAGTTCGGTTATATGGCTGCAAACGGTGCCATAGCAGATGCGTGCAGAAACGAAGACCGCATTCACATTATCGACTTCCAAATCGGCCAAGGGACTCAATGGATGACTCTTCTACAAGCACTTGCTGCAAGACCTAGCGGTGCCCCTCACGTGAGAATCACGGGGATTGATGACCCGGTTTCGCAATATGCTCGGGGAGATGGTTTAGCAGCCGTGGGGAAACAGCTAGCTGCAATTTCCGAGAAGTTCAGTATCCCGGTTGAGTTTCATGCAGTCCCGGTTTTCGCCCCAAAAGTCACCCGGGATATGTTGGATATCCGGCCTGGCGAGTCACTAGCTGTGAACTTCCCTTTTCAGCTCCACCACACTCCCGACGAGAGCATAGACATGAACAATCCACGGGACGGTCTTCTTAGGTTCGTGAAGTCGCTCTCCCCCAAGGTAGTCACTTTGGTGGAGCAAGAATCGAACACAAACACCGCCCCGTTCTTCCCGAGATTCTTGGAAACTCTGGACTACTACTCAGCAATCTTCGAGTCAATAGACGTGACCCTAGCAAGGGACAAGAAGGAGCGGATCAACGTCGAGCAGCACTGTCTCGCACGAGACATCGTCAACGTCATAGCTTGCGAAGGCCAGGAGAGGGTGGTGCGCCACGAGCTATTAGGCAAGTGGAAATCCCGGTTCACAATGGCAGGATTTCGCGAGTACCCTCTCAGCTCCTACGTTAACTCCGTAATTAAAAGCCTCCTGAAGTACTACTCCGAGCACTACACGCTCGTGGAGAAAGACGGGGCCATGCTGCTCGGGTGGAAACAGCGCAACCTCATCTCCGCTTCAGCTTGGCATTGA

>IbGRAS3

ATGGAGTTTGAGAGTTTTGATGAGGTTAGCCTTTCTCCATCCTACATTCATGAGTTTCAAAAACATGGGTCGAAAAACAGCTCATTTACTTCTTGGGTGGTAGATTATGGGCAAGAATACGGGTTGGAAGCTGGCACAGATAGCTTCGATTCTTGGATAACTGATTGTGGACAAGAATATGGGTTGAAAGTCCACACGGGTGGTTCTGTTGGTTCGTGGGTGACAGAATGTGGACAAGAATATGAGTTAGAAGTCTGCACGGATAACTCAGTTGGTTCTTGGGTGACAGATTATGGACAGAAAGACCAGTACTACTCGAACTCCAACGATGTGGACATTCAAGAATGTCTCCAACTAGAGACAGACATCATGGACCTCGATTTCATCGACGAAGATGCTGTCTTTTCCCAGAACAGGGAGCAAGAAACTCCAACGGAAAAGGCCGGCGTCTCTGGCTTCTTAACCCCAGAAAATTACACCTCCCATCTCAAAACCATCCAAGAAGAACTCATGGAGGAAACCAGCCTAACTGATCTTCTCCTGATGGGAGCCCAAGCTATCCAGGCCGGGAACAAAGATCTAGCCTCAATGATAGTCCTCAAGCTCAATAATCCCCAGATTTCCAGCGAAGAAGAAACAGGGCATCTTGATCGCCTAGCTCTTTACTTCACTCAAGGATTGGCCCTAAAAACCATGAATTTTGGGGATCAGAATTGGCAATTGGGATCAATGTCGGCTTTCCAAATGCTCCAAGAAATCTCCCCCTACATAAAATTCGCCCATTTCACCGCAAACCAGGCCATCTTAGAAGCCACCCAGAGCCAAAGAGATGTTCATATAATCGATTTCGACATCATGGAAGGAATCCAATGGCCGCCATTAATGGCGGAATTAGCAGACAGGGAAAACGCGTCTCTCAGAATAACCGCCATGGTTACAGAGCCCCAAACCTGGGCTCATACTCAACAAACAGGGAAACGGCTACACGAATTCGCGAACTCAATTAATCTTTCCTTCTCATTTGATCAGATTTTACTAACCAAAGAGCAAGATTTGGAGCAAATTCAGAGCCTGGGGAATAACCCAATCGCCAATTTAATGATCCACCAGCTTCACATCCCACACAGAGAGAGCTCACTAATCAAGATTTTCCTAAACGGGCTCAGAAATCTCTCCCCACAGATGATAATCATGGTGGAAGAAGAGCTCTTCAACATTTCAAGAACCCCATCCATGTCGTTCGTGGAGTTTTTCAGGGAAGCCATTCATCATTACACTTCCCTATCCGATTCTCTGCAGGGAGGGTTCTGTGGGGGGTACAAATTGGCGCAGAGGGTCATAGAAAAAGAGTTTCTTGGCCCCAGAATTGTGGACTGTGTGAGGCAATTCCCGTCTGGGAAAAGAGAGAGACAAATATGGAGTCAAGGGGTGTATTCCCTAAAAGGGTTTAAGGCGATTCCGATGAGTTCTTGCAATGTTAGCCAGGGTAAGTATCTTGTGAGCCTCTTCAATGGAGGTTTTTGGGTGCAGCATGAAAAGTGCAGGCTTGCCCTGTGCTGGAAATCACGGCCGTTGATCACTGCCTCCATTTGGGTTCCCACATCATCATCAATTAGTCACAGTCTTTCAAGGTCAACTTCTTTCGATTGA

>IbGRAS4

ATGGATAATAATGAAGACGACTTTTCTTCTTCCTCTTATAATAATACCCACTTCTCTGATTACTCTCACCATCAAACTCCCATCGATCATAATTTCTCCTTCTCCCCGGGTTTTGATTCCGGGTCGGCTTTTTCCGGCGACGATATTCTGCTCCACGCCGCCAGAGCCGTGGCCGACAAGAACGCCCCGCGCGTGCGGCAGTTAATATGGGTGCTCAACGAGATCAGCTCTCCGTACGGCGACACCGATCAGAAGCTGGCGTATTACTTTCTCCAGGCGCTGTTTAGCCGCGTAACGGATTCCGGCGAGCGGAATTGCCGGAAGTCGGCGTCGGCGTCGGAGAGGAATTGCTCGTTCGAGTCGACGAGGAAGACGGCGCTGAAGTTCCAGGAGGTGAGCCCGTGGACCACTTTTGGCCACGTTGCGTGTAACGGAGCGATCATGGACGCCATTGACGGCGAATCCAAGATTCATATAGTTGATATCAGCAATACTTTTTGCACGCAGTGGCCTACTTTTCTTGAATCGCTCGCTACCCGAACTGACGACCCGCCTCGTCTACGGCTCACCACGGTGGTGGTCCGGAAACTGGGCGGCGGCGGTGGGCCGCCTGCGGCGGTTCAGACGGTGATGGAAGAGATTGGGAGTAGGATGGAGAAGTTTGCTAGACTTATGGGTGTGCCCTTTAAGTTCAACGTTGTATATCATACAGGTGATCTATCTGAATTCGATATTCTTGGATTGGATGTGGAAGAAGATGAAGCATTGGCAATAAACTGCGTGGGGGCGTTACATTCCGTCGCCAACAGCCGGCGGGGCCAAATGATCTCGACTTTCCGGCGATTACAGCCGAGGGTTTTGACGGTGGTGGAGGAGGAGGCTAATCTTGACGTGGGCGTTGATGGGTCCGACTTCATCACCGGTTTTCAAGAATGTTTGAGGTGGTTTAGGGTTTATTTCGAGTGTTTGGAGGAAAGTTTCCCGAAAACGAGCAACGAGCGTTTGATGTTGGAGCGGCAGGCCGGACGGGCGATAGTGGACCTAGTGGCGTGCCCGCCGTCGGAGTCGGTGGAGCGACGCGAGACGGCGGCGCGTTGGTCACACCGCCTCCATGCAGGCGGGTTCAGCCCCGCTTCATACGGTGATGAAGTGTGCGATGACGTACGCGCCCTTTTGAGGAGGTACAGAGAAGGATGGTCAATGGGACAGTGCTCCGGGGAATCTTCTGCCGGAATATTCTTGTCCTGGAAGGATCAGCCGGTGGTGTGGGCTAGTGTGTGGAAGCCGGCATTGAGCGACGGCGATGGCCGGTGA

>IbGRAS5

ATGCAAGAGGAAGCAGACGAGGGAGAGAAGGGGCATGCAATCATATTCAAATTCCAATTACTAGATGTCAGCCTCACTTGTCTTAGAGTAGGTTTAATTTTGATCATGTCTGAAAATAATGGTATTAAACTCATCAAATTGTTGCTCACTTGTGCTTCTCATGTATCTTCCGGTGATCTTCACCAAGCTGATGTGTGCCTTCGACAAATTTCCCAGTCTAATTCTGCCAGTAATGATTCCATGCATCGATTGGCAACTTGGTTTGCTTCTGCCTTGGCGGTTCGATTAGTGAAGCGTTGGCCGGGTGTTTATAAGGCCTTGAATTGTAGCAGCCTAATGAAATTCGATCTTGATCGTGCACGGTCTATTTTCACAAAGGCCCTTCCTTATTTGGGCTTTGCATATGCAGTTATAAATAGAACTTTAGTTCAAGCCATGTTGGGAGAGCGAGTAGTCCATGTAGTTGATTTGGGCTCAAGTGACCAACAATTGTGGGTCCCATTTATGAGAATCCTTGCAACTTCACCCGATGGACCTCCCCATTTAAGAATCACTTGTGTTAGTAGCAACAAGACTGCGTTGGACAAACTTGGATCATGCCTAAAAAAAGAAGCCGAGCAATTAGACATGCCCTTTCAGTTCAACCCAGTCAATGTTCACCATCTTCGAGACTTGAATTTCAACTCAATCGTTAAGGTAAGATCAGGTGAAGCATTAGCTTTCATATCAGTACTAAATCTTCACGTCCTTTTAGCCGAGGACGATCGCATTGATGCACAATTTGGACTAAACAAGGACTCTAAAAACATTAAACATTGCAAACATGCTGATGAATTCCTAGCAAAGTTATGTTCTTTGTCACCAAAGTTAGTAATGTTGGTAGAGCAAGAATCTAACCATAACTTACAAAAATTTGTTGATAGATTTGTTGAGGGGTTACGATATTACAGCGCAATGTTTGATTCAATTAATGTTGCATTCAAGGGGGATTTATGTGAGGAGAGGTTGTTGGTGGAAGAAATGATTGGCAAAGAAATTGAAAACATTGTGGCATGCGATGGTTTAGAAAGAGAAGAAAGGCATGAAAAATTTGCTAATTGGATGGTTAGGTTAGGGCATGCCAAGTTTAGACCGGTAAGATTGTGGGGTGACACTATGGATGACGCTATAAGATTTGTAGAGTACGTACGGTGGAGATGGGTACAAGATTAG

>IbGRAS6

ATGATATGGAATATGGAAGGTGTAGTAGATGAGGAGTTGTTGAGCCTTAGACTAGGCATTGCCACAGGATCAAATGAGAAGAAAATTATGAGGAGGAAGAGAAAGAGGAGAGATCACGACGACTTTATCAACAACAATAGGCTTGTGTTGAGTGATGACGAAAGTTATGAGGGTCAAATCTTTAGTCTCCTTCAAACGAGAGAACAGATGTTGAAGGTAGAGAAAAGAGTCAGAGCGGTGACGGAGGAGGAGGAGAACGACAAAGGCGGCGGCCTTCATCTCATCCATCTCTTGCTAGTTTCTGCCACCGCCGTGGACGAAAATACCCCCGGCCCCGCCGCGGAGAGCCTCTCGGAGCTCTACCGGCGCGCCTCCTTGAGCGGGGACGCGGTCCAGCGCGTGGCGGCCTACTTCGCCGACGGGTTAGTGGCGCGTCTCCTCACCCGAAAATCCCCCTTCTACGACATGATCATGAAACCCCCTACCCCTCACGAACACCTCTTCGCCTTCACCCACCTCTACCGAGCCTCCCCCTTCCACCAATTCGCCCACTTCACCGCCAACCAAGCCATCATCGAAGCCTTCCACCAAGAATCCCAAACCAACAACGCCTCCCTCCACGTCATCGACTTCGACGTCTCTCACGGCTTCCAATGGCCTTCCCTCATCCAATCCCTCTCTCAAAGCCTCTCCCCACCCTCCAAAATATCCCTCAAGATCACCGGCTTCGGCCCAAGCCTCTCCCAGCTTCGGGAAACCGAAGCTAGGCTAGTGAGCTTCGCCAAGGGCTTTCGCAACTTATCCTTCGCCTTCACCGGGCTCTTACTCGACTCCATTGACTATTACACGTCAGCCAAAACCAAAACCAAAACCAAAACCCACGAAACCGTCGCCGTAAACCTCGCGTTTTACCTAAACAGATTACCCAATTTCTCCGACACTCTAAGAACCGTCCATCTCCTCAGCCCTTCCGTTGTGACTCTAATCGAACAAGAAGGGTGCAGAAGCCCTAGGAATTTCTTGTCGAGATTCATGGAATCCCTCCATTACTTCGCGGCCATGTTTGACTCGCTCGACGATTGTCTCCCCATAGACAGCCCCGAGAGGCTCAGCATCGAGAAGAACTATCTGGGGAACGAAATCAAGAGAGCGATGACGAACAATGGGAGTGAGGAATTGGTTCCCAGGTACGAGAAGATGGATACGTGGAAAGCGAGGATGGAGAGTCACGGGTTTTGTGGGGTCAGATTGAGCTGCAAAAACGTGATGCAAGCGAAGCTGCTGTTGAAAATCGCGAGTAGCGGCCATTGCTGTAGGGTGAGCTTTGATGGTGGGTTTAGGGTGTTTGAAAGAGACGATGGGAAGGCCATTTCTCTGGCTTGGCAAGATAAGCCTTTGACAACTGCCTCTGTATGGCGCTGTGTATGA

>IbGRAS7

ATGGCGAATGATGGAGTTTGTGAGACATTGGTGGATCGGAACGAAGAAATGGGAAATCTAAATAGTGAAGAAGCCCCACTGCCTCAATCCCATCAGAGGTTATTGAATGAAGATTATGATCTTAGGGATCAAATGCTCAAGTGGATTAATCAGATGCTAATGGAAGATAACGCGGAAGAGAAGGCCTACATGCTTCGACAGTCTGCTGCCCTTAAAGATGCAGAAAGATCATTTTACGAGCTTATTGGGGAGGAGTACCCGCTATCCCCGAATCTTCAAAGAGTTCCTGATTTGGATCGAAATGAAAACTATGGTAACGATGATTCGTTATGTCCTAACAGGGATCCCTATCCCCGGGAGCGTCAAACTACTAGTGACCATGTTGCTTCGAGTACTACTTTTCAGTCATCCTCATTGCTTGGCCAGGGCACCGAAAATGATGGGACTGTGGACTTTGCTGCCAGTTCAATTACTATTCCCGATTTTTCCAATTGTACTGAATCTGTTGAGAAAGGGGTTAGGGAAGCGAGTTCGTTCCTTCCTACTCGCAATAGCTTGCTTGTTGATGGAATTGGGGCTAAGAAGAATACTGGGAATCAGGACACCCTTGAAGGGCGAAGAGGAACGAAAAATACTCTTCGTGAAGATACGCATTTGCCAGAAGGGAGAAGTTACAAGCAATCAGCAATTTATGCTGAACCAAGCATTAAACAAGAAGAGTTTGACAAGGTGTTATTATGGAGTGGAGAGGATGAATCCAGTCTTTGTCATTCCTTGCGGGGCGTTTTGTGTGAAGGCAATGATGATTCGAAGGGTTCTAATAGGAAGAAATCGAGTGGAAAGAAACCGGGGTCTGAACGAACTGTGGTAGACTTGCGATCTCTCTTGATGCTTTGTGCACAAGCTGTAGCTGTTGAGGACATAAGGACTGCCAACGATTATTTGAAGCGTATCAGACAACATTCGTCTCAAACTGGAGACGATATGCAGAGGCTGGCGCATTATTTTGCTGACGGTCTCGAGGCAAGGATAGCGGGATCTGGGACGAGAATACATAAAGCACTGATGAAATATCCTAGGAAGATCTCGAATATGTTCTCGAACAAGACCATCACAACTCTAGCCCAGAACGCTTCATCACTGCACATTATCGATTTTGGGATTGGTACTCTTTTCGGCTTTCAATGGCCTTGCTTAATACAACATCTCTCGTCTAGGCCCGGTGGACCTCCCAAGCTTCGTATAACCGGGATTGATTTTCCACTGTCAGGTTTCCGGCCAGCAGAGAGGGCTGAGGGGACAGGGCGACTTTTAGCTTATTATGCTGAGAAGTTCAATGTCCCATTCGAGTTTAATGCTATAGCAAAGCAGTGGGAAACAATTACAGTCGAGGATCTGAAGATCATCGAGGGCGAGGTGCTTGCTGTGAACTGTCTATATCGACTTAGGAACCTTCTCGATGAAACAGTGGTTGTAAACAGCCTCAATCCGAGGGATACTGTTCTGAAACTCATTCACGATGTCCGTCCAGATGTTTTCATACACGGGATTCTCAACAGTGCTTTTCATGAGAGGATGCTGATCGAAAGCTACATATTTGGTCAGCAAGCCATGAACGCGATTGCATGTGAAGACACCGAGAGGGTCGAGAGGCCAGACACGTACAAGATGTGGCAGGCCAGGAACACGAGGGCTGGATTTCTTCAGCTTCCTTTGAACCGGGAGATCGTGAAGATGTCGATGCATATGTTGAAGCGATACCACAAGGAGTTCGTGATCGATGAAGATGGACACTGGCTGCTTCTGGGATGGAAGGGGCGCGCCATATTCGCGCTTTCATCGTGGAAACCAGCTTAA

>IbGRAS8

ATGGCGAATGAGGGAGTTTATGAGACATTGGTGGATCGGAACGAAGAAATGGGAAATCTAAATAGTGAAGAAGCCCCACTGCCTCAATCTGGTCAGAGGTTAAATGTAGTAAACTTATTGAATGAAGATGGTGGATATGAAGATTATGATCTTAAGGGTCAAATGCTTAAGTGGATTAATCAGATGCTAATGGAAGATAACGTGGAAGAGAAGGCCTACATGTCTCAACAGTCTGCTGCCCTTAAAGATGCAGAAAGATCATTTTACGAGCTTATTGGGGAGGAGTACCCGCCATCCCCAAATCTTCAAAGAGTTCCTGATTTGGATCGAAATGACAACATTGGATCGGGAGACAATGGTAACGATGATTCTTTATGTCCTAACTTGGATCCCAATCCCCGGGAGGGTCAAACTACTAGTGACCATGTTCCTGTCAATGTTGCTTCGAGTACAATTACAATTCCTGATATATCCAATTGTACTGAATCTGTTGAGAAAGGGGTTAGGGAAGCAAGCTCGTTCCTTCCTACTCGCAATAGCTTGCTTGTTGATGGAGTTGGGGCTGAGAAGAATACTGGGAATCAGGACGCCCTTGAAGGACGAAGAGGAACGAAAAATACTCTTCGTGAAGATACACATTTGCCAGAAGGGAGAAGTTACAAGCAATCAGCAATTTATGCTGAACCAAGCATTAAACAAGAAGAGTTTGACAAGGTGTTATTAAGCAGTGGAGAGGACGAATCCAGTCTTTGTCATTCCTTGCAGGGCGTTTTGTGTGAAAGTGCAACAGGCAATGATGATTCGAAGGGTTCTAATAGGAAGAAGAAACCGGGGTCTGAACGAACTTTGGTAGACTTGCGATCTCTCTTGACGCTTTGTGCACAAGCTGTAGCCGTGGAGGACATAAGGACTGCCAACGATTATTTCAAGCGTATCAGACAACATTCGTCTCAAACCGGGGACGATATGCAGAGGCTGGCGCATTATTTTGCTGAGGCTCTCGAGGCAAGGATAGCAGGATCTGGGACGAGAATATATAAAGTACTGATGAAATATCCTAGGTATGCTGCTAGGGCCTTGAAAGCTTTCCAGCTCTATCTTTCTTCGTGTCCATTTGTGAAGATCTCGTATTTGTTCTCAAACAAGACCATCACAACTCTAGCCCAGAACGCTTCATCACTGCACATTATTCATTTCGGGATTGGTTTTCTTTTCGGCTTTCAATGGCCTAGCTTAATACAACATCTTTCATCTAGGCCCGGTGGACCTCCCAAGCTTCGTATAACCGGGATTGATTTTCCACAGTCAGGTTTCCGGCCAGCAGAGAAGGCTGAGGGGACAGGGCGGCTTTTAGCTTATTATGCTCAGAAGTTCAATGTCCCATTCGAGTTTAATGCTATAGCAAAGCAGTGGGAAACAATTACAGTTGATGATCTGAAGATCATCGAGGGCGAGGTGCTTGTAGTGAACTGTATATATCAACTTAGGAAAGTTCTCGACGATACAGTGGTTGTAAACAGCCTCAGTCCGAGGGATACTGTTCTGAAACTAATCCACGAGGTCCATCCGGATGTTTTCATACACGGGATTCTCAACAGTGCTTGTAATTCCCCCTTATTTACATCGAGATTTCGAGCAGCTCTGTCTCATTACTCAGCTGTGTTCGATATGCTCGAGGTCACTATTCCCCGCGAAGTTCATGAGAGGATGCTGATCGAGAGCTACATATTTGGTCAGCAAGCCATGAACACGATTGCTTGTGAAGACACTGAGAGGATCGAGAGGCCAGAAACGTACAAGATGTGGCAGGCCAGGAACACGAGGGCTGGATTTCTTCAGCTTCCTTTGAACCGGGAGATCGTGAAGATGTCGATGCATACGTTGAAGCGATACCACAAGGAGTTCGTGATCGATGAAGATGGACACTGGCTGCTTCTGGGATGGAAGGGGCGCGCCATATTTGCGCTTTCGTCGTGGAAACCGGCTTAG

>IbGRAS9

ATGGATAGGGGACCATATGAGGCCACATCCCAATTTAACTTTGAGGGTGAATATGAACATTATGATCTTAGGGATCAAATGCTTAAATGGATTAATCAGATGTTAATGGAAGATAATGTGGAAGAGAAGGCCTATATGTCTCGACAGTCTGCTGCCCTTAAAGATGCAGAAAGATCATTTTACGAGCTTATTGGGGAGGAGTACCCGCCATCCCCGAATCTTCACAGAGTTCCTGATTTGGATCGAAATGAAAACTATGGTGGTAGTGGTTATTATGGATCGGGAGACAATGGTAACGATGATTCATTATGTCCTAACTGGGATCCCAATCCCCGGGAGCGTCAAACTACTAGTGACCATGTTCCCATCAATGTTGCTCCGAGTACTACTTCTCAGTCATCCTCATTGCTTGGCCAGGGCACCGTAAACAATGGGACTGTGGACTTTGTTGCGAGTACAATTACAATTCCTGATATATCCAATTTTACTGAATCTGTTGAGAAAGGGATTAGGGAAGGGAGTTCGTTCCTTCCTACTCGCAATAGCTTGCTCGTTGATGGAGTTGGGGCGGAGAAGAATACCGGGAGTCAGGACTTGCTTGAAGGGCGAAGAGGAAAGAAAAATATGTGTCGTGACGATATGCATTTGCCCGAAGGGAGATTTTACAAGCAATCTGCAATTTATGCTGAACCAAGCATTAAACAAGAAGAGTTTGACGAGGTGTTATTATGCAGTGAAGAGGACGAATCCAATCTTTGTCATTCCTTGCAGAGTGTTTCGTGTGAAAGTGCAACGGGTAATGATGATTCGAAGGGTTCTAATAGGAAGAAATCGAGTGGAAAGAAACCGGGGTCTGAACGAACTGTGGTAGACTTGCGATCTCTCTTGATGCTTTGTGCACAAGCTGTAGCTGTTGAGGACATAAGGACTGCCAACGATTATTTGAAGCGTATCAGACAACATTCGTCTCAAACTGGAGACGATATGCAGAGGCTGGCGCATTATTTTGCTGACGGTCTCGAGGCAAGGATAGCGGGATCTGGGACGAGAATACATAAAGCCCTTGTGAAATATCCTAGGTATGCTGCTAGGGCCTTGAAAGCTTTCAAGCTCTATCTTTCTTGTTGTCCATTTAGGAAGATCTCGAATATGTTCTCGAACAAGACCATCACAACTCTAGCCCAGAACGCTTCATCACTGCACATTATCGATTTTGGGATTGGTACTCTTTTCGGCTTTCAATGGCCTTGCTTAATACAACATCTCTCGTCTAGGCCCGGTGGACCTCCCAAGCTTCGTATAACCGGGATTGATTTTCCACTGTCAGGTTTCCGGCCAGCAGAGAGGGCTGAGGGGACAGGGCGACTTTTAGCTTATTATGCTGAGAAGTTCAATGTCCCATTCGAGTTTAATGCTATAGCAAAGCAGTGGGAAACAATTACAGTCGAGGATCTGAAGATCATCGAGGGCGAGGTGCTTGCTGTGAACTGTCTATATCGACTTAGGAACCTTCTCGATGAAACAGTGGTTGTAAACAGCCTCAATCCGAGGGATACTGTTCTGAAACTCATTCACGATGTCCGTCCAGATGTTTTCATACACGGGATTCTCAACAGTGCTTGTAATTCCCCCTTATTTACATCGAGATTTCGAGCAGCTCTGTCTCATTACTCAGCTGTGTTCGATATGCTTGAGGCCACTATTCCCCGCGAAGTTCATGAGAGGATGCTGATCGAAAGCTACATATTTGGTCAGCAAGCCATGAACGCGATTGCATGTGAAGACACCGAGAGGGTCGAGAGGCCAGACACGTACAAGATGTGGCAGGCCAGGAACACGAGGGCTGGATTTCTTCAGCTTCCTTTGAACCGGGAGATCGTGAAGATGTCGATGCATATGTTGAAGCGATACCACAAGGAGTTCGTGATCGATGAAGATGGACACTGGCTGCTTCTGGGATGGAAGGGGCGCGCTTTCGTCGTGGAAACCAGCTTAGTTAATTGA

>IbGRAS10

ATGTTTCAGGATAACGATGGATCCTCGTCCATAACTTCCTTGTCGCCGAGGATGCCCGCCGGAATTGGGTCGCTGTGTCACTGGTTCAAGGAATTGAAGCCGGAGGAAAGGGGCCTTTACCTCATCCACCTCCTTCTCACCTGCGCTAATCACGTCGCCGCCGGCGCCCTCGACAGCGCCAACGTCGCTCTCGACCAAATCTCCCACCTCGCCTCCCCCGACGGCGACACCATGCAGCGCATCGCTTCATATTTCGCCGAGGCCCTCGCGGATAGGATTCTCCGGTCGTGGCCGGGTCTTTATAAGGCCCTGCATTCCACTAGACTTCCGGTTCTCTCCGATCAAGTCACGGCCAAGAAGATGCTTTACGACCTTTTCCCTTTCTTGAAGGTGGCTTTCATGGTGACTAACCAAGCCATAATCGAAGCCATGGAAGGGGAAAAGCTAGTCCACATCATAGACCTAAACGCCGCCGAGCCCACCCAGTGGCGGCCACTTCTCCAGGACTTGAGCGCCCGCCCCGAAGGCCCGCCGCATCTCCGCATAACCGGCGTTCACCCCCACCGAGAGCCCCTAGACCAAATGGCCCGAACCCTCACCGACGAAGCAGAAAAACTCGACATCCCTTTCCAGTTCACCCCAATCGTAACCCACCTGGAAACCCTAGATTTCCAACAACTCCGCGTAAAAACCGGCGAGGCTCTCGCCATCACCTCCTCCTTAAAACTCCACACCCTCCTCGCCCACAACCACGAACCCGGAAAAAACCCTAACCCCCAAAGATTGGGGGACAACAATTTTCTCGAAAACCACAAAATCAAAGGATTCCTCGACGCCATACGAGGTTTATCCCCCCAAAATCGTGGTGATCTCCGAGCAAGAGAGCAACCACAACGGGGAAACTCTCATGCAGAGACTATCAGAGTCGCTCCACTACTATGCAGCATTATTCGACTGCCTAGAATCCACGCTGCCCCGAACATCCTCGGACAGACTAAAGGCGGAGAAGCTGCTGTTGGGCGAGGAGATCAAGAACATCATAGCCTGCGAGGGAATCGAGAGGAAAGAACGGCACGAGAAGCTGGACAAATGGTTCTGGAGATTCGGGTCGTCGGGTTTCTGGAACGTGCCGCTGAGCTACTACGCCATGTTGCAAGCTCGGAGGTATTTGCAGAGCTGCCGGTGCGATGGGTATAA

>IbGRAS11

ATGGATCCTCGATTCACTGGGTTGCCCGACTCTGTAAGCAGTTTCAAGGTGGAGGATGAGTTCCTTTTTTCTAGCTTTCAGCAATCTCAGGATTTTCCTAACAATTATTTGGATGGCTCTCTACCTGATTTCATGGGTGTCCCTTTGATTACACCCAGTCCCGACTCTGGCTATTTTCCTCCACCTGCAGAGTTGGACTCCCCAGATGATCAGGATGCCGATCCAGTGCTCAAGTACCTTAACCAGATACTATTGGAGGAGAACATCGATGAGAAGCCCAGTATGTTCCATGATCCAATTGCTCTTAAAGCTGCTGAGAAATCATTCTATGAAGCCCTTGCTAAGGGCCCTCCTTCACCTTATCAATCCCATGGTAACTATAATACAGAAAGCCCCGATAGCTTATTTGGGAATTCCTGTGAACCCAGTACAAGTAGCAGTACCTTTGGTAGCAGTAACACTGACCCTCTGTGGATAGTTGAACCTGAAGAATCGAAATCATCTTTTGCGAGCTATCCTCCTGACCACTTTTTCCATTCTTCTTTTGAAACCACCTCTGAGCGGTCAAATGAGTCCATACTGCAATTCAAGAGAGGAATGGAGGAAGCGAGTAAATTCCTCCCTGCTAGTAATCAGTTGGTTATTGATTTAGATCAATACTCGTTGCCCCCAAAGACAGAAGAGTTGCCTAGGGATACTGTCATCAAGTCTGAGAATAGCGAGAGGGACAGTTCACATAATTCCTCTAGAGGAAAGAAGCATCACCATCTAGAGGATGATGGATTTGAAGAAGAGAGGAGTAGCAAGCAATCTGCAGTTTATGTGGAAGAGGAGTTATCTGAGCTTTTTGATAAGGTTTTGCTGTGTAATCCTAATGAGTGTGTCTTGACTACTCGCCCTCCTGAAGTTGACAAGGGTGTGCCACATAACGTGCCAAACAGTGGGAAAAGCCGTTCAAAGAAACAGGGAGCTACAAATGAAGCCGTGGATCTAAGGACTCTTTTAATCAGCTGTGCGCAATCTGTTGCTGCTGATGATCGCAGGACTGCAAATGAGCAATTAAAACAGATCCGGCAGCACTCTTCACCTAACGGTGATGCCAACCAGAGGCTTGCAAATGTGTTTGTGAATGGTCTCGAGGCACGGTTGGCTGGCACTGGGACCCAGCTCTATGCAGCCCTAGCCCCGAAAAGGATCACAGCTTTTGAGAAGTTGAAAGCATACCAGGTTTACATGTCAGCATGCCCATTTAAGAAGATAGCAATGACCTTTGCAAATAAAATGATTCACAAAATATCCTTGGGAGCTCCAACACTACATGTCATAGATTTTGGTATTCTTTATGGTTTTCAGTGGCCCATCCTTATCCAGCATCTTTCAAGCAGGCCTGGTGGGCCTCCTAAACTTCGCGTTACAGGAATCGATCTTCCCCAACCCGGTTTTCGGCCAGCAGAAATGATAGAGGAGACTGGGCGCCGCTTGGGGAAGTATTGTGAGCGCTTTGGTGTACCATTCGAGTACAACGCTATAGCAACTCAGAATTGGGAGACAATTAAAATTGAAGATTTGAAGCTCGTAAGCGGTGAGGTGGTTGCTGTAAACTGTTTGTATCGGTTTAAAAACCTGCTTGATGAGACAGTGGCGGTGGACAGCCCCCGCGATGCAGTTCTAAGCCTAATCCGGAAAATCAACCCGAGTATTTTTGTGCATGCTGTCATAAATGGATCTTACAGCGCTCCTTTCTTTGTTACTCGGTTCCGAGAGGCTCTCTTCCACTTCTCTGCACTCTTTGATATGTTTGACGCCACAATACCCCGCGACGATCAACATAGGTTTCATTTCGAGCAAGAATTCTATGGGCGTGAGACATTGAATGTGATTGCATGTGAGGGCGTAGAGCGGGTCGAGAGGGCTGAGACATACAAGCAATGGCAAGTGCGGACTATGAGGGCCGGGTTCAAGCTTCTCCCATTGAACCCCGAGCTGATGACCAAACTGAGAGAGAAGAAGGCTGCAGGAGGATACCACCGAGATTTCTTGTTTGATGAAGATGGTAAATGGATGCTGCAGGGATGGAAGGGCCCGGATTATTTGCGCTAG

>IbGRAS12

ATGGATCGTTTTGATTCAAATTACAATCATCAATTTACTTCAACCTCTTCTTCTTCTTCTTCTTCTGGTGATCTTTCAATCGAATCATCATCTTGTGGTGGCGATGATTCAACTCCTCTGGAGCGAGATTACTTTGATGGAGTTCTCAAGTACATCAATCAGATGTTAATGGAGGAGGAGGATTTGGAGAACCGGCCTTGTATGCTTCAAGATTCCCTGGCGTTACAGGCCGCCGAGAAGTCCTTCTATGAAGCCCTAACGGACTGCAATTTCTCCGATGAAAGGAATAGGAAGAGAGATAACTATGGCGATGGGGATGTGGAAGGGAGAGCTAACAAACTGGTTGCGGGATTCACGGCGGAAGAATCGGAACAAACTGAGGCGTATGATAAAACTCTGCTCTGTTCTGCAAATAATCCTGGCTTCTACAGTGATCCGCCATGGTGTCATCTTGATTACTCCATGGAACAAACCCCGAGATTCACTCCGGCCGTTACTGTCCAGTCGAAACGAGGGAGGCCACGCGCGGGCGAGAAGCGCGTGGGGTCCGGTAAACCGGTTGATCTGAGGAGTCTTTTGCTTGAATCTGCAGAGGCGGCGGCGAACTATAACGGCCGGACGGCGAGTGACCGGCTGAAGCTCATCCGGCAGCACAGTTCTCCTTACGGCGACGCCGCCGAGAGAACCGCTCACTACTTCGCCAACTCCCTGGAAGCGCGCCTGGCCGGAACGGGAACGGAGCTATACACCGCCTTCTCCCGGCGGCGAATGTCGGCGGCGGAAATGCTCAAAGCTTATCAGGAAAGCAACGGCGATTCACATAATAGATTTCGGGATTCTATACGGATTCCAATGGCCGTGTTTCATCCAGGGAATCTCCCTCCGCCCATCCGGCCCGCCAAAGCTTCGAATCACCGGCATAGATTTCCCCAGCCCGGTTTCCGGCCGGCGGAGAGAGTGGAGGATACCGGCCGCCGCCTGGCGAATTACTGCAAGCGATTCAATGTCCCATTCGAGTATACCGCCATAGCCGCAAAAGATGGGACACCATTAAGTTTGGATGAACTAAAGACGGATAGAGAGGAATTATTAATCGTGAACTGCTTATACAGGCTAAAGAACACGCCTGATGATGAGAATAGTCCCCGGGACGCAGAAGCTCTATTCCACTTTTCCTCCCTATTCGACATGTTGGACGCCGCTATCCCGAGGGAAGATCAGGAAAGATTGGTGTACGAGCGGGAGATATGGGGGAGGGACATCATGAACATTGTGGCATGCGAGGGAAGTGAGAGGGTAGAAAGGCCAGAAACGTACAAGCAGTGGCAGCTGAGGAACCAAAGGGCAGGGTTTAAGCAGGTTGCCCTAAACCAGGATGTGGTTAAGGAAGTTAGGGCTAAGGTAAGGTTGCGTTACCACAAGGATTTCCTGGTGGATGAACACAGCAATTGGATGCTGCAAGGTTGGAAAGGAAGAGTTCTGTGTGCCCTTTCTTGTTGGACACCTGTTGAGAAAGCTATGACGGGCTGA

>IbGRAS13

ATGGGTACCCTTTTCCGAGCTCATCATAATTCCAGGGATGAATCTGAGTTTAACCACCACTGCTCTGTTCCAACTTCGGGTGGTCTAGACAGGAACTTGGTTCTAAACGAAGGCTGCAGTGATGTTCTTGGGTTTTCAGGCATGAAGTCTGTCCATGATGGCCCAGCTCCATCTGAGGGGGTGGAAGGAGACTACTTTGATGGAGTGTTTAAGTACATACAGAATATGCTTATGGAGGAAGATGATCTGGAACATATACCTTATATGTTCCAAGATTGTATGGCTCTCCAGGCCGCCGAGAAATCATTCTATGATGCCCTAACTGAGAATCCTCCCCCCCCCCCCCTTAATCATAGAAACTTGTCTGATTCTGGAAATAGAAATGCTCAGTTCTCAGTATCTGATAATCAAGTAGCTTCTGAGCCTTTCCCGGCTCAATTTCCCCTTCCTGGAAACCAAAATTTTCAGGCTGGTTTGCAGGAATTGCAGCCAGGCTATAATCCTTTTCCTGGGCAAGTAGAAAATCCTATGATTCCCATGAATAATTCATCTGGATCCATGTTTAGTACTCAATTCTCCGGCGAGTCGGGGACGTACTTGATCGGAAATGGCTGTTCTGGAAACGGAGAACGGAGCAATGGAAACGGTCCAGAGAGGAGAAGAAATCGCAACCGGGAAAACGGCAGAGAAGGGCAGAGGAGCAAGCAGATAGCGACGGGGAATTCCGACCAAGAGCCGGAGAAAACGGAGAAGTACGATAAGGCGCTGTTGTGTCCTTCCATGAATCCGTTATTCTACGACGATTCAATCCCCTATCTTTCCGACGAATCTTCGGAAACGGAAGCGCGTGACAAGAAATACTTGCAGGGCCCGAAGCGCGGGCGGCCACGTGGCAGCAAGAAAGGCGCCAAGCCCAAGCAGATCGTCGACCTAACCGACCTCCTCGCTCGGTGCGCCCAGGCCGAGGCCGCGCACGACAAGAAGAACTTCGACCTCCGCCTCGCAGCCATCCGGCAACACTCCTCCCAGTACGGCGACGCCACCGAGCGCCTCGCCCATTGCTTCGCCAACGCCGTGGAGGCACGCGCCGCCGGCACGGGAACATCCTTGTACGCCAGCATAACCAGGCGTAGAATGTCGGCCGCGGAATATCTCAAAGCTTATCAGACCTACATAACCGCCTGCCCCTTCAAACGCATGTCCAACATATACGCTAACAAATCAATCGCGAAACTAACCAGAGAAGCCGAGAAGATCCACATCATCGACTTCGGGATCCTCTACGGGTTCCAATGGCCCTGCATCATCCACGGCATCTCCCTCCGACCCGGCGGCCCGCCGAGGCTCCGCATCACGGGAATCGACTTTCCCCAGCCGGGGTTCCGCCCGGCCGAGCGAATAGAGGAAACAGGTCGGCGCCTGGAGAATTTCGCCAGGAGATTCAACGTCCCCTTCCAGTACACCGCCATAGCCAAGAAATGGGAAACAATCACCCTGGAAGACCTGAAAATCGAGAAGGACGAAATCCTTGTAGCAAACTTGCCTATACCGGATGAAAAACGTCCCGGACGAGACCGTAATGGAAAACAACAACCCCAGAGCCGCCGTTCTCAAGCTAATAAAAAAAATCAACCCGGAATTCTTCGTCCACGGGATCGTCAACGGGATGTACAACGCCCCGTTCTTCACAACCCGATTCCGAGAAGCCTACTTCCACTTCTCGGCCCTGTTCGACATGTTCGAAGCCACAATGCCCCGAGAAGACGAAGGGAGGATGCTGGCCGAGCAAGAACTGCTGGGCCGAGACGTCCTGAACGTGATCGCGTGCGAAGGGAGCGAGCGAGTGGAGCGGCCCGAGACGTACAAGCAGTGGCAGATGAGGACGCAGAGAGCCGGATTCCGAGCGCTCCCATTGCACCGGGAGATCATCAAGGAAGTGAAGGCGAAGACTAG

>IbGRAS14

ATGTACTATACTACAGTTGCCGGAGAAGCTGCGGAGGCGCAACTAACCTGCTGCCGCCGGAAAGAAAACAGAACAGAGGCGCGACTGGACCCTACTGCCGCCGGAAAGAAAATAGACGGAAGAAGCGTCGCCGTCCTCTGCAGCCAGAAGTTGTCGCGCCGCTGTTGCCGCCGGAAGTCCACCGTCACTGTCGCGTCGCAGATCTTGAAGAAGAAGCTTCTAAGTTGCTGTGACCATTTTCACCAGAGTCGTTCAGGTGCTAAGCTACAACGAAGAAGAAGAAGAAAAGCTAGAAACCTTAACACTCTCTTCTCTTCATCATCACTCTCATTGTTTCAGACCCTCTGTTCTTCCACCTATTTCTTGTTTTTTGGGGTTCCTTGTGTCTCAATCTCTGTGATGGAGGCTCTGGTCCAAGAATTGATTACCACGCCGAATGATTTCGTCTACAACCACCACTCAAAACCAGTGAATCCGACGCTGAGTGGGCAGAGATTCAACCTTGTTGAGAGATCTGCAGGCGAGGGGGAGGAAGGGGACTACTCTGATGCGATTCTCAAGTACATTAGCCAGATGTTAATGGAGGAGGAGGACCTGGAGAATCAGCCCTGCATGTTTCGCGACTGCATGGCTCTCCACGCCACCGAGAAATACTTCTCCGACGTGCTTAATGGCTCCGGGGACAACTCCCCGTTATCCATCCCCCAATATGATGCCAGTTTCGCGGGGAGCAGTCCCAGCAACTGTTCATCTGATCTCAACAATGGGGATTCAGATTTAAGCCCTCAGTTTCAGAGCTCCTTCAATGGCTCTTTGTTCAGTCAATATGCAAGCAGAGGAGCAGCAATAGGCCTTCCAAATGACAGTAATGTTCTCAATAATCTTGACATGGAAGTGAAGGAGAGCGAGGGGGAGAATTCGCCCTCGAAGGGGAAAAGAAACCATTACTACTACTCCTCCGACGATGGTTCTGCAGAAACACAGAGAAGCAACAAACACCTCGCAAGTTATGCCCCTGCTGATGAACCGGAGCCATTAGACATCAGAAGGGGCAATCCAAGGGAGGGAGAAATCCGGCGTGGCAAGAAGAAAGAAACGCAGAAGGAGTTCGTGGACCTCAGAGGCTTACTGACTCAATGTGCACAGGCCATGGCGAGCTATGATACCAGGACGGTGACTCAGCTTCTGAAGAAGATAAGAGACCACTCTTCTCCTCAAGGAAATGGCAATGAAAGAATGGCGTTTTATCTCGCCAATGCGCTCGAAGCGCGCTTGAATGGAACAGGGACGGCATTGTATATATCTAACTCTCCCAGCAATATCTCAGCCGCAGATATCTTGAAAGCTTATCAGATGTATATCACGGCCAGCCCTTTCAAGAAAGTGTCCAACATGTTTGCCAACAAATATATCAGGAAGGTTGCAGCCGGAGCCCCGAGGCTACACATAATTGATTTTGGAATTCTGTATGGATTTCAATGGCCCTGCCTTATTCAAGGTCTCTCGACAAGGCCGGGCGGGCCGCCAAGGCTTCGGATTACCGGAATAGATTTTCCCCAGCCGGGGTTCCGTCCGGCGGAGAGAGTGAAGGCCACCGGCCGTCGCCTAGACAATTATTGTAAGAGATTCCATGTCCCGTTTGAGTTTAAAGCTATAGCACAGAAATGGGACACGATAAAATTGGAAGACCTAGAAATTGACAGGGACGACGTGTTGGTGGTGAATTGCCTGGACCGGCTGGGCAACGTGCCGGACGAGACGGTAGTGCCGGATAGTCCAAGAGACATTGTATTGGATCTGATCAAGAAGATCAATCCCGATGTGTTCATCCATGGAGTTGTGAATGGCACGTACAACACACCGTTCTTCGTCACGCGATTCCGGGAGGCGCTCTTCCACTTCTCCTCCTTGTTCGACATCTTCGAGGCAACAGTGCCAGGACAAGGACCGACAGTTGTTCGAGGAAATGGTGTTCGGAAGAGACGCGATGAACGTCATAGCCTGTGA

>IbGRAS15

ATGATGAAAGGGGGAGGATTTGAAGTGATTCAACATGAACAGTTATGGGATCATCATCATCATCACCGCTATGGTGATAGTGTGAGTGAAATGGGAATTTCTATTGTTGGTGGTGGTGGTGCGAGCTCTAATGGCGCGGCGGGGGAGCTCTCCCAGTGGGTGGAGCATGTGACGCGGCAGCTGATTGAGGACTTGCCGGAAAGTGAAGAAGTTGCAGCGCCGCCGCCGCCGCGCGGCGAGGCGGTTCAGCCTTCTACTACCGCAGGCTGCCAAGAGAGCAAACGGAGCTCCGAGGACGACGATGACGGCGGTATGAGGCTGATAAGTCTCCTCTTGGAATGTGCGGTGGCGATTTCCGTCGATAATCTCGGCGAAGCTCACCGGATGCTCCTTGAGCTGACGCAAATGGCGTCCCCGTACGGCGCGTCCTGCGCCGAGCGAGTAGTGGCGTATTTCGCCAACGCCATGGCGAGTAGAGTCATCAATTCATGGCTGGGAATCTGCTCGCCATTGATAAACCTCAAGACTCTCCATTCCTCCTTCCAAATCTTCAACAACGTCTCCCCTTTCATAAAATTCGCTCATTTCACCTCCAACCAGGCCATCCTCGAGGCGGTTCACGGCCACGCGCGCGTCCACATCGTCGATCTCGACATCATGCAGGGCCTCCAATGGCCCGCGCTCTTCCACATCCTCGCCACGCGCGTGGAGGGCCCACCGCCGCATCTCAAGATGACCGGGCTCGGAACCTCCATGGACCTCCTAGTCGAGACCGGAAAACACCTCTCGAGCTTCGCTAAGCGCCTCGGGTTATCCTTCGAGTTCCACCCGGTCGGGAAAAAGTTCGGAGAGATCGACGATGTCTCCGCGCTGAAAATCCGGCGAGGAGACGCCGTCGCCGTGCACTGGCTCCACCATTCCCTCTACGACGCCACCGGGCCGGACTGGAAAACCATGCGGCTCCTCCGGCAGGTCTCCCCGACCGTGATAACCCTAGTGGAGCAAGAAATCGTGCACGGCGGTTCGTTCCTGGACCGGTTCGTCGGCTCGCTCCATTACTACTCCACCGTGTTCGACTCGCTCGGCGCGCTGCTGCCGAGCGACGACGCGAGCCGGCACACGGTGGAGCACTGCCTTCTCCGGCGGGAGATTAACAATATCCTGGCGATCGGCGGGCCGGCTCGGAGCGGGGAAGACAAGTATAGGCAATGGAGGAGTGAGCTTTTGGGGAATGGGTTTTTGCAAGTGGGAATGAGCAGAAACTCCATAGCTCAAGCTCAGCTCATCCTCAATATGTTCCCTCCCTCTCATGGCTATAGCCTTGTACAAGGAGATGGAACCCTAAGGCTGGGGTGGAAGGAGACCAGCTTGTACACTGCTTCTGCATGGACTTCACCTGCACCAAATTCTAGATAA

>IbGRAS16

ATGGGTGATTTGGTAATATATGTGGGGGATGATGATTGCGAAATAGTTTGTGGTGCAATGTGTTTCTGCAGAATTTGCCATGAAGCAGAGTTTGAAAGCTCCAAGATCTTGGAAGCTCCTTGTGGATGTTCTGGGACTCTCAAGAAGTTTGAAGATGGCTACACGGCTCCACCTCCCAAGATGCTTCACACACCACCTGTCACCATTTGGGAGAGCTCGGAAAATCCAAGAATAGAAGAAAATTCGAGAGTGGCGGGTGAAGAAGATGAACGATGTGCATTGAATGATCTGACATTTTTGGTCCTAATGGGGCATCTATTTGAGTTGCTCGCCGGTGAAGCAAGGCGTTACCCATTTTCACTTATCACGGTTGTGGTTATAAAGGCCGTTGGCATACTGCTGCCAGCATACCTGCTAATTCGTATAATCACACTTATCCAGAATGGTGTAATTAGGCCGCATTTGCTAGCTGGGCACGAAGAAATGGAGAAGCAGAGGAGTTTCTCCATCAAGCCCACTCGATTCTTGGTTTTTACATTTACAATCTGTTTATCCGCCGTCTTTCTAATCTTCTTCTCCGTCTGGCTCGTCAAAATCTCTCCTTCCCCTCAAGAAAACCATCTTCACCTCAACAGCAACAAGGATCCAAGTGTTCAAGTTCAAGAAAATGGTTCCCCAGTTGCGGAGGAATATGAGAGTGAATTCGCAGTTGAAGAAGATGCAGGTGAGAGTAAATTCGCGGCTGGTGAGGTAAATGCGACCGATGTATTGAATGGTAACTTTACCATTACCACTGTCGCATATTTTTCTTCAATCGTTGTAAATGATACACTTCTGCGTGGTACCCACTTGACAAAATCTGATTTAGCTGTAAATCAATCTGAAAATGTCCAAGACTGTGGAAGTAATGGTGTTGACTGTAATAGTAAGCAACAACATGTCTCAGATATTACTTTTTCCAAGAAAATAAGAGGCCCAAGAAGTAGTGTGATTGAGAAGAAGAGTGGAAGATCTGTTTGTGATGTCACAATAGGGAAATGGATTGTTGATGAGAGCTATCCTTTGTACACCAATATTTCTTGCCCTTTTATAGATGAAGGGTTTAGTTGCCAAGCTAATGGAAGACAGGATAAAGACTACATGAAATGGAGGTGGAAACCTCAAGATTGTGACATTCCAAGGTTTAATGCTACTCACATGTTGGAATTGATAAGAGGGAAGAGGTTAGTATTTGTTGGGGATTCCATTAACAGGAACCAGTGGGAGTCCATGTTGTGCTTGTTGATGGGAGCCATCAAAGACCCAAAAAAAGTGTATGAGACCCATGGTCGAAGAATAACCAAAGGGAAGGGAAATTATAGTTTCAAGTTTGTGGACTACAAATGCACAGTTGAATTCTACGTTACACATTTTTTGGTTCGTGAAGGTAAGGCAAGGATAGGCAGGAGACGAGGTCAGACTTTGCGTATTGATGCCATTGACAGAGGGTCATCCAGATGGAGTGGTGCTGATATTCTGGTCTTCAACACTGCACATTGGTGGTCACATCACAAAACAAGAGCAGGGATCAATTATTACCAGGAAGGGGATCAAGTTCACCCCCGCCTTGATGTTTCAACAGCTTTCCAAAGAGCTCTAATGACTTGGGCATCCTGGGTTGACACACATATCCATCCTAGGAAGACACAAGTTTTCTTCAGAACCTCTTCACCCGCTCATTTCAGCGGTGGGCAATGGAACACTGGTGGCCATTGCAGAGAAGCTTCTCGCCCTCTCAAGGAGAGTTTTAGCACAGCTTATCCCAAGAAGAATATGATCGTGGAACAGGTCATAAGGCAGATGAAAACTCCAGTAACCATTCTAAACATAACTGGTTTGTCAGATTATAGGATAGACGGCCATCCATCTACATATGGAAGAAAATCTGGTAGCAAGTCTGGTGTCCAAGATTGCAGCCATTGGTGTCTTCCTGGGGTTCCTGATACTTGGAACGAGATATTGTATTACCACTTGCAACTTACTCAACAGCACAGAATCAAATTGGCTGATCTAGAATTTGGTGGACAGGAATGGGGTAGTGATGAGCTTTATTGGTTAAGGATTGAATCACCTGACCGAAATTCGAAAATCGCAATCGAAATCTCAAATCTGGGAAGTGGAAAGCCGCTCCGCTGCTCCTGCTCGCCGGTTGGCCGCCGTCGCGGCGTCGCCTGCCCCCGCCCGCCTGCTCCGCTGCTCGCCGAGTCGCCGATTGGCCGTCGGCAGAATGGCAGATCTATCTCGCGTCGTCGCTGGCTCGCTGCTGCACGCCTCACGCCTGCACCTGACCGGCTGTCGGCTGACCTTCCTCCGCAACCGCAACAACGCCGGCGGCCGGTCTCTCACTCTTCGTCTGACCTTCCTCCGCAACAGCTGTTACGGACTTTACCTGCGCTCCAAACCTCAAACGGACAGTCTCCACGCCTCAAACTCCACGTTGACGACTCCACCTGCGCTCCAAGCCTCCAAACCAAGTCCAAACCCTCCACTTTCACTTTCAGTCACATTCCAATGAGCAGTAATAGTTCTGCCAAAGCTTATGACCAAGGTAAGGCCAGTAGCAGCGGCAAGATTATTGATGATAATAAGCAGTTAGCGTCAGGATTTCAAGGTCATGGAGACGATGTTGATTTATTTTGTGCCAAGTACGGATTCTATCAAGAAAATGATCAGCCACTGGGTGTAGAAAGGCAGTTTATTACCAGTAATTCATCAAACAATATTCAAGAAAGCACCAAACTTCAAGCGGAGAAGGCCTGGCCACCTCTTTCTCCGGCAATTTTAAGGATCCTAGGTAATCGTTCAAATCCTTTCGCCGCTACTACCAGTAGTAGTGGAGGTGGCCGCGATCATCATCAGAAACTGTCAGCTATGCAAATCGTGAGGCTTGCAGGAGAAAGATTCGTACAATTCTCTGATCACAAGTTCATTAATATCAACATCTTCAAGCATCCTTATAGTTCGACCCTCTCTGAATTGTGTTCTCAAGATAAACAAGATGTGGAGCTTGTCCAGCTCCTCCTGGCCGCAGCCGAGAAGGTTTCCGAGCAGCAATTCGACAGGGCAATCAGATTGGTGAGCCAGTGCCGGAACAGCGCGTCCTGCACTGGCTCGCCGGTTCAAAGAGCAGCATTCTACTTTGCGGATGCTCTTCAGGCCAGGATTGAGCGACAAACGGGTAGCTCCGGTCATGAAGATGATGATAGGAACGGTGTGAAGGACGGCGAGTGCCTAGCGTCAAGATTCAACAAGGCGTACCTGATCTTGCATCAAACGCTTCCGTTCTCCCAAGTCGTGCAATTAAGCGCTACGCAGACGATATTAGACCATGTGGTCACGAAACCTAAGGTCCATCTCGTGGATTTCTATTTGAGGACCGGAGTTCAGTGGTCCACACTGATGCAAGCTCTATCGGAACGGGCCGCGGCCGAAGATTGCGGCAAACAACATTATTTCCGGTTCACCGCCATCGAAACCACCGAGAAAGAAAAGGTGGAGGAAACGGGGAAGCGGCTACAAAGCTTCGCCGCCCAGTTCAACTTGCCCTTCTCGTTCAACGTCCTCTATATTCCAGACCTAAAGCACTTAAAAGCAGAGCAAGTGGAGATAAAGGCAGACGAAGCCGTAATTATCCACACCTCCTTCACTCTCCGCGCAATGATTTCCAAACCGCTGGAGCTAGAAAGCGTGATGAGAGCCATCACGAGGCTGAAGCCCTGCGTGATGGTGGTCCAAGAAGTGGAAGCCAATCTCAACTCTCCCTCATTCGTGCACAGATTCATCGACGCCCTGTTCTACTACAGCGCGTACTTCGACGCGCTGGAAGACACAATGCGGCGCGACGATCAGCACCGGGCGAGCATCGAAGCCGGGTCGGTCCGCGACGGGATCCGGAACATCGTGGCGGCGGAAGGGCGGGAAAGGGTGACGAGAAGCGTGAGCTTGGAGGTTTGGAGAGAGTTCTTTGGGAGGTTTGGGCTTGAAGAAACGGAGCTGAGCCAAACTTGCTGGGCCCACGCTAACATGGTGGTTCAACGGTTCGCGTGTAAGTCTTGTTGCGCAGTAAGCGGCAATGGTAGGTCGCTGGTGGTGGGATGGAAAGGGACGCCTATATATTCCTTTTCTGCTTGGAAGTTCCCATCCCTGCGTCGTGACAATTGA

>IbGRAS17

ATGGGGACTTCTGGTTTTGACCCTCAGAATGCTGCTCAAAACGAGGCTAACGGTGAAGAAGAAGAAGACGACCACCAAATACTTGTCCTTGTCAATTCTCCAACCCTCCACGACTTGTATCTTGATGTTGCTGTACAACCACCATTTGATGAATCCAGCAACAAGGAAACCCATCCTGCAATATTGCCTTCTTCCCTCGCCCTCCTCAAACGCTTCGGAAGAAGATTCAGTAAACTAAAGGGACAAAAAAAGACCAATCCCAGACACCGTAAAGATGTAGAGCTGGTAGGATACCTTCTTTCTGCTGCCGAGAAAGTGGGGCAATCCGACTATGATTCTGCAGAAATTCTACTCACCAGATGCGATGAACTAAGTTCCCATCAGGGGAATTCCGTGGAAAGACTAGTCCACTATTTCTCCCAATCTCTCTGGGCAAAAATCTTTTGCCAAACAGATTCCAGCGCTCTGTTCTTTCAAGAAGACCTTGAAGAAGCACTCATGAGCCTAAGGCCCTGCATTGCTTACCATCAAAAAGTACCCATCACTCAGGTCTTCCAATTCACCAGCATTCAAACCGTGATAGAGCACGTAGAAGACGCAAGAAAGGTGCACATCATTGATCTCGAGATTAGGAGTGGGGTTCAGTGGACGATTCTGATGCAAAGCTTCACCGAATCCCCCCGCCCCGAGCATCTAAAGATTACTGCTCTACAGGCAACTAAACACCAATCCAAGATCGAGGAAGAAACGGGCATGAGATTAAGAAGCTTTGCCCAGTCCTTAAACTTGTGTTTTTCGTTTAACATAGTGGCTTTAGAGGACCTACTAAATGATAATAAGGAAATTAGCCTCTCTGGGTTTCAGCCAGATGATGAAGAAGAAGTAGTGGTAGTATATGCAAGTTGCTTTTTCGCAACAATGATTTCAAAGCAGGAAAAAATGGAGTCTTTGATGAGGGTGATCAAAAGTGTGAACCCCAGGGTGATGTTCCTGACAGAAGTGGAAGCCAACATGAATTCCGTGGGGTTTGTGAACCGTTTCACTGAAGCCCTGTTCTACTACGGGGCGTATTTCGACGCATTGGAGGATTGTATGAAGAGTGATGAAGCCAATAGGACAACCATGGAAGCCAAACACTTTGGTCAGGGAATAAGGAACGTAGTGGCAAGTGAGGGGGAGAGTAGGGTGATTCGACATGTGAGCATAAAGGTGTGGAGAGAGTTCTTTGTGCGGTTTGGGATGGAGGAGATGGAGCTGAGCACGCCTTCTGTGTACCAAGCAACTCTGGTGTTGAAGAGATTTGGTTGTGGGAAGAGTTGCACACTTGACATGGATGGGAAGGCCTTGACTGTTGGGTGGAAAGGAACTCCTCTTCTCTCACTCTCTGCTTGGAAATTTCTTTAG

>IbGRAS18

ATGGTCCAAGAGGAGGGTTCATCTTCGATTGGTTCGTCGCCACTTCAGTACTTCTCCATGATGTCGCTCTCGCCTGGAATAGGGTCACCGTATCCCTGGCTTGGAGAGATGAGGTCAGAGCGACGGGGATTGTTCCTAGTCCATCTTCTGGTCACCTGTGTGAACCATGTTGCTGCTGGGAACATTGAGAATGCTAACATTGTCCTCGAGCACATCTCGCATCTCGCTGCTTCTGATGGCGATTCGATGCAGAGAGTGGCTGCATACTTCAACGAGGCGCTTGCTGACCGCATCCTTAAAGGCTGGCCGGGGCTGTACAAGGCCTTAAAGTCCACCCGAATAACGTCTGCAGCTGATGAAAATGTTGTGCAGAAGATGTTTTTTGAGCTCTGCCCGTTCCTGAGATTGTCTTATGTGATCACGAATGAGGCTATCATGGAGGCGATGGAAGGGGAGAAAGTTGTCCACATTATTGATCTCAACGCTTTCGAGCCTGCACAGTGGATTAGTCTTCTGCAGGCTATGAGTGTGCGCCCCGAAGGTCCACCACATATCAGGATAACGGGGATTAACGAGCATAAAGAGGTGCTGGAGCAGATGGCACACCAGCTAAATGAAGCTGCAGAGAAGCTGGATATCCCGTTTCAGTTTAATCCTATAGTTAGCAGATTGGAGAGTCTCAGCATCGAGAGCTTGCCCGTGAAGATGGGCGAAGCGATTGCAATAAGCTCGGTGCTGCAGCTGCATCCTTTTTTAGCATTCGACGACGAAATGCTGCAGAGGAACACCCCCCCGGTGGTGTCCAGGCACGCGAATTCTGTCCATCTGCAGAGGATCCTGCAGGTGAATCCTCGTACCTTGGGGGATTTTCTCGAGAAAGAAGTGGCTAATCCATACGGTGCTAGCCCCGATTCCACTTCCTCGTCGCCTTTGCCTTTAGCCACTGCACCAAAGATGATGAGTTTCCTCAATTCCCTGTGGAGCCTCTCCCCCAAAATAATGGTCATGACTGAGCCAGAAGCAAACCACAACGGGTTCAGTCTGATGGACAGAACCATGGAGGCCTTAAACTTCTACGCAGCCCTTTTCGACTGCTTGGAATCCACCATCCCACGAGCATCACCCGAGCGCCAGAAGATCGAGAAGATGGTATACGGGGAGGAGATCAAGAACATCATATCCTGCGAGGGACTAGAACGAAAGGAAAGGCACGAAAAGCTCGAGAAATGGATCCCAAGGCTCGAGCTGTCTGGTTTCAGGAAGGTGAGATTGAGCTATCACATCATGATGCAAGGGAGGAGATTGTTACACAGCCATAACTACGATGGATACAACCTTAAAGATCAAGACGGGTGTTTCCTCATTTGCTGGCAGGATCAACCCCTCTTCTCGGTCTCGGCCTGGAGATTTCAAAGGTACAGCTAA

>IbGRAS19

ATGAAGGGGATGCCCTTTCCCTTTGAATTTGAGAGGAAGGGGGTGATAGAATTGGGTAATAGGAATAATAGAAATTGTGTTTCTGGGAATCATTGTTGGGATATTAAGGATTGTGTTGTGGGGAGTCCAGGTGGGAGTAATAGTGAGCCGACGTCTGTTCTGGATAGGCCTCTTGCGTCATCTTCAACGCTGTCTTCTTCTTGCGGCGGCGGCGGCGGCGGAGGGGAGACGGACGCGGCCAGGGTGGCGGCGGTTTCTGGTAATCCGGCGTCGAAATGGCAGCAAGACAACACCACTGCCACGAGTTCTAATGCAGGGGGAGGTGAATCCGAGCTTCTCCAGCCTGTTCCGCCGTCTCTTGATTTCGGCGGCGGCGCGCCGCCGCCGTGGGAGGCGGCGAGACGGCGGAGAAATGTGGGATGGAGGAGTGGGATAGTTGGCGGCGGCGGCGCCGCCGAGTACGATTTTAATGGTGGGTTTGGGGTGGTGGATCACGGGTTTGGAGCCGTCGACCCGGTTTCTTCATCCGGGGAGAGTTTCCTCCCTTCAATCCCCATCTCCGGGTCAAATTTCCTGGCGAATAGACTCCCCAACCCGCCGGCTTCCCTCCCCGGCTTCAAATTCTCTGCGCCGCCGCCGCTGTTCCCGCCCGTCTCCAACAACCTCGGCGCCGCCGCATTCAACCCGTCGCTTCTAGAACCCTCAGATCTGAAACCCCAGATTTTCAATCCCGGGAATCCCCACTTCTTGATTAACCAGCCCCCCCAAAACCCATCATTCCTCATGCCATTGCCGTTCTCCCGGCCGGAGCTAGCTCCGCCGCAGGCGAAGCGGCATAACCCCGGCGGGAATCTTGAATCTCCGGGACCCCAGATCCCCCGAGGGCTATTCTCCGATCAACAAACCCCATCTCCCCATCACATGCTCCCCCACCAGCTCCAATTACTCCCAAACTATCCCCAAAGGCCCAAACCCCCGGACATCTCCGGGGAAGAAATGGGGCATTTTCATCAAAACCAGCAAACAATGATCGACCAGCTTTTCAAGACGGCCGAGCTGAGCAGCTTTCTATTGCAAGAATCATTGCAATTGCTCCTCCAACATGCCACCAATAACAACATGAACCCCCCTCCTTCGTCGTCCTCGGTGCCATTTAGCCTAATTTTCAAGATTGGTGCTTACAAGTCCTTCTCCGAGATCTCGCCTGTCTCTCCCTTTGCCAATTTCACCTGCAACCAAGCCCTGCTCGAGGCCTTGGAGGGGTTCGATAGGATTCGCATTGTGGATTTCGATATTGGGTATGGAGGCCAATGGGCTTCTCTAATGCAAGAGCTTGCCTTGAGGAGTGGAGGCTCCCCTAGCCTCAAGATCACCGTGTTAGCCTCGCCCGCAATGCACGATCAGCTCGAACTCGGGCTCACACGGGAGAATTTGATCCATTTCGCTAGTGAGATCAATATGGCATTCGAGTTTGAGGTTTTGAGCATTGATTCTTTGAATTCGACATCGTGGTCACTGCCACTTCATGTATCGGATAATGAAGCGATTGCTGTTAATCTTCCCGACAGAGTCTGTGATCGGACTGACCTCTTATTTCCCAACCATGTAATCCACGCTCTTCAGTATTATGCCAACCTTCTCGAGTCTCTTGATGCTGTGAATGTGAACTTCGATGCCCTGCAGAAGATCGAGAGGTTCTTGCTCCATCCCGGGATTGAGAAAATCATAATGGGTCGTTATCGTTCCCCCGAGAAAACACAGCACTGGAGGACTCTGTTTCTATCGTCTGGATTCTCCCCATTGACTTTCAGCAATTTCACGGAGTCTCAAGCCGAGTGCGTGGTGAAAAGAACTCCAGTCCGAGGGTTTCATGTCGAGAAGAGACAGTCTTCTCTTGTTCTTTGCTGGCAGCGGAAGGAGCTTATCTCGGTTTCAGCTTGGAGGGATTCCATTCCAATGGAAGCATTCATTCAGGTCTCTTCTGTTCTAAATGATAACCGTAGATCTTTATTCCTGTCATTGCCCTCTGCCTAG

>IbGRAS20

ATGGCTAGCATCTCCTTCCCTCTTGCTGGAAAACATAAACAGGGTACATGGTTTGGAGTTGAAGGATCTGAAGATTATGATTCTTTCTTCTCCTGCTTGTATGGCCTCAGTCCCCAGGACAGCAGGGCGGATTCAAAGATTGATGATGATCAAAAGCAGAGCATCCAAGTTTCACAGCCGGCTGAGAAATCTGTGCAGAATGAACAACAACCAACTCCATTTTCTTTGGCATGTTTGGAGGTGTTGAAAACTTATGACAAGTTGTTCAATAAACCAACAGATGAGGGGAAGGGGGTAAACCACAATAATAAATATAATGCATCCTCCTGTTCCTGTACCTGTGGTAAATTGTCAGTAAATGAAGTATTGAAGCTTGGCGGAGAAAGGTACATCCAGTACATCACTCCAAGGGCAGATGGGTTTTCCATGTTTATGCACCCTTACGCCTCTGCTCTCTCAGCTCTTACAGCCCACGAAACCCGTGACGTGGAGCTTGTGCACCTTCTTCTTGCAGCAGCAGAGGATGTGAGTCGCCGCCAATATGACGTGGCAGCCAACCTCATCTCTCGTTGCATGTGGACGGCTTCTCATTCCGGCAATCCTGTCCAGAGACTCGTCTTCTACTTTGCTGAAGCTCTGAAAGAGAGGATAGACAGGGAAACAACAGGAAGATTATTCACTGCCGCCAGGTACCATTACCAAGAATATTATTGTATGGGATTGACTACTACCCCTGCAACCTTGGCCTGTCACCAAGGACTTCCTTTTAGCCAAGTTATGCAATTTGCAGGAATTCAAACCATCATCGAAAATGTCAACACTACTAAGATCCACCTGCTTGATTTCAATATCCGGAGTGGGATACAATGGACGATCCTGATGCAAGCTCTGGCAGAAGAACATCACGACCGCCCAATACAGCTCATCAAGATAACTGCAGTTGGAGTCGCAGATCAGCGCAAATTGGAGGAATGCGGCAACAGATTGGAGAGCTTTGCCAGGTCCTTGAATCTCCCATTTGCTTTCCATCTAGTCTTCTTATCGGACTTGAAAGATTTCAGGGAAGATTTAGTCCATTTAGAAGCGGACGAATCCGTGGCTGTATATGCAAACACTGTGTTCAGGACAATGATCGGGAGGCCCGATTGTTTGGACAGTTTGATTTTAGCAATCAGAAAGCTCAAACCAGTGGTAATGGTAGTTGCTGAAGTAGAAGCAAATCATAATTCCCCTTCCTTTATCACTCGATTTATCGAGGCACTTTTCTTCTACGGTGCATTCTTTGACTGCTTTGAAGATTGCATGGAGAGGAAGGATCCGTGTAGAAGGACTATCGAGGGAATTCATTTTGGGGAAGGCATCATCAACATAGTTGCAGCCGAGGGGGAGGAGCGCTTCACTAGAAATGTAAAGATTGATGTGTGGAGAGCTTTCTTTGCTAGGTTGGGAATGCTGGAAATTAAACTGAGTGAGTGTTCTATGTATCAGGCAAAGTTGATTCTGAAGCAATTTGAACATGGGAGCTCCTGCAATCTTTACAGTAATGGGAAGGGCCTTATTGTTGGATGGAAGGGAACACCAATCCATTCTGTTACCTGTTGGAAGTTCAACTCTCATGATGATTATTAA

>IbGRAS21

ATGGAGGCTCTAGTCCAAGAATTCTATGCCAACACCAATGGTTTCATGTTGAATCGCCACTCAAAGCCAGCAAACCCGAAGTTAAATGGGCAAAAAAGACGAGATCTTGACAGTTTTCAGGATTTTCCGGCTGCCGGAGGAGGGGAAGAAGAAGATGAGGACTACTCCGATGCGGTGTTGAAGTACATAAACCAGATGCTGATGGAGGAGGAGGACTTGGAGAACCGGCCGTGTATGCTCCATGACTGCATTGCTCTCCAGGCCACCGAGAAATACTTCTCCGATGTCCTCCATGGCTCCGACCATGCCGATCTCAGTAGCTCTAACGCTTCTTCTGATCCCAACGACTATGGCGCCTCTTCAGATTGTCTGTTGAGTACACCTGACAGCCAATCTGGGTCGGTTTCGCCCGGTAGCGATGGATATTTTCCGGCTCCTAATCTCGTTCTACCTCACAGTAACGCCATCAATCTTGAGTTGGAAGATCGTTCACCGCCAAAGGGGAAGAGAAATCACTACTCGAACAAGGAAGATGAATCTGAAGAAAAGCAGAGGAATAAGCAGTTGGCAACGTCCACCCACGAAACAGAGCCGCCATTAGAGAAGTTCAACGAAGTTCTCCTCTGCAACATACAAGAACCCAGGAAGAAATCCGAGGACGAATTCAAGGGCGTCGCGGCGCCTCGCCGCCGCAAGAAACGAGAATCGCACAAGGAAGTGGTCGACCTGAGAGGCCTATTAACTCAATGCGCGCAGGCCATAGCGAACTACGACGGCCGGGCGGTGAACGAGCTTCTGGCGAAGATCCGACACCACTCTTCGCCACGCGGCAACGGAATGGAGCGGCTAGCGTTTTACCTGGCCAACGCGCTCGAAGCGCGTCTCAACGGCGCCGGCACGGCGATCTTCACCGTCCAATTCTCTAACAATATCTCGGCCGCGAATATCCTCAAAGCTTATCATATGTACATTAAAGCCAGCCCCTTCAAGAAGATCTCAAATATTTACGCAAATCATTACATCATGAAAATGGCGGCCGGAAAACACGCCCTGCACGTGATTGATTTCGGGGTTCTCTACGGTTTCCAATGGCCGTGTATGATCCAAAGCCTCGCCAACCGCCCCGGTGGGCCCCCCAAGCTCCGGATCACCGGAATCGATCTCCCCCAGCCGGGGTTCAAGCCGGCCGAGCGCGTGGAGGCCACGGGAGCTCGGTTAAAGAAATTCTGCGAGCAATTTAACGTCCCCTTCGAGTTCAAAGCCATAGCCAAGAGATGGGAAACCATAACCCTCGAAGATCTCGAGATCGATAGGGACGAAATTCTCGCCGTGAACTGCCTTTACCGTCTGGAGAACGTGCCGGACGAGACGGTGGTCCCCGACAGCCCGCGCGACGCCGTCCTAGGTTTAATCAACAAAATCCGCCCCGACATCTTCATCCACGGGGTCGGCAACGGCGCGTACAACTCCCCATTCTTCACCACGCGCTTCCGCGAGGCCGTCTTCCACTTCTCCACGTTGTTCGACATGTTCGAGGCCACGGTGGCGCCGGAAGACGAGGACCGGCGGCTGTTCGAGGAGACGGTGCTGGGGAGGAACGCGCTGAACGTCATCGCTTGCGAGGGGACGGCGAGGGTGGAGCGCCCGGAGACGTACAAGCAGTGGAAGGGGAGAAACAAGAGGGCTGGATTCCGGCAAGTGCCGCTAGATCAAGAGCTCGTGAAGTTAGTGAAGGATAAGGCGAGATCGGATTACCATAAGGATTTCTCGGTGGACGGCGACGGGAAGTGGTTATTGCAGGGGTGGAAAGGGCGCGTGGTTTATGCTCTCTCTTGCTGGAAACCTGCCATGGAGTGA

>IbGRAS22

ATGGCTATGGATCATCAGTTCAGCAGGTTGACTGACTCTGTAAACAGATTCTTGTTGGAGGATGAGAACATATTTTCGAGCTTAAAACGATCGCCGGATATTCCAGGCAGTTATAATGTTGACTCTTTACCTCTCGATATCGTGGATGCCCCTTTAGTTATACATGATCCCAACCTTGGTAGTTATGCTCCTACTTTGGACTACCCAGATGATCACGACTCTGATACTGTGCTCAAGTACCTTAATCAGATACTACTGGAGGAGAACATTGATGAGAATCCCAGCATGTTTTATGATCCAATTGCTCTTAAAGCTGCTGAGAATTCCTTTTATGAAGCCCTGAAAGAGAAGCCACCCTCACCTCATCAAGCCCCCCTTTTTGTTAACAGTAATGCTAGAAGCCCAGACAGCATATTGCCAAGTTCCGGTGGTTATAGTACAAGCAGCAGTAGCATCGGTAGCTGTAACGCTGACCCTCAGTGGATTGTTGATCCTGGAGAATCTAAATCCTCTGTCACTAGTTATTCTCCTGAGTTCTCCTTCCAGTCATCAAGTCAGGCTAACTCATATAGGTTAAACGGTTCTTTAAATAGCTTTAGTAGTGTTATGAATGCCCAAACGGACTCTTTTGTGAATGCTAATTTGGTTCCAAATATATTTAGTGATACCGAGTCCATCTTGCAGTTCAAGAGAGGAATGGAGGAAGCGAGTAGATTCCTTCCTACGGGTAATCAGTTGGTTATTGATTTGGATAAATACTCGTTGCCTCCAAAGACAGATGAGTTGTCTGGGGATGCTGTTATCAAGATAGAGAAGGACGAGAAGGACCGGTCCGCTAATTCCTCTAGAGGAAAGAAACACCACCATCCAGATGACAGTGGGTTAGAAGAAGAAGAAAGAAGCAGCAAGCACTCTGCAGTTTACGAGGAGGAGGTTGAGTTATCCGAGGTCTTTGATAGGGTTTTGCTGTGTACTGATAATTATGGTTGTAACATAAATGTTGAGGGAAAGCAGCAGAATGGGGCAAGCGGTGGGAAGGGCCGTACAAAGAAACAAGGGGGTAATAGAGAAACTGTGGATCTGAGGTCTCTATTATCAAGCTGTGCACAATCTATTGCTGCTGCTGATTATAGGACCGCAAATGACCAATTAAAGAAGATCAGGCAACACTCTTCGCCTACTGGTGATCCAAATCAAAGGCTGGCCAGTGTATTTGCAAATGGTCTTGAGGCGTGGCCCATCCTTATCCAGCATCTTTCTCAAAGGCCTGGTGGGCCTCCTAAACTTCGAGTAACTGGAATTGAGCTTCCTCATCCTGGTTTTCGGCCAGCAGAAAAGGTAGAACAGACTGGCCGCCGCTTGGCAAATTATTGTGAGCGTTTTGGTGTACCATTTGAGTACAATGCCATAGCAAGTCAGAATTGGGAAACAATTAAAATTGATGATTTGAAGCTTGCAAGTGGTGATGTGGTTGCTGTTAACTGTTTGTTTCGATTCAAAAACCTACTGGATGAGACTGCGGTTGCTGACAGCCCTAGGGATGCAGTTCTAAGCTTAATCCGGAAAATAAATCCAGATATTTATGTGCAAGCTAGGTTGGATTTTGAGCAAGAATTCATTGGGCGTGAAATTATGAATGTCGTTGCATGTGAGGGCATGGAAAGATTAGAGAGGCCTGAAACATACAAGCAGTGGCAGGTACGCAATATGAGGGCTGGGTTCAAGCCTCTGCCTGTAAAACCAGAACTTGTTAAGAAGCTAAGAGGTAAGGTGAAGGCGGGATATCACAAAGATTTTGTGTTCGATGAAGACGGTCATTGGATACTGCAGGGTTGGAAAGGTCGGATTATGTGTGGCAGCTCTTGCTGGGTACCTGCATAA

>IbGRAS23

ATGGAAAGCATAGATGAGGAGGATGAGTTCTTGAGCCTTAAACTTGCCATAGCCACACAACAACCATTAGGGCATGAAAGGAACAAGAAAAGGAAGAAACGAGAAGACTTAGTCGATATTTTGAGTTATGAAGAGGAGGTATATAGTCTCCTCCAAATAGGAGAGCAGATGCTGAACTCGACTCACAAAACCAGCAAAGAAAGCTTGGGAGAAGGCCTAGAAGGCCTCCACTTGATCCACTTGTTGTTGGTGGCCGCGGCGGCGGTCAACGACAACAACCTCACCTCGGCCATGGCAAACCTTAGTGAGTTGTGCCAAAATGTGTCCCCCCCCAACCCCCAAGAAGAATTCTTAGCCTTCACACACCTCTACAAAGTCTCCCCTTTGTGCCAATTCGCTCACTTCACCGCTAACCAAGCCATCATTGAAGCCTTCGACAACACTAACGACGCCTCTTTGCACGTTATTGACTTCGACATCTCCTATGGCTTCCAATGGCCATCCCTAATCCAATCCCTCTCACAACCGTTATTAAACCGGGTTTCCCTTAGGATAACCGGGTTCGCGACAACCTTAAACGAGCTACGAGAAACCGAGGCGAGGCTCCTAAGCTTCGCCAAAGGCTTCCGCAATTTGTCGTTTGAGTTTCACGGTTTGTTGAAAGGGTCCCACCTAGGAAACATAGTAACACGCGAGAATGAAACCACGGCGGTCAACTTATCCTTTCGCGTCAACAGATTATTAACCGACAACATTAGTGAAACCTTAAAAGCGGTCCGTTCTCTCCGCCCTTCAATCGTCACCGTGGTGGAGCATGACGTTTGCAGGAAATTACCTCGGAGTTTCCTACCAAGGTTCATGGAATCTCTGCACTATTTCGCGGCCATGTTTGATTCCTTGGACGATTGTCTCCCCGTGGAGAGCCACGAGAGGTTGAGCATCGAGAATCATCTCGGGAGAGAGATCAAAAGTGTGATGAATTTTGATGATCAAAGAAATGATGAGAGGGAGGTAATGGAGATGTGGAAAGGGAGAGGAGTATTGGAGAATTGTGGGTTTTGTGAGATGGAATTGAGCTCTAAGAATGTAATGCAAGCCAAACTGCTTTTGAAAATCAGAAGCCATTCTCCTTCTCCTTCTTCTGATTCTTCTTGTTCATCGTCTTGTGTTAATGGTGGATTTAGAGTTGTTGAGAGAGATGATGGGAAGGGTATTTCTCTAGCTTGGCAAGATAGGTGTTTGATAACTGCATCTGCATGGCAGTGTGTATGA

>IbGRAS24

ATGATGATGGCTGACGAAATCCTCCCTGATTTTGACTTTTGTGGGTACAGTGGCTATAGCACCACCACCACTACCACCACCTCGTCATCCGACGGTGACCACGCTGCCACGTGGAACGGAAGTTTGCCGTTGGTTGACTGGGGATTTTTTTCCGGCGACGATGAGTTTGGCGATCTTATTGATTCCATGATGGAGGGGCAGGCCGGGAACACCACCGTCGGTTTGAGTTTTCCGGCCATCCCGACGGTGGTGGAGGTGGAGAACGAGGACCAGGACGAGGACGAGGAGTATAATATGGTGGAGGATACGAAAGGATTGAGGCTGGTCCACCTTCTGATGGCGGCGGCGGAGGCGCTCACCGGCTTGAACAAGAGCCGCGAGTTGGCTCGCGTGATATTGGTTCGGCTCAAGGAACTGGCTTCCCCTCGCGACGGCTCCAACATGGAGCGGCTCGCGGCTTACTTCACCGACGCGCTCCAAGCCCTTCTCGACGGCGCGGGAAGCCTGCAAGCCAAGAGTTTCCCGGCGAGCCACGAGCACTCTCACGCCGACGCGCTGGCGGCGTTCCAGCTCTTACAGGACATGTCTCCATACGTGAAATTCGGTCATTTCACCGCGAATCAAGCGATTCTCGAGGCCGTTACGCACGATCGGCGAGTCCATATCGTGGATTACGATATAATGGAAGGAATCCAATGGGCTTCTTTAATGCAAGCTTTAATCTCCAGAGAAGACGGCCTCCCGGCCCCACATCTCCGGATCACCGCATTGTCACGCGGCGGCGGCGGCTCCAGCCGCCGCTCCTTCTGCTCGATTCAGGAAACCGGTCGCCGCCTCACCGCCTTCGCGGCGTCGATCGGCCAGCCGTTTTCGTTCCACCAGTGCCGGCTCGACTCCGACGAAGCGTTCAAGCCGTCGGCGCTGAAATTGGTCAGGGGAGAAGCCTTGATTATCAACTGTATGCTTCACCTCCCCCATTTCAGTTACCGCTCGCCGGATTCCATCGCCTCGTTCCTCTCCGGCGCGGCGACGCTAAACCCCCGGCTCGTGACGCTGGTGGAGGAGGCGGCGCCGGCGCCGCCTCCGGACGGCGGGTTCGTGGACCGGTTTATGGACACTTTACACCACTACTCTGCCTTATATGATTCACTGGAAGCGGGTTTTCCGATGCAAGATCAGGCGCGTACGTTAGTTGAGCGCGTGTTCTTGGGGCCGAAAATATCGGTGGCTATAACCCGAAGTTATCGGTCTCGGGGAGAGGAGTACGGTTGGTCGTGGGGAGAGTGGGTGCCGGAAAACGCGGGATTCCGGCCGACAACCATAAGCTTTGCGAATCGTTGTCAGGCAAAATTGTTGCTGGGACTGTTTAACGATGGGTACAGAGTTGAAGAAATTGGAAACCATAAACTTGTTTTGGGGTGGAAATCCAGGCGACTCCTCTCTGCTTCTGTCTGGACCTCTCCAGATTCTGATCTGTGA

>IbGRAS25

ATGATTGGAATGCAATATAGTAACAATAGCTTGCAAGGGAAAGGTGAGGTTGGTCGGTTTGGGTCTTTGATTTCTGCATCTTCTTCTGCGGCTCAAGATGGCAAGTTGAAGAGAGATGGAAGCTTTGGGAGCAATGAACCCATTTCTGTGCTGGACAAGAGGAGGAGCCCAAGCCCTTCAACTTCCACGTCAGCTTCCTCTTCCTCTTTTGGGTGCACGGCGGCCGTGAAGGACGCCCCCGCTCCGGCCGCCGTGGAGGAGTGGGTGGTGGGGGAATTGCAGCCTCTTGTTCCTTTTGAAAAATTTGGTCTTGGATTGGAAGATTGGGAGTATTTCTTGTCTGAATCTGGGGCCGGGGTTGGGGGTTCCGACCAGTCTATTCTCCGGTGGGTTTCCGGCGCCGAGTTTGAGGATCCCTCCTCATTACATGAGATCCAGGGCAATGCGGGTTTGGGTGATGCTGCTGATCAAACTACTACTGGATTTGGTGCCCTAATTGCCTCAGATAATTTCTTCACAAATGTGAGTTCTTCTGTTATTCCCATTTCTTCTTTGAATTCCAACATTGGGAAATTTGGTTCTACTGTCAACCATGTTAATTCTCAAGACTCCAATCTTAATTTTACACCTAATAACAATCTGGTGCCTGGGTTGAGTTTTCAAGAACCTGAGCAGAAGCCTCAGATTTCCAATTTCCAAATACCTGTAGTGAACCAAACCCAGAATGCCACAAACAGAAATGTGTTTGTCTCACCATCCTATGGGGGCATTCTTCATGAAGAACAACTGCCTCCTCCTGCAAAGCGCCAGAATTCGGAAATCTCGAATTCTCAGCTACCCGAAATTCCTTTAGTTGGTTTGAGTCATGGGTTGTTGCTTGGGAAACAACAAGATTTTGCCCAACTACAGCACCAGGGAATGATGGGCTCGGGCCAGCACTCGAGTTTGCTGGTGCCCAAGCAAGAAGAGGTGGTGATGCCACACCACCAACACCAGCAACAGGTTGTGTATGACCAGATTTATAAGGCGGCAGAGTTGATTCTGACCGGGCAATTCTCACACGCGCAAATGATATTGGCGCGGCTCAATCACCAGCTCTCTCCTGTTGGAAAATCGTTGCAGAGGGCTGCTTCTTATTTCAAGGAGGCTCTAATGTTGCCTCTCCTTATGCCTGGGTCTTCCATCTCTCTGCCTTCAAGGGTTCCCTCGCCCGTTGATTTTGTGTTCAAGATGGGCGCTTATAAGGTCTTTTCTGAAGCGTCCCCTATCCTCCAATTCATGAATTTTACATCCAACCAGGCACTTCTCGAAGCCCTTGGCGATGCAGAATATGTTCACATCTTTGATTTCGATATTGGATTTGGTGCCCAATGGTCCTCTTTCATACAAGAGCTTCCTAAAAGGAATAACGGAGGACGAGGAGGAGCGCCTTCGCTCAAGATAACAGCCTTTGCTTCTCCTTCAACTCACCATCCCGTTGAAATCAGCCTTATGCACGAGAGTTTGACGCAGTTTGCCAACGATGTTGGTGTCAAATTTGAGCTTGAGGTGGTAAACTTGGACACATTCGATCCTAGTTCTTATCAGTTGTCCTCTTTTCGACCATGTGGGAGCGAGGTGGTTGCTGTGAATTTCCCCATTTGGTCCCTTTCGAACCATCTCTCTGCTCTTCCTTCACTGCTTCATTATATAAAGCAGCTCTCGCCAAAAATTGTAGTTTCACTGGAACGAGGATGCGAACGAACTGAACTGCCTTTCCCGCACCACATTCTCAATGCCCTAAAATACTACGAGGTCCTCTTCGAGAGTATGGGTGCTGCAAAGGTGACTCCGGACATGGCAAACAAGATGGAGAGGTTCCTCTTCCAGCCTAGCATCGAGAGCATTGTGCAAGGGCGCCTCTGTTTCCCCGATCAAATGTCCCCATGGAGAACCCTGTTTACTTCAGCTGGCTTTCTACCCATGCCATTTAGTAATTTCACTGAAACTCAAGGCGAGTGCATCATGAAGCGAAATCAAGTTAGAGGGTTTCACGTGGAGAAGAGGCAGGCATCGCTCGTGCTGTGCTGGCAACGCAGAGAGTTGTTGACCGCCATGGCTTGGAGGTGCTGA

>IbGRAS26

ATGTTGGCTGGGTGTTCTTCTACATTGGTTTCACCAAGGCATAGATTGAGGAGCGAAGCATCTGAGCAGTTTCAAGCTTGTCATTTCCCTGCAATGAGCACACAGAGATTGGATTTGCCATGTAGTTTTATCCGGAAAGAGAGCTCGAGGGCTCAGGCCGTGAGGCCGGTTGGGCTCTCGGTTGAGAAGCCCGGAGAAGCCAAGACCAGTGGGTGTGGTCTGAAGCAGAATATACGTCTTCCCCCGACCCCGACAGCCATTCAGACGCCGCGTTTTGAAGGTAAAAGAGAGAGTTGGGAGTGGGAGAAGAAGAGCAGGTGCTTGAAGAGGTATGCTGCAGAGCAGGAGAGTTGTGATGAGGGTTTTGTGAGCAGAGCTAATAAGAGGAAGAAGGGCTGTGGAGAAGAAGAAGAAGATGAAGAAGAAGAACAAAAGGGTCATCATGGTTTGAGTTTGGGGCATCTGGGAAGTAGTAGTGGGGGTTTTTGGTTTCAATCAGGAGTTGGAGGGGCTAATCCCAATCCATCATCTCAGGGGCCTTTCTCTCTTTCCTCTTCAGGAGGAGATGAAGAAAGTGTGTGTTTTGTGCCAAGTGAAGTGAGGCCTCCACCTCTCCCTCTGTCACACCATCCATGGCTGGATTCTGTTGTGACTGAGATCACAGATTTTAGTGACAAGAATGTGGTGGAGACAAGCCAGGGTCCTGCCAAAGAAGCTTCTGGATCAAGCTCTTCTTCAGAGGGGGGTGGCAGAGGTGAAAGTGAGCCTAACAATGGGGTTGAGCTAATCACCTTGCTGGTGGCTTGTGTTGAGGCAGTGGGCTTAAAGAACTTTGCAGCTGTGAATCATTGCATTGGGAGATTAGGGGAGCTTGCTTCTCCTAGGGGGCTGCCTGTTAGCCGCCTCACCGCCTACTTCACAGAGGCTTTGGCTTTGAGAGTGGCATGGCATTGGCCTCATATCTTCCACATTACCCCTCCAAGAGACCTCGATCGCCCCGGCCTTGATGATGATCATAACAATGGAACTGCATTGAGGCTTTTGAATCAGGTCACCCCAATCCCAAAGTTCATCCAGTTCACATCCAATGAGATTCTCCTCAGAGTTTTTGAAGGAAAGGATAGGGTTCACATCATTGATTTTGACATCAAGCAAGGACTGCAATGGCCTAGCCTGTTCCAGAGTTTGGCTTCCAGGGCTAATCCCCCGAGTCACATTAGGATCACTGGCATCGGGGAATCGAAGCAAGAGCTGCTCGAAACTGGTGATAGATTGGCTGGATTTGCTGAGCAACTTAACCTGGCCTTTGAGTTCCACCCAGTGGTTGATAGGCTAGAAGATGTGAGGCTGTGGATGCTGCATGTGAAGGAGGGTGAAAGTGTTGCTGTGAATTGTATGCTTCAGATGCACAAGGTCTTGTACGACACTTCCGGTAGAGCCCTCGTGGATTTCCTGGGGTTGATCAGAAGCACAAACCCCATCGCTGTCGTGATGGCAGAACAAGAAGCTGAGCACGACGAGTCCAGCTTGGAATCGAGGCTTGTCAACTCATTGAAGTACTACTCTGCTGTCTTCGACTCCCTCGACTCAAGCCTGCCACTGGACAGCCCGGTCAGGACCAAGATCGAGGAGATGTTTGCCCGGGAGATCAGGAACATCATCGCCTGCGAGGGACGAGAAAGGCTCGAGAGGCACGCGAGTTTTGGGAAGTGGAGGAAGCTGCTGATGGAGCAAGGGAATTTTCGGTGCGTTGGGATCACGGAGAGGGAGCTTCTGCAAAGCCAAATGTTGCTTAAGATGCATTCTATGGAGAGCTACAAGGTGGAGAAGCAAGGGGAGGATGATGGATTGACTCTTAGTTGGGAAGATCAGCCACTTTATACAGTCTCAGCATGGACACCTTTTGATGTTGCAGGGAGTTCATCTTCTTATTCTCAGCCAAGTTGA

>IbGRAS27

ATGGCGGTTTCGCCGGCCATGCTTTTTGCTGCCGATCCGTTTCAGGTTCCCGCCGTTGAGTCCGGGTTTCAGTTCCCCAGCTTGGATAACCAGTTGGGGGCGTTTCGGTTTCCGGATTTTGGGGGCGGAGGAGGTGAGTTTGACTCGGATGAGTGGATGGAGGGGTTGATGGACGGTGGAGATTCCACCGCGAGTTCAAATCTTCATTCTGGCTGCGACACCTGGCACAACAACTCCGATTTCACCGCTCTATACTCTGCCGATCCGTTTTCTGCTTGTCCGAGTCGACTCCGTATCGCCTCGTCTTCTCCTTCAGCCCTCAATTCCGTCATTTTCACCGACAATCAGAAGAATCAGAATCACGCGCCTCCACCGCCTCAGACCTTGCCGTGGTTCCCAACGCCTCCGCCGCCTTCCGCTAAGGACTCCAAGGAAGCGGCGCCGAGAAACGACGTCGTTGCAAAAGGTAGTTCTCCGGAGAGTTTATCTTCGAAGCCATTACTAAAAGCCTTGGTAGACTGTGCTAGACTCGCCGATTCGCAGCCAGAGAATGCCGTGAAATCACTGATACGAATCAGAGACTCCGTTTCCCAGCTCGGAGACCCGACGGAGCGAGTCGCGTACTATTTCTCCGAAGCTCTTTACAATCGCCTCTCAAACTCGCCGGAAAAGCGACCGGCGAACTTCGAAGCATGCTCAGAAGAGCTCACGCTGTCCTACAAAGCTCTCAACGACGCATGTCCGTACTCCAAATTCGCTCATCTAACTGCAAATCAAGCAATTCTCGAAGCTACTGAGAAAGCCTCGAAGATTCATATAATCGATTTCGGTATTGTTCAGGGGATTCAATGGGCCGCTCTTTTGCAGGCTTTGGCTACCAGGTCCGGCGGTAAACCTGAGAGCATCCGGATCTCCGGCATTCCCTCGCCGGTGCTCGGAAACTCCCCGGCGGCGTCCCTGTTGGCAACCGGTAACCGCTTGCGCGATTTCGCGAAAGTTCTGGATCTGAATTTCGAGTTTGAACCGGTATTGGCTCCAATCCACTCGCTAAACGGGTCGAGTTTCCGGGTCGATCCGGATGAAATTCTAGCCGTTAACTTCATGCTCCAGCTGTACAATTTGCTGGACGAGACAACGGCTACCGTTGGAACCGCACTCAAGCTAGCCAACTCACTAAACCCTAGCATTGTAACTCTGGGAGAATACGAAATGAGCCTGAACCGGGTCGGGTTTCTGAAGAGATTCGAGAACGCGCTCAAATACTACTCCGCCATATTCGAGTCTCTGGATCCGAACATGACCCGAGATTCGCCGGAGAGGGTGAAGGTCGAAAGCCTGTTACTTGGCCGGAGAATCGCGGAATTGGTGAGGCCCGAGGAACAGGGAGCCAAAACAGAGTGTGTGGAAGACAAAGATCACTGGAGAATTCTGATGGAAAGTGCTGGGTTTAAGGCTGTAGCCATCAGCCATTACGCCCATAGTCAAGCAAAAATTCTTCTATGGAACTATAACTACAGTTCTTCATACAGTCTGATTGATTCTCCCCCAGGTTTCCTCTCCTTAGCTTGGAATGATGAGCCTCTCCTCACTGTTTCCTCGTGGCATTGA

>IbGRAS28

ATGAAGGAATTGGGTTTGCAAGATGAGAGAAAGCCCGATAATCATCATAATCTCCTCTTCGCCCCCTCCGAGTCACAGACTCACCATCACTCCGAGTTGACTCACTCGCTGGAGTTCGTGCCTTCCGATTTCAATTTCTCCTCACAATTCCAGGGAAATGAGAATATTAGTTCGTTTGATGAAAACTGGAGCGTTGGATTTGATTTCATCGACGAGTTGATCCAATTCGCCGAGTGTTTCGACACGAACGCCGTCCAGCTCGCTCAGGTGATTCTGCCGCAGCTCAATCAGAAGCTGAGTTCCGGCGCCGGAAACCCGCTCCGGCGAGCTGCGTTCTATTTCAAGGAAGCGCTTCAGTCCCTACTCACTGGGTCGACTCGCTCGGGTTCGAGTTCTTTTGAGGTTATTCAGACTATTAAAGCTTATAAGATCTTCTCCAATATTTCTCCTATTCCCATGTTCTCAAGCTTCACGGCTAACCAAGCCATGCTGGAAGCCGTCGACGGCGCAATGCTCGTCCACGTCATCGACTTCGATATCGGCTTCGGCTCCCACTGGGCTTCCTTCATGAAGGAGCTAGCCGATAAAGCCGAGTCGACCCACACCAAGCCGCCGATTCTCCGCGTCACGGCTTTGGTTCCCGAGGAGTACGCCGTGGAGTCGCGGCTGATCAGAGAGAATTTGGCTCAGTTTGCGCGGGAACTGAACATCGGCTTGGACGTGGATTTTGTGCTGATCCGTACATTCGAGTTGTTATCTTTCAAAGCTATCAAGTTCATAGACGGGGAAAAGATCGTCGTTTTGTTATCCCCGGCGATTTTCCGGCGGGTCGGGTCGGTTCAATTTGCCGCCGATCTCCGTCGGGTGTCTCCGCACGTGGTGGTGCACGTGGACAGTGAAGGGGCGGCGGCGAACGTCGTCGGCGGCGGGGATTGGGTTAAGAAGATTGAGAATTTCGTGTTGTTTCCGAAGATTGTGGAGATGGTTAGGGCTGCCGGCGCCGGCGCCGGCGGGATGACGTGGAAGGAGGCTTTTGTGGCCGCGGGGTTCCGGCCGGTGGGTTTGAGCCAATTTGCTGATTTTCAGGCTAACTTCTTGCTCGGGAGAGTGCAGATTGGAGGGTTCCACGTGGCAAAGAGACACGCGGAGATGTTGCTTTGTTGGCATGATAGGGCCCTCGTCGCCACGTCAGCTTGGAGGTGA

>IbGRAS29

ATGGAGTTAGGGTTGGGAGCCAATCCATGCCCTTTTTTCCCAGACCCTTGTGTTAGTAATAGATTGAATTTTGAAGATGTATATCAAGATCGGGAACTGGTTGATGGGCCTAGAGCTGATAATTCATTGGGGGTGCACAGTTTTGAGGGTTTCCATGATCCTTTATCAAGAAATGTGGCATTGACTTCTCATCAGGATGATTATGAGGATGACGATTTCTCGGATGCGGATTTGAGGTATATAAACCAGATTCTCATGGAGGAAGAAATGGAGGACAAGACGTTTATGCTTCAAGAATCTTTGGAGCTTCAAGCTAAAGAAAGGTCATTCTATGAGGCGCTTGGTAAGAAGTACCCGCCCACGCCAGAACAAAATCCAACTTTGCTTGACCAAAGCAGTTTGAGTCGAGGCAACTATGAAGTTGAAAATCACCAAAATTGCATCACCAATAGTGGTCGGGGTAGCAGCTCGGTAGTTAACCAAAGTAGTGGGAGTCTAGGTGAATATGAATCCGAAAATCATCACAATTACTACTACATAACCAGTAGCAACGAGGGGAGCAGTTATCTAATTGATCCCGGGTCGATCAACGTTACTGGTGATTACATTAGTCCTTATTTACATGGTTTTTCGGTTCCTAATGGCTCCAACTCATCGGTTAGATCCTTGAATAGTTTTAACAATAGAGTAGATGGCTTTGTTGAATCCCCGGAGCCAACAAGAGAAACCGGTGAAGTGGAGGGGACAGGGGAGAAATCTCTAAGAGTGAGAAAGAATCCTCATAGAGAAGATTTGGAAGACCAGAGAAGTAGCAAGCAGGCTGCAATTTATACAGAATCAACAATCCGGTCTGAGGAATTTGACATAGTATTACTTCATAGCATGGGGAAGGGTGAGGAAGCTTTAACAGCGTATCGTCAGAACTTGCAGAGTGCTATTTGCAAAAACATACAGCAGAATGGGAACTCGAAAGGTCCCGGTGGAGGGAAGGGCCGTGGTAAAAAGAAGAACGGGAAAAGAGATGTTATTGATTTGAGGACGCTTTTGATTCATTGTGCTCAGGCTGTTGCTGCTGATGACCGTAGGAGTGCAAATGAACTCCTGAAACAGATCAGGCAGCATTCATCGCCTTTTGGTGATGGATCTCAGAGGTTAGCTAATTGCTTTGCAGATGGTTTGGAGGCACGGTTAGCTGGCACTGGCAGCCAGATATATAAGGCCCTTGTCAACAAAAGAACATCGGCTGCTGATTATTTGAAGGCCTATCATTTATACCTTGCATCATGCCCTTTCAGGAAGATTTCGTGCTTTGCGTCAAACAAGACAACCATTATAAAATCTGCAAACTCTATGAGGGTTCATATCATAGATTTTGGCATCCTCTATGGCTTTCAATGGCCAACCTTTATTCAACGTATTGCTGCCAGGGATGGTGGGCCACCTAAAGTTCGGATTACAGGCATAGAGTTTCCCCAACCTGGCTTCAGACCAGCGGAAAGAATTGAGGAGACTGGGCGCCGTTTGGCTGATTATGCAGAGTCTTTTAACGTGCCATTTGAATACAATGCCATAGCAAAGAAATGGGAAACTATCACCCTTGAAGATCTTAAGCTTGACAAGGATGATTTTCTTGTTGTCAATTGCTTGTATCGGTTCAAGAACTTACACGATGAGACTGTGCTGGCTGAAGGCTCTAGAACTCTTGTTCTCAATCTGATAAGGAAGATAAATCCTGACATCTTCATCCATGGGATTGTCAATGGAGCCTATAGTGCCCCTTTCTTTGTAACTCGGTTCCGTGAGGCCCTGTTCCACTTCTCAGCTCTCTTTGATATGCTTGAAACCAATGTGCCTCGTGATGTTCCAGAACGGATGTTAATTGAGAGAGAGATATTTGGAAGGGAAGCCCTGAATATCATGGCTTGTGAGGGCTGGGAGAGAGTTGAAAGGCCCGAGACATACAAGCAATGGCAAGTACGTAATCTGAGGGCAGGCTTCACTCAAATACCTTTTGCAAATCTAATCATGAACAAAGCAAGGGACAAGGTGAGAACAGGCTACCACAAAGACTTTGTGATTGATGAAGATGGCCAGTGGCTATTGCTAGGATGGAAAGGGAGAACGATTTATGCCATTTCTTGCTGGGTTCCTGTTTGA

>IbGRAS30

ATGATGCAGCCACAGCTCCTTCATCAGCCCTCGTGGCCATCCTCTTATAACGTCGATTACTCAACTCTCCCTCGAACTAGGTTGTGCGGTTTACATGAAGATTCCTATGTAAGAGACAACTATACTCACTCTTCACTGCTCACCACAACTGACTCCTTTGGAGTTTCCTCAGTCTCCGACTTAACGGCCTTGTTTCCAGATGCGTTTACTGAACTTTCAAGTTTGCAATTGCAAAATGATATGCAAATACTGCCCCCGCTCCCGGATGGTGAGTTTGAGGATGTTTGCAAGTGGCTAAACACCAATGACAGCGAAGATGGCATATCTTCTGAGGCTTCGTTTTCGATTGCTCAATCATCTGATGCAAACCGGCTGCCAGCTATATTTCCAGGGAGTGGAGTGGAAGTAGACACTCAATTGAGCCTTCATCACTTGCTGGGGGCTTATGCAGAGGCCATGGAAAACGGGCACGAGGAACTTGCAGAGGTTATTGTGAAGCGTATAAGGGGAAAGGCGAGCCCTTTGGGCGAAACACTAGAGCGTGTTGCACACAGCCTATTTGAATCCACTGAAGAAGATCAAGAAGGGTACCTAAGACAAGAATCAAGCAAGAACTTTGAGCAAGCATTCAGGGCATTCTACCAAATTCTCCCATTTGGGAGATTTGCTCACTTTGCTGCAAACTCAGCCATTCTTGAAGCCTTGCCAGATGATGCAGAAACAGTAGTTCACATAATAGATTTTGATATGGGAGAAGGGGTTCAATGGCCTCCGGTGATCGAAGCCATGGCTCGAAAAAGAAGGGCTTTAAATTTAAGACTCACATCCATAAAACCCAACCATGAATCCACCAGTAACCAATATGAGGAGACAAAGAGAAGGCGTTATGATCACGCAAAGCCATTCGCCCTAAATCTGCAGATTGAGGAAATGAGTGTTGAGGAACTCGCCATCGAAACGAAAACGAAGAAGAACAAAGGCCCAGGGAAACAATGGTTGGCTTTCAACTGCATGTTTAGGCTCCCACACATGGCAACAAACATGCCACAAACAACTCAAGCCATGGAGTTCCTAAAGATAGCTAAGCAACTTTTAGCCCATTCTGAAACCCAAACCGGAATCATCATTTTCGCCCACGGAGAATCAGAGGGCTGCAACACTCCCACTTCAAACTACACCTTCTTTCTTTAA

>IbGRAS31

ATGGTCATGGAAAGAAATGTGAGGGTGATATGTGAGGCTTCAGGAGATAAATTGAAGGGTAAAACCCAATCTGTTTTCCTGGATCCAAATTTGATTAATAGTTTGAACATTAGTGAAACCCTCGCAGACTCGAATGGAATGACCTCGAACCTATGTGATGAAGGCCTGCCAAATTTTCTGGACCCTACTGTTATTGACAGCTTGAGAATTAGCCACACCTTGGTTGATCGAAACCGAGGTATTGAAAATTTATATGGTGAAAGCATATCAAAGTTTTCGGACACGGTTTTGATTGAAAACTCGAGAGGTGAGCAAAGCCTGCACATAGGAAATTCATGCCATCAAGGTATACCGAGTTTTCCAGATCCAATTTTGGTTGGTAGTCTGAATGTTAGCCAAACCTGTTTGGATCAAAACGAACTCGCTGGAAATTTAAATGGTCAGGCTGTATCAGATTTTCTGGATCCGGTTCTGATTGAGAACTTGAGAATTAGCCAAACTTCGGTGAATCAAAATGGGCTCACGGGAGTTTTAAATGATGATGGTCCACCATGTGTTATGGATCTGCATTTGATTAACAGTCTCGGAGTTTGTGAGGACTTGTTGGATCAAAATCAGTTTGCATTTCCACAGCTTCAATCTGATCCGAGGTTAAACGTTGTAGCCCCATCTAACGAGGGTGATGCTCACGAGGACTTTGATTTTAACGATGGAGATCTTAACTATATTGGCCAGATGCTCATGGAAGAAAACATGGAAGAGAAGGTGTGCGAGAAGTATCCACCGCCTGCTGGTCATTATACAGTTTCCAATTCCGATCAAAATGGAATTTTGGTTGATGGAAATTATCACAATTCTATCAAAAATGCTAACTCTCGTTTGTTGTGTCCCAAAATGGATCCGTATCCCAGTGAATCTGACATTTATAGTGCCCAATGTATTCAAGTTAGTGTCCCATTGCAGACTATATCTCAGTCGTCCTACAGCACATCAAGCAGCTCGGGCACCGTAAATGATGGGCACGTTGATTCTCCTGTGAGTTTGGGTTATAATGGGAAGAAGCGAAGTGCAGAGAAAAATGAAGTGGTAGTTAATATGGAGAAGAGATACGAGAATCAGCACTCTCGTGAAATATCCAAAGCGAAAAAGAATCTGCACAACGAAGATCTGGAAGCGTTGGAGGGGAGAAGTAACAAGCAGTCAGCAGCCTTGCAGATTGTATCGATAAAAAATGCAACTGACAATGATGATTCCAAGGCATCCAATGGGGGGAAGAAATCCCGAAGAAAGAAACAAAGAGGTAAAAGAGACACGGTGGACTTGAGAACGCTTTTGACACTTTGTGCAGAAGCTGTTGTGGCTGATGACCGTAGGAATGCGAGCGAGTTTCTGAAGCAGCTCAGGCAACACTCGAGTCAAACGGGGGATGGTATGCAAAGGCTGGCTCATTACTTTGCAGATGGTCTCGAGGCAAGGATGGCTGGTTCCGGGACCCAAATATATAAAGCCCTTATAACCAAACCTACATCGGCTGCTGATATCCTAAAAGCGTATCAGCTATTTCTTGCTATCTGTCCATTCAGGAAGATCTCGAATTTCTTCTCGAACAAGACAACCATGAATCTAGCCCAGAGTGCAACATCTGTGCACGTTATTGATTTCGGTATCCTTTATGGTTTTCAATGGCCTTGCTTCATCCAGCGTCTTTCCTCTAGAAAGGGTGGACCTCCAAGGCTTCGTATAACTGGAATTGATCTTCCACAGCCGGGTTTTCGACCAGCAGAGAGGGTTGAGGAAACAGGGAAACGCATGGCTAATTATGCTGAGAGGTTCAATGTTCCATTTGAATTTAATGCTATAGCAAAGAAGTGGGAAACGATTAAAATCGAGGATATAAAGATAAACAAGGACGAGGTTCTTGTAGTTAACTGCCTATTTAGGCTTAGGAATCTCCTTGACGAGACCGTGGTTGTAAACAGTCCGAGAGATATTGTTTTGAAGCTCATCCGGGAGTTGAATCCACATGTTTTCATACAGGGGATTGTCAACGGTGCTTACAATTCCCCCTTCTTTATCACACGATTTCGAGAGGCTTTATTTCATTTCTCGTCTTTGTTTGATATGCTCGACACTAACGTTCCCCGCAATATCCACGAGAGGATACTGATTGAAAAGACGATATTTGGGCAGGAAGCAAAGAACGTTATTGCATGTGAAACCGCAGAGAGGGTCGAGAGGCCAGAGACATACAAGCAATGGCACGTTAGGAACATGCGGGCAGGGTTTCTTCCGCTTCCTTTGAACAAGGAGATCATGAAGATGTCCAGGGACCGGGCCAAGGTATACAACAAGGATTTTGTAATTGATGAAGACGGAGAATGGCTTCTTCAAGGATGGAAGGGACGCATTGTCTATGCACTTTCCTCCTGGAGACCCGCTTCTTGA

>IbGRAS32

ATGTCGTCTGGATTTCCCGGCAGCGTTCAGGAATTTTACGGCGGTCCCGACGGGATCTCCAACGGAAGATCTGTCCCCGTGGGGAGTAATATCGGGAATCTTATGCAGCAGGGGGGAGTGCAAGTGCAAGTTCCTTATGGCTCTCAGCTCCCCGGAATTGTGTCCGATTCGGCTTCTCAGATCGCTCATCGGAGGTCCGATTTGATCGGGAAAAGGTCGCTGGTGGAGTTTCAACAGCAGCAGCAGCAACTGCAATTTCTCCAGCAACAGCGGCAAGGAGCGCTAGGGCTTTATCTTCGCAACGTTAAGCCTCGGACTTATCAGCACAGTTCTCCGATATCTCCGCTCTCGCCGGTGGATTTGTCGGCGCTGTCCTCGATTTCTTCGAATTCGAATTCTCCGGCGATGAACGCGAGGTACGGCGTTCCGATTCTTCAACAATTCCGGCCTCAACTGTCCATGCCTGCTGGGAGCGTTAACATTAACGGTGTTTTACCTTCTGGGCCCGGTAACCCTAATTATGCTCCCGGGTTTTCCTTTCCAAATTCGGTCCAAAACAGGGGCGGTTTAGGTTCAGAACGGGCCGGGCTGGAAACGGAGAAGAAGATGATGAATCGGCTTCAGGAGCTCGAGAAACAGCTTTTGGATGATATCGATGAGGAGGAAGGCGATACTGTTTCCGCCGTAACGAACAGCGAGTGGTCGGAGACGATACAGAGTCTGATTAGCCCGGCCCAGACTCAAACCCAAACCCAAGGCCCGAACCAGAATAACAATAAACCGCAAATTTCGCCGTCTCCAACATCGTCGACGTCGTCGTGTGCGTCATCCATGGAGTGCCCTGCGATAACATGCCCGAAGCAGACCATATCAGAGGCGGCGACCGCGATAGCCGAAGGCAAAAACGAGGTAGCGGCGGAGATTCTCACGCGCCTCGCCCAGGTGGCCAACGTGAATGGCACCGCCGAACAGCGACTGACAGCTTACATGACTTCCGCTTTACGATCGCGCGTGAGCCCCGTGGAATATCCTCCCCCAGTATCGGAACTGCTCACCAAGGAACACGAGCTCTCCATACAGAAACTCTACGAAGCTTCGCCGTGCTTTAAGCTAGGGTTCATGGCGGCGAATCTAGCCATCCTCGACGCCGTATCAGACCAGAGATTCTGTAAGCTCCACGTCATTGATTTCGACATCGGCGAAGGCGGACAGTACTTGCATTTGCTCTACGCGCTCGCGGCGAGGAAAGCTGAAAACCCTACGGTGTTGAAGATCACCACGTTCGCCGACGTCCCCGGCGGCGATCAGCAATTGAGAGCCGTGGAAGAGGAGCTTCAGAAACAGGCGCAGACCGCCGGAGTTTGCTTGAGTATCAACATTATTCCGTGTTCAAACACCGAGCTGAGCCGCGAGCGCCTCTCCGTCGATCCCGACGAGGCTCTGGTCGTGAATTTCGCTTTCAATTTGTACAAACTCCCTGACGAAAGCGTGACGACGGAGAATCGGCGCGACGAGCTGCTCCGCCGCGTGAAGGCGCTGTCGCCGAAAGTGGTTACGGTGGTGGAGCAGGAGCTGAACGGCAACACCGCGCCGTTCGTTGCGCGCGTGAACGAGGCGTGCGGGTACTACGGCGCGTTGTTTGACTCGCTTTACCTGACCGTTTCGCCGGAGAATATTTACCGAGTCAGAATCGAGGAGGGACTGGGTCGCAAAATGGGTAACTCGGTTGCTTGCGAGGGGAGGGACCGTGTTGAAAGATGCGAAGTGTTGGGCAAGTGGCGGGCCCGGCTCAGCATGGCCGGGTTCACCGCGATTCCGATGAGTCAACACGTGGCCGACTCGCTCCGGTCCAAGCTCAACTCGGGGCCACGTGGCAACCCAGGATTCACCATAAGTGAACAAGCCGGGGGTATCGGCTTCGGCTGGAAGGGACGAACACTCGCCGTCGCATCCGCCTGGCGTTAA

>IbGRAS33

ATGTGTTGGAGAAAAGTGGGTGTCCGGGGATCCTATGCAGAGGCTAAGTGCATACTTGTTGAAGGGCTTAGAGCACGGATATTGTCATCCGGAAGCATAATCTACAAGAAGTTGAAGTGCAAAGAACCAACTAGCTCAGAGTTGCTGTCTTACATGCAAAACAGAATCCACATCATTGATTTCCAGGTTGCGCAAGGAAGTCAGTGGATGTTCCTCATCAAGTCTCTTGCAGATCGACCCGGTGGACCCCCATTCAGCCTACGCATCACAGGTGTCGATGATTCCCAACCATGCTCGGGTGGAGGACTTCAACTGGTTGGTGAAAGGTTAGCAAATTTGCAGAGTCATGTGGTGTGCCCTTTGAATTCCATGCAGCAGGTATTTCTGGCTCTGAGGTTGAACTCGAGAACCTCTGGATTCAACCCGAGAAGCAGTTGTGTTAATTTTCCTTACATGTTACATCACATGCCAGACGAGTGTGAGTACCATGAATCACAGAGACCGCTTATTGAGACTGGTAAAGAGTCTGTCCCCAAAATTGTGACCCTCTCAACAAGAATCCAACACCAACACCACTCTTTCCTTCCAAGGAATATAGCCCGAATTTCGATTGCAGAAGGCCATGGCGATTGTATCTTGGATGGAAGAACCGCTTGGCATCAGCCTGGAGATGATACTCCCTCGACGCATTTTCAACTCTCTCTGTTTGCATACCCATCCATCTTTAACCTTTTCGCTTTGCTTGGTATGATTAGAACAGAGTTCTTTCTCTGA

>IbGRAS34

ATGCAAGCCTCCCAGGGACCTCAATCGTCTAGCAGTGTACAGAGGTTTTACCATCAGCCTCAGCAGCAAGTTGAGCAATATTATGCCACTTTCCATGTTTTGAACAACAATGCTTCCAATGACAGTGGTAGTGTAGGGGAACAAGGTTTTTTCCAGACCCAAAACGAGCAGTTCTTCACTTTGGATTCAGCACCTGCTGCGATTGATTCCGTCTATTATGATTCACCCCCTGCTGCCAGTGTTTCTTCCAATCGGAGCGCCTTTTCTCCCCAATGCTCTCAGTCATACATGTCTGATATGCATCACTCCTCAGATAATACTACTTGTGGTTCGCCTTTAAGCGGGTGCTCTGGTGTTGTTGATGGCAATGAACTGAGGCATGTGTTGCGAGAATTGGAGAATAAATTGCTAGGCCCTGAATCTGAAATTGATGATAACTACAGTTGCTCCTTTAGTGATGCAGTCCCCAAGTCTTCTTCAATGATGAAGTGGAAGAGAATGCTGGACATAGCTCCGAGTTTGGACACGAAAGAGCTCCTCTTTGCCTGTGCTGAGGCAGTATCAGATGCCGATATATCAACGGCAGAAGTTTTGATGAATGTGTTGGAGAAAAGGGTGTCGGTGTCCGGGGATCCTATGCAGAGGCTAAGTGCATACTTGTTGGAAGGGCTTAGAGCACGGATATTGTCATCCGGAAGCATAATCTACAAGAAGTTGAAGTGCAAAGAACCAACTAGCTCAGAGTTGCTGTCTTACATGCAAGTTGCGCAAGGAAGTCAGTGGATGTTCCTCATCAAGTCTCTTGCAGATCGACCCGGTGGACCCCCATTCAGCCTACGCATCACAGGTGTCGATGATTCCCAATCCGCTCATGCTCGGGGTGGAGGACTTCAACTGGTTGGTGAAAGGTTAGCGAAATTTGCAGAGTCATGTGGTGTGCCCTTTGAATTCCATGCAGCAGGTATTTCTGGCTCTGAGGTTGAACTCGAGAACCTCTGGATTCAACCCGGAGAAGCAGTTGCGAGACAGCTTATTGAGACTGGTAAGAGTCTGTCCCCCAAAATTGTGACCCTCGTCGAACAAGAATCCAACACCAACACCACTCCTTTCCTTCCAAGGTTCCGCGAAACTTTAGACTACTACACAGCAATGTTTGAGTCAATAGATGCAGCTTGCCCAAGAGATGACAGACAGCGCATCAGTGCAGAGGAGCATTGCGTTGCACGGGATATTGTCAACATAATAGCATGCGAGGGGGCTGATAGAGTGGAAAGACACGAACCGTTTGGCAAGTGGAGTATGAGATTTACGATGGCTGGATTCACTCCGTGCCCATTAAGTCCCTCGGTTGGTGAGGCCATGAGGCACATGTTGCAGGAATATAGCCCGAATTTCGGGATTGCAGAAGGCCATGGCGCATTGTATCTTGGATGGAAGAACCGCGCTTTGGCATCTTCCTCAGCCTGGAGATGA

>IbGRAS35

ATGATGCAATCAGTGCCTTTTCAGCCCTCCTGGCCATCCCATAATCTTCAGTTCTCAACTTCCCCTCAGACTATTTTCTGTGGTTTATACGACGAAACCTATATTCACTCTTCCACTGCCATCACAACTGACTCCTCTGATAGTCCCTTGTACTCGAGTTTTGCAGCCTTATTCCCAGACGCGTTTACTGAGCTCACGAGTTTGCAAAACGATACTGCTATGGATCGTTTAGAGTCCATAGAGTTTGAGGATTTCTGCGAGTGGTTAAATAACAGTGACAGTGAAGAGCAGCCAAAGGGAGATATGTGGAGTCCTAGCCTCTCAGTGGTATCGAGTGAGGCTTCGATGGTTTTACCATCAAGAAACACGGCAGTCACAGTTCCAGGAACCGGGATGGAAGTAGAAGGCCTAACGAACCTTCATCACTTGCTCGAGGCTTATGCAGAGGCCACAGAAGACGGGCACGAGGAGCTAGCAGAGGTGATTGCGAAGTGCATAGCTGGAAAAGTGAATCCGTTGGGAGAACCAATCGAACGTGTTGCTTTCAACTTGTTCCATCCCTCGGAAGATCTCCTAAAAGAATCAAGCAACAATTTCGAAGCAGCATTCATGGCCTTTTACCAAATTCTCCCATATGGGAGATTTGCTCACTTTGCTGCTAACTCAGCCATCCTTGAGGCCTTACCCTCTTCTGCAGAAACAGTTACCATAGTAGACTTTGACATGGGAGAAGGCATCCAATGGCCTCCACTCATCGAAGCCATGGGCCCGAAACCAATATCTTTGAAACTCATATCCATAAAAACAGAACAAGAGCCAACCAGTAGTAGTCGCTGGAGATTCGAGACTACACAAAGAAGACTCTATGATCATGCAAGGCAATGCGGCCAAAAATTGCAGGTCGAGGAGATGACCATCGAGGAATTAGTCACTGAAACGAAACGAAACACTGGAAAACAATGGTTAGCCTTCAACTGTATGTTCAGACTGCCACACATGGCTAAGAAGCAACCAAGAAGTCAAGCCATGGAGTTCTTGAAGATTGCCAAGGAACTGTTATCCTCTTCAGGAATTGTTGTTTTTGCAGACGGGACCCCGGGGCAATGGCGATTCCTCTCCTGGTTACACTTCTTCCTTTAA

>IbGRAS36

ATGGTCATGGATAGGAATTTTGGAGATTTTTATCAAGACACATTTGGGGGTAAATCAGGGGATGAATCCCTGCAATTTTTTCTTGATCAGGAAGCTATTAGTGGGCTAAGTGTTAATGATCCCTACATGGATGTGAATGTGGGGAAAAATTCAAAAGATGGAGGCGAATCGAATGTTTTGGATCCGAATTTGGTGAGCAATGGGAGTGTTGGTGAGACTGTGGTTGGCCAAAACCAAATCACCGAGAATGTGGATGCTGTAACCCCACCATCCATTCCATATCCAAATTCGAATAATGGTCTGGGAATTGGTGGTGGTTCTTTGGAAGATAGAAGTGAACTTGCCTTTCCACCGCTTCAGTCTGATCTGAGCTTAGATGTTGTAGCCCCGTCAAGTGAGGGCGATGGTCACGAGGATTATGATTTTAGTGATGTAGTGCTCAAGTATATTAGCCAGATGCTTATGGAAGAGGAAATGGGAGAGAAGGCCTGTATGTTTCAAGAATCTGCTGCCCTCCAAGCTGCAGAAAAATCTTTGTATGAGGTTATTGGAGAGGAGTACCCACCGAATTCCCTTGATCAAAATGGAAATAATGGCGATGGTAACAATGGTGATTCTGGTTTGGTATACCCGAATTGGGATCCTGATCCCAGTGAGTCTGAAAATTCTACTGGACAACATGCCCCTGTTGGTGTCACTTTGCGGACTAATTCTCAGTCATCCTACAGTTCATCAAGCAGCTCGGGCACTGTAAATGACGGGCATTTGGATTCACCCGTGAGTACACTCAGGATCCCTGACGTTCATGTGGATTCTCCTCTGAGTACACTCAGGATCCCTGAGATATTCAACAGCACTGAATCTATTATGCATGGGCTCGGGAAGGGGCAGAGCACAGATGGAATTCATGGAAAGGATATGGCAGTTAAGGACGGGAAGAATAACGAGAATCAGCTCTCTCGTGAAGGGTCGAGAAGGAAGAAAAATCCGCATTATGAAGATGAGGATTTGGATGAGGGGAGAAGTCACAAGCAATCAGCAGTGTCCTCTGAATCAACCGTTAAATTAGAAATGTTTGACAAGGTTTTGCTCTGCAGTGGGGGAAAAAACGAATCTGCTCTTCGCCAGTCTTGGCAGACCGTGTCAAGTAAAAACGCAATGGATAATGATCTTCCAAAAGGATCCAACGGGAAGAAATCCCGGGGGAAAAAACAAGGTGGGAAAAGCGAGGTAGTAGACTTGAGAACCCTCTTGACACTTTGTGCACAAGCTGTTGCTGCTGATGATCGAAGGACCGCCCATGAGTTTCTGAAGCAGATTAGGCAACATTCGAGCCAAACAGGTGATGGAATGCAAAGGGTAGCTCATTATTTCGCGGATGGCCTCGAGGCACGAATGGCAGGGTCCGGGACCCAAATCTATAAAGCACTTATAACCATGCCTACATCAGCTGCTGATGTTCTGAAAGCCTACCAACTATATCTTGCTGCCTGCCCGTTTAGGAAGATCTCTAACTTCTTCTCAAATAAGACAATTATGAATGTAGCTAAAGATGCTACATCAGTTCACATCATTGATTTCGGTATACTTTATGGCTTTCAATGGCCTTGCTTCATACAACGCCTCTCGTGTAGACCTGGTGGACCTCCCAAGCTTCGTATAACCGGGATTGATTTCCCACAGCCAGGTTTCCGGCCTGCAGAGAGGGTTGAGGAAACTGGGCGACGCTTAGCCAATTATGCTGAGAGGTTCAACGTTCCGTTTGAGTTCAATGCCATCGCCCAGAAATGGGAAACAGTTAAAATCGAGGATCTTGGGATCAACGGGGACGAGGTACTTGTGGTGAACTGTCTATATCGGTTTAGGAATCTGCTTGATGAGACGGTGCTTGTGGACAGTCCAAGAGATATCGTTCTGAGCCTCATCAGGAAGCTGAATCCAGCTGTTTTTATAACGGGGTGTGCTAACATCCCCCGAGAAATTCACGAGAGGATGCTGCTCGAGAAGACTATATTTGGTCGGGAAGCAATGAATGTCATTGCGTGTGAAGGTGCTGAGAGGATTGAGCGGCCTGAAATATATAGGCAGTGTCAGGTTCGACACATGAGGGCAGGGTTTCGCCAACTTCCTCTGAATGATGAAATAATGCAAATGTCAAGGGATCGTGTCAAGGCATACCACAAAGACTTTATAATCGACCAGGATGGAAAATGGCTGCTTCAAGGATGGAAAGGGCGCGTTATATATGCGCTCTCAACATGGAAAGCGGCTTATTAA

>IbGRAS37

ATGAGTCCCCATGACTCTTCCATAACAAGCAGCAGCGGCAGCTCCTCCTCCTCTTGCTCCGCCGCTCACGTGGACCAGATCGACAGCCTCCTCGCCGGCGCCGGATATATGGTCCGCTCCTCCGACCTCCGCCACGTGGCTCAGCGTCTCGAGCATCTGGAGAGTTTCATGGTGAATAGCCCTATGGGGTCCGAGATCTCTCAGCACCTAGGAAACGACGTCGTCCACCAGAACCCTTCTGATCTCGGGTCGTGGGTCGACTCCTTGCTGTCGGAGCTCCACCCGCCGCCCGTACCCGAATTCGCCGCTCCGTGCCCCTCGGATTCCAATTACGTCGCCGCGGGTCCCACCGGGTGGAGCGAATGTGAGGCGATGCAGCAGCAGCCACAGATTGTTTCCCCTTCGCATCTAACGGTTGTAACGGCCACGGAGCAAGAAGATTCCGGCATCCGATTGGTGCATGCGCTGATGACGTGTGCGGTTTCCGTGCAACGTGGCGAGTTCTCCTTGGCCGGATCCTTGATCGACGAATTGCAGCTGCTCCTGACACGTGTCAATTCCGGCTGCGGCATCGGCAAAGTCGCCGGCTACTTCATCGACGCCTTGAGCAAACGGCTGTATACGCCGCAAGGGGTTAGCCTAATGGGATCGGCTTACGAGGACGAGATTTTGTATTCCCATTTCTACGAGGCGGCTCCGTATTTAAAATTCGCTCACTTCACGGCTAATCAAGCCATATTAGAGGCATTCCACGGCCACGATTGCGTCCACGTCATCGATTTCAACTTGATGCATGGCTTGCAATGGCCCGCATTGATTCAGGCGCTGGCTTTACGTCCCGGCGGGCCGCCGTTACTCCGGTTAACCGGCATTGGCCCGCCTTCCCCCGACGGCCGCGATTCCTTACGTGAAATCGGGCTCAAATTAGCGGAATTGGCGCGTTCCGTTAACGTCCGGTTCGCCTTCCGCGGCGTGGCGGCTTCGCGGCTCGACGATGTCAAGTCGTGGATGTTACAAGTCGGGTCAAATGAAGCCGTGGCGGTGAATTCGATAATGCAGCTGCACAAATTACTCGGCCCGGACCCGATCCGCGGCTCGCCCATCGATACGGTTCTGGGCTGGATCAGGAGTTTGAACCCGAAAGTTGTAACCGTTGTTGAGCAGGAAGCGAACCATAACCAGCCCGAATTTCTGGACCGGTTCACCGAATCTTTGTACTACTACTCCACAATGTTCGACTCGCTGGAGGCTTGCCCGGCTCAGCCGGAGAAAGCCCTAGCCGAGATGTACATTCAGAGAGAGATTTGCAATGTGGTGTGTTGCGAGGGCGTGGCTCGGGTGGAGAGGCATGAGCCGTTGGGAAAGTGGAAGGCTCGACTCACCGGAGCCGGCTTCAAGCCCCTCAATTTAGGCTCTAATGCATTCAAGCAAGCCAGCATGTTATTGACTCTATTCTCAGCCGAAGGCTACAATGTTGAAGAGACTGAAGGGTGTTTAACACTAGGGTGGCACAGCCGACCTCTCATCGCGGCTTCGGCTTGGCAGGCCAGCCGTGACTGA

>IbGRAS38

ATGATGCAATTCACGGAGACTTTACCGGCGCCGTCGCACCAAATCTCACCGTTCTCTAGCCTGGTGACGAACAAGAATCAGGTTCATCGGACGCGACCATGGCCGGGATTCCCGACATCGTCCAAGAACCTAGGAACCAGCATAGGGGATGCTAACTGCATGGAGCAGTTGCTAGTCCACTGTGCGAATGCGATCGAGAGCAACGACGCCACTCTAGCTCAGCAGATCTTGTGGGTCCTCAATAATATAGCCACTCCCGACGGGGACTCCAACCAGCGCCTCACTTCCGCCTTTCTGCGGGCCCTCATAGCCCGGGCCGCCATGAGCGGCACGTGCAAGCTCCTGGCCGCCATGGCTGACCTCCACCCCGGCCTCAACCTCAACATGATGGACACCCACAGATTCTCAATCATAGAGCTGGCCAGCTTCGTTGACCTCACGCCCTGGTACCGCTTCGGCTTCACCGCCGCCAACGCCGCTATAATCGACGCCGTGGAGGGCTACTCCGTCGTTCACATTGTCGACCTAAGCTCCACCCATTGCATGCAGATCCCTACTCTTATCGACGCCATCGCCGCCCGCCCCGAAGGCCCGCCGTTGATCAAGCTTACACTCGCCGCCGCCGCCGACGAATTCCCGCCCATGCTCGACCTTTCCTACGAGGACCTCGGCGCCAAGCTCGTCAACTTCGCCAGGTCCCGAAACGTGGAGTTAAATTTCACGGTCGTCCCCTCCACTCCTTCCGACGGGTTCTCCTCATTGATCCAGCAGCTCCGCCTGCAAAACCTCGTACGAGCCGAAAACGGCGAGGCGCTCGTGATAAACTGTCACATGATGTTACACTACATCCCCGACGAAACCCTGTACGAGAATTCCCATTCTCAATCTCAATCCGCCGCTTCCTCGTTCCGGACGATGTTTCTCAAATCAATCCGGAGCCTAGTCCCGACAATCCTTCTCTTAATCGACGAGGACGCGGATTTCACCTCCAACAACCTCGTCTGCAGACTAAGGTCGGCTTTTAACTACCTATGGATTCCGTACGACACGGTGGACACGTTCCTTCCCAGAGGCAGCAAGCAACGGCAGTGGTACGAGGCTAACATCTGTTGGAAAGTGGAGAACGTGATAGCCCAGGAAGGCGTCCAGAGAGTGGAGCGGCTGGAGCCCAAAACGCGGTGGGTGCAACGGCTGAGGAACGCCAATTTCAGAGGCGTCCCGTTCTCTGACGACGGCGTTTCCGAGGTGAAGAACATGCTGGACGAGCACGCCGCCGGCTGGGGACTCAAGAGAGAAGACGATGATGTTGTGCTCACTTGGAAAGGCCATAATGTCGTGTTTGCCACTGCTTGGGTCCCTAACTAG

>IbGRAS39

ATGGATACTTTGTTTAGACTGGTGAGCCTTCAGCAATCCGATCAGTCGTTCAACTCCAGCAGGACTTCCAGCAGCTCAAGATCTTCCAGGCACAACGACAACGCCAACGCCAATAATAATTACCATCATCACCAGGAAGACGAAGAATGCTTCAACTTTTTCATGGATGAAGATGACTTCTCTTCTTCTTCTTCCCACAACAAGCACCCTTACCCGCCATCATCATCTTCATACCATCATCAGTATCAGCACTTTTCCAACACCCCCACTCCCACCACCACTAGCAGCACTCCTCCCCACTCCTACTCCCACCACCACCAGCCCCCCACGTTCGACCCTAATCTCGAATTCGGTTCCGATTTTTCCGGAAAGTGGGCCACCGAGATTCTCGTGGAGACCGCGCGTGCCGTGGCGGACAAGAATAGCGGCCGCGTGCAGCAGCTGATGTGGATGCTGAATGAGCTCAGCTCGCCCTACGGCGACACTGATCAGAAGCTCGCGGCTTACTTTCTCCAGGCGCTGTTTAGCCGCATGACGGATACCGGCGATCGGACGTACCGGTCCTTGGTGTCCGCCTCCGACAAGACTTGCTCCTTCGAGTCAACCAGGAAGACGGTGTTGAAGTTCCAGGAGGTGAGTCCTTGGACGACTTTCGGTCACGTGGCTTGTAACGGCGCGATCATGGAGGCTTTCGAAGGGGAGAACAAGTTGCATATTGTTGATATTAGCAACACGTTTTGCACGCAGTGGCCTACTTTGCTTGAAGCGCTGGCTACTCGGACGGACGAGACGCCTCATCTCCGCCTCACCACGGTGGTGGTTAACAAAGCCTTCGGCGGCGCTGGAGGCGGAGGCGCGGCGTCGATCCAGAAGGTTATGAAGGAGATCGGGAATCGGATGGAGAAGTTCGGTAGACTCATGGGAGTGCCGTTTAAATTCAACGTTATTCATCATTCTGGCGATTTATCTGATCTGGATTTATCCGCGTTAGATATTAAGGAAGACGAAGCCCTGGCGATCAACTCCGTCGGCGCGTTGCATTCCGTCACGGCGGTGGGGAGCCGGCGGGATTACTTGATATCCGTTTTCCGGCGCTTGCAGCCGAGGATTTTGACGGTGGTGGAAGAAGAAGCGAACGTGGACGTAGGAGTTGACGGCTCCGATTTCGTTAGGGATTTCCAGGAATGTCTGAGGTGGTTTAGGGTTTACTTCGAGTCTCTGGACGAGAGCTTCTCCAAAACCAGCAACGAGCGTTTGATGCTAGAGAGGCAAGCCGGGCGAGCGATCGTCGACCTAGTGGCCTGCCCGCCTTCCCAGTCCATCGAGCGGCGCGAGACGGCGGAGCGGTGGTCACGTCGCCTCCATGCAGGCGGTTTCGCCCCCATTTCCTACAGCGACGAAGTCTGCGACGACGTGCGCGCCCTGCTCCGAAGGTACAGAGAAGGCTGGACAATGGCACAGTGCTCCGGCGACTCATCCGCCGGAATATTCCTATCGTGGAAGGATCAGCCGGTGGTTTGGGCTAGTGCATGGAGACCTTAG

>IbGRAS40

ATGAAAGTGTCCTTTACTTCTAATGAAAACGTGAGTTCAAAAACGTCGACGTTGAGTAGCTGTGGGAGTAATATCGGTATCCAGGCCCCACCTTTTCCGGCCGCCGCCACCGCCGCCGCCAGGGTGGTCAGTTACGAGCCGAAATCTGTTCTCGAGCTCCGCCGCAGTCCCAGCCCTAACGTAACAGAAAACGCGCATAATCCGGCACTAGATGCGGCTGATATCTCGGCCGGTTGCGACGACCCTCTCCAGCTGGCAGATCATGTGCTAACCAACTTTGAAGATTGGGACTCTTTGATGAAAGACTTAGGTCTGAAGGAAGATACAACAAAACCTAATTCGGAGTCGCTCCAGACTCAGTTCCCCAGTTCCCGCTCGCTCACTCGCTCGATTCCGCTCAGTTCCTCCCGTCTGAGCATTTCAGCTTCTCCGATAACGTCACGGCGGCGCAGTACCCGCCTCCGCCGCTCAGCGCCGGGGAATAGTAATTTCAGTCTGTCCGGTAATGATTTCCAGAACCATAACTGGAACTTAGGGTTTGATTATGTGGACGAGTTGATCCGGTTCGCTGAGTGCTTCGAAACCAACGCTGTCCAACTCGCTCACGTGATACTGGCACGGCTCAACCATAAACTCAGATCCGCAACCGGAAAGCCGCTCCAGAGGCTGCCTTCTACTTCAAGGAATCTCTCCAGTCTCTACTCACCTGCTTCACGGCTAACCAAGCCGTGCTGGAACAGGCTCTATGCTTGTCCACGTCATCGACTTCGACATCGGGCTCGGCGGCCACTGGGCTTCGTTCATGAAAGAGCTAGCCGAGAAAGCCGAGTCGGCTCGCGCCAACCGGCTCTACGCATAACCGCTCTCGCGATTAAGTTCATGGAGGGAGAAAATCGCGGTGATTTTATCTCCGTCGATATTCAGGCGGGTCGGGGCGGGGTTCGTGAACGATCTCCGCGTCGGATTTCGCCGCACGTGGTGGTGCACGTGGACAACGAAGGGCTGGTAGGTTTTGGCCCGTCATCTTTCCGGCAGACGGTGATAGACGGGCTGGAGTTTTACTCGACGCTGCTGGAGTCTCTGGAGGCGGCGAACATTGGCGGCGGAAGCGGCGGCTGGCGACTGGATGAGGAAGATCGAGACGGTGCAGGTAAGAGGATTCCACGTGGCGAAGAGGCAGGCGGAGATGCTGCTCTGCTGGCACGATAG

>IbGRAS41

ATGAAAGTGTCCTTTACTTCTAATGAAAACGTGAGTTCAAAAACGTCGACGTTGAGTAGCTGTGGGAGTAATATCAGTATCCAGGCCCCACCTTTTCCGGCCGCCGCCACCGCCGCCGCCGGAGTGGTCAGTTACGAGCCGAAATCTGTTCTCGAGCTCCGCCGCAGTCCCAGCCCTAACGTAACAGAAAACGCGCATAATCCGGCACTAGATGCGGCTGATATCTCGGCCGGTTGCGACGACCCTCTCCAGCTGGCAGATCATGTGCTAACCAACTTTGAAGATTGGGACTCTTTGATGAAAGACTTAGGTCTGAAGGAAGATACAACAAAACCTAATTCCGAGTCGCTCCAGACTCAGTTCCCCGAGTTCCCGCTCGCTCACTCGCTCGATTCCGCTCAGTTCCTCCCGTCTGAGCATTTCAGCTTCTCCGATAACGTCACGGCGGCGCAGTACCCGCCTCCGCCGCTCAGCGCCGGGGGGAATAGTAATTTCAGTCTGTCCGGTAATGATTTCCAGAACCATAACTGGAACTTAGGGTTTGATTATGTGGACGAGTTGATCCGGTTCGCTGAGTGCTTCGAAACCAACGCTGTCCAACTCGCTCACGTGATACTGGCACGGCTCAACCATAAACTCAGATCCGCAACCGGAAAGCCGCTCCAGAGAGCTGCCTTCTACTTCAAGGAATCTCTCCAGTCTCTACTCACCTGGTCAACTCGGATGACTCGCCCCAACTCTTCCTCCGAGATTGTGCACACCATCAAAGCCTACAAGATCTTCTCCAACATCTCCCCTATCCCTATGTTCTCCAGCTTCACGGCTAACCAAGCCGTGCTGGAGGCCGTCGAGGGCTCTATGCTTGTCCACGTCATCGACTTCGACATCGGGCTCGGCGGCCACTGGGCTTCGTTCATGAAAGAGCTAGCCGAGAAAGCCGAGTCGGCTCGCGCCAAGCCGGCTCTACGCATAACCGCTCTCGTCCCGGATGAATACGCCGTGGAGTCGAGGCTGATCAGAGAGAATCTGACTCAGTTCGCGCGCGATCTAAACATGGCTTTCGATATAGATTTTGTGTTGATTCACACGTTTGAGCTGTTATCTTTCAAGGCGATTAAGTTCATGGAGGGGGAGAAAATCGCGGTGATTTTATCTCCGTCGATATTCAGGCGGGTCGGGGCGGGGTTCGTGAACGATCTCCGCCGGATTTCGCCGCACGTGGTGGTGCACGTGGACAACGAAGGGCTGGTAGGTTTTGGCCCGTCATCTTTCCGGCAGACGGTGATAGACGGGCTGGAGTTTTACTCGACGCTGCTGGAGTCTCTGGAGGCGGCGAACATTGGCGGCGGAAGCGGCGGCGGCGACTGGATGAGGAAGATCGAGACGTACGTTTTGTATCCGAAGATAATGGAAATGGTGGGGGCGGCGGGCCGCCGCGGATCGTCGTGGAGGGAGGCGTTTATTGCGGCGGGTTTCAGGCCGGTTGTTTTGAGCCAGTTTGCGGACTTCCAGGCCGACTGCTTACTTGGCAGGGTGCAGGTAAGAGGATTCCACGTGGCGAAGAGGCAGGCGGAGATGCTGCTCTGCTGGCACGATAGGGCCCTCGTAGCCACGTCAGCTTGGAGGTGA

>IbGRAS42

ATGAAAGTGTCCTTTACTTCTAATGAAAACGTGAGTTCAAAAACGTCGACGTTGAGTAGCTGTGGGAGTAATATCAGTATCCAGGCCCCACCTTTTCCGGCCGCCGCCACCGCCGCCGCCGGAGTGGTCAGTTACGAGCCGAAATCTGTTCTCGAGCTCCGCCGCAGTCCCAGCCCTAACGTAACAGAAAACGCGCATAATCCGGCACTAGATGCGGCTGATATCTCGGCCGGTTGCGACGACCCTCTCCAGCTGGCAGATCATGTGCTAACCAACTTTGAAGATTGGGACTCTTTGATGAAAGACTTAGGTCTGAAGGAAGATACAACAAAACCTAATTCCGAGTCGCTCCAGACTCAGTTCCCCGAGTTCCCGCTCGCTCACTCGCTCGATTCCGCTCAGTTTCTCCCGTCTGAGCATTTCAGCTTCTCCGATAACGTCACGGCGGCGCAGTACCCGCCTCCGCCGCTCAGCGCCGGGGGGAATAGTAATTTCAGTCTGTCCGGTAATGATTTCCAGAACCATAACTGGAACTTAGGGTTTGATTATGTGGACGAGTTGATCCGGTTCGCTGAGTGCTTCGAAACCAACGCGGTCCAACTCGCTCACGTGATACTGGCACGGCTCAACCATAAACTCAGATCCGCAACCGGAAAGCCGCTCCAGAGAGCTGCCTTCTACTTCAAGGAATCTCTCCAGTCTCTACTCACCTGGTCAACTCGGATGACTCGCCCCAACTCTTCCTCCGAGATTGTGCACACCATCAAAGCCTACAAGATCTTCTCCAACATCTCCCCTATCCCTATGTTCTCCAGCTTCACGGCTAACCAAGCCGTGCTGGAGGCCGTCGAGGGCTCTATGCTTGTCCACGTCATCGACTTCGACATCGGGCTCGGCGGCCACTGGGCTTCGTTCATGAAAGAGCTAGCCGAGAAAGCCGAGTCGGCTCGCGCCAAGCCGGCTCTACGCATAACCGCTCTCGTCCCGGATGAATACGCCGTGGAGTCGAGGCTGATCAGAGAGAATCTGACTCAGTTCGCGCGCGATCTAAACATGGCTTTCGATATAGATTTCGTGTTGATTCACACGTTTGAGCTGTTATCTTTCAAGGCGATTAAGTTCATGGAGGGGGAGAAAATCGCGGTGATTTTATCTCCGTCGATATTCAGGCGGGTCGGGGCGGGGTTCGTGAACGATCTGCGTCGGATTTCGCCGCACGTGGTGGTGCACGTGGACAACGAAGGGCTGGTAGGTTTTGGCCCGTCTTCTTTCCGGCAGACGGTGATAGACGGGCTGGAGTTTTACTCGACGCTGCTGGAGTCTCTGGAGGCGGCGAACATTGGCGGCGGAAGCGGCGGCGGCGACTGGATGAGGAAGATCGAGACGTACGTGTTGTATCCGAAGATAATGGAAATGGTGGGGGCGGCGGGCCGCCGCGGATCGTCGTGGAGGGAGGCGTTTATTGCGGCGGGTTTCAGGCCGGTTGTTTTGAGCCAGTTTGCGGACTTCCAGGCCGACTGCTTACTTGGCAGGGTGCAGGTAAGAGGATTCCACGTGGCGAAGAGACAGGCGGAGATGCTGCTCTGCTGGCACGATAGGGCCCTCGTAGCCACGTCAGCTTGGAGGTGA

>IbGRAS43

ATGGCATATATGTGTACGGACAGTGGAAATCTAATGGCGATTGCTCAGCAGGTCATCAAGCAGAAGCAGCAGCAAGAACAACAACAGCAACAGCAGCAGCAGCAGCAACAGATTCTGGGTGTGAACTCTTTTTGCTTGAATCCATGGCAGACGCCTCATCCGGGGTTGAGTGGTGGGCCCAGTTTGGGATATGGGCTCGGCGGGGCGGCGTTTGCGGACCCGTTTCAGGTCGGCGGCGCGGGCGGGGATGGTGCGGAGGCGGGGTTTCAGTTTCCGAGCTTGGAGCAGCACGGCGGAGGGGTGTTTCCGTTTGCTGATTTTGGTGGCGGGGCGGGCGGCGAGTTTGACTCGGATGAGTGGATGGAGAGTTTGATAGGCGGTGGGGATTCCACGGGGAGTTCTAATCTTCACTCTGGATGTGACGCGTGGCAGACTGGCTCGGAGTTTGGTCTCTACGGTTCGGATCCGTTTGCTGGCCCGAGTCGACTCAGTATTGCCTCGTCGGCGCCTTCGAATCTCATTTTCTCTGAAGCTCAGAAGAATAATAATATTAATACTTGCCCTCTACAACCTCAAACGTCGGAGTGGGCCCCTACTTCAACTTCGCCGCCGGCACAGCAAACAAGCCCGACTCACCGAAACGACGTCGTAGCAGCAGCAGCGGGGACCTCCTTCAGTTCGCCGGACAATTTATCGTCGAAACCGTTACTTAAAGCGTTAGTAGACTGCGCTAGATTGGCGGAGTCGGAGCCGGACAATGCTACGAAATCGCTGGTTCGACTGAGGGATTCGGTTTCCCAGGACGGAGATCCGACGGAGCGAGTTGCGTACTATTTCTCCGAAGCGCTCTACAGTCGGCTTTCTCGGCAACCGGCGAAGATTCCGTTTGTAGAAGCGAGCTTGGAAGAGTTCGCGCTGTCTTACAAAGCTTTCTACGACGCTTGCCCGTATTCAAAGTTCGCTCACTTGACCGCTAACCAAGCGATTCTAGAAGCTACCGAGAAAGCTTCGAGGATTCACATCGTTGATTTTGGTATCGTGCAGGGCATTCAATGGGCGGCTCTTTTACAGGCCCTGGCAACCCGACCCGCCGGAAAACCCAAATACATTCGGATTTCCGGCATTCCCGGTCCGTTCGATCCGGTTCTGACCCCTATTCCGGAGCTAAACGAGTCCAGTTTACGGGTCGACCCGGATGATGCTCTCGCCGTCAATTTCATGCTTCAGCTGTACAATTTATTGGACGAGACGACGGCCACCGTGGAAGCCGCACTGAAGCTCGCGAAATCGCTGAATCCCAGCGTTGTAACGTTGGGGGAATACGAATTGAGCCTGAATCGGGTCGGGTTCCTGGAGAGGTTCACCAATGCGCTGAATTACTACTCGTTAGTTTTCGAGTCTCTGGATCCGAATATGCCTCGAGACTCGCCGGAGAGGCTGCAGGTGGAGAGGCTGTTGCTTGGGCGGAGAATCGCCGTGATGGTGGGCCCGGCGGAACAGGAGACCAAGAGAGAGTGTACTGAAGATAAAGAACAGTGGAAAATTCTGATGGAAACCGCAGGTTTTGAGCCGCTACCACTCAGCCATTACGCAATGAGCCAAGCAAAAATTCTTCTCTGGAACTACAGTTACAGTGCATCATATGGTCTGATTGAATCTCCGCCTGGGTTTCTCTCCTTGGCCTGGAACGATGAACCTCTCCTCACTGTTTCTTCATGGCATTGA

>IbGRAS44

ATGAAGAGGGAACGCGATCGACCCAAGGCGGGGAGCTCGTCAATAGGTAAAGCCAAGATTTGGGAAGACCAGCCGCCGGATGCGGGAATGGATGAGCTACTCGCGATGTTGGGGTATAAGGTGAAGACTTCTGATATGGCTGACGTGGCTGAGAAGCTTGAGCAGTTAGAGATGGCCATGACCATGACCATGGGCACTGCAAAGGAAGATGGGATTTCTCATCTTTCCACTTCTACTGTTCACTATAACCCTTCTGATCTCACCGGGTGGATCCAGAGCATGCTCTCCGAGCTCCACACCTCCGACGCCCTTTCCCAGTCTTCGGTCGGCGATGAGATGATGCTCGCCGGCGAATCTTCAAATATTATAAGCTTTTCCGGGAATAAGAACATCGGAAATGGGAATAGGAGGATTTCCGATGATGATTTGAGGGCGATTCCGGCGGGGCTATTTTCGGTAATAAGGGGAAAGATTCCTCATACTCCTCGATCGGTTTTGGTGGATTCTCAAGAAGCCGGGGTTAGGCTTGTCCATGCGCTGATGGCTTGCGCCGAGGCGGTCCAGCAGGAGAATTTCAAGCTAGCCGACGCGCTAGTTAAGCATATCGGCATACTCGCTGTGTCCCAAGCCGGCGCGATGAGGAAAGTGGCGACTTACTTCGCCGAAGCCTTGGCTCGTCGTATTTACAAAATCTACCCTCAAGACACCCTGGAATCCTCCTACACCGATGTTCTCCAGATGCACTTTTACGAGACTTGCCCCTACCTCAAATTCGCGCACTTCACGGCTAACCAAGCCATTCTCGAGGCGTTCAATGATTGCAGCCGAGTCCACGTCATCGACTTCAGCTTGAAGCAAGGGATGCAGTGGCCGGCGCTTATGCAGGCGCTGGCTCTCAGGCCCGGCGGCCCACCGGCTTTTCGGCTCACTGGGATTGGCCCTCCCCAGCCGGATAACTCCGACGCTTTGCAGGAAGTTGGGTGGAAGCTGGCTCAGCTCGCGGAAACTATCGGCGTGGAGTTTGAGTTCCGCGGCTTCGTGGCTAACTCGTTAGCCGATTTGGATGCCTCAATCTTGGATATTAGGCCCAGCCACGTGGAGGCCGTGGCCGTTAACTCCGTTTTCGAGCTTCATAGGTTGCTGAGCCGGACCGGCGGGATAGAAAAGGTGTTGAATTCGATCAAAAGTATGAAGCCTAAGATTGTGACTATTGTGGAACAAGAAGCGAACCACAACGGCGTCGTTTTTCTGGACCGGTTCAATGAAGCTTTGCATTACTATTCCACCATGTTTGACTCGCTGGAGAGCTCCGGGTTAACTCAGCCCAACAGCCAGGACCTGGTGATGTCGGAGCTCTACCTAGGTCGGCAGATCTGCAACGTGGTGGCGTGCGAGGGGCCGGACCGGATCGAGCGCCACGAGACGCTGAGCCAGTGGAGAGCCAGGATGAAATCGGCCGGGTTCGACCCGGTTCACCTCGGTTCAAACGCGTACAAGCAGGCGAGTATGCTCCTGGCGCTTTTCGCCGGCGGAGACGGCTACGCGGTGGAGGAGAACGACGGGTGTCTCATGCTAGGGTGGCACACCAGGTCGCTCATTGCCACGTCGGCCTGGCAGCTCGGCGGCGGAGGTGGGGGCGAGCCGTAG

>IbGRAS45

ATGGGTGGAGCTGAATCTGAGCTCCTCCAGCCGGTTCCAGTGTTGTTGGAGATCAGCGGCGGCTCATCCGCCGCGGCGCCGGAGGAGCAGGAGCAAGAGCCGCTGCTCCGTTGGATAACGGGTGATGCAGATGACCCATCCATGGCTAATTTGAGCAAGCTGTTGCAGGGTGGCGACCAGGCAGAGTATGAATTCAACGCCAGTTTGGGGTTACAAGATCATAACTTTGGAGCTGACCCAGATTCCTCCTCCTCCGGCAGTGCTTTCTTGCCCACTTCAAGTTCTCCGGCTAACCTGCAAACCCCATCTTTTCTTTCGTTTCTTGATAGCTCAGATATGAAGCCACAACACTTGCAGAGCCCTTCATTTTTCATGCCACTGCCATATTATCAGAGTCAGGATCAGAGCATACAGCCGGCGAAGAGACACAACCCCGGAACTCTCGGAGTCCCCGAGACAATCGACCATCTATTCAAAACCGCAGAGCTGATCCAGGCAGGGAATTCAGTACTCTCCCAGGAGATATTGGCGCGGCTCAATCATCATCTCTCTCCTACCGGCAAGCCTTTCCACAGAGCAGCTTTCTACTTTAAAGAAGCCTTGCAATCTCTCCTCCTCCCACATGCCACCAAACCCACTGTTCTATCTTCACCGTTTAGCCTCCTTGTTTTCAAGATTGGTGCCTACAAATCTTTCTCTGAGGTCTCACCAATGCCACAGTTTGCCAATTTCACCACCAACCAAGCCCTGCTTGAGGCCTTGGAAGGGTTTCACAGAATTCACATTGTAGATTTCGATATTGGGTATGGAGAGCAATGGGCTTCACTCATGCAAGAACTTGCCTTGAAGAACAGCAGCAAACCTAGCCTGAAAATTTCAGCTTTAGCATCTCCTCCATTGAAGCAGGAGCAGCTTGAGCTTGGGATTATCAGAGACAATTTGATCCAGTTTGCGGCTGAAATCAATATGGGATTTGAGTTTGAAACTCTGGGTGTTGATCATCTGAATTCGTCGTTGTGGTCAGACTCAGACAGTGAAGCCGCCATTGCTGTCAACCTTCCAGTTGGTTGGCTTTCAAGAAACCAACAAGTATCACTCCCTCTAGTTCTTAGCTTTGTTAAGCAGTTACAGCCTAAAATTGTGGTTTCTGTGGAGAGAGGCTGCGACAGGACTGACCTCCCGTTTCCCGACCATATCATCCACGCCCTTCACTCCTGCTCCAACCTTCTCGAGTCTCTTGACGCTGTAAACATGAATCCGGACGCCCTGCAGAAGATTGAGAGGTTCTTGATCCAACCAGGAATCGAGAAAACCATAACTGCGCGGTTTGATTCTCCTGAGAAAACACAAACAGAGCACTGGAGGACTCTTTTCTTGTCGTCTGGATTCACCCCATTTAGTTTCAGCAATTTCGCCGAATCACAAGCTGAGGGTGTGCTGAAAAGAACTCCAGTTGGGGGATTTCATGTGGAGAAGAGGCAATCTTCTCTGGTTCTTTGCTGGCAGCGAAAGGAGTTGATCTCTGCATCTGCCTGGAGGTGCTGA

>IbGRAS46

ATGAAAGCAGACCTAAGAGGGAACCCAACATCCTTTCTTTTCCAAAACCCTAACCTTTTCGTCGCCAGCTCTCAACCCTCCTCTGATCTCACAGGCGCACTCAGAGGATGCAGCAGTCTTGGCAGCCTTGACGGAGCGTGCACGGAGAAGCTGTTACTTCACTGCGCAAGTGCACTGGAGGCCAATGACGTCACCCTGGCTCAGCAGGTGATGTGGGTGCTCAATAACGTCGCCTCACCCACGGGTGACCCTAACCAAAGGCTCACTTCTTGGTTCTTGAGGGCCTTGGTTTCCAGGGCGTCTAGGGTTTGCCCTAACGCTCCGGTCACCGGTTTCGGGGGCGGCAATGGCGTTCACACTAGGCTGATGACTGTGACTGAGCTTGCAGGGTATGTGGATGTGATTCCTTGGCATAGATTTGGGTTTTGTGCGGCGAATAGTGAGATTTTAAGGGCGGTTCAAGGGCAAAATAGGGTTCATATATTGGATTTTAGTATTACTCATTGTATGCAATGGCCTACTCTTATAGACGCCTTGGCTAAGCGCCCTGAAGGCCCGCCTTTTTTAAGAATATCAGTCATGTCTGGGCGGCCGTCTGTCCCGCCCTTGCTTAATGCTTCATGTGAACAGCTCGGTGTTCGTCTCGCGAACTTTGCAAAGTTCAGAGACGTGCCATTTGAGTTCAATGTGATCGGATCAGAATCAGACTATATCCACCAAGAATTCCCGGATTTTCACCACGATTTCATCCTATCTTGTCTCACCCCTTCCTGTCTGAATCTCAGGCCCGACGAGGTTTTAGCAGTGAACTGCCAGAACTGGCTGCGGTATCTCCCCTCTCGCGACACTTTCATCGACGCAATCAAAGGTTTAAACCCCAGCATCGTCACCATAGTCGACGAGGATTGCGATCTGGGCGGTTCCAGTTTAGTTTCGAGAATCACGACATGTTTTAACTACCTATGGATCCCCTTTGACGCCCTGGAAACGTTCCTGCCGAAAGACAGCCCGCAGAGAATCGAGTACGAGGCGGACATCGGACACAAAATCGAGAACATCATCGGATTCGAAGGGGATCAGAGGATAGAACGGTTAGAATCCGGGATGAAATTATCGCAGAGGATGAAGAACAACGGGTTTCTCAGCGCCCCGTTTTGCGAGGAAACAATTAGAGAAGTTAAGTGTTTGTTAGATGAACATGCTAGCGGTTGGGGAATGAAGGTAGAGGACGAGATGTTGGTTTTGACATGGAAAGGGCACAGTGCAGTTTATGCGACGACTTGGGTCGTCTCACCTCAAACTCAAGTCGACATTGAAATCAAAGATTAG

>IbGRAS47

ATGCAAGCGTCTCAACGTCTCAGGACATCAAGCATGTCGAATACAATGTACTATCAGCCTGTTCAAAAGGCGGAGCCCTACTGTCTGCCTCAATTTCAGAATTTAGACCACCAGCCGAGCTACACTAACAGCATAAATGGAGGTAATCACGCCATTCATCCTCATTGTGCCAGTTATTACACCGAATTTTCCTTGATGAGTAGGGCTCACGGTCTTCACAACTCATCGTCAACTCTCAGTTTCTCGCCAAATGGGAATTCTGTTTCCCAACAAGAATCTCAGGCGTACCCATCAGGCCTGCATCAATCCCCTGCCACTACTTACAGCTACCCCATTCGTGAATCGTGCTTTGGGGACGAGGTGAATGATCTTGATTTCAAAGACAAGTTAAGGGAATTAGAAACCGTAATGCTTGGACCTGGTTCGGATATTGTGGAGAGTTATGCAAATGCCATAGTGTCGCCAGAAATAGACAGCTGGGAGCAAATGATTGTGGCCATCCCCCGAGGGGACTTGAAACAGCTCCTCGTTGCCTGTGCGAAAGCAGTGTCGGATAATGATTTGCTGACAGCACAATCGTTAATGTCTGAGCTATGGCAAATGGTATCAGTGTCGGGGGAGCCAATTCAGCGACTAGGAGCATACATGTTGGAAGGGCTAGTAGCGCGTTTGCCTGTTTCAGAAACCTCGATTTACAAATCTCCGAGATGCAAGAAACCATCGAGTTTCGAGCTGCTTTCTTCTATGCATATCCTGAACGAGGTCTGCCCTTACTTCAAATTCGGGTACATGTCAGCTAATGGCGCGATTGCAGAAGCAATGAAAGACGAAAAAAGAGTTCACATAATTGATTTCCAAATCGGGCAAGGCAGCCAGTGGGTGACTCTAATCCAAGCTTTTGCTGCTAGGCCAGGGGGGCCACCCGAGATTCGAATAACAGGAATAGACGATTCTACATCAGCTTATGCGCGCAGGGGAGGCCTAAGCATCGTGGGGAAGAATCTATCCAATCTCGCGGAGTCATTCAAAGTACCATTTCAGTTCCACGCTGTAACCATGCCAGGCTGCAAAGCTCAACTCGAAAACCTGACAATTCAACGCGGAGAAGCACTGGCAGTTAACTTCGCCTTCATGCTACACCGTATGCCAGATGAAAGCGTGAGCACGAGCACTGATAATCACCGGGACCAGCTACTTAGGCTCGTTAAAAGCCTGAACCCCAAGGTAGTCACCCTCGTCGAGCATGAATCCCAATCAAACAGTCCTGCTTTCTACCCCCGGTTCCTTGAAGCCTTGGATTATTACAGCGCGATGTTTGAGTCTCTGGATATGACCCTCCCAAGGAACCACAAACAGCGCATCAACGTGGAGCAGCATTGCTTGGGGAGAGACGTTGTCAACATAATAGCCTGCGAGGGAACCGAGAGGGTGAAGAGGCATGAACTTCTTGATAAGTGGAAATCGCGGTTTAGAATGGCGGGTTTTAGTCCATACCCTTTGAGTTCTTTGGTGAATGGTACAATCAAAACATTGTTGGAAAACTACTCTGATAAATATAGGCTGGAAGAAAGAGATGGAGCCCTTTATCTTGGCTGGAGGAATAGAGATTTGGTTGCTTCATGTGCTTGGAAGTGA

>IbGRAS48

ATGTCTCCTGGTTTGATCAGTGACGAATTGGCGTATAGCTCTAATCCTTACGCCACTATCCTGAAGAGAAATGCGGATAGCTGTATTCCTGTTTTTTGTGACGACGAAATGCTGGAGAGTAAGAGGCCGAAGAGAGCACCGAGTATAGGTGACTGTCTTGGGAGCAACGAAATCCGGAATAGCTTATCCCGGGTTCACTTCAGAGATCACGTTTTGGCTTACAGCCAGAGGTTCCTAGCCGCCGAAGCCGTGGAGCTGCAGGCTGCGGACGAGATGATCGGCTCGGATAACGGCGAGAGTGCAGACGGGATGAAGCTGGTTCAGCTCCTCATTTCCTGCGCCGAAGCCGTGGCTTGCAGGGACAAGTCACGCGCTTCGGTTTTGCTGTCGGAGCTACGCTCCAGCGCGTTGGTTTTCGGGACTTCGTTCCAGCGCGTGGCGTCCTGTTTCGTGCAGGGGCTAGCGGACCGGCTCGCCCTGGTTCAGCCGCTCGGGACGGTCGGGTATCTGACTCCGGCGGCGGCTTTGGATGCCGCCGCCGGCGACGCCGCGTCGGAGAAAGACGAAGCGCTCCGGCTGGTTTACGAGATTTGTCCGCACATTCAATTCGGTCATTATATAGCCAACGCTTCTATTGTAGAAGCCCTTGAGGGAGAGAGTTTCATTCATGTGGTGGATTTGGGGCTAAGCTTGGGCCTTCCTCATGGACATCAATGGCGGCACCTAATCCAACGCCTCGCCTCCTCCAATCGCCCCTTGCGCCGCCTGAGGATCACGGCGGTCGGAACATTACTCCACCGCTTCCGATCGATCGGAGAAGAGCTCAAGGACTACGCATCCGGCCACGGAATAAACCTAGAATTCTCAATCGTCGAGAGCAATTTGGAAACCCTAAAAGCATCGGACATCAAAATCGTCGACGGCGAGGTCCTCGCCGTGAACAGCGTCCTCCAGCTCCACCGCGTCGTGAAAGAGAGCCGCGGCGCTCTCAACTCCGTCCTCCAAATCATCCACGAGCTCTCGCCGAAGATCCTGGTGGAGCAGGATTCCGGCCACAACGGCCCCTTCTTTCTCGGCAGATTCATGGAGGCTCTGCACTACTATTCCGCCATTTTCGATTCCCTGGACGCGATGCCCAAAATACGACACGAGGAGAGCGAAGATCGAGCAGTTCTACTTCGCGGAGGAGATTAA

>IbGRAS49

ATGGCTTCCAAGCGCTCTGTGATTGACCTCGGCGGCGCAGCTGCGGCGGAGACGGCGGCGGCGGCGGTGGAGGATTCTTCGTTTAAGAGGCCTCGTCATTTGTCTTCCTCCGGCGATCCTTCTGTTTCCGGCGAGAAGGGGGAGGAGGAGGAGGAGGAGGGGGTTATGGATACTGAGTCAACGGGGCTTAGGTTACTGGGCCTGCTGCTACAATGCGCCGAGTGTGTGGCTATGGATAACCTCGATGACGCTAGCAATCTGTTGCCGGAGATCGCGGAGCTCTCGTCGCCGTTTGGCTCGTCGGCGGAGCGCGTCGCGGCTTATTTCGCCGAGGCGCTCTCGGCGCGGATTATTAGCTCTTACCTCGGCTCCTACTCGCCTCTCACCCTCAAAACCCTAACCCTAACCCACTCGCAGAAGCTCTTTGCCGCTTTACAGAGCTATAACTCCATCAGTCCCCTTGTGAAATTCTCGCACTTCACCGCGAATCAGGCGATCTTCCAGGCGCTGGACGGCGAGGATCACGTCCACGTCATCGACTTAGATATCATGCAGGGCCTCCAGTGGCCCGGATTGTTCCACATCCTCGCCTCCCGCAGCCGCAAGATCCGGTCTATCAAGATCACCGGCGTCGGATCCTCCATCGAGCTCCTGGAATCCACCGGCCGGCGACTCGCCGAGTTCGCGACCTCGCTCGGCCTCCCCTTCGAGTTCCGCCCGCTGGAGGGGAAAATCGGGAGCATAACCGACCCGAGTCAACTCGGAGTGAAACTCGGCGAGACCACGGTGGTGCACTGGATGCACCACTGCCTTTACGACGTCACCGGCAGCGATTTCGGGACGCTGAGACTCTTGACTCTGCTCCGCCCCAAGCTAATCACCATCGTCGAGCAGGATCTGAGCCACGGCGGCAGTTTCCTCGGCCGATTCGTGGAGGCGCTGCACTATTACTCGGCGCTGTTCGACGCGCTGGGGGACGGATTGGGTGCGGACAGCGTAGAGAGGCATACGGTGGAGCAGCAGCTCTTCGGCTGCGAGATTCGGAACATCGTGGCCGTTGGTGGGCCCAAGAGGACCGGCGAGGTCAAGGTGGAGAGATGGGGAGAGGAGCTCAAACGGGTCGGGTTCGGACCCGTTTCTCTGGCGGGTAGCCCGGCGGCCCAAGCGGGTCTTTTGCTTGGGATGTTCCCTTGGAAAGGGTATACTTTGGTGGAAGAGAGTGGGTGCTTGAAGTTGGGGTGGAAGGATTTGTCTTTGTTGACCGCCTCCGCCTGGCAGCCGTCCGATTAG

>IbGRAS50

ATGGGGCAAGTGGTACGAGGAAACCCAACTTTTCAAAACCTCGCAACCTTCCTGAACAATAATCCTCAGACCTCTCTCTCCTCCGCCGGCGCGATCGGAGGGTGCCTGCTCGGGAGCCTCGACGGCGCGTGCACGGAGCGGCTCCTGCTCCACTGCGCCAGCGCGCTGGAGAGCAATGACGTCACGCTGGCCCAGCAGGTGATGTGGGTCCTCAACAACGTCGCCTCGTCCACCGGCGACGCCAACCAGCGGCTGACGTCATGGTTCTTGAGAGCCCTCGTGTCACGTGCCGCTAGGGTTTACCCCAAGAGCTACCCCACGTGCGGCGGCGGAAATGGCGCGTGCGGGAGGGTGATGTCGGTGACGGAGCTCGCCGGCTACGTGGATCTTATTCCGTGGTATCGGTTCGGGTTTTGCGCGGCGAATAGTGCGATTATTGAGGCGGTTCAAGGGTGCGCGAAAGTTCATATTTTGGACTTTAGTGTTACTCATTGCATGCAATGGCCGACTCTCATTGATGCCTTGGCGGATAGGCCGGAGGGGCCGCCGGCTCTCCGGCTATCGGTCCCTTCATGGCGGCCGCCGGTGGCTCCGTTGCTTAGCCTCTCCACTGAGGAAGTCGGGCTGCGTTTGGCCAACTTCGCTAAATCCAGATCCATCCCTTTCCAGTTTAGTGTCATTGCAGATATCAACAATGGCGGCCTTGGCTTATTTGAGGACGCCGACGAGGCTCTGGTGGTGAACTGCCAAAACTGGCTGCGATATCTGCCGTGCCGCCAAACGTTTCTGGACACCATCAAGTGCCTAAACCCGACACTCATAACCGTCATCGACGAGGACGCCGATTTGGACGCGCCAAGCCTGTCGTCAAGAATCGTCAACTGCTTCAACTACCTGTGGATAATCTTCGATTCCTTGGAGACATTCCTGTCCAAGGACAGCCAGCAAAGGGCGGAATACGAGGCGGATGTGGGGCAGAAAATCGAGAACATTATTGGGTTCGAAGGGGGGCAAAGGATAGAGCGGTTAGAGTCCAGTACGACGTTGTCGACCAGGATGAGGAACAGTGGGTTTTTGAATGTTCCCTTCTCCGAGGAGACAATTAAGGAAGTGAAGTTTGTGTTGGATGAACATGCGAGCGGTTGGGGGGATGAAGAAGAAAGATGA

>IbGRAS51

ATGGCGGAGCTGTGGGGTTTTAGAGAGGGACTCATCCTTGCCAAGGATCAAGGCTTCGAGAAAGTTGCGGTTGAGACAGATTCTGAGACTATGATCCATGACATTAAGATCCTTCATGTCCTCCGAGAAGGGAATCAATGCGCCGACTTCCTTGCCAACCTAGGCCAAGAGTCACCTTGGGGTACTACCGTGCTCGATCGTCCGCCTGATAACCAGCAGAGAACTGAGCAGAAGGGTACACAGCACAGAACAGTAATACTATTGAAACACAAAAAGTGGTGTAGCCTAGCAACTAGCAAGCTAGGCAACCCAGAACTGGTGAAGGGTGAGGTATGGTGTAGAGAGAATGGAGCTCCTGCAATGGTGATGATAGGAATGCAGTATAATAGCAATAGCTTGCAGGGGAAAAGTGTTCTTGAAGTTTCTCGGTTTGGGTCTGCAATTTCTCCCTCCTTGGTGCCCTCAGATGCGGCCAAGCTCAAGAAAAATGGCGGCTTTAGCAGCAGTGAACCGGTTTCTGTTCTTGATACAAGGAGCCCTAGCCCCTCCACTTCCACCTCCGCTATGGAGGCTAATCCTAATCCAGTCGGCCGGAAAGAAAAGAGCTTGGGAGAGCTTCACCCGGCGCCGGAGTGGAGTGAGGGGCCGGAAAAATTTGATCTTGGAATGGAAGATTGGGAGGGTTTGTTGCCGGAATCCGGCGGGTCGGACCAGTTTCTTCTCCGGTGGATCTCCGGCGACGTGGAGGACCCATCTTTAAGCTTGAAGCAGCTGCTGCAAGGAGGGAACCCTAGTGAGATCCATTGCAATGTGGGGTTTGGGGCTTCTGATTCTGATAACTACAATGGTAACTCTCAAGGCCCAAATGTTAATTTCCCACTGGCAATTTTAGGGTCTGGTTTGAGTGTTCAAGAATTGCAAAATGAACAAAAGCCCCAAAATTTCAATTCCCCAGTACTGACTGAGCTTCAGAATGTTGTGACAATACCTAATGTGTTTAGCTCTGAACTGTATGGGATTCAGCAAGAGCAGCCCCCGTTAAAGCGGCAGAATTTGGGTGGTGTTTCGAGCTCGGGTTTTAGGGTTTCAGAAGGAGGTGTGTTTGTGAATCCGATTAATGGGTTGTTGGTTAGGAAACAGGTGGAATTGGGGCAATTGGAGCAGGCACAGATTGGTTGTGATCGGCCACCGGATATATTGTGTGTTCATCGGAGCGAGCAGGCGCCTTTGCTCGTGCCCACGCGAGAGGCGGGATTTGGGAATGATAGCCTGCAGGAACAGCAGGCTGTGTATGATCATGTTTTTAAGGCGGCAGAGGTAATTCTGACCGGGGATTTCTTACACGCTGAAATGATATTGGCTCGGCTCAATCACCAGCTCTCTCCCGGGGTTAAACCCTTGACGAGGGCTGCTTTTTACTTCAAAGAGGCTCTGCATTTGCCTCTTACTATGCCCATCTCCGCTACCTCTCTGCCCCCGAGAATTCCTACACCCGTTGATGGCGTGTTCAAGATGGGGGCTTATAAGGTCTTTTCTGAGGTCTCACCGCTTATCCAATTCATGAATTTCACCTCCAATCAGGCAATTCTCGAGGCCGTTGATGGTGCAGAGTGCATTCACATCTTTGACTTCGATATTGCCTTTGGTGCCCAATGGTCATCTTTCATGCAAGAGCTTCCTAGGAGGAATAGAAGAGCGCCTTCGCTGAAAATAACCGCCTTCGCTTCTCCTTCGACCCACCATCCCATTGAAATCAACCTTATGCACGAGAATTTGACCCAATTTGCTAATGAAGTCGGTGTTAAATTCGAGCTTGAAGTGGTGAACTTCGATTCTTTCGATCCGAGTTCTTATCCGGTATCTTCTTTCCGATCGTCCGAGAGTGAGCCCAAAGTCGTGGTTTCTCTCGACCGTGGATATGAACGAACCGAGCTCCCGTTCCCGCACCACCTCCTCAACGCCCTTCAGTACTTCGAGGCTCTTTTCAAGAGCATCGACGCTGGCAATGTGACACCTGAAGCATCAAACAAGATCGAAAGGTTCCTCTTCCAGCCTAGCATTGAGAGCATCGTGTTTGGGCACCTCCGTTTCCCCGACCAAATGCCTCCGTGGAGAAACCTCTTCGCATCTGCCGGGTTCCTACCACAGCCATTTAGTAACTTCGCCGAAACCCAAGCCGAATGTGTCGTGAAAAGAACCGAAACTCGGGGATTCCACGTGCAGAAGCACCACGCATCTCTCGCCCTATGCTGGCAAAATCGGGAGCTACTCTCGGCTACGGCTTGGAGGTGCTGA

>IbGRAS52

ATGCAAAGCAACGTTAGCCGGTCGCCACCGGCGGCGGAGGAGCCCTCCGCCGCCGCTAGCGACGGCGGGAAGTGGGCGGAGCGTCTCCTGAGGGAATGCGCGGGCGCGATTTCCGAGAAAGATTCCGCCAAGATCCACCAGCTGCTGTGGATGTTAAACGAGCTAGCGTCGCCGTACGGGGACTGCGACCAAAAACTCGCCGCGCATTTTCTTCAAGCCTTGTTTTGCAAGGCAACGGAGACGGGTCCCAAGTGCTACAAAACCCTCCTCTCGGTCTCCGAGAAGAGCCACAGCTTTGATTCCGCTCGGAAGCTCATCCTCAAGTTCCAAGAGGTGAGCCCCTGGACAACTTTCGGTCACGTCGCCTCCAACGGCGCCATCTTGGAGGCCTTGGACGGGGAAACCAAGCTTCATATTATCGACATTAGCAACACGTTTTGTACCCAATGGCCTACTTTACTCGAAGCCCTGGCCACGCGGAACGACGAGACGCCCCACCTAAAGCTCACGGTGGTGGTCACGGCCGCGACGGTGGTGAAGTCGTTCATGAAGGAAATCGCGCAGAGGATGGAGAAATTCGCGAGGTTGATGGGGGTACCGTTCGAGTTCAACGTCGTGAGCGGGCTAACCCATCTCGGAGAAATCACCAAAGACGCTTTAAACGTCCGAGACGACGAATCCGTCGCAATAAATTGCATCGGCGCCCTCCGACGAGTAACCGTGGACGAAAGGTCCGCGATTCTCCGCACCTTCCTAACCCTCCGCCCCAAAGTCGTCACGGTCGTGGAAGAACACGCGGATTTCACCCACACCCGACACGATTTCGTCAAATGCTTCGAGGAGTGCCTCAGATTCTACACGCTCTACTTGGAGATGCTGGCCAAGAGCTTCCCAGCAACCAGCAACGAGCGGCTGATGCTTGAGCGAGAGTGCTCGAGGAGCATACTTAGGGTTTTGGGCTGCGACGATCCGAGCAGTAACGACGGCGACTCCGAGAGCAGAGAGAGAGGAACCCAGTGGTCCGAGAAGCTCCGAGACGCCGGCTTCTCGCCTTTTACCCTCAACGACGACGCAGTTGACGACGTTAAAGCCTTGCTGAAAAGATACAAATCCGGATGGGCACTTCAGCCACCACAACCACAAGCCGGAGAAGACAACACAACCGGAACCTCATCATCATCAACATCATCAGGAATATATTTGACATGGAAAGATGAGCCAGTAGTATGGGCTTCAGCTTGGAAGCCCTAG

>IbGRAS53

ATGAGTGTTGAAACAAGAAAGAACAGAGATAACCAACCTCTTGAAGATGATTCTGTTGTTGTTGCTGAATCAAAGAGGGAACAGCCTTCAGAATCATCGTTGCAAATTCTTCAGAGATATTGGAGTGATTTGATGCATGGCGGAGGGAAGAAGCTGAATACGATACGCATTGATAAGCCGAGCAACTCCTCTGCTGCTGTGCAGATGGTTCTATCACCGGAAGAGATCATGCAGATAGCTAGGTCAAAATTAGAGCAGTGTGCCTTCCAGAGCTGCTGTGTTCATTCTGTTGTTTTTGAACATTCTTATCTGTTGGGTGAAACTGACATTAAAGGAGGAGATGTGGAGCTTGCTCTGCTACTTCAAGCTTCTGCAGAAATGGTTGCCAATCAACAATTTGATCGCGCGAGGAAGTTGCTGGGCCTCTGTAATCAGTCTGCTTCTGCTAATGGTAGCACAGTTGAAAGAATTGTTTATTATTTCGCTAGGGCTCTTAAAGAGAGGATGGATCTGGATAGAGATACTGAAACAGAAGAAAGTGAAAAGGTTCCATTGAATGTTGAAGAGGCTGTGATGAGTATGGAGGCTGCGATAATTGCTTGCGTGCAAGATCTCCCCTTCTCCCAGGTCACCAATTTCACAGGCGTCCATGCGATTCTGGACAACATCACATCCGCGAGGAAGGTTCATTTGGTTGATTTCGAAATTGGAAGCGGATCGCATTGGACGATCATCATGCAAGATCTTGCTAACAGATCTGAACCTCCGATTGAGTCGCTAAAGATAACCGCCGTCGGATCCTCGAAGCGGAGGATTGAAAGGACCGGTAAGTGGTTGTCCTCCTTTGCAGAGACCATGAACTTACCCTTCTCATTCAAAGCAATCGTGTGCGACGCGAAAGATCTGAGGAAAGAGCTTTTCGAGATGGAAGCAGACGAAGTAGTAGCAGTTTTCGCCGAGTATCGTCTTTCAACTCTGCTAGTGTGCCCTAATCAATTACAAAATCTGATTGCAGTTATTCAAACCTTCAATCCATCTGTAATGGTGGTTGCTGAAACTGAAGCCGACACAAACACTCCATCTTTCTTAGCTCGTTTCTACAATCTGCTATCTTACTGCACTGCAACTCTTGATTCTGTCGCTACATGCATGGATCGCGATCATCAATATAGGAAAATAACCGAACAAGTTATACACTGGGAGTTGATTCGAAACGTCATCACAACGGAAGGTGCAGACAGGATCTACCGCCATGCAAAGATTGATTTCTGGAGACAATTTTTCGCGAGATTTGGCATCGAGGAAGAAGCACTAAGCCACTCCGCCTTGTACCAAGCAAGCTTCTTGATTCGGAAATATCCTAGCTGGAGCCATTGCAGTTTGGACATGAATGGAAAGTCCATGATTATAAAGTGGAAAGGAACTCCAGTAAAGTCCTTATCTGTCTGGAAATTCTGCCCAGTTAAAAACAATTAA

>IbGRAS54

ATGTCGGGTTTAAACAAATCTGATGTTTTTGGTGGAGCCCCTGATAGGAACAGATGCATAGATGATGTTGAAAACAATGATGACAACATGAAAGCAGTGAAAAGCAGACCACCTGCAGATGAGGTTGAGGAGTGGGGAGATTCCACTGAAATCGCCTGGTTTTCTTCTTGTCAAGTTTTGGATCAAGATGGCGACGAAAGACCTAGAAAGGAAGCACCTCTCCTGCCCAGGCATAAGCATGAACACCAACTGCAACTTGTTGTAGATTGCATGAGTCTAGATGATCTGGATTTTGATGAGGAGGCTTCATCAGTTCAGCCATTTTCAGGGAATCAAGAGCTCTCTGAATCCAAGAGAAAGAGGGCTGAGAACGCGTTGCCTTCATCCCTGAAGCTTCTCAATAACTTCCAGAACCGGTTCAGGAGATTAACTGGGGAGAAGGTGAATGCAGCAGCAAGCTGCAGCAGTCATGATGAATCCAGAGAGATGAATTGCTGCAGCAGTACTAGGTTGTGGATAAACGAGGTTTTGCAGTTGGCTGCACATAAGTTCATTGAGAACTCTTCCCATGGAGACAGTGAACTAAACAACCTATTTCCCAATTCATGTTGGGGCCTTCATGGTGAAGATTATAAAGATGTTGAACTGCTTCTACATCTCCTAGCTTCGGCTGAGAGAGTTGGCCAGAAAAAGTTTGACAGCGCGAAAGATTTCCTCAGCATGTGCGACAAGCTGAGTTCCAAGAATGGAAATCTTGTGCAGAGATTAGTATACTATTTCTCTGAAGCTCTTCGCGACAAAGTTGATTGGCAGACGGGAGAGAAGACGCCAGAGAATTTTGGAAAGAAACAGATTGAGTATCTCAAAGAGGAACTCACCTGCCCAAGAAAATGCATCCTTGAAACTCATCAGAATTTTCCTTTCTTACAAGTGATCGAGTTCGCCAGTGTCCAAGCAGTGATAGAACATGTAGCAGAAGCAAAAAAGATCCACATTATTGACCTTGAGATCAACCATGGGATGCAATGGACGATTCTGATGCAAGCTCTCGCAGCAGCTTGCCATGCTGAATCTTCCATTGAGAACCTCAAGATATCTGCTCTGGTGATCAAGTCGGGGCCACTGATCGAGGAGACAGGTAAGCAACTAACAAGCTTTGCTGATTCTTTGAAGCTCCCCTTTTCATTTAAAACAGTAAAGGTCCAAGACATTCTTGAAGTGAATGAACAGAGCTTTGAGGTTGAGGAAGATGAAGCACTTGCAGTGTATGGACTGTTTTTCTTCATGACAATGATTTCAAAACAGGACCGGCTGGAACACTTGATGAGAGTGATGAGAACCATCCGTCCACGAGTGATGGTCATAACAGAGGTGGAAGCTAACCACAACTCTCCAGTTTTTGTAAACCGTTTCACCGAGTCCCTCTTCTTCTACAGCGCATTCCTAGATTCCTTGGAGCATTTCCTAAAGCACGATGAATATATCAGAGCGAATTTAGAACGTGAACAACTGAGTCGCCGTATAAGGAACATTGTGGCAGCAGAGGGGGAGGAGAGGGTGATCCGACATGTAAGTATGAATGTATGGAGGGCATTTTTTGCGCGGTTTGGGATGAAGGAGGTGGAGATGAGTATGTCATCAGTGTTGCAAGCCAATTTGGTGTTCAACAATTTTCCCTATGGGAAATATTGCACACTTGACAATGACGGCAAGTCTATGATTATTGGGTGGAAGAGAACACCCCTCTTTTCAGTTTCAGCATGGAAATTGATATGA

>IbGRAS55

ATGTCCTTAGTTAGATCATCCACTCCATTTGGAAACCGTAAATTGTATTTGCTGAATGGTCATGGTGACAGCTCCACTGGCTTGTCTACATCCATGTTCAACCCAGAAAAACATGAGATAGCATATGCAGCTGAATCTTACAGCAGTGGAAGTTATGATGCAAACTATTTCCTAGATTCCCCATCCCCGTCATCTGATCTTGTACACCCATCTGCTTCTGAAGCTTTGGTAAATGCATTCCAGCATTGTCCTCCTCCATGCCCAGTTTCGTCAGGAGAAAATTCCTTAAGCTCTATCCAATCCTTGAGGAAATGTGGTACATATCAAGTTAATTATGATTCAGAGTACATATCTAGCCAAAGTCCTGATCCCCTAGACTTTGAAGAAGGTAACGTGAGATTGAAGCTTCAAGAACTGGAGAGAGCACTTCTTGATGATAATGACGATGATGCCATGTTTGGGTGTACTCAAAGTATGGAAGTTGATGGTCAGTGGGCCGATCCAATTTGTAGTCTGTTTCAAAACGACTCACCCAAGGAATCTTCATCCTCGGAGTCTAATGTTAGTACTAGAAGCAGCAATAAGGTAGATACTCATACACATCAGACTCCAAAGCAGTTGCTTTTTAGTTGTGCTGCTGCAATTCAACACGGGAATTTAGAGCAAGCATCAGCAATGATAAACGAGCTGAGGCAGATGGTATCAATACAGGGAGAGCCTTCTGATAGAATTGCAGCCTACATGGTAGAAGCTCTTGCAGCACGAATGGATACATCTGGGAAAGGTCTTTACAAAGCTCTGAAATGCAAAGCGCCCCCCTCTAATGACCGACTTTCTGCCATGCAAGTCCTGTTTGAGGTGTGTCCGTGCTTTAGGTTTGGGTTTATGGCAGCAAATGGTGCAATTTTGGAGGCATTGATGGGTGAAAAAAATGTCAACATTATTGACTTTGACATCAACCAAGGGAGTCAATACTATACGCTGTTGCAAACACTTGCCAGTATGCCTGGAAAGCCGCCTCATCTTAGGTTAACGGGGATTGATGACCCTGAATCAGTTCAACGTCCTACTGGGGGTTTACGCCTTATTGGGCTGAGGCTTGAGAAACTGGCTGAAGATCTTAAACTTCCATTTGAGTTCAGAGCAATGCCTGCTGAATCTGCATTAGTTGCTCCAACCATGCTGGGTTGCCAACCTGGAGAAACTGTGATTGTGAACTTTGCTTTCCAGCTTCATCACATGCCAGATGAGAGCGTGTCAACAGTAAACCAACGAGACCAGCTTCTACGGATGGTGAAGAGCCTGAACCCGAAGCTTGTAACTGTTGTCGAGCAAGATGTGAACACCAATACTGCCCCATTTCTACCGAGATTTGCAGAAGCCTACAACTACTACTCAGCTGTCTTCGAATCTCTTGATGCAACTCTTCCAAGGGATAGCCAAGAGAGGATGAACGTAGAAAGGCATTGCCTGGCACGCGATATCATCAACATTATTGCTTGCGAGGGAGAGGAGAGAATAGAGCGGTATGAGGTAGCGGGGAAATGGAGAGCGAGGATGATGATGGCTGGATTCAATTCATGCCCTATTAGCCGAAATGTCAATGATTCAATTCGAAAGCTTATAAAGCAATACAGCGAGAGATACAAGGTGAAGGAGGATGCAGGGTCACTGCATTTTGGATGGGAAGACAAAATCTTGATTGTGGCTTCTGCATGGAGATAA

>IbGRAS56

ATGTCCTTAGTTAGATCATCCACTCCATTTGGAAACCGTAAATTGTATTTGCTGAATGGTCATGGCGACAACTCCACTGGCTTGTCTGCATCCATGTTCAACCCAGAAAAGCGTGAGATAGCATATGCAGCTGAATCTTACAGCAGCGGAAGTTATGATGCAAACTACTTCCTAGATTCCCCATCGCCATCATCTGAGCTTGTGCACCCGTCTGCTTCTGAAGCTTTGGTAAATTCATTCCAGCATCCTCCTCCTCCTCCATGCCCAGTTTCGTCAGGCGAAAATTCACTAAGCTCTATCCAATCCTTGAGGAAATGCGATACTTATCAAAGTAATTATGATTCGGAGTACATATCTAGCCAAAGTCCTGATCCCCTGGACTTTGAAGAAGGTAATGTGAGGTTGAAGCTTCAAGAACTGGAGAGAGCACTTCTTGATGATGATAATGATGATGATGCCATGTTTGGGTGTACTCAAAGTATGGAAGTTGATGGTCACAATAAGGTGGATACTCATACACAGCAGACTCCAAAGCAGTTGCTTTTTAGTTGCGCTGCTGCGATTCAACACGGGAATTTAGAGCAAGCATCAGCAATGATAAACAAGCTGAGGCAGATGGTATCAATACAGGGAGAGCCTTGTGATAGAATTGCGGCCTACATGGTAGAAGCTCTTGCAGCACGAATGGATACATCTGGGAAAGGTCTTTACAAAGCTCTGAAATGCAAAGCGCCCCCCTCTAATGACCGACTTTCTGCCATGCAAGTCCTCTTTGAGGTGTGCCCGTGCTTTAGGTTTGGGTTTATGGCAGCAAATGGTGCAATTTTGGAGGCATTGACGGGTGAAAAAAATGTCCACATTATAGACTTTGACATCAACCAAGGGAGTCAATACTATACGCTGTTGCAAACACTTGCCAGTATGCCTGGAAAGCCGCCTCATCTTAGGGTAACGGGGATTGATGACCCTGAATCAGTTCAACGTCCTACTGGGGGTCTACGTCTTATTGGGCTGAGGCTTGAGAAACTGGCTGAAGATCTTAAACTTCCATTTGAGTTCAGAGCTATGCCCGCTCAATCTGAACTAGTTGCCCCAACCATGCTGGGTTGCCAACCTGGAGAAACGGTTATTGTGAACTTTGCTTTCCAGCTTCATCACATGCCAGACGAGAGCGTGTCAACAGTAAACCAACGAGACCAGCTTCTCCGGATGGTGAAGAGCCTGAACCCAAAGCTCAGAACTGTTGTCGAAGCAAGATGTGAACACCAATACTACCCCATTTCTACCGAGATTTGCTGA

>IbGRAS57

ATGGATTATGTTAACTGTGGCTCGCCATTAAGTGGATCTTCTTCTGGTGTTGATGATGGAAATGAACTGAAGCATGTGTTAAGAGGGTTGGAGAATAAATTGTTGGGTCCAGAACCCGAAGATAGTTGCTCCTTGAATGATGATTTAGTAGTAGTAGTATCGAAACCTTCTTTTTCATCGATGGGATTGAAAGAGCTGCTGCTTGCCTGTGCTGAAGCTGTATCAGATGCTGATATATCGACGGCAGAGGCTCTAATGAATCTGTTGGAGCAAAGGGTATCGGTTTCTGGGGATCCTATCCAGCGATTAGGTGCATACATGTTGGAAGGGCTTAGAGCGCGGGTATTAGCATCAGGAAGCATAATCTACAGAAAGTTGAAGTGCAGGGAACCAACTGGCTCAGAGCTCTTGTCTTACATGCAAGTGCTCTATCACATCTGCCCATACTACAAGTTCGCTTACATGTCTGCTAATATCGTGATCCAGGAGGCAATGGTGAACGAGAAAAGCATCCACATCATCGATTTCCAGATTGCACAAGGGAGTCAGTGGGTGTTTTTCATCCAGGCCCTTGCAAATCGTCGTGGTGGATGTGGATCCCCGCCATTCGTCCGTATCACAGGTGTTGATGATTCCCAATCAACTCAGGCTCGGGGCGGTGGGCTTCAGCTGGTTGGTGAAATGCTGGCAAAAGTTGCAGCATCTTGTGGAGTGGGCTTCGAATTTCATGCTGCAGCTATATCTTGGTGTAGTGAGGTCGAAATGGAGGACCTCAACATTCGACACGGAGAAGCCCTGGCTGTTAATTTTCCTTACATGTTACACCACACGCCAGACGAGAGTGTAAGCACCAAGAATCATAGAGACCGGTTGCTAAGACTGGTTAGAAGTTTGTCCCCGAGAGTTGTTACCCTCGTGGAGCAGGAGTCCAACACCAACACTGCAGCCTTCCTCCCAAGGTTCCGCGAAACTTTAGATTACTACACAGCAATGTTCGAGTGCATTGATGCATCAGCTCGGCCTAGGGAAGACAGGCAGCGAGTCAGTGCAGAGGAGCACTGTGTGGCACGGGATATTGTCAACATAATAGCGTGTGAAGGGAGTGATCGAGTGGAGAGACACGAACTTTTTGGCAAGTGGAAATTGCGACTCGTGATGGCTGGATTTAGCCTGTGCCCATTGAGCCCCTCCGTTGGTCTCGCCATGAGGGACATGTTGAAGGAATACAGCCCAAATTACAGGCTAGCAGAAAGTGATGGTGCACTGTATCTTGGATGGAAAAATAGAGCTTTGGTAACTTCTTCTGCCTGGAGATGTTAA

>IbGRAS58

ATGCAAGCATCAGAGGTTTCCCGAACATCAGGCGAGCAATATTATAACCCTTTCCATGTTCTAAATAACTATATTTCCAATGACCACTGCAACTCAGCAACTTGGGATCCCTCCATTCACACCCACAACCAACACTTCTTCACTCTGGACTCATCCCCAACAATGGATTATGTTAACTGTGGCTCGCCATTAAGTGGATCTTCTTCTGGTGTTGATGATGGAAATGAACTGAGGCATGTGTTAAGAGAGTTGGAGAATAAATTGTTGGGTCCAGAACCCGAAGATAGTTGCTCCTTGAATGATGATGATCTAGTAGTAGTAGTATCAAAACCTTCTTTTTCATCGATGGGATTGAAAGAGCTGCTGCTTGCCTGTGCTGAAGCTGTATCAGATGCTGATATATCGACGGCAGAGGCTCTAATGAATCTGTTGGAGCAAAGGGTATCGGTTTCTGGGGACCCTATCCAGCGATTAAGTGCTTACATGTTGGAAGGGCTTAGAGCGCGGGTATTAGCATCAGGAAGCATAATCTACAGAAAGTTGAAGTGCAGGGAACCGACTGGCTCAGAGCTCTTGTCTTACATGCAAGTGCTCTATCACATTTGCCCATACTACAAGTTCGCTTACATGTCTGCCAATATCGTTATCCAGGAGGCAATGGTGAACGAGAAAAGCATCCACATCATCGATTTCCAGATTGCACAAGGGAGTCAGTGGGTGTTTTTCATCCAGGCCCTTGCAAATCGTCGTCGTGGATGTGGATCCCCGCCATTCGTCCGTATCACAGGCGTTGATGATTCCCAATCAGCTCAGGCTCGGGGGCGGTGGGCTTCAGCTGGTTGTGAGGTCGAAATGGAGGACCTCAACATTCGACACGGAGAAGCCCTGGCTGTTAATTTCCCTTACATGTTACACCACATGCCAGACGAGAGTGTAAGCACCAAGAATCATAGAGACCGGTTGCTAAGACTGGTTAGAAGTTTGTCCCCGAGAGTTGTTACCCTCGTGGAGCAGGAGTCCAACACCAACACCGCAGCCTTCCTTCCAAGGTTCCGCGAAACATTAGATTACTACACAGCAATGTTCGAGTGCATTGATGCAGCAGCTCGGCCTAGGGAAGACAGGCAGCGAGTCAGTGCAGAGGAGCATTGTGTGGCACGGGATATTGTCAACATAATAGCGTGTGAAGGGAGTGATCGAGTGGAGAGACACGAGCTTTTTGGCAAGTGGAAATTGCGACTCGTGATGGCTGGATTTACCCTGTGCCCATTGAGCCCCTCCGTTGGTCTCGCCGTGAGGGACATGTTGAAGGAATACAGCCCAAATTACAGGCTAGCAGAAAGTGATGGTGCACTGTATCTTGGATGGAAAAATAGAGCTTTGGTAACTTCTTCTGCCTGGAGATGTTAA

>IbGRAS59

ATGGATCACAAAGCCAATAATCCCTCTAGCTCGAATTATAACTATTTTCAGGGAGTTCTTGACTACATAAACCAAATGCTTATGGAAGACGATGATTTGGAGAATGGGCGTTTTGTATATCCAGACTTGTTGGCTCTCCAGGCTGCTGAAAAATCTCTAGGTGATGTTCTTAACGCTGAAGAATCAAGTGGCAATGATGGCGGCCGGCGATTTCCAGATAGACCAGGGGAGAAGAGACACCGTTGTGGAGAAGACATTAATGGTGTGGAAGCCGGGAGAAGAAGAAGAAGCAACAATAATAATAATGTAGCGAGAGACGAAGAGCAAGTGCAGATAGAGATGTATAATGGCATCAAGGGGCTGTGTTATTGCAGAGAATTGGATGCGTCAGCAAAGCGCGTTGTTCAGCCGAGAGTGAGGGCGAGACGAGGGAGGCCACGCGCCGGCGAGCGGGGAGGCAATGGAAAGGGGGAGGTGGATTTCGCGGGCCTTTTAAACCTGTGTGCACAAGCCGTGGGGGGCGAGGATTTCAGGACGGTGAATGAACTCCTGGGCAGAATCCGACGACATTGCTCCCCTCACGGCGGTGCCGCCGAGAGGCTGTCGCATTATTTCGCTAAAGCCCTGGATGCGCGCTTGGCCGGCACCGGCGCAGCGCTATACACTGCGAACAGAGCATCCCTTGCAGATACACTGAAAGCCTATCAGATGTATTTCACCGCATGCCCTTTCAAGAAACTGTCCAACATGTTCGCCGACAAGTCAATCGGGAAATTGACGATGGAGGCAACCAAGATTCACATAATTGACTTTGGTATATCGCATGGCTTTCAATGGCCGTGTTTGATTCAGGGTCTGTCTCGGCGGCCGGGCGGACCTCCCGCGCTTCGTATCACCGGAATAGATCTTAATGCTGAGGCTACAGCTACAGGGCGTCATCTATCTTACTATTCCAACAAATTCAATGTCCCTTTCCAATACACCGCCGTGACGAAGAAATGGGAAACTATTTGTTCAGAGGATGTTAGGATTGAAAGGGATGAAGTTCTGGTGGTGAACTGTTTATACAGGCTACAAGACGTTCCAGACGAGATAGCATCGGAAGAGAGTCCTAGGGACACGGTCCTTAACTTCATCAAACAACTGAATCCCCAGGTTTTCATGCACGGAGTCGTGAGTGCATCGTACAACGCCCTTGTATTCTCCACCCGGTTTCGCGAGGCGATCTTCCATTTCTCGTCCCTGTTCGACATGTTGGAGGGGACGGTTGGTGGGGGAGATGAAGGGAGAATGGTGTACGAGAGAGAGATTCTTGGACGGGAGATTATGAACGTGGTGGCGTGCGAAGGGGCGGAGAGAATGAAGCGGCCGGAGACGTATAAGCAGTGGCAAGAAAGGATTCAGAGAGCTGGGTTTGAGCAGATGTTGCCGGATGGGGATATCATGAGACAAGTGAGGGCTAAAGTGATGGATTACAGCAATGATTTCTTCGTGGAAGAAGATGGAAAGTGGATGTTGCAGGGGTGGAAGGGTCGAGTTATCTATGCAACCTCGTCTTGGAAGCCTGTCATCAATCACTGA

>IbGRAS60

ATGCCTAGTGTCTCCTTCCCCTCCGGATCCGCTCCTTTCAATCACGATCGTCATCTTCATCCTAAAGGAAGTGTGAATGATGAACTGCGCAGCAGCAAAGATGATAATAATGATTGGTTTGAAATGGAAGGATGTGATGATTTTAACTCCCTCTTTGCTGGAGATAAAAGGATCGAACACCTTATTATTGAAGATCAAAATCAAATTCCTTCCCCAAATCCTCAAGTTTCTGAGCTGAAACCTCAGCAGACTCCATTTTCTTCTGCGTGTTTCGAGATCTTGAAATGTTATGGTAGGTTTTTCAAGGATCGAAGCAGTCTAACCAGCAAAACTGATCGTATCGATGTCAGCGGCGCCGGCGGTAAACTGTCTGTGGCTGAAATTTTGAAGCTCGCCGGAGAAAGGTACATTCAATTCTTCACCCAAAAGGCGGACGGATTTTCCGCGTTCTTCCATCCTTACGCCTCCTCACTCTCCGGAATTTCCGTTGACGACATCAGAGACGTGGAGCTCGTTCACCTTCTTCTGGCCGCCGGAGAGGAAGTGAGCCGCCGGCAATATCATCTCGCTAGCGGCTTTATCTCTCGCTGCCTGTGGATCGCATCCGATTCCGGCACTCCGGTGCAGAGACTGGCGTTCCAATTCTCCGAAGCCCTAACGGAGAGGATAGAGAGAGAAACGGGGAGGTTTAAGGGGGTGAGCGAGGAACGGTTAGCCAGGTGTCGAGAGAGTATGGCGTTGAGTTCGAATCCCGCAATCTTAGCTAGCCACCAAGGGCTTCCCTTCGGTCAAGTGATCCAATTCGCCGGAATCCAGGCCATCATCGAGCGTGTGAAGAACGCAAGGAAGATCCGCTTGCTTGATATCAATATCCGGACCGGAATTCAGTGGACGATTTTGATGCAAGCTCTGGCAGAGCAGGACCGCAGTTCCCCGATCGAGCGTATTAGGTTAACCGCCGTCGGAGTAGTGGAGAGGGAGAAGATGGAAGAGTGCGGCAAGCGATTGCAGAGCTTCGCAGACTCCTTGAATCTCCCGTTTTATTTCGATACAGTCTTCCTCTCAGATCTGAAAGATTTCAGGGAAGATTTAGTCCAGGTAGAAGATGAGGAAGTTGTGGCGGTGATGGCAAACATCGTGCTGAGAACAATGATCGCACGGCCCGATTGCCTCGACACTCTAATGAGAGGAATCAGACGGTTGAGGCCGGCGGTGATGGTGGTGGCGGAAGTGGAAGCGAACCATAACTCGCCGTCCTTCATTAACCGATTCGTGGAGGCGCTTTTCTTCTGGCGTGCCGATTGCTTGGAAGATTGCGGATCGCGACGATCCGTGCGAAGGATCATGGAAGGGCTCCATTTTGGGAAGGGATCGAAACATTGTGGCGGCGGAGGAGAGGAGAGGTTCACTAGAAAGCGTGAAGATTGA

>IbGRAS61

ATGCCTGGTATATCCTTCCCCTCCGCTCCTTTCAATCACGATCATCATCTTCATCGTAAAGGAAGTGTGAATGATGAACTGCGCAGCAGCAAAGATGATAATAATGATTGGTTTGAAATTGAAAAATGTGATGATGATTTTGACTCCCTCTTTGCTGGAGCTAAAAAGATCGAGCACGTTATTGAAGGTCAAAATCAAATTCCTTCCCCGAATCCTCAAATTTCTGAGCCCAAACCTCACCGGACTCCATTTTCTTCTGCGTATTTCGAGATTTTGAAATGTTATGGTAGGTTTTTCAAGGATCGGAGCAGTGTAACCAGCAAAACCGATCATACCGATGCCGGCGGCACCGGCGGTAAACTGTCGGTGGCTGAAATTTTGAAGCTCGCCGGGGAAAGGTACATCCAATTCTTCACCCAAAAGGTCGACGGATTTTCCGCGTTCTCCCATCCTTACGCCTCCGCTCTCTCCGGAATTTCTGTTGACGACGTCAGAGACGTGGAGCTCGTTCACCTTCTCCTGGCCGCCGGAGAGGATGTGAGCCGCCGGCAATATCATCTCGCTACCGGCTTTATAACTCGCTGCCTGTGGACCGCATCCGATTCCGGCACTCCGGTGCAGAGACTCGCGTTCCATTTCGCCGAAGCCCTAACGGAGAGGATAGAGAGGGGAAACGGGAAGGTGATCCAATTTGCCGGAATCCAGGCCATCATCGAGCGTGTGAAGAACGCAAGGAAGATCCACTTGGTTGATATCAATATCCGGAGTGGAATTCAATGGACAATTTTGATGCAAGCTCTAGCAGAGCAGCACGACAGTTCCCCGATCGAGCGTATTAGGTTAACCGCAATCGGAGTGGCGGAGAGGGAGAAGATGGAAGAGTGCGGCAAGCGATTGCAGAGTTTCGCCGACTCCTTGAATCTGCCGTTTTGTTTCCATACAATCTTCCTGTCAGATCTGAAAGATTTCAGGGAAGATTTGGTCCAAATAGAAGATGAGGAAGTTGTGGCGGTGCTAGCAAGCACGGTGCTGAGAACAATGATCGCACGGCCCGATTGCCTCGACAATCTAATGAGAGGAATCCGAGGATTGAGGCCGGCGGTGATGGTGGTGGCGGAAGTGGAAGCGAATCATAACTCGCGTTCGTTCATTAACCGATTCGTGGAGGCGCTTTTCTTCTACGGCGCGTACTTCGATTGCTTGGATGATTGCCTGGATCGCGACGACCCGTGCAGAAGGATCATTGAAGGGCTCCATTTTGGGGAAGGGATCAGAAACGTGGTGGCGACGGAGGGAGATGAGAGGTTCAGTAGAAACGTGAAGATTGAGGTGTGGAGAGCGTACTTTGCGAGGTTTCCAATGGAGGAAATGCAACTCAGCGATTCATCTGCGTATCAGGCTAATCTGGTGGGCAGGAAGTTTGGAAATGGAAATTCCTGCACGCTTGATCGTAGTGGGAAGGGCCTCATTTTTGGGTGGAAGGGAACACCAATGCATTCTGTTACCTGCTGGAAGTTTCCTTAA

>IbGRAS62

ATGAAAAGAGGTAATTTCAATCGTAGTGTTCAGGGTTCTTTTGCCGGCGCCGCAAGCGGCGGCGGCGGCGGTAGTGGGAGTAGCAGCGGGGTGTCGAAGGGGAAGAAGGTGTGGGAGGAGAGTGAGCAGGACGCGGGAATGGATGAGCTTTTGGCGGTTTTAGGGTATAAGGTGAAGTCGTCGGACATGGCGGAGGTGGCTCAGAAGCTGGAGCAGTTAGAGGAAGTGATGGGGAGCGTTCAGGGAGATGACCTCTCGAATTTCGCGTCGGAAACGGTTCATTACAACCCCTCAGATCTCTCCTCCTGGGTGGACTCCATGATTTCCGAGCTCAACCCGGGCGACCCGTTTTTGTCCCAAACCGAATCCTCCGCTATCACCAGTCTCCCCACTCAGATCTTCGACGATTCCTCTTTCGATTCAGATCTCACCGCGATTCCAGGCAAAGCCGCCTATCCTCAACCTCCGAGCAAGCGATTTAGAACCGCTTCCACCACTTCCTCCACCTCCTCCAACATGCAGCTCGGCGGCGCCGCCGCCTGGGGAAGTCCTAGCGAGTCAATCTCTCCTTCCTCCGAGTCAACTCGCTCAGTGGTCCTGGTTGACTCGCAGGAAAACGGCGTCCGACTCGTCCACACCTTGATGGCCTGCGCGGAGGCGATTCAGAAGGAGAACATGAAATTGGCGGAGGCTTTGGTGAAACAAATCGGTTTCCTCGCCGTTTCTCAAGCCGGAGCCATGCGAAAGGTCGCTACCTACTTCGCCGAAGCCCTAGCTCGGAGAATTTACCGTCTCTACCCTTCCAACCACAACGACTCCGCCTTCTCCGACCTCCTCCAAATGCACTTCTACGAGTCCTGTCCCTACCTCAAATTCGCCCATTTCACCGCCAATCAAGCCATCCTCGAAGCCTTCGCTAACAAAAACCGCGTCCACGTCATCGATTTCTCCATGAAACAAGGGATGCAATGGCCGGCTCTCCTCCAGGCTCTCGCCCTACGCCCCGGCGGCCCTCCTAGCTTCCGATTAACCGGAATCGGCCCGCCGTCCAACGATAACACAGATCATTTACAGGAAGTAGGCTGGAAATTAGCTCAACTCGCGGAAACTATCAATGTCGAATTCGAGTTCCGAGGCTTTGTGGCGAACTCCTTAGCGGATCTCGACGCCTCGATGTTCAACATCCAGGACGGGGAGACGGTGGCCGTTAACTCCATCTTCGAGCTCCATCAGCTCCTCGCCCGCCCCGCCGCCATCGAGAAGGTTCTATCCGTTATCAAGGACATGAAGCCGGAGATCGTGACGGTGGTTGAACAGGAAGCGAATCACAACGGTCCGGTTTTCATGGACCGGTTCACCGAGTCTCTACACTACTACTCCACCCTCTTCGACTCGCTCGAGGGCTGCGCCGCCAGCGAAGGCGGCACCGTCTGCGACCAAGATAAGGTGATGTCGGAGGTGTATTTGGGGCGGCAGATCTGCAACGTGGTGGCCTGCGAGGGCGTGGACCGGGTCGAGCGGCACGAGACGTTGGCTCAGTGGCGAACCCGGTTCAAATCGGCCGGATTCTCCCCGGTTCACTTGGGCTCTAACGCCTACAAGCAAGCTAGTATGCTGTTGGCCCTGTTCGCCGGCGGCGATGGGTACAGAGTGGAGGAGAACGACGGCGGGTTGACGCTGGGTTGGCACACGAGGCCACTCATTGCCACCTCGGCCTGGAAACTCACCAGTTAA

>IbGRAS63

ATGTTTCAGGACGAGGGATCATCGTCGTCTATTACGTCATCGTCGCCGCTGCAAGCTTTGCCGGTGGGGGTTTCGGTTTCGCCGGGATCGCCGTACCCGTGGCTGAAGGAGCTGAAATCGGAGGAGAGGGGTTTGTACTTGATCCATCTCTTGCTAACGTGCGCCGGCCACGTGGCCGGCGGCAGCCTGGAGAACTCCAACGTGGCGCTCGACCAGATTTCCCACCTCGCCGCGCCGGACGGCGACACCATGCAGCGCATCGCCTGCTACTTCGCGCAGGCGCTCGCCGACAGGGTGCTCCGAACTCTGCCCGGCGTGTACCGGGCCCTCCATTCCACCAAGCTGGCGTCGCCGGCGGATGAGTTCCTTGCACGCAAGGTGTTTGTGGAAATGTTCCCGTTCTCGAAAGTGGCGTTCCTGGTGGCCAACCAGGCCATAATTGAGGCCATGGAGGGCGAGAAAATGGTGCACATAATTGATTTCCACGCGGCCGACCCGACCCAGTGGCGCGCGCTGCTTCAAGACCTCAGTGCCCGGCCCGAAGGCCCGCCGCACCTCCGGATAACCGGCGTGCACCCTGTGAAAGAGGTGATGGAGCAAATGGGTCGGGTTTTATCCGAGGAGGCAGAGAAATTGGATATCCCTTTCCAGTTCAACGCCATTGTCAGCAAATTCGAAAGCTTGGATTTGGAGAAACTCCGGGTCAAAACAGGGGAAGCTCTGGCAATTTCATCTCCAATGCAGCTCCACACTCTTCTAGCCTACGACGATGACAAGAAACCATCCCCATTTGCATCCAAGATTCCCAACAGAAGGATCCAAATTACCCAAAACTCACTGGGAGATTTCATCGAACAGGATGTGGGCAATGGCTATAGCCCAAGCACCGACTCCGCATCTTCATCCCTATCAAATTCACCCAAAATCGAAGCTTTCCTCAGTGCCCTCTGGGGCTTATCCCCCAAACTAATGGTGGTCACAGAACAAGACAGCAACCACAACGGCCAAACAATCATGGAGAGACTCTCAGAATCCTTATACTTCTACGCAGCATTATTCGACTGCCTCGAATCCACCCTCCCACGATCCTCACTGGAGCGATTGAAGGTCGAGAAAGCGCTTCTAGGCGAAGAAATCAAGAACATTATCGCGTGTGAGGGCTCCGAGAGGAAGGAAAGGCACGAAAAGCTTGAAAAATGGTTCCAGAGACTCGAGTCTGCTGGGTTTGGCAATGTCCCTTTCAGCTACTATGCAATGCTGCAAGCCAGGCGGTTCTTGCAGAGCTTTGGGTGCGATGGGTATAGGATCAAGGAGGAGAATGGGTGCGTTGTGACTTGCTGGCAAGATAGGCCATTGTTCTCTGTGTCAGCTTGGAGATGTAGGAGGTGA

>IbGRAS64

ATGCAAAGCAACGTTAGCCGGTCGCCACCGGCGGCGGAGGAGCCCTCCGCCGCCGCTAGCGACGGCGGGAAGTGGGCGGAGCGGCTCCTGAGGGAATGCGCGGGCGCGATTTCCGAGAAAGATTCCGCCAAGATCCACCAGCTACTGTGGATGTTAAACGAGCTAGCGTCGCCGTACGGGGACTGTGATCAGAAACTCGCCGCGCATTTTCTTCAAGCCTTGTTCTGCAAGGCAACGGAGACGGGTCCCAAGTGCTACAAAACCTCCTCTCGGTCTCCGAGAAGAGCCACAGCTTCGATTCCGCTCGGAAGCTCATCCTCAAGTTCCAAGAGCAACACGTTTTGTACCCAATGGCCTACTTTACTCGAAGCCCTCGCCACGCGGAACGACGAGACGCCCCACCTAAAGCTCACGGTGGTGGTCACGGCCGCGACGGTGGTGAAGTCGTTCATGAAGGAAATAGCGCAGAGGATGGAGAAATTCGCAAGGCTGATGGGGGTACCGTTCGAGTTCAACGTCGTGAGCGGGCTAACCCATCTCGGAGAAATCACCAAAGACGCTTTAAACGTCCGAGACGACGAATCCGTCGCAATAAATTGCATCGGCGCCCTGCGACGTGTCGGGGTCGACGAAAGGTCCGCGATTCTCCGCACCTTCCTAACCCTCCGCCCCAAAGTCGTCACGGTCGTGGAAGAACACGCGGATTTCACCCACACCCGACACGATTTCGTCAAATGCTTCGAGGAGTGCCTCAGATTCTACACGCTCTACTTGGAGATGCTGGCCGAGAGCTTCCCAGCAACCAGCAACGAGCGGCTGATGCTTGAGCGAGAGTGCTCGAGGAGCATACTTAGGGTTTTGGGCTGCGACGATCCGAGCAGTAACGACGGCGACTCCGAGAGCAGAGAGAGAGGAACCCAGTGGTCCGAGAAGCTCCGAGACGCCGGCTTCTCGCCTTTTACCCTCAACGACGACGCAGTTGACGACGTTAAAGCCTTGCTGAAAAGATACAAATCCGGATGGGCACTTCAGCCACCACAACCACAAGCCGGAGAAGACAACACAACCGGAACCTCATCATCATCAACATCATCAGGAATATATTTGACATGGAAAGATGAGCCAGTAGTATGGGCTTCAGCTTGGAAGCCCTAG

>IbGRAS65

ATGCAGCCATCTCAGAACAATATGAACATGGATGGGTCTCGTAGGTTCTATAACCAACCGATGCAGGATCTCGAGTCCTATTGCTTACCTACTGATAACCAGTTACTTGCTGTTGGTGATAACCATAGCGCAGAATTCTTTGCTCAAACTAACAGCCAGTATAACGATGTTATGTCCTATGGTTACCCGGGTCACAGTTATACTGCTGATAGCTTCTTGTCTGATACGAGTGGCATTCTGCAGCAAGATTCGCAGTTATATCTAGCAGACGTGCATCATCATTCTCCCGAGGAGACCTACCACTCGGCAGCTAGCAAGTCTAGCTTATCGGATGAGAATGACTTGAAGCACAAATTAAGAGAGCTGGAATCTGCGATGTTTGGCCATGGTTCGGATGCATTGGAAGCATATGGGAGTAATATGGGGACTCCTGATCAAATGTCATCAGAGGCTGAGAAGTTAGAGGAGATGATGGGGATAACTTCTCGTGGGAGCTTGAAAGAATCACTTGTGGCCTGTGCAAAGGCGATAGGAGATAACGATATTCCAAGAGCCGAATGGTGGATGTCAGAGCTTCGCACGATGGTTTCAGTTTGTGGTGAACCGATACAGAGGTTAGGGGCGTATATGTTGGAGAGTATGGTTGCAAGACTAGCCTCATCGGGAAGCTCCATCTATAAGGCCCTGAGGTGCAAAGAGCCTACGAGCAACGAGCTCTTCTCGTACATGTACTTGCTCTATGAGGTCTGCCCGTTCTTCAAATTTGGATATTTGTCTGCCAATGGAGCAATCGTTGATGCCATGAAAGATGAAGACAGAGTTCATATAATTGATTTCCAAATTTCTCAGGGTACCCAGTGGATTACCCTAATCCATGCCCTTGCAGCTCGACCCGGTGGGCCACCTCAGATCTGCATTACAGGGATTGATGACTCTACATCGGCTCATGCCAGGGGAGGAGGGCTCGAGATTGTGGGTAAGAGGTTGTCTACACTCGCAGAATCATGCAAAGTCCCGTTTGAATTTCGGCCCGTGGCTGCTTCATGTGCTGATGTCAAGATTGAGCATCTAAACGTTCGACCCGGAGAAGCTCTGGCTGTGAACTTTCCACTCGTATTGCACCATATGCCAGATGAGGACGTTGGCACACAAAACCATAGAGAAAGACTGGTGAGGATGGTGAGGAGCTTGTTTCCCAAAGTCGTGACCCTTGTGGAGCAAGAATCAGATACAAACAAAGTCCCGTTCTTTCCCAGATTCTTGGAGACACTAAACTATTACCTAGCTGTATTTGAATCAATAGACGTGGCTCTGCCAAGGGACCATAAGGAACGTATAAATGTTGAGCAACATTGCTTGGCTCGGGAAGTTGTCAATATTCTAGCATGTGAAGGGGTGGAAAGAGTGGAGCGTCACGAGGTTCTCGAGAAATGGAGGTCAAGATTTTCAATGGCTGGTTTTAAACCTTACCCATTAAGCTCTTCTGTTAACGCCACCATCAAGACACTTCTGGAGAACTACTGCAAGGACTATACACTTGAAGAGAGAGATCAGGCTCTTTATCTCGGTTGGATGAACAGAGCCTTGATAGCCTCTTGTGCATGGCAATGA

>IbGRAS66

ATGAGAGCATCACGTCTCAGGAGAACGGGGATGTCCAATACGCTGTGCTTTCAGCAAAAGGCGGAGGCGTACTGTGTGCCTCAGTTTCAGATTTTAGACCAGCTGAGCCACAGTGATAACGGAGGCAACTACTCGATCCAGGCTTATTCTGATCATTGTTGCACTCGGGAATTGTCCTCGAAGAATGGCAGTCATGCTCGTTGCAACTCATCGTCGACCATAAGTTTCTCGCCCTATGGAAGCCCCATGTTGCAGCAACAAGAATTTCAATCGTACCCGTTAGACCTACACCAATCTCCAGAAACCAAGTATAGTTCTCCAATCAGTATGTCTTCTTCCATTACGGATGATATGAGTGATTTCAGGCACAAGTTAAAGGAACTCGAGAGTCTGGAGACAAATGATGGAAAGCATACCGTCGGGGATTTGAAACAGGTGCTCGTTACGTGTGCCAAAGCAATATCAATCAACGATTTACCAAAAGCGGAATGCTTGATGTCTCAGCTAAGACAGATGGTATCTGTGTCGGGCGAGCCAATTCAGAGGCTCGGGGCATACGTGCTCGAAGGGCTCGTTGCACGATTAGATGCATCAGGGAGTTCCATCTACAAATCCCTGAGATGCAAGGAACCCGAGAGCTTCGAGCTGCTTTCTTATATGCACATCCTCTACGAGGTCTGCCCCTACTTCAAATTCGGGTACATGTCAGCTAATGGCGCCATTGCAGAAGCAATGAAGGGCGAAAATCGAGTTCATATCATTGATTTCCAGATAGGTCAAGGCAGCCAATGGGTGACACTAATACAAGCTTTTGCTGCTAGGCCAGGAGGGCCTCCCCATATTCGAATAACCGGGATAGATGACTCCACTTCAGCTTATGCACGAAGGGGAGGACTAAACATAGTGGGCAAGATGCTATCTAAGCTCGCTGAGTCCTTCAACGTGCCATTCGAATTCCACGCTGCATCGATGTCAGACTATGAAGTTCAGGTAGCAGACTTGGGAATTCACACGGGCGAAGCACTGGCTGTGAATTTCGCGTTCATGCTGCACCACACGCCAGACGAGAGCGTGAGCACACAGAACCACCGGGACGAGCTGCTAAGGCTCGTGAAAGGGCTGAATCCTAAGGTGGTTACCCTCGTCGAGCAAGAATGCAATACAAACACTGCACCTTTCTTCCCCCGGTTCCTCGAGACCTTAGACTACTATGTTGCAATGTTCGAGTCAATCGATGTCAATCTTCCCCGGGAGGACAAGCAACGCATAAATGTCGAGCAGCATTGCCTGGCAAGAGATGTTGTCAACATAATAGCGTGCGAGGGAACAGAGAGGGTGGAGAGGCATGAGCTTCTGGGGAAGTGGAAGTCGCGGTTCAGGATGGCCGGGTTTTCGCCATACCCGTTGAGTTCTTTGGTGAATGCCACAATTAAGACTCTGTTGGGAAACTATTCTAGCAAATACAGGCTTGAAGAAAGAGATGGAGCCCTTTATCTTGGTTGGATGAAGAGAGATTTGGTTGCCTCCTGTGCATGGAAGTGA

>IbGRAS67

ATGGCCGCGAAGGCATTTCCTATGGTTGGAGAGGCGGCGAATGTGAGCGGAAGTAGCGGGAGCGCCACCAGTAGTAGGGAGTATCACCACCACGACCACAATAATATCCTTCCGTTGCATTCTTCTTCGGCTTCTCCTTCTCATCTGGCGCTGCTTTGCGATAACGCCAAGATGGTGAGGAAGAGGGCGGCGTCGGAGATGGAGCTGCAGATCGGCGGCGGCGGGGGAATCGGGGAGCACGGCAGGTTTCTCCGGCGGGCGGCGGCGGCGGGGATGAACGCGCCGCTACTTGGGGATTTGAGGGTTTGCGGGATAGCGCCACCGCCCAGCTCCACCAATTTGAGCGTGACGTCAACGTCCGACGCCACGCACTTGACTTACATGGAGACGTTACCGTTACCGTTACCGTTACCGTTACCGTTACCGAATCCACCGCCGAATGAAGCGCAGCCGCTTCCGCTCTGTGTTTTCTCCGGTCTTCCGCTCTTCCCGGCTCCTACGAGAGCTAGAAACGCCGCCGGCGCGTTGCAGCCGCCGCCGCTCCCGGCGGCCGGCTGCGGTGGTAGTGCAATTGGAGTTAACTCCTCCTCCGGCATGGGGGATAACGGCACCGCGATGGCGTGGATCGACGGCATCATTAAGGATCTAATCCACAGCTCCACTCACGTCTCGATCCCTCAGCTTATCCAGAACGTGAGAGAAATCATCCACCCGTGTAACCCTAACCTCGCCGCCCTCCTCGAGTACCGCCTCCGCTCTCTCACCACCGCCGATCCTCTCGCGGCCAACGTTTACGACGATTGGAGAAGAAAGGAAACACTACAACCTCAGAGCCAAGACGCCGCCATTACTCATCCCCTCCACCTTCCGGATTCCATGCCTCATCCTTGGGAAATAACTCTCCCTCCCGCCGCCGCAACCGCCTCAACTACCAGGCACCACCACCACCACCACCACCAACAACATCAGCTCCGTGACAATTCTCCGTCAGTAACCGCCGGCCTGCCGTTTGTCCCGGCTCCGAGCTCAGATCGTCAAGAGCAACAACAACAACAAGGGAGAATGGATCATGAAAAGCAACCAGAATCTCAGTCCCAGTCCCAATCCCCGCCGCCGAGCGAGAGCACCGCCGCGGCCGCGGCGTTGATTAGGACGGAATCACTCAGAAGAGAAAAGGATGAGCTAGAACAACAGAAGAAGGACGAAGAAGGCCTCCATCTATTAACCTTACTCCTCCAATGCGCGGAGGCCGTGGCGGCCGACAACCTAGACGAAGCAAACCGAATGCTCCTCCAAGTCTCGGAGCTCTCAACCCCCTACGGAACCTCCGCGCAGCGAGTCGCCGCCTACTTCTCGGAAGCCATGTCCGCCCGCCTCGTCTTCAACGGCATCAGCCCCTTCGTCAAATTCTCCCACTTCACAGCCAACCAAGCCATCCAGGAAGCCTTCGAGCGAGAAGACCGAGTGCACATCATCGACCTCGACATCATGCAAGGTCTGCAATGGCCAGGCCTGTTTCACATCCTAGCTTCTCGTCCCGGTGGGCCCCCTTTGGTCCGCCTAACCGGCCTAGGCACCTCCATGGAAGCCCTCGAGGCTACCGGCAAACGCCTCTCGGATTTCGCCCAGAAACTCGGATTACCGTTCGAATTCTTCCCCGTCGCCGATAAAGTCGGGAACCTGGACCCACAACGGCTTAACGTGAACAAGCGGGAAGCCGTCGCCGTTCACTGGTTGCAGCATTCTCTGTATGACGTCACCGGCTCCGACACAAACACGCTTTGGCTCTTGCAAAGATTGGCTCCAAAAGTGGTGACAGTGGTAGAGCAAGACCTGAGCCATGCAGGATCGTTTCTGGGGCGATTTGTGGAAGCCATACACTACTACTCGGCGCTGTTCGACTCGCTGGGGGCGTGCTACGGCGAGGAGAGCGAGGAGCGGCACGCGGTGGAGCAGCAGCTGCTGTCGAGGGAGATTCGGAACGTGCTGGCGGTGGGGGGCCCGTCGAGGAGCAGCGAGGTGAAGTTCAACAACTGGAGGGAGAAGTTTCAGCAGTCGGGGTTCCGGGGGGTGTCTTTGGCCGGAAATGCGGCGGCTCAGGCCACGCTACTGCTCGGAATGTTTCATTCCGATGGGTATACATTGGCGGAGGATAACGGCGCACTAAAGCTTGGTTGGAAGGATCTCTGCTTGCTCACAGCTTCTGCATGGAGGCCACCTCCCCTTGCACAATAA

>IbGRAS68

ATGATGCAACCACAGCTTTTTCATCCTTCCTGGCTATCCTATGAAACCAATTACTCAACTTCTCCTTGTACTGGATCCTTAAGTTTTTTTGAGGATGTTTTTGTAACAGACAACAGTTACGTTTCTCCAGTCATCACAGCAGACTCCTCGGGGCTAGATTCGACCTTATTCCATGATGATTTTCCTGAATTTGCATATCTCCCTCCACTCCTGGAGGGTGATGTTTCCATGGATGATATTGAGGATGTTTGCAGGTGGTTAAACAATGAGGAGAGTGAAGAGGGAACTAATAATACCTCTTCGGAGCTGACAAAGGATGTTTTGAGCCCCGATTTCTCAGTTGTGTCTGCTGAGGATTCCATGGCGGTTTTACCAGGAAATGGGGTGGAAGTGGATGATAGCCATTGGTGCCTTCTTCACTTGCTAGCAGCTTATGCAGAGGCCATGGGGGACATGCAGAGGGAGCTAGCAAAGGAGATCGCGGGATGCATAAGGCGAAAAGCAAATGCTTTAGGAGAAACACTTGAGCGGGTTGCATACAACGTTGTCCAGACCACGGAGGATCAAGGAGGCAGTTATCTGAGGCGCGAAGCAATCAAGAACTACGAGACAGCATTCAAAGTGCTGTACCAGGTTCTGCCACACGGGAGATTTGCACACTTTTCTGCTAATTCTGCTATCCTTGAAGCCATACCGGATGGTGCAGAAGCAGTTCGTATAATAGACTTTGATATGGGAGATGGAGTCCAGTGGCCCTCCCTGATTGAATCCATGGCTCAGACAAGAAGGGCTCTGAGACTCACATCTGTGAAGAAGGGAAGAGGAGTCTACCATCAACGAAGCGAAAAGAACAAAGAAAACAGGCAAAAGGAAGAATGGTTAGCCTTCAACTGCATCTTCAGGCTCCCACACATGACAAACAGGCAACAAAGAAGTCAAGCCATGCAGTTTCTAGAGATAGCTAAGGAACTGTCACCCTATTCTGCAATCCAATCTGGGATTGTGGTTTTTGCTGATGGAGAATCAGGGTGTTGGAGCAGTTCATTTTCAGATTACAGTTCTTTCTTTAACAGGCAGTTAGTACACTACAAATCCCTGTTTGAATCAATGGAGTGGCACTTCCCGGTTAATCTCACCGATGCAAGGATTGCAGTAGAGAGTCTTTTCTTAGCACCTCATGCCTGCTCTGATTCTTGGTTTCATGACTGGCAAGAAAACAAGATGAAGGCAATTTCCAATCTCCGGGCAGAAATGGGGTTGCAAGGCCGGAAGCTAAGCATAGAAAACATTTTACAGGCTAAAGAAATGGTGAATGAAAGAGAGAGTCCATACAGAGTTAGGATTGAAGAAGAGAATCAGCATGAGATGATCTTGGAATGGAGAGAAACGCCATTGGTGAGAGTTTCAACCTGGATGTAA

>IbGRAS69

ATGCAAGCAGCACAACGCCTCAGGAGAACGGGCATGTCTAACGTGTTATGCTATCAGCCTGTGCAAAAGGCCGACCCTTACCAGCTGAGCTACGACAACACAAATGGAGCTAGCTCGTTTCATGCTTCTCGTAACCTTTATTGCACTCTCGAATCATCCTCGGTGAGCGAGAGTCGTGTTTTATACAACTCACCATCAGCTGTCTGTTTCTCAACCGATGGAAGCCCGATGTCCCAGCAGGATTCTTGTTCATACCCATTGGACAACAACTATGGTTCCCCTATTAGCGGGTCTTGCATTACAGATGATATGAGTAGTTTCATCCACAAACTAAAGGAATTAGAAACGGTAATGCTTGGACCCGATTCAGATATTCTGGGGAGTTACGATAACGCCTTCCCGAGCAGTATAGCCTCCCCGGAAATAGACAGCTGGAGACAAATGATGGAGGCCCGAGGGGATTTGAAACAGGTGCTTATTGCTTGTGCAAAGGCGGTCTCGGATAATGATTTGCTGACAGCACAGTGGTTGATGTCTGAGCTCCGGCAAATGGTGTCTGTATCGGGCGAACCAATCCAACGCCTGGGAGCATACATGTTGGAAGGGCTCGTTGCTCGGTTAGCTGCATCGGGGAGCTCCATCTACAAATCCTTGCGGTGCAAGGAGCCAACGAGTTTCGAGCTGCTTTCTTATATGCACATCCTGTACGAGGTTTGCCCTTACTTCAAATTCGGTTACATGTCAGCTAACGGCGCCATTGCTGAAGCAATGAAGGATGAAAATAGGGTTCACATCATAGACTTCCAAATCAATCAAGGCAGCCAGTGGATTACTCTAATCCAAGCTTTTGCTGCTCGGCCTGGCGGTCCACCCCATATTCGAATAACAGGTATAGATGACTCGAGTTTATGTTTATCTAATGCACACAGTGGAGGAGGATTGAGTCTTGTAGGTAAAAGGCTATCTAAACTCGCGGAGTCCTTCAACGTGCCCTTCGAATTCCATGCTGCAGCCATACCTGGCAGCGACATTCACCTCGAAAACCTCCGGATTCAACCGGGGGAAGCACTGGCTGTGAACTTCGCCTATATGCTGCACCACATACCAGACGAGAGCGTGAGCACTCAAAATCACCGGGACGAGCTATTGAGGCTCGTGAAGAGCCTGAATCCCAAGGTGGTTACTCTTGTAGAGCAAGAGTCTAATACCAACACTTCTGCATTCTTCCCCCGATTCCTCGAAACCTTAGACTACTACACTGCAATGTTTGAGTCAATTGATGTCACTCTTCCTAGGGACCATAAGGAACGGATCAACGTGGAGCAGCACTGTCTGGCGAGAGACGTGGTGAACATTATAGCGTGCGAGGGAATTGAAAGGGTGGAGCGACACGAGCTGCTCGGGAAGTGGAAGTCTCGGTTCAGAATGGCCGGTTTTACCCCGTACCCATTGAGTTCAGTGGTGAATGCCACAATCAAGAAACTGTTGGAAAGCTATTCTGATAAATACAGGCTTGAAGAGAGAAATGGAGCCCTTTACCTTGGTTGGATGAACAGAGATTTGGTTGCTTCCTGTGCCTGGAAATGA

>IbGRAS70

ATGACCATTGATGAACCAGCAGAGCCCCGTCCTTTCACAGACCAAATCTTGGAATGGTTCGATTCATCGTTTCTTGATTGCCCTTATGATTCCAATGACCATTTCTTTGGGGATTCATGGTGGGGGGATCAAGGTCAAAATCTAGAGATTCTGCACAAGAGTGATGATAATGGTGGTGGTGTTTCTACTTCTTTGAATAGTTTCTCTAGCGTCACGACGGCTGTGGAGGCGCCTGTTGTGTTGGATCATCCGGCGGCTCAGCCGCCGGTGGATAAGAAGCGGAAAGGCCGTGAGGAGGGGGAGGCGGAGGTGGAGGTGGAGCAGGCGGCGGCTCCGGTGAGGAAAGGGGGTGGGAATAAGAAAGGGGGGAATAAGGGAGGAGGGAATAGTAATAGTAATTGTAACAAGGATGGGAGGTGGGCTGAGCAGCTGCTTAACCCTTGCGCCGCCGCGATAACGGCGGGGAATATGAACCGGGTTCAGCATCTGTTGTATGTTCTCAGTGAGCTGGCTTCGTTGACGGGCGACGCTAACCACCGGTTGGCCGCCCACGGCCTGCAAGCGCTGACGCATCACCTCGGCTCCGGTTCCTCGTTCGCCGGAGTGACGACTTTTGCGTCGACCACTAAAAAATTCTTTAGGGAGTCGTTGATGATTTTCAACGACATAAATCCGTGGTTTCGCATCCCGAATAGCTTCGCGAACTCGTCGATTCTCCAGGCTCTGGCGGAGCAGCAGGATCGGCCTAGATGTCTTCACATCCTGGATATCGGAGTTTCCCATGGGATCCAATGGCCGACGCTTCTAGAAGAGCTTACTCACCGCCCGGGAGGGCCGCCGCCGCTGGTTCGTCTCACGGTCATCACCCCCACCGTCGATAACCAGCAGTTGAGCTGCAATAACACTCCATTTCTGATTCCTCCATCAGGTTACGATTTCTCCCCCAATCTTCTAGGGTTTGCCAAGGCTATTAACATCAATCTACAGATCAATATCCTAGACAATCTGCCGCTTCAGAACCTGATCGCCCACGCGCAATCCATCAAATCCTCCCAGGATGAGATTCTAATCGTCTGCGCGCAGTTTAGACTCCACAATCTGAACCACCACGCGCCGGACGAGAGAACGGAGTTTTTGAAATCCCTAAGGAATCTGGCCCCGAAAAGAGTTGTTCTGAGCGAGAACAATGCGGAGTGCAGCTGCAGCAACTGCGGCGACTTCGCCGCCGGATTCTCCCGGAAAGTTGAGTACCTGTGGAGGTTTCTGGAGTCGACTAGCATGGCGTTTAAGGGGCGGTGGAACGAGGAAAGGAGGATGATGGAAGGAGAAGCGGCAAAGGCGTTGATAAATACGGGAGAAATGAACGAGAGAAAAGAGAAATGGTGCGAGAGAATGAGAGGCGTTGGGTTCGCGAGGGAGGAGTTCGGAGAGGACGCCATTGATGGAGCTCGGATGCTGCTGAGAAAATATGATAACAACTGGGAGATGAAAGTGGAAGAGAAAGATGGGTGCGTGGATTTATGGTGGAAAGGGCAACCACTTTCCTTTACCTCACTGTGGAAGATGGATCCTAATCATGGTTTTAATTGA

>IbGRAS71

ATGGACTCACATCAGTTTATTGCATACGGAGTGGATTTATCGTTCTCGTCCTTCCCCCAAACTTCATCACTACCTGCCACGCTATTTGATTCCTTGAAATTTGACTCGACAAGTTCTCCCAATTCGCCGTTCAGTAATTGTTTTGATCCCCAGACCGCGACGACTTTAAGTGACAGCCAGGAGCTCTACAGCTCTACAGAAAATCTTTCGGGAGCCAGTCCCTCCAGTAATTCTTCGCTGGATTATAACAGTTATCCCCAACGGTGCAGTCCCGCCTCGGACTGCCTGCCAGAAAGCTTGGTTCTTCCTTCTGGTGAATACACTTTCCTTCGAAATGTAAATCACAATGAGAAAATGAAGCATGTTTTGTGGCAGTTGGAGAGCGCTTTGATGGGGCCGGATGGGGTAGGAGCCACGAATTCAGATCCATCTGCGGGGGAAAATACACAAAAACAAACATCGAGTCAGGGGTCTAGGTCTTGGAGCCAGGAGGCCCAGGGTTCTGGCCGATATGAATCTCAGCAGTCTTCATTTGGGAGATCGGGTGAAGGAATTCATAGCGAGAAACGCCACAAGACGATGTTAGATTTTCCTGCACAGGGCGGTCCTGTTAATATCAAACAGCTGCTTATTGAATGTGCGAGGGCTCTCGATGAAAATAAACTGCTCGATTTCGACAGACTGATTGAGGTAGCACGGTGTGCTGTGTCTATTACAGGGGATCCTATTCAGCGCCTTGGGGCGTACATGATCGAAGGCCTAGTTGCGAGGAAAGAAGCGTCGGGCACAAACATTTATCGGACGTTGAAATGTAAGGAGCCAGCGGGGGACGACTTACTCTCCTACATGCACATCTTGTACGAGATATGCCCGTATCTTAAGTTCGGTTACATGGCTGCAAATGGCGCCATAGCGGAAGCGTGTAGAAACGAAGATCGCATTCACATTATAGACTTCCAAATTTGCCAGGGGACTCAATGGATGACACTTCTACAAGCGCTCGCTGCAAGACCTGGCGGTGCCCCCTACGTGCGCATCACGGGGATTGACGATCCGCTTTCAAAACATGCTCGGGGGGATGGTTTAGTTACAGTGGGGAAACGCCTTGCAGCTATATCCGAGAAATTCAATATCCCGGTTGAGTTCAATCCGGTCCCAGTTTTTGCCCCGCAAGTCACCCGGGAAATGCTCGATATAAGGCCTGGCGAGGCCCTGGCGGTGAACTTCCCGTTACAGCTCCACCACACCCCAGACGAGAGCGTCGACGTGAGCAATCCTAGAGACGGGCTTCTAAGGATGGTGAAGTCGCTTTCTCCCAAGGTGGTCACGTTGGTGGAGCAAGAATCGAACACGAACACCGCCCCCTTCTTCAGCAGGTTCATCGAAGCTCTCGAATACTACTCGGCCATGTTCGAGTCGCTGGACGTGGTCCTACCGAGGGACAGGAAGGAGCGCATCAACGTCGAGCAGCACTGCCTGGCCCGAGACATTGTGAACGTGATAGCTTGCGAGGGCAAGGAGAGGGTGGAGCGCCACGAGCTGTTGGGGAAGTGGAAATCGAGGTTCACCATGGCGGGATTTCAACAGTATCCCCTAAGCTCTTACGTTAATTCGGTAATTAAGGGCCTGTTGAAGTGTTACTCTGAGCATTACACACTTTTGGAGAAAGATGGGGCTATGCTATTGGGGTGGAAGGAGAGAAACCTCATCTCTGCTTCAGCTTGGCACTGA

>IbGRAS72

ATGATGTTAAACACTCTGTGTGGGAGCATGGGAAGTTTGAGCAGTGAGGTGGGGAACCTAAAGATTGAGGAGGAACCCAGCAACAGCGTAAAGCGTGAAAATTCATCATTGTTGTCATCGTCCTCGTCTTGTTCTACTCTGCAGCAGCATCAATCGGACAAGGTTCATGTCCCTGCACCATCCGATTATGAAGACCACAGTAGCGTTGTTAATGGCATCAGCTTTCCCGCCATCAAATTTGAGGTGCCGGGCGGGGGCGATGACATCGGGATTGATGATCAGTCATACTGGGAGTCATTTTTCGCGGATCAGCTTGAGGGGGATTTTATGATCTCTTCCCCTGTTAGGAGCAATAATATGGCCGCCTCTCCTCAGGTTTCCAGCTTTATGAACGATGTTCAGATGATGATCACATCTCCGGTCAGGAACTGCTATCATCACCATCAGAATGGGATCACCATGTCCCCTCCCCGGATGATGATGATGTCTCCTCTCGGCCCCGGCCACAACAACAAAGGCAAAGGGTTGAGCCCACTCCACCGGGTATTCAACTCCCCCAACGCCCAACACTTGATGCAAATGCAAACCCATGACTTCTCCCACCTGCCCGCTCTCGACAACTTGCTAGATTTTGACGCTCACCAAGACGTACTTGACGATAATCATGATTTCTCCTCTTACTCCTCCACCTTAAAGCACCTCCCTGCATCAAGCAGCGTTCTATCGGAGTCCTTGTTGGACTGCGGTGGCCTGCAGCTGCCCCGCCTTCATTCTTCTTCTTCTTGTTCGGCCTCGGTATCATCGATCCAAGCTCCACCACTAGAAGCAGATGATGACATAATTTACCGGACCACTACTACTACTACTGCAGCTGGTGGATCCCTCCATATCGCACCTTTGTCGCACCAACTACGAGAGGAGCGCAACCAAGAACAACAGCACCACCATAATAATAATAATATTAGACGTAGTGGACAACACCTCCACTCTGCACCCTTAGTTCTTCCTCTCTCTGCTCCCCCCGCCGACCTCCAGGAGGAGGAGGAGGAGCAAGACAGTGGATTGCAACTGTTGCACCTTCTGTTAGTGTGTGCAGAAGCAGTATCCAAAGAGAATTACATGTTAGCTCGGAGATACCTCCACCACCTGAACCGAGTGGTGACGCCCCTCGGAGACTCTATGCAACGCGTTGCGTCTTGTTTCACCGAGGCCTTGAGTGCTAGACTAGCTGCCACTCTCGCCTCTGCCTCTGCCAAATCCATCACCTCCAATAGGCCCTTTAACCCTCCCTACCCTCCAAACTCCCTGGAGATCCTCAAGATCTATCAGATCCTTTACCAGGCTTGCCCCTACGTTAAGTTCGCCCATTTTACTGCTAATCAGGCAATCTTCGAGGCATTCGAGGCTGAAGAGCGGGTGCACGTAATCGACCTTGATATACTCCAGGGCTACCAGTGGCCGGCTTTCATGCAGGCCTTAGCAGCCCGGCCGGGCGGCGCCCCGTTTCTCCGGATCACCGGAGTGGGGCCCTCCCCGGAAGCAGTCCGAGAGACGGGTCGATGCTTGACAGAGCTAGCTCACTCCCTCCACGTCCCTTTTGAATTCCACCCGGTGGGAGAGGAGCTGCAGGACTTAAAGCCCCACATGTTTAACCGGAGAGTGGGGGAGGCGTTGGCGGTGAACGCGGTGAACCGCCTCCACCGGGTTCCGCCAAACTGCATGGGGAACTTGTTAGGCATGATTCGAGACCAGGCACCCAACATCGTGACCATCGTGGAACAAGAAGCGAATCACAACGGTCCATACTTCTTGGGGCGGTTCCTGGAGGCGCTGCATTACTACTCCGCAATCTTCGACTCGCTGGACGCGACTTTCCCGGCGGATTCTTCGCAGAGAGCGAAGGTGGAGCAGTACATATTCGCGCAGGAGATATGGAACATAGTGGCCTGCGAGGGAGCGGAGAGAGTGTATCGGCACGAGCGGCTTGAGAGGTGGAGGAGAGTGATGGAAGGGAAAGGGTTCAAAGGGGTGCCGCTCAGCGCCAACGCGGTCACGCAGTCAAACATATTGTTGGGTTTGTATTCTTGCGATGGCTACAGGTTGACGGAGGATAAAGGTTGCTTGCTCTTGGGGTGGCAGGACAGACCCATTCTTGCTGCGTCTGCATGGCGATGTTGA

72 amino acid sequences

>IbGRAS1

MLGSFGSSSSSRSLDEQDTTNNHNNNEPPSSVVAPLPPFQFHQIQRRRTVFASSSSSPPAIQMRQFLISCAELISRSDFSAAHRLISFLASNTSPFGDSSERLAHQFTRALSLRLHRSISASAASAVAPLPFPELQMEQPGLVQSSYLSLNQITPFIRFTHLTANQAILEAIDDGQRNAVHILDFDVMHGVQWPPLMQALADRYPPPTLRITGTGNDLDILRRTGDRLAKFAHSLGLRFQFHPLLLSNNEDDEDVDPPLSSVVLLPDETLAVNCVLYLHRLLKNREKLNRFLHRIKSMRPRVVTVAEREASHNSSCFLQRFVEALDHYAVVFDSLEATLPPSSRERVAVEQVWFGREIVDIVAAEGEKRKERHEKFRTWEMLLRSTGFSNIGLSPFALSQAKLLLRLHYPSEGYQLRVIADSFFLGWQNHPLFSVSSWH

>IbGRAS2

MDSHQFFGYGVTGADFAYSSYPDIPSIPIRHIGSLKFDSRTSPNSPFAPHFDPQTPTSLSDSQEQQSSTDNLSGVSASSNSLLDYSSYFQQRSSPLDCGPESLLIPSGATSFHRNQKAKHVFWQVEPALIVPDQTGAKTSDLSLSENKHQQPLGQKSRSWSQEGQISGLIEFQPSRVSSIGKPDGSVHSLKRYKTVQDFPLQGVPQGNIKQLLISCARALLAENKDNFDRLVEEARVSVSITGDPIQRLGAYMIEGLVAKKEASGANFYRGLKGKEPAGKDLLSYMHILYELCPYLKFGYMAANGAIADACRNEDRIHIIDFQIGQGTQWMTLLQALAARPSGAPHVRITGIDDPVSQYARGDGLAAVGKQLAAISEKFSIPVEFHAVPVFAPKVTRDMLDIRPGESLAVNFPFQLHHTPDESIDMNNPRDGLLRFVKSLSPKVVTLVEQESNTNTAPFFPRFLETLDYYSAIFESIDVTLARDKKERINVEQHCLARDIVNVIACEGQERVVRHELLGKWKSRFTMAGFREYPLSSYVNSVIKSLLKYYSEHYTLVEKDGAMLLGWKQRNLISASAWH

>IbGRAS3

MEFESFDEVSLSPSYIHEFQKHGSKNSSFTSWVVDYGQEYGLEAGTDSFDSWITDCGQEYGLKVHTGGSVGSWVTECGQEYELEVCTDNSVGSWVTDYGQKDQYYSNSNDVDIQECLQLETDIMDLDFIDEDAVFSQNREQETPTEKAGVSGFLTPENYTSHLKTIQEELMEETSLTDLLLMGAQAIQAGNKDLASMIVLKLNNPQISSEEETGHLDRLALYFTQGLALKTMNFGDQNWQLGSMSAFQMLQEISPYIKFAHFTANQAILEATQSQRDVHIIDFDIMEGIQWPPLMAELADRENASLRITAMVTEPQTWAHTQQTGKRLHEFANSINLSFSFDQILLTKEQDLEQIQSLGNNPIANLMIHQLHIPHRESSLIKIFLNGLRNLSPQMIIMVEEELFNISRTPSMSFVEFFREAIHHYTSLSDSLQGGFCGGYKLAQRVIEKEFLGPRIVDCVRQFPSGKRERQIWSQGVYSLKGFKAIPMSSCNVSQGKYLVSLFNGGFWVQHEKCRLALCWKSRPLITASIWVPTSSSISHSLSRSTSFD

>IbGRAS4

MDNNEDDFSSSSYNNTHFSDYSHHQTPIDHNFSFSPGFDSGSAFSGDDILLHAARAVADKNAPRVRQLIWVLNEISSPYGDTDQKLAYYFLQALFSRVTDSGERNCRKSASASERNCSFESTRKTALKFQEVSPWTTFGHVACNGAIMDAIDGESKIHIVDISNTFCTQWPTFLESLATRTDDPPRLRLTTVVVRKLGGGGGPPAAVQTVMEEIGSRMEKFARLMGVPFKFNVVYHTGDLSEFDILGLDVEEDEALAINCVGALHSVANSRRGQMISTFRRLQPRVLTVVEEEANLDVGVDGSDFITGFQECLRWFRVYFECLEESFPKTSNERLMLERQAGRAIVDLVACPPSESVERRETAARWSHRLHAGGFSPASYGDEVCDDVRALLRRYREGWSMGQCSGESSAGIFLSWKDQPVVWASVWKPALSDGDGR

>IbGRAS5

MQEEADEGEKGHAIIFKFQLLDVSLTCLRVGLILIMSENNGIKLIKLLLTCASHVSSGDLHQADVCLRQISQSNSASNDSMHRLATWFASALAVRLVKRWPGVYKALNCSSLMKFDLDRARSIFTKALPYLGFAYAVINRTLVQAMLGERVVHVVDLGSSDQQLWVPFMRILATSPDGPPHLRITCVSSNKTALDKLGSCLKKEAEQLDMPFQFNPVNVHHLRDLNFNSIVKVRSGEALAFISVLNLHVLLAEDDRIDAQFGLNKDSKNIKHCKHADEFLAKLCSLSPKLVMLVEQESNHNLQKFVDRFVEGLRYYSAMFDSINVAFKGDLCEERLLVEEMIGKEIENIVACDGLEREERHEKFANWMVRLGHAKFRPVRLWGDTMDDAIRFVEYVRWRWVQD

>IbGRAS6

MIWNMEGVVDEELLSLRLGIATGSNEKKIMRRKRKRRDHDDFINNNRLVLSDDESYEGQIFSLLQTREQMLKVEKRVRAVTEEEENDKGGGLHLIHLLLVSATAVDENTPGPAAESLSELYRRASLSGDAVQRVAAYFADGLVARLLTRKSPFYDMIMKPPTPHEHLFAFTHLYRASPFHQFAHFTANQAIIEAFHQESQTNNASLHVIDFDVSHGFQWPSLIQSLSQSLSPPSKISLKITGFGPSLSQLRETEARLVSFAKGFRNLSFAFTGLLLDSIDYYTSAKTKTKTKTHETVAVNLAFYLNRLPNFSDTLRTVHLLSPSVVTLIEQEGCRSPRNFLSRFMESLHYFAAMFDSLDDCLPIDSPERLSIEKNYLGNEIKRAMTNNGSEELVPRYEKMDTWKARMESHGFCGVRLSCKNVMQAKLLLKIASSGHCCRVSFDGGFRVFERDDGKAISLAWQDKPLTTASVWRCV

>IbGRAS7

MANDGVCETLVDRNEEMGNLNSEEAPLPQSHQRLLNEDYDLRDQMLKWINQMLMEDNAEEKAYMLRQSAALKDAERSFYELIGEEYPLSPNLQRVPDLDRNENYGNDDSLCPNRDPYPRERQTTSDHVASSTTFQSSSLLGQGTENDGTVDFAASSITIPDFSNCTESVEKGVREASSFLPTRNSLLVDGIGAKKNTGNQDTLEGRRGTKNTLREDTHLPEGRSYKQSAIYAEPSIKQEEFDKVLLWSGEDESSLCHSLRGVLCEGNDDSKGSNRKKSSGKKPGSERTVVDLRSLLMLCAQAVAVEDIRTANDYLKRIRQHSSQTGDDMQRLAHYFADGLEARIAGSGTRIHKALMKYPRKISNMFSNKTITTLAQNASSLHIIDFGIGTLFGFQWPCLIQHLSSRPGGPPKLRITGIDFPLSGFRPAERAEGTGRLLAYYAEKFNVPFEFNAIAKQWETITVEDLKIIEGEVLAVNCLYRLRNLLDETVVVNSLNPRDTVLKLIHDVRPDVFIHGILNSAFHERMLIESYIFGQQAMNAIACEDTERVERPDTYKMWQARNTRAGFLQLPLNREIVKMSMHMLKRYHKEFVIDEDGHWLLLGWKGRAIFALSSWKPA

>IbGRAS8

MANEGVYETLVDRNEEMGNLNSEEAPLPQSGQRLNVVNLLNEDGGYEDYDLKGQMLKWINQMLMEDNVEEKAYMSQQSAALKDAERSFYELIGEEYPPSPNLQRVPDLDRNDNIGSGDNGNDDSLCPNLDPNPREGQTTSDHVPVNVASSTITIPDISNCTESVEKGVREASSFLPTRNSLLVDGVGAEKNTGNQDALEGRRGTKNTLREDTHLPEGRSYKQSAIYAEPSIKQEEFDKVLLSSGEDESSLCHSLQGVLCESATGNDDSKGSNRKKKPGSERTLVDLRSLLTLCAQAVAVEDIRTANDYFKRIRQHSSQTGDDMQRLAHYFAEALEARIAGSGTRIYKVLMKYPRYAARALKAFQLYLSSCPFVKISYLFSNKTITTLAQNASSLHIIHFGIGFLFGFQWPSLIQHLSSRPGGPPKLRITGIDFPQSGFRPAEKAEGTGRLLAYYAQKFNVPFEFNAIAKQWETITVDDLKIIEGEVLVVNCIYQLRKVLDDTVVVNSLSPRDTVLKLIHEVHPDVFIHGILNSACNSPLFTSRFRAALSHYSAVFDMLEVTIPREVHERMLIESYIFGQQAMNTIACEDTERIERPETYKMWQARNTRAGFLQLPLNREIVKMSMHTLKRYHKEFVIDEDGHWLLLGWKGRAIFALSSWKPA

>IbGRAS9

MDRGPYEATSQFNFEGEYEHYDLRDQMLKWINQMLMEDNVEEKAYMSRQSAALKDAERSFYELIGEEYPPSPNLHRVPDLDRNENYGGSGYYGSGDNGNDDSLCPNWDPNPRERQTTSDHVPINVAPSTTSQSSSLLGQGTVNNGTVDFVASTITIPDISNFTESVEKGIREGSSFLPTRNSLLVDGVGAEKNTGSQDLLEGRRGKKNMCRDDMHLPEGRFYKQSAIYAEPSIKQEEFDEVLLCSEEDESNLCHSLQSVSCESATGNDDSKGSNRKKSSGKKPGSERTVVDLRSLLMLCAQAVAVEDIRTANDYLKRIRQHSSQTGDDMQRLAHYFADGLEARIAGSGTRIHKALVKYPRYAARALKAFKLYLSCCPFRKISNMFSNKTITTLAQNASSLHIIDFGIGTLFGFQWPCLIQHLSSRPGGPPKLRITGIDFPLSGFRPAERAEGTGRLLAYYAEKFNVPFEFNAIAKQWETITVEDLKIIEGEVLAVNCLYRLRNLLDETVVVNSLNPRDTVLKLIHDVRPDVFIHGILNSACNSPLFTSRFRAALSHYSAVFDMLEATIPREVHERMLIESYIFGQQAMNAIACEDTERVERPDTYKMWQARNTRAGFLQLPLNREIVKMSMHMLKRYHKEFVIDEDGHWLLLGWKGRAFVVETSLVN

>IbGRAS10

MFQDNDGSSSITSLSPRMPAGIGSLCHWFKELKPEERGLYLIHLLLTCANHVAAGALDSANVALDQISHLASPDGDTMQRIASYFAEALADRILRSWPGLYKALHSTRLPVLSDQVTAKKMLYDLFPFLKVAFMVTNQAIIEAMEGEKLVHIIDLNAAEPTQWRPLLQDLSARPEGPPHLRITGVHPHREPLDQMARTLTDEAEKLDIPFQFTPIVTHLETLDFQQLRVKTGEALAITSSLKLHTLLAHNHEPGKNPNPQRLGDNNFLENHKIKGFLDAIRGLSPQNRGDLRAREQPQRGNSHAETIRVAPLLCSIIRLPRIHAAPNILGQTKGGEAAVGRGDQEHHSLRGNREERTAREAGQMVLEIRVVGFLERAAELLRHVASSEVFAELPVRWV

>IbGRAS11

MDPRFTGLPDSVSSFKVEDEFLFSSFQQSQDFPNNYLDGSLPDFMGVPLITPSPDSGYFPPPAELDSPDDQDADPVLKYLNQILLEENIDEKPSMFHDPIALKAAEKSFYEALAKGPPSPYQSHGNYNTESPDSLFGNSCEPSTSSSTFGSSNTDPLWIVEPEESKSSFASYPPDHFFHSSFETTSERSNESILQFKRGMEEASKFLPASNQLVIDLDQYSLPPKTEELPRDTVIKSENSERDSSHNSSRGKKHHHLEDDGFEEERSSKQSAVYVEEELSELFDKVLLCNPNECVLTTRPPEVDKGVPHNVPNSGKSRSKKQGATNEAVDLRTLLISCAQSVAADDRRTANEQLKQIRQHSSPNGDANQRLANVFVNGLEARLAGTGTQLYAALAPKRITAFEKLKAYQVYMSACPFKKIAMTFANKMIHKISLGAPTLHVIDFGILYGFQWPILIQHLSSRPGGPPKLRVTGIDLPQPGFRPAEMIEETGRRLGKYCERFGVPFEYNAIATQNWETIKIEDLKLVSGEVVAVNCLYRFKNLLDETVAVDSPRDAVLSLIRKINPSIFVHAVINGSYSAPFFVTRFREALFHFSALFDMFDATIPRDDQHRFHFEQEFYGRETLNVIACEGVERVERAETYKQWQVRTMRAGFKLLPLNPELMTKLREKKAAGGYHRDFLFDEDGKWMLQGWKGPDYLR

>IbGRAS12

MDRFDSNYNHQFTSTSSSSSSSGDLSIESSSCGGDDSTPLERDYFDGVLKYINQMLMEEEDLENRPCMLQDSLALQAAEKSFYEALTDCNFSDERNRKRDNYGDGDVEGRANKLVAGFTAEESEQTEAYDKTLLCSANNPGFYSDPPWCHLDYSMEQTPRFTPAVTVQSKRGRPRAGEKRVGSGKPVDLRSLLLESAEAAANYNGRTASDRLKLIRQHSSPYGDAAERTAHYFANSLEARLAGTGTELYTAFSRRRMSAAEMLKAYQESNGDSHNRFRDSIRIPMAVFHPGNLPPPIRPAKASNHRHRFPQPGFRPAERVEDTGRRLANYCKRFNVPFEYTAIAAKDGTPLSLDELKTDREELLIVNCLYRLKNTPDDENSPRDAEALFHFSSLFDMLDAAIPREDQERLVYEREIWGRDIMNIVACEGSERVERPETYKQWQLRNQRAGFKQVALNQDVVKEVRAKVRLRYHKDFLVDEHSNWMLQGWKGRVLCALSCWTPVEKAMTG

>IbGRAS13

MGTLFRAHHNSRDESEFNHHCSVPTSGGLDRNLVLNEGCSDVLGFSGMKSVHDGPAPSEGVEGDYFDGVFKYIQNMLMEEDDLEHIPYMFQDCMALQAAEKSFYDALTENPPPPPLNHRNLSDSGNRNAQFSVSDNQVASEPFPAQFPLPGNQNFQAGLQELQPGYNPFPGQVENPMIPMNNSSGSMFSTQFSGESGTYLIGNGCSGNGERSNGNGPERRRNRNRENGREGQRSKQIATGNSDQEPEKTEKYDKALLCPSMNPLFYDDSIPYLSDESSETEARDKKYLQGPKRGRPRGSKKGAKPKQIVDLTDLLARCAQAEAAHDKKNFDLRLAAIRQHSSQYGDATERLAHCFANAVEARAAGTGTSLYASITRRRMSAAEYLKAYQTYITACPFKRMSNIYANKSIAKLTREAEKIHIIDFGILYGFQWPCIIHGISLRPGGPPRLRITGIDFPQPGFRPAERIEETGRRLENFARRFNVPFQYTAIAKKWETITLEDLKIEKDEILVANLPIPDEKRPGRDRNGKQQPQSRRSQANKKNQPGILRPRDRQRDVQRPVLHNPIPRSLLPLLGPVRHVRSHNAPRRRREDAGRARTAGPRRPERDRVRRERASGAARDVQAVADEDAESRIPSAPIAPGDHQGSEGED

>IbGRAS14

MYYTTVAGEAAEAQLTCCRRKENRTEARLDPTAAGKKIDGRSVAVLCSQKLSRRCCRRKSTVTVASQILKKKLLSCCDHFHQSRSGAKLQRRRRRKARNLNTLFSSSSLSLFQTLCSSTYFLFFGVPCVSISVMEALVQELITTPNDFVYNHHSKPVNPTLSGQRFNLVERSAGEGEEGDYSDAILKYISQMLMEEEDLENQPCMFRDCMALHATEKYFSDVLNGSGDNSPLSIPQYDASFAGSSPSNCSSDLNNGDSDLSPQFQSSFNGSLFSQYASRGAAIGLPNDSNVLNNLDMEVKESEGENSPSKGKRNHYYYSSDDGSAETQRSNKHLASYAPADEPEPLDIRRGNPREGEIRRGKKKETQKEFVDLRGLLTQCAQAMASYDTRTVTQLLKKIRDHSSPQGNGNERMAFYLANALEARLNGTGTALYISNSPSNISAADILKAYQMYITASPFKKVSNMFANKYIRKVAAGAPRLHIIDFGILYGFQWPCLIQGLSTRPGGPPRLRITGIDFPQPGFRPAERVKATGRRLDNYCKRFHVPFEFKAIAQKWDTIKLEDLEIDRDDVLVVNCLDRLGNVPDETVVPDSPRDIVLDLIKKINPDVFIHGVVNGTYNTPFFVTRFREALFHFSSLFDIFEATVPGQGPTVVRGNGVRKRRDERHSL

>IbGRAS15

MMKGGGFEVIQHEQLWDHHHHHRYGDSVSEMGISIVGGGGASSNGAAGELSQWVEHVTRQLIEDLPESEEVAAPPPPRGEAVQPSTTAGCQESKRSSEDDDDGGMRLISLLLECAVAISVDNLGEAHRMLLELTQMASPYGASCAERVVAYFANAMASRVINSWLGICSPLINLKTLHSSFQIFNNVSPFIKFAHFTSNQAILEAVHGHARVHIVDLDIMQGLQWPALFHILATRVEGPPPHLKMTGLGTSMDLLVETGKHLSSFAKRLGLSFEFHPVGKKFGEIDDVSALKIRRGDAVAVHWLHHSLYDATGPDWKTMRLLRQVSPTVITLVEQEIVHGGSFLDRFVGSLHYYSTVFDSLGALLPSDDASRHTVEHCLLRREINNILAIGGPARSGEDKYRQWRSELLGNGFLQVGMSRNSIAQAQLILNMFPPSHGYSLVQGDGTLRLGWKETSLYTASAWTSPAPNSR

>IbGRAS16

MGDLVIYVGDDDCEIVCGAMCFCRICHEAEFESSKILEAPCGCSGTLKKFEDGYTAPPPKMLHTPPVTIWESSENPRIEENSRVAGEEDERCALNDLTFLVLMGHLFELLAGEARRYPFSLITVVVIKAVGILLPAYLLIRIITLIQNGVIRPHLLAGHEEMEKQRSFSIKPTRFLVFTFTICLSAVFLIFFSVWLVKISPSPQENHLHLNSNKDPSVQVQENGSPVAEEYESEFAVEEDAGESKFAAGEVNATDVLNGNFTITTVAYFSSIVVNDTLLRGTHLTKSDLAVNQSENVQDCGSNGVDCNSKQQHVSDITFSKKIRGPRSSVIEKKSGRSVCDVTIGKWIVDESYPLYTNISCPFIDEGFSCQANGRQDKDYMKWRWKPQDCDIPRFNATHMLELIRGKRLVFVGDSINRNQWESMLCLLMGAIKDPKKVYETHGRRITKGKGNYSFKFVDYKCTVEFYVTHFLVREGKARIGRRRGQTLRIDAIDRGSSRWSGADILVFNTAHWWSHHKTRAGINYYQEGDQVHPRLDVSTAFQRALMTWASWVDTHIHPRKTQVFFRTSSPAHFSGGQWNTGGHCREASRPLKESFSTAYPKKNMIVEQVIRQMKTPVTILNITGLSDYRIDGHPSTYGRKSGSKSGVQDCSHWCLPGVPDTWNEILYYHLQLTQQHRIKLADLEFGGQEWGSDELYWLRIESPDRNSKIAIEISNLGSGKPLRCSCSPVGRRRGVACPRPPAPLLAESPIGRRQNGRSISRRRWLAAARLTPAPDRLSADLPPQPQQRRRPVSHSSSDLPPQQLLRTLPALQTSNGQSPRLKLHVDDSTCAPSLQTKSKPSTFTFSHIPMSSNSSAKAYDQGKASSSGKIIDDNKQLASGFQGHGDDVDLFCAKYGFYQENDQPLGVERQFITSNSSNNIQESTKLQAEKAWPPLSPAILRILGNRSNPFAATTSSSGGGRDHHQKLSAMQIVRLAGERFVQFSDHKFININIFKHPYSSTLSELCSQDKQDVELVQLLLAAAEKVSEQQFDRAIRLVSQCRNSASCTGSPVQRAAFYFADALQARIERQTGSSGHEDDDRNGVKDGECLASRFNKAYLILHQTLPFSQVVQLSATQTILDHVVTKPKVHLVDFYLRTGVQWSTLMQALSERAAAEDCGKQHYFRFTAIETTEKEKVEETGKRLQSFAAQFNLPFSFNVLYIPDLKHLKAEQVEIKADEAVIIHTSFTLRAMISKPLELESVMRAITRLKPCVMVVQEVEANLNSPSFVHRFIDALFYYSAYFDALEDTMRRDDQHRASIEAGSVRDGIRNIVAAEGRERVTRSVSLEVWREFFGRFGLEETELSQTCWAHANMVVQRFACKSCCAVSGNGRSLVVGWKGTPIYSFSAWKFPSLRRDN

>IbGRAS17

MGTSGFDPQNAAQNEANGEEEEDDHQILVLVNSPTLHDLYLDVAVQPPFDESSNKETHPAILPSSLALLKRFGRRFSKLKGQKKTNPRHRKDVELVGYLLSAAEKVGQSDYDSAEILLTRCDELSSHQGNSVERLVHYFSQSLWAKIFCQTDSSALFFQEDLEEALMSLRPCIAYHQKVPITQVFQFTSIQTVIEHVEDARKVHIIDLEIRSGVQWTILMQSFTESPRPEHLKITALQATKHQSKIEEETGMRLRSFAQSLNLCFSFNIVALEDLLNDNKEISLSGFQPDDEEEVVVVYASCFFATMISKQEKMESLMRVIKSVNPRVMFLTEVEANMNSVGFVNRFTEALFYYGAYFDALEDCMKSDEANRTTMEAKHFGQGIRNVVASEGESRVIRHVSIKVWREFFVRFGMEEMELSTPSVYQATLVLKRFGCGKSCTLDMDGKALTVGWKGTPLLSLSAWKFL

>IbGRAS18

MVQEEGSSSIGSSPLQYFSMMSLSPGIGSPYPWLGEMRSERRGLFLVHLLVTCVNHVAAGNIENANIVLEHISHLAASDGDSMQRVAAYFNEALADRILKGWPGLYKALKSTRITSAADENVVQKMFFELCPFLRLSYVITNEAIMEAMEGEKVVHIIDLNAFEPAQWISLLQAMSVRPEGPPHIRITGINEHKEVLEQMAHQLNEAAEKLDIPFQFNPIVSRLESLSIESLPVKMGEAIAISSVLQLHPFLAFDDEMLQRNTPPVVSRHANSVHLQRILQVNPRTLGDFLEKEVANPYGASPDSTSSSPLPLATAPKMMSFLNSLWSLSPKIMVMTEPEANHNGFSLMDRTMEALNFYAALFDCLESTIPRASPERQKIEKMVYGEEIKNIISCEGLERKERHEKLEKWIPRLELSGFRKVRLSYHIMMQGRRLLHSHNYDGYNLKDQDGCFLICWQDQPLFSVSAWRFQRYS

>IbGRAS19

MKGMPFPFEFERKGVIELGNRNNRNCVSGNHCWDIKDCVVGSPGGSNSEPTSVLDRPLASSSTLSSSCGGGGGGGETDAARVAAVSGNPASKWQQDNTTATSSNAGGGESELLQPVPPSLDFGGGAPPPWEAARRRRNVGWRSGIVGGGGAAEYDFNGGFGVVDHGFGAVDPVSSSGESFLPSIPISGSNFLANRLPNPPASLPGFKFSAPPPLFPPVSNNLGAAAFNPSLLEPSDLKPQIFNPGNPHFLINQPPQNPSFLMPLPFSRPELAPPQAKRHNPGGNLESPGPQIPRGLFSDQQTPSPHHMLPHQLQLLPNYPQRPKPPDISGEEMGHFHQNQQTMIDQLFKTAELSSFLLQESLQLLLQHATNNNMNPPPSSSSVPFSLIFKIGAYKSFSEISPVSPFANFTCNQALLEALEGFDRIRIVDFDIGYGGQWASLMQELALRSGGSPSLKITVLASPAMHDQLELGLTRENLIHFASEINMAFEFEVLSIDSLNSTSWSLPLHVSDNEAIAVNLPDRVCDRTDLLFPNHVIHALQYYANLLESLDAVNVNFDALQKIERFLLHPGIEKIIMGRYRSPEKTQHWRTLFLSSGFSPLTFSNFTESQAECVVKRTPVRGFHVEKRQSSLVLCWQRKELISVSAWRDSIPMEAFIQVSSVLNDNRRSLFLSLPSA

>IbGRAS20

MASISFPLAGKHKQGTWFGVEGSEDYDSFFSCLYGLSPQDSRADSKIDDDQKQSIQVSQPAEKSVQNEQQPTPFSLACLEVLKTYDKLFNKPTDEGKGVNHNNKYNASSCSCTCGKLSVNEVLKLGGERYIQYITPRADGFSMFMHPYASALSALTAHETRDVELVHLLLAAAEDVSRRQYDVAANLISRCMWTASHSGNPVQRLVFYFAEALKERIDRETTGRLFTAARYHYQEYYCMGLTTTPATLACHQGLPFSQVMQFAGIQTIIENVNTTKIHLLDFNIRSGIQWTILMQALAEEHHDRPIQLIKITAVGVADQRKLEECGNRLESFARSLNLPFAFHLVFLSDLKDFREDLVHLEADESVAVYANTVFRTMIGRPDCLDSLILAIRKLKPVVMVVAEVEANHNSPSFITRFIEALFFYGAFFDCFEDCMERKDPCRRTIEGIHFGEGIINIVAAEGEERFTRNVKIDVWRAFFARLGMLEIKLSECSMYQAKLILKQFEHGSSCNLYSNGKGLIVGWKGTPIHSVTCWKFNSHDDY

>IbGRAS21

MEALVQEFYANTNGFMLNRHSKPANPKLNGQKRRDLDSFQDFPAAGGGEEEDEDYSDAVLKYINQMLMEEEDLENRPCMLHDCIALQATEKYFSDVLHGSDHADLSSSNASSDPNDYGASSDCLLSTPDSQSGSVSPGSDGYFPAPNLVLPHSNAINLELEDRSPPKGKRNHYSNKEDESEEKQRNKQLATSTHETEPPLEKFNEVLLCNIQEPRKKSEDEFKGVAAPRRRKKRESHKEVVDLRGLLTQCAQAIANYDGRAVNELLAKIRHHSSPRGNGMERLAFYLANALEARLNGAGTAIFTVQFSNNISAANILKAYHMYIKASPFKKISNIYANHYIMKMAAGKHALHVIDFGVLYGFQWPCMIQSLANRPGGPPKLRITGIDLPQPGFKPAERVEATGARLKKFCEQFNVPFEFKAIAKRWETITLEDLEIDRDEILAVNCLYRLENVPDETVVPDSPRDAVLGLINKIRPDIFIHGVGNGAYNSPFFTTRFREAVFHFSTLFDMFEATVAPEDEDRRLFEETVLGRNALNVIACEGTARVERPETYKQWKGRNKRAGFRQVPLDQELVKLVKDKARSDYHKDFSVDGDGKWLLQGWKGRVVYALSCWKPAME

>IbGRAS22

MAMDHQFSRLTDSVNRFLLEDENIFSSLKRSPDIPGSYNVDSLPLDIVDAPLVIHDPNLGSYAPTLDYPDDHDSDTVLKYLNQILLEENIDENPSMFYDPIALKAAENSFYEALKEKPPSPHQAPLFVNSNARSPDSILPSSGGYSTSSSSIGSCNADPQWIVDPGESKSSVTSYSPEFSFQSSSQANSYRLNGSLNSFSSVMNAQTDSFVNANLVPNIFSDTESILQFKRGMEEASRFLPTGNQLVIDLDKYSLPPKTDELSGDAVIKIEKDEKDRSANSSRGKKHHHPDDSGLEEEERSSKHSAVYEEEVELSEVFDRVLLCTDNYGCNINVEGKQQNGASGGKGRTKKQGGNRETVDLRSLLSSCAQSIAAADYRTANDQLKKIRQHSSPTGDPNQRLASVFANGLEAWPILIQHLSQRPGGPPKLRVTGIELPHPGFRPAEKVEQTGRRLANYCERFGVPFEYNAIASQNWETIKIDDLKLASGDVVAVNCLFRFKNLLDETAVADSPRDAVLSLIRKINPDIYVQARLDFEQEFIGREIMNVVACEGMERLERPETYKQWQVRNMRAGFKPLPVKPELVKKLRGKVKAGYHKDFVFDEDGHWILQGWKGRIMCGSSCWVPA

>IbGRAS23

MESIDEEDEFLSLKLAIATQQPLGHERNKKRKKREDLVDILSYEEEVYSLLQIGEQMLNSTHKTSKESLGEGLEGLHLIHLLLVAAAAVNDNNLTSAMANLSELCQNVSPPNPQEEFLAFTHLYKVSPLCQFAHFTANQAIIEAFDNTNDASLHVIDFDISYGFQWPSLIQSLSQPLLNRVSLRITGFATTLNELRETEARLLSFAKGFRNLSFEFHGLLKGSHLGNIVTRENETTAVNLSFRVNRLLTDNISETLKAVRSLRPSIVTVVEHDVCRKLPRSFLPRFMESLHYFAAMFDSLDDCLPVESHERLSIENHLGREIKSVMNFDDQRNDEREVMEMWKGRGVLENCGFCEMELSSKNVMQAKLLLKIRSHSPSPSSDSSCSSSCVNGGFRVVERDDGKGISLAWQDRCLITASAWQCV

>IbGRAS24

MMMADEILPDFDFCGYSGYSTTTTTTTSSSDGDHAATWNGSLPLVDWGFFSGDDEFGDLIDSMMEGQAGNTTVGLSFPAIPTVVEVENEDQDEDEEYNMVEDTKGLRLVHLLMAAAEALTGLNKSRELARVILVRLKELASPRDGSNMERLAAYFTDALQALLDGAGSLQAKSFPASHEHSHADALAAFQLLQDMSPYVKFGHFTANQAILEAVTHDRRVHIVDYDIMEGIQWASLMQALISREDGLPAPHLRITALSRGGGGSSRRSFCSIQETGRRLTAFAASIGQPFSFHQCRLDSDEAFKPSALKLVRGEALIINCMLHLPHFSYRSPDSIASFLSGAATLNPRLVTLVEEAAPAPPPDGGFVDRFMDTLHHYSALYDSLEAGFPMQDQARTLVERVFLGPKISVAITRSYRSRGEEYGWSWGEWVPENAGFRPTTISFANRCQAKLLLGLFNDGYRVEEIGNHKLVLGWKSRRLLSASVWTSPDSDL

>IbGRAS25

MIGMQYSNNSLQGKGEVGRFGSLISASSSAAQDGKLKRDGSFGSNEPISVLDKRRSPSPSTSTSASSSSFGCTAAVKDAPAPAAVEEWVVGELQPLVPFEKFGLGLEDWEYFLSESGAGVGGSDQSILRWVSGAEFEDPSSLHEIQGNAGLGDAADQTTTGFGALIASDNFFTNVSSSVIPISSLNSNIGKFGSTVNHVNSQDSNLNFTPNNNLVPGLSFQEPEQKPQISNFQIPVVNQTQNATNRNVFVSPSYGGILHEEQLPPPAKRQNSEISNSQLPEIPLVGLSHGLLLGKQQDFAQLQHQGMMGSGQHSSLLVPKQEEVVMPHHQHQQQVVYDQIYKAAELILTGQFSHAQMILARLNHQLSPVGKSLQRAASYFKEALMLPLLMPGSSISLPSRVPSPVDFVFKMGAYKVFSEASPILQFMNFTSNQALLEALGDAEYVHIFDFDIGFGAQWSSFIQELPKRNNGGRGGAPSLKITAFASPSTHHPVEISLMHESLTQFANDVGVKFELEVVNLDTFDPSSYQLSSFRPCGSEVVAVNFPIWSLSNHLSALPSLLHYIKQLSPKIVVSLERGCERTELPFPHHILNALKYYEVLFESMGAAKVTPDMANKMERFLFQPSIESIVQGRLCFPDQMSPWRTLFTSAGFLPMPFSNFTETQGECIMKRNQVRGFHVEKRQASLVLCWQRRELLTAMAWRC

>IbGRAS26

MLAGCSSTLVSPRHRLRSEASEQFQACHFPAMSTQRLDLPCSFIRKESSRAQAVRPVGLSVEKPGEAKTSGCGLKQNIRLPPTPTAIQTPRFEGKRESWEWEKKSRCLKRYAAEQESCDEGFVSRANKRKKGCGEEEEDEEEEQKGHHGLSLGHLGSSSGGFWFQSGVGGANPNPSSQGPFSLSSSGGDEESVCFVPSEVRPPPLPLSHHPWLDSVVTEITDFSDKNVVETSQGPAKEASGSSSSSEGGGRGESEPNNGVELITLLVACVEAVGLKNFAAVNHCIGRLGELASPRGLPVSRLTAYFTEALALRVAWHWPHIFHITPPRDLDRPGLDDDHNNGTALRLLNQVTPIPKFIQFTSNEILLRVFEGKDRVHIIDFDIKQGLQWPSLFQSLASRANPPSHIRITGIGESKQELLETGDRLAGFAEQLNLAFEFHPVVDRLEDVRLWMLHVKEGESVAVNCMLQMHKVLYDTSGRALVDFLGLIRSTNPIAVVMAEQEAEHDESSLESRLVNSLKYYSAVFDSLDSSLPLDSPVRTKIEEMFAREIRNIIACEGRERLERHASFGKWRKLLMEQGNFRCVGITERELLQSQMLLKMHSMESYKVEKQGEDDGLTLSWEDQPLYTVSAWTPFDVAGSSSSYSQPS

>IbGRAS27

MAVSPAMLFAADPFQVPAVESGFQFPSLDNQLGAFRFPDFGGGGGEFDSDEWMEGLMDGGDSTASSNLHSGCDTWHNNSDFTALYSADPFSACPSRLRIASSSPSALNSVIFTDNQKNQNHAPPPPQTLPWFPTPPPPSAKDSKEAAPRNDVVAKGSSPESLSSKPLLKALVDCARLADSQPENAVKSLIRIRDSVSQLGDPTERVAYYFSEALYNRLSNSPEKRPANFEACSEELTLSYKALNDACPYSKFAHLTANQAILEATEKASKIHIIDFGIVQGIQWAALLQALATRSGGKPESIRISGIPSPVLGNSPAASLLATGNRLRDFAKVLDLNFEFEPVLAPIHSLNGSSFRVDPDEILAVNFMLQLYNLLDETTATVGTALKLANSLNPSIVTLGEYEMSLNRVGFLKRFENALKYYSAIFESLDPNMTRDSPERVKVESLLLGRRIAELVRPEEQGAKTECVEDKDHWRILMESAGFKAVAISHYAHSQAKILLWNYNYSSSYSLIDSPPGFLSLAWNDEPLLTVSSWH

>IbGRAS28

MKELGLQDERKPDNHHNLLFAPSESQTHHHSELTHSLEFVPSDFNFSSQFQGNENISSFDENWSVGFDFIDELIQFAECFDTNAVQLAQVILPQLNQKLSSGAGNPLRRAAFYFKEALQSLLTGSTRSGSSSFEVIQTIKAYKIFSNISPIPMFSSFTANQAMLEAVDGAMLVHVIDFDIGFGSHWASFMKELADKAESTHTKPPILRVTALVPEEYAVESRLIRENLAQFARELNIGLDVDFVLIRTFELLSFKAIKFIDGEKIVVLLSPAIFRRVGSVQFAADLRRVSPHVVVHVDSEGAAANVVGGGDWVKKIENFVLFPKIVEMVRAAGAGAGGMTWKEAFVAAGFRPVGLSQFADFQANFLLGRVQIGGFHVAKRHAEMLLCWHDRALVATSAWR

>IbGRAS29

MELGLGANPCPFFPDPCVSNRLNFEDVYQDRELVDGPRADNSLGVHSFEGFHDPLSRNVALTSHQDDYEDDDFSDADLRYINQILMEEEMEDKTFMLQESLELQAKERSFYEALGKKYPPTPEQNPTLLDQSSLSRGNYEVENHQNCITNSGRGSSSVVNQSSGSLGEYESENHHNYYYITSSNEGSSYLIDPGSINVTGDYISPYLHGFSVPNGSNSSVRSLNSFNNRVDGFVESPEPTRETGEVEGTGEKSLRVRKNPHREDLEDQRSSKQAAIYTESTIRSEEFDIVLLHSMGKGEEALTAYRQNLQSAICKNIQQNGNSKGPGGGKGRGKKKNGKRDVIDLRTLLIHCAQAVAADDRRSANELLKQIRQHSSPFGDGSQRLANCFADGLEARLAGTGSQIYKALVNKRTSAADYLKAYHLYLASCPFRKISCFASNKTTIIKSANSMRVHIIDFGILYGFQWPTFIQRIAARDGGPPKVRITGIEFPQPGFRPAERIEETGRRLADYAESFNVPFEYNAIAKKWETITLEDLKLDKDDFLVVNCLYRFKNLHDETVLAEGSRTLVLNLIRKINPDIFIHGIVNGAYSAPFFVTRFREALFHFSALFDMLETNVPRDVPERMLIEREIFGREALNIMACEGWERVERPETYKQWQVRNLRAGFTQIPFANLIMNKARDKVRTGYHKDFVIDEDGQWLLLGWKGRTIYAISCWVPV

>IbGRAS30

MMQPQLLHQPSWPSSYNVDYSTLPRTRLCGLHEDSYVRDNYTHSSLLTTTDSFGVSSVSDLTALFPDAFTELSSLQLQNDMQILPPLPDGEFEDVCKWLNTNDSEDGISSEASFSIAQSSDANRLPAIFPGSGVEVDTQLSLHHLLGAYAEAMENGHEELAEVIVKRIRGKASPLGETLERVAHSLFESTEEDQEGYLRQESSKNFEQAFRAFYQILPFGRFAHFAANSAILEALPDDAETVVHIIDFDMGEGVQWPPVIEAMARKRRALNLRLTSIKPNHESTSNQYEETKRRRYDHAKPFALNLQIEEMSVEELAIETKTKKNKGPGKQWLAFNCMFRLPHMATNMPQTTQAMEFLKIAKQLLAHSETQTGIIIFAHGESEGCNTPTSNYTFFL

>IbGRAS31

MVMERNVRVICEASGDKLKGKTQSVFLDPNLINSLNISETLADSNGMTSNLCDEGLPNFLDPTVIDSLRISHTLVDRNRGIENLYGESISKFSDTVLIENSRGEQSLHIGNSCHQGIPSFPDPILVGSLNVSQTCLDQNELAGNLNGQAVSDFLDPVLIENLRISQTSVNQNGLTGVLNDDGPPCVMDLHLINSLGVCEDLLDQNQFAFPQLQSDPRLNVVAPSNEGDAHEDFDFNDGDLNYIGQMLMEENMEEKVCEKYPPPAGHYTVSNSDQNGILVDGNYHNSIKNANSRLLCPKMDPYPSESDIYSAQCIQVSVPLQTISQSSYSTSSSSGTVNDGHVDSPVSLGYNGKKRSAEKNEVVVNMEKRYENQHSREISKAKKNLHNEDLEALEGRSNKQSAALQIVSIKNATDNDDSKASNGGKKSRRKKQRGKRDTVDLRTLLTLCAEAVVADDRRNASEFLKQLRQHSSQTGDGMQRLAHYFADGLEARMAGSGTQIYKALITKPTSAADILKAYQLFLAICPFRKISNFFSNKTTMNLAQSATSVHVIDFGILYGFQWPCFIQRLSSRKGGPPRLRITGIDLPQPGFRPAERVEETGKRMANYAERFNVPFEFNAIAKKWETIKIEDIKINKDEVLVVNCLFRLRNLLDETVVVNSPRDIVLKLIRELNPHVFIQGIVNGAYNSPFFITRFREALFHFSSLFDMLDTNVPRNIHERILIEKTIFGQEAKNVIACETAERVERPETYKQWHVRNMRAGFLPLPLNKEIMKMSRDRAKVYNKDFVIDEDGEWLLQGWKGRIVYALSSWRPAS

>IbGRAS32

MSSGFPGSVQEFYGGPDGISNGRSVPVGSNIGNLMQQGGVQVQVPYGSQLPGIVSDSASQIAHRRSDLIGKRSLVEFQQQQQQLQFLQQQRQGALGLYLRNVKPRTYQHSSPISPLSPVDLSALSSISSNSNSPAMNARYGVPILQQFRPQLSMPAGSVNINGVLPSGPGNPNYAPGFSFPNSVQNRGGLGSERAGLETEKKMMNRLQELEKQLLDDIDEEEGDTVSAVTNSEWSETIQSLISPAQTQTQTQGPNQNNNKPQISPSPTSSTSSCASSMECPAITCPKQTISEAATAIAEGKNEVAAEILTRLAQVANVNGTAEQRLTAYMTSALRSRVSPVEYPPPVSELLTKEHELSIQKLYEASPCFKLGFMAANLAILDAVSDQRFCKLHVIDFDIGEGGQYLHLLYALAARKAENPTVLKITTFADVPGGDQQLRAVEEELQKQAQTAGVCLSINIIPCSNTELSRERLSVDPDEALVVNFAFNLYKLPDESVTTENRRDELLRRVKALSPKVVTVVEQELNGNTAPFVARVNEACGYYGALFDSLYLTVSPENIYRVRIEEGLGRKMGNSVACEGRDRVERCEVLGKWRARLSMAGFTAIPMSQHVADSLRSKLNSGPRGNPGFTISEQAGGIGFGWKGRTLAVASAWR

>IbGRAS33

MCWRKVGVRGSYAEAKCILVEGLRARILSSGSIIYKKLKCKEPTSSELLSYMQNRIHIIDFQVAQGSQWMFLIKSLADRPGGPPFSLRITGVDDSQPCSGGGLQLVGERLANLQSHVVCPLNSMQQVFLALRLNSRTSGFNPRSSCVNFPYMLHHMPDECEYHESQRPLIETGKESVPKIVTLSTRIQHQHHSFLPRNIARISIAEGHGDCILDGRTAWHQPGDDTPSTHFQLSLFAYPSIFNLFALLGMIRTEFFL

>IbGRAS34

MQASQGPQSSSSVQRFYHQPQQQVEQYYATFHVLNNNASNDSGSVGEQGFFQTQNEQFFTLDSAPAAIDSVYYDSPPAASVSSNRSAFSPQCSQSYMSDMHHSSDNTTCGSPLSGCSGVVDGNELRHVLRELENKLLGPESEIDDNYSCSFSDAVPKSSSMMKWKRMLDIAPSLDTKELLFACAEAVSDADISTAEVLMNVLEKRVSVSGDPMQRLSAYLLEGLRARILSSGSIIYKKLKCKEPTSSELLSYMQVAQGSQWMFLIKSLADRPGGPPFSLRITGVDDSQSAHARGGGLQLVGERLAKFAESCGVPFEFHAAGISGSEVELENLWIQPGEAVARQLIETGKSLSPKIVTLVEQESNTNTTPFLPRFRETLDYYTAMFESIDAACPRDDRQRISAEEHCVARDIVNIIACEGADRVERHEPFGKWSMRFTMAGFTPCPLSPSVGEAMRHMLQEYSPNFGIAEGHGALYLGWKNRALASSSAWR

>IbGRAS35

MMQSVPFQPSWPSHNLQFSTSPQTIFCGLYDETYIHSSTAITTDSSDSPLYSSFAALFPDAFTELTSLQNDTAMDRLESIEFEDFCEWLNNSDSEEQPKGDMWSPSLSVVSSEASMVLPSRNTAVTVPGTGMEVEGLTNLHHLLEAYAEATEDGHEELAEVIAKCIAGKVNPLGEPIERVAFNLFHPSEDLLKESSNNFEAAFMAFYQILPYGRFAHFAANSAILEALPSSAETVTIVDFDMGEGIQWPPLIEAMGPKPISLKLISIKTEQEPTSSSRWRFETTQRRLYDHARQCGQKLQVEEMTIEELVTETKRNTGKQWLAFNCMFRLPHMAKKQPRSQAMEFLKIAKELLSSSGIVVFADGTPGQWRFLSWLHFFL

>IbGRAS36

MVMDRNFGDFYQDTFGGKSGDESLQFFLDQEAISGLSVNDPYMDVNVGKNSKDGGESNVLDPNLVSNGSVGETVVGQNQITENVDAVTPPSIPYPNSNNGLGIGGGSLEDRSELAFPPLQSDLSLDVVAPSSEGDGHEDYDFSDVVLKYISQMLMEEEMGEKACMFQESAALQAAEKSLYEVIGEEYPPNSLDQNGNNGDGNNGDSGLVYPNWDPDPSESENSTGQHAPVGVTLRTNSQSSYSSSSSSGTVNDGHLDSPVSTLRIPDVHVDSPLSTLRIPEIFNSTESIMHGLGKGQSTDGIHGKDMAVKDGKNNENQLSREGSRRKKNPHYEDEDLDEGRSHKQSAVSSESTVKLEMFDKVLLCSGGKNESALRQSWQTVSSKNAMDNDLPKGSNGKKSRGKKQGGKSEVVDLRTLLTLCAQAVAADDRRTAHEFLKQIRQHSSQTGDGMQRVAHYFADGLEARMAGSGTQIYKALITMPTSAADVLKAYQLYLAACPFRKISNFFSNKTIMNVAKDATSVHIIDFGILYGFQWPCFIQRLSCRPGGPPKLRITGIDFPQPGFRPAERVEETGRRLANYAERFNVPFEFNAIAQKWETVKIEDLGINGDEVLVVNCLYRFRNLLDETVLVDSPRDIVLSLIRKLNPAVFITGCANIPREIHERMLLEKTIFGREAMNVIACEGAERIERPEIYRQCQVRHMRAGFRQLPLNDEIMQMSRDRVKAYHKDFIIDQDGKWLLQGWKGRVIYALSTWKAAY

>IbGRAS37

MSPHDSSITSSSGSSSSSCSAAHVDQIDSLLAGAGYMVRSSDLRHVAQRLEHLESFMVNSPMGSEISQHLGNDVVHQNPSDLGSWVDSLLSELHPPPVPEFAAPCPSDSNYVAAGPTGWSECEAMQQQPQIVSPSHLTVVTATEQEDSGIRLVHALMTCAVSVQRGEFSLAGSLIDELQLLLTRVNSGCGIGKVAGYFIDALSKRLYTPQGVSLMGSAYEDEILYSHFYEAAPYLKFAHFTANQAILEAFHGHDCVHVIDFNLMHGLQWPALIQALALRPGGPPLLRLTGIGPPSPDGRDSLREIGLKLAELARSVNVRFAFRGVAASRLDDVKSWMLQVGSNEAVAVNSIMQLHKLLGPDPIRGSPIDTVLGWIRSLNPKVVTVVEQEANHNQPEFLDRFTESLYYYSTMFDSLEACPAQPEKALAEMYIQREICNVVCCEGVARVERHEPLGKWKARLTGAGFKPLNLGSNAFKQASMLLTLFSAEGYNVEETEGCLTLGWHSRPLIAASAWQASRD

>IbGRAS38

MMQFTETLPAPSHQISPFSSLVTNKNQVHRTRPWPGFPTSSKNLGTSIGDANCMEQLLVHCANAIESNDATLAQQILWVLNNIATPDGDSNQRLTSAFLRALIARAAMSGTCKLLAAMADLHPGLNLNMMDTHRFSIIELASFVDLTPWYRFGFTAANAAIIDAVEGYSVVHIVDLSSTHCMQIPTLIDAIAARPEGPPLIKLTLAAAADEFPPMLDLSYEDLGAKLVNFARSRNVELNFTVVPSTPSDGFSSLIQQLRLQNLVRAENGEALVINCHMMLHYIPDETLYENSHSQSQSAASSFRTMFLKSIRSLVPTILLLIDEDADFTSNNLVCRLRSAFNYLWIPYDTVDTFLPRGSKQRQWYEANICWKVENVIAQEGVQRVERLEPKTRWVQRLRNANFRGVPFSDDGVSEVKNMLDEHAAGWGLKREDDDVVLTWKGHNVVFATAWVPN

>IbGRAS39

MDTLFRLVSLQQSDQSFNSSRTSSSSRSSRHNDNANANNNYHHHQEDEECFNFFMDEDDFSSSSSHNKHPYPPSSSSYHHQYQHFSNTPTPTTTSSTPPHSYSHHHQPPTFDPNLEFGSDFSGKWATEILVETARAVADKNSGRVQQLMWMLNELSSPYGDTDQKLAAYFLQALFSRMTDTGDRTYRSLVSASDKTCSFESTRKTVLKFQEVSPWTTFGHVACNGAIMEAFEGENKLHIVDISNTFCTQWPTLLEALATRTDETPHLRLTTVVVNKAFGGAGGGGAASIQKVMKEIGNRMEKFGRLMGVPFKFNVIHHSGDLSDLDLSALDIKEDEALAINSVGALHSVTAVGSRRDYLISVFRRLQPRILTVVEEEANVDVGVDGSDFVRDFQECLRWFRVYFESLDESFSKTSNERLMLERQAGRAIVDLVACPPSQSIERRETAERWSRRLHAGGFAPISYSDEVCDDVRALLRRYREGWTMAQCSGDSSAGIFLSWKDQPVVWASAWRP

>IbGRAS40

MKVSFTSNENVSSKTSTLSSCGSNIGIQAPPFPAAATAAARVVSYEPKSVLELRRSPSPNVTENAHNPALDAADISAGCDDPLQLADHVLTNFEDWDSLMKDLGLKEDTTKPNSESLQTQFPSSRSLTRSIPLSSSRLSISASPITSRRRSTRLRRSAPGNSNFSLSGNDFQNHNWNLGFDYVDELIRFAECFETNAVQLAHVILARLNHKLRSATGKPLQRLPSTSRNLSSLYSPASRLTKPCWNRLYACPRHRLRHRARRPLGFVHERASRESRVGSRQPALRITALAIKFMEGENRGDFISVDIQAGRGGVRERSPRRISPHVVVHVDNEGLVGFGPSSFRQTVIDGLEFYSTLLESLEAANIGGGSGGWRLDEEDRDGAGKRIPRGEEAGGDAALLAR

>IbGRAS41

MKVSFTSNENVSSKTSTLSSCGSNISIQAPPFPAAATAAAGVVSYEPKSVLELRRSPSPNVTENAHNPALDAADISAGCDDPLQLADHVLTNFEDWDSLMKDLGLKEDTTKPNSESLQTQFPEFPLAHSLDSAQFLPSEHFSFSDNVTAAQYPPPPLSAGGNSNFSLSGNDFQNHNWNLGFDYVDELIRFAECFETNAVQLAHVILARLNHKLRSATGKPLQRAAFYFKESLQSLLTWSTRMTRPNSSSEIVHTIKAYKIFSNISPIPMFSSFTANQAVLEAVEGSMLVHVIDFDIGLGGHWASFMKELAEKAESARAKPALRITALVPDEYAVESRLIRENLTQFARDLNMAFDIDFVLIHTFELLSFKAIKFMEGEKIAVILSPSIFRRVGAGFVNDLRRISPHVVVHVDNEGLVGFGPSSFRQTVIDGLEFYSTLLESLEAANIGGGSGGGDWMRKIETYVLYPKIMEMVGAAGRRGSSWREAFIAAGFRPVVLSQFADFQADCLLGRVQVRGFHVAKRQAEMLLCWHDRALVATSAWR

>IbGRAS42

MKVSFTSNENVSSKTSTLSSCGSNISIQAPPFPAAATAAAGVVSYEPKSVLELRRSPSPNVTENAHNPALDAADISAGCDDPLQLADHVLTNFEDWDSLMKDLGLKEDTTKPNSESLQTQFPEFPLAHSLDSAQFLPSEHFSFSDNVTAAQYPPPPLSAGGNSNFSLSGNDFQNHNWNLGFDYVDELIRFAECFETNAVQLAHVILARLNHKLRSATGKPLQRAAFYFKESLQSLLTWSTRMTRPNSSSEIVHTIKAYKIFSNISPIPMFSSFTANQAVLEAVEGSMLVHVIDFDIGLGGHWASFMKELAEKAESARAKPALRITALVPDEYAVESRLIRENLTQFARDLNMAFDIDFVLIHTFELLSFKAIKFMEGEKIAVILSPSIFRRVGAGFVNDLRRISPHVVVHVDNEGLVGFGPSSFRQTVIDGLEFYSTLLESLEAANIGGGSGGGDWMRKIETYVLYPKIMEMVGAAGRRGSSWREAFIAAGFRPVVLSQFADFQADCLLGRVQVRGFHVAKRQAEMLLCWHDRALVATSAWR

>IbGRAS43

MAYMCTDSGNLMAIAQQVIKQKQQQEQQQQQQQQQQQILGVNSFCLNPWQTPHPGLSGGPSLGYGLGGAAFADPFQVGGAGGDGAEAGFQFPSLEQHGGGVFPFADFGGGAGGEFDSDEWMESLIGGGDSTGSSNLHSGCDAWQTGSEFGLYGSDPFAGPSRLSIASSAPSNLIFSEAQKNNNINTCPLQPQTSEWAPTSTSPPAQQTSPTHRNDVVAAAAGTSFSSPDNLSSKPLLKALVDCARLAESEPDNATKSLVRLRDSVSQDGDPTERVAYYFSEALYSRLSRQPAKIPFVEASLEEFALSYKAFYDACPYSKFAHLTANQAILEATEKASRIHIVDFGIVQGIQWAALLQALATRPAGKPKYIRISGIPGPFDPVLTPIPELNESSLRVDPDDALAVNFMLQLYNLLDETTATVEAALKLAKSLNPSVVTLGEYELSLNRVGFLERFTNALNYYSLVFESLDPNMPRDSPERLQVERLLLGRRIAVMVGPAEQETKRECTEDKEQWKILMETAGFEPLPLSHYAMSQAKILLWNYSYSASYGLIESPPGFLSLAWNDEPLLTVSSWH

>IbGRAS44

MKRERDRPKAGSSSIGKAKIWEDQPPDAGMDELLAMLGYKVKTSDMADVAEKLEQLEMAMTMTMGTAKEDGISHLSTSTVHYNPSDLTGWIQSMLSELHTSDALSQSSVGDEMMLAGESSNIISFSGNKNIGNGNRRISDDDLRAIPAGLFSVIRGKIPHTPRSVLVDSQEAGVRLVHALMACAEAVQQENFKLADALVKHIGILAVSQAGAMRKVATYFAEALARRIYKIYPQDTLESSYTDVLQMHFYETCPYLKFAHFTANQAILEAFNDCSRVHVIDFSLKQGMQWPALMQALALRPGGPPAFRLTGIGPPQPDNSDALQEVGWKLAQLAETIGVEFEFRGFVANSLADLDASILDIRPSHVEAVAVNSVFELHRLLSRTGGIEKVLNSIKSMKPKIVTIVEQEANHNGVVFLDRFNEALHYYSTMFDSLESSGLTQPNSQDLVMSELYLGRQICNVVACEGPDRIERHETLSQWRARMKSAGFDPVHLGSNAYKQASMLLALFAGGDGYAVEENDGCLMLGWHTRSLIATSAWQLGGGGGGEP

>IbGRAS45

MGGAESELLQPVPVLLEISGGSSAAAPEEQEQEPLLRWITGDADDPSMANLSKLLQGGDQAEYEFNASLGLQDHNFGADPDSSSSGSAFLPTSSSPANLQTPSFLSFLDSSDMKPQHLQSPSFFMPLPYYQSQDQSIQPAKRHNPGTLGVPETIDHLFKTAELIQAGNSVLSQEILARLNHHLSPTGKPFHRAAFYFKEALQSLLLPHATKPTVLSSPFSLLVFKIGAYKSFSEVSPMPQFANFTTNQALLEALEGFHRIHIVDFDIGYGEQWASLMQELALKNSSKPSLKISALASPPLKQEQLELGIIRDNLIQFAAEINMGFEFETLGVDHLNSSLWSDSDSEAAIAVNLPVGWLSRNQQVSLPLVLSFVKQLQPKIVVSVERGCDRTDLPFPDHIIHALHSCSNLLESLDAVNMNPDALQKIERFLIQPGIEKTITARFDSPEKTQTEHWRTLFLSSGFTPFSFSNFAESQAEGVLKRTPVGGFHVEKRQSSLVLCWQRKELISASAWRC

>IbGRAS46

MKADLRGNPTSFLFQNPNLFVASSQPSSDLTGALRGCSSLGSLDGACTEKLLLHCASALEANDVTLAQQVMWVLNNVASPTGDPNQRLTSWFLRALVSRASRVCPNAPVTGFGGGNGVHTRLMTVTELAGYVDVIPWHRFGFCAANSEILRAVQGQNRVHILDFSITHCMQWPTLIDALAKRPEGPPFLRISVMSGRPSVPPLLNASCEQLGVRLANFAKFRDVPFEFNVIGSESDYIHQEFPDFHHDFILSCLTPSCLNLRPDEVLAVNCQNWLRYLPSRDTFIDAIKGLNPSIVTIVDEDCDLGGSSLVSRITTCFNYLWIPFDALETFLPKDSPQRIEYEADIGHKIENIIGFEGDQRIERLESGMKLSQRMKNNGFLSAPFCEETIREVKCLLDEHASGWGMKVEDEMLVLTWKGHSAVYATTWVVSPQTQVDIEIKD

>IbGRAS47

MQASQRLRTSSMSNTMYYQPVQKAEPYCLPQFQNLDHQPSYTNSINGGNHAIHPHCASYYTEFSLMSRAHGLHNSSSTLSFSPNGNSVSQQESQAYPSGLHQSPATTYSYPIRESCFGDEVNDLDFKDKLRELETVMLGPGSDIVESYANAIVSPEIDSWEQMIVAIPRGDLKQLLVACAKAVSDNDLLTAQSLMSELWQMVSVSGEPIQRLGAYMLEGLVARLPVSETSIYKSPRCKKPSSFELLSSMHILNEVCPYFKFGYMSANGAIAEAMKDEKRVHIIDFQIGQGSQWVTLIQAFAARPGGPPEIRITGIDDSTSAYARRGGLSIVGKNLSNLAESFKVPFQFHAVTMPGCKAQLENLTIQRGEALAVNFAFMLHRMPDESVSTSTDNHRDQLLRLVKSLNPKVVTLVEHESQSNSPAFYPRFLEALDYYSAMFESLDMTLPRNHKQRINVEQHCLGRDVVNIIACEGTERVKRHELLDKWKSRFRMAGFSPYPLSSLVNGTIKTLLENYSDKYRLEERDGALYLGWRNRDLVASCAWK

>IbGRAS48

MSPGLISDELAYSSNPYATILKRNADSCIPVFCDDEMLESKRPKRAPSIGDCLGSNEIRNSLSRVHFRDHVLAYSQRFLAAEAVELQAADEMIGSDNGESADGMKLVQLLISCAEAVACRDKSRASVLLSELRSSALVFGTSFQRVASCFVQGLADRLALVQPLGTVGYLTPAAALDAAAGDAASEKDEALRLVYEICPHIQFGHYIANASIVEALEGESFIHVVDLGLSLGLPHGHQWRHLIQRLASSNRPLRRLRITAVGTLLHRFRSIGEELKDYASGHGINLEFSIVESNLETLKASDIKIVDGEVLAVNSVLQLHRVVKESRGALNSVLQIIHELSPKILVEQDSGHNGPFFLGRFMEALHYYSAIFDSLDAMPKIRHEESEDRAVLLRGGD

>IbGRAS49

MASKRSVIDLGGAAAAETAAAAVEDSSFKRPRHLSSSGDPSVSGEKGEEEEEEGVMDTESTGLRLLGLLLQCAECVAMDNLDDASNLLPEIAELSSPFGSSAERVAAYFAEALSARIISSYLGSYSPLTLKTLTLTHSQKLFAALQSYNSISPLVKFSHFTANQAIFQALDGEDHVHVIDLDIMQGLQWPGLFHILASRSRKIRSIKITGVGSSIELLESTGRRLAEFATSLGLPFEFRPLEGKIGSITDPSQLGVKLGETTVVHWMHHCLYDVTGSDFGTLRLLTLLRPKLITIVEQDLSHGGSFLGRFVEALHYYSALFDALGDGLGADSVERHTVEQQLFGCEIRNIVAVGGPKRTGEVKVERWGEELKRVGFGPVSLAGSPAAQAGLLLGMFPWKGYTLVEESGCLKLGWKDLSLLTASAWQPSD

>IbGRAS50

MGQVVRGNPTFQNLATFLNNNPQTSLSSAGAIGGCLLGSLDGACTERLLLHCASALESNDVTLAQQVMWVLNNVASSTGDANQRLTSWFLRALVSRAARVYPKSYPTCGGGNGACGRVMSVTELAGYVDLIPWYRFGFCAANSAIIEAVQGCAKVHILDFSVTHCMQWPTLIDALADRPEGPPALRLSVPSWRPPVAPLLSLSTEEVGLRLANFAKSRSIPFQFSVIADINNGGLGLFEDADEALVVNCQNWLRYLPCRQTFLDTIKCLNPTLITVIDEDADLDAPSLSSRIVNCFNYLWIIFDSLETFLSKDSQQRAEYEADVGQKIENIIGFEGGQRIERLESSTTLSTRMRNSGFLNVPFSEETIKEVKFVLDEHASGWGDEEER

>IbGRAS51

MAELWGFREGLILAKDQGFEKVAVETDSETMIHDIKILHVLREGNQCADFLANLGQESPWGTTVLDRPPDNQQRTEQKGTQHRTVILLKHKKWCSLATSKLGNPELVKGEVWCRENGAPAMVMIGMQYNSNSLQGKSVLEVSRFGSAISPSLVPSDAAKLKKNGGFSSSEPVSVLDTRSPSPSTSTSAMEANPNPVGRKEKSLGELHPAPEWSEGPEKFDLGMEDWEGLLPESGGSDQFLLRWISGDVEDPSLSLKQLLQGGNPSEIHCNVGFGASDSDNYNGNSQGPNVNFPLAILGSGLSVQELQNEQKPQNFNSPVLTELQNVVTIPNVFSSELYGIQQEQPPLKRQNLGGVSSSGFRVSEGGVFVNPINGLLVRKQVELGQLEQAQIGCDRPPDILCVHRSEQAPLLVPTREAGFGNDSLQEQQAVYDHVFKAAEVILTGDFLHAEMILARLNHQLSPGVKPLTRAAFYFKEALHLPLTMPISATSLPPRIPTPVDGVFKMGAYKVFSEVSPLIQFMNFTSNQAILEAVDGAECIHIFDFDIAFGAQWSSFMQELPRRNRRAPSLKITAFASPSTHHPIEINLMHENLTQFANEVGVKFELEVVNFDSFDPSSYPVSSFRSSESEPKVVVSLDRGYERTELPFPHHLLNALQYFEALFKSIDAGNVTPEASNKIERFLFQPSIESIVFGHLRFPDQMPPWRNLFASAGFLPQPFSNFAETQAECVVKRTETRGFHVQKHHASLALCWQNRELLSATAWRC

>IbGRAS52

MQSNVSRSPPAAEEPSAAASDGGKWAERLLRECAGAISEKDSAKIHQLLWMLNELASPYGDCDQKLAAHFLQALFCKATETGPKCYKTLLSVSEKSHSFDSARKLILKFQEVSPWTTFGHVASNGAILEALDGETKLHIIDISNTFCTQWPTLLEALATRNDETPHLKLTVVVTAATVVKSFMKEIAQRMEKFARLMGVPFEFNVVSGLTHLGEITKDALNVRDDESVAINCIGALRRVTVDERSAILRTFLTLRPKVVTVVEEHADFTHTRHDFVKCFEECLRFYTLYLEMLAKSFPATSNERLMLERECSRSILRVLGCDDPSSNDGDSESRERGTQWSEKLRDAGFSPFTLNDDAVDDVKALLKRYKSGWALQPPQPQAGEDNTTGTSSSSTSSGIYLTWKDEPVVWASAWKP

>IbGRAS53

MSVETRKNRDNQPLEDDSVVVAESKREQPSESSLQILQRYWSDLMHGGGKKLNTIRIDKPSNSSAAVQMVLSPEEIMQIARSKLEQCAFQSCCVHSVVFEHSYLLGETDIKGGDVELALLLQASAEMVANQQFDRARKLLGLCNQSASANGSTVERIVYYFARALKERMDLDRDTETEESEKVPLNVEEAVMSMEAAIIACVQDLPFSQVTNFTGVHAILDNITSARKVHLVDFEIGSGSHWTIIMQDLANRSEPPIESLKITAVGSSKRRIERTGKWLSSFAETMNLPFSFKAIVCDAKDLRKELFEMEADEVVAVFAEYRLSTLLVCPNQLQNLIAVIQTFNPSVMVVAETEADTNTPSFLARFYNLLSYCTATLDSVATCMDRDHQYRKITEQVIHWELIRNVITTEGADRIYRHAKIDFWRQFFARFGIEEEALSHSALYQASFLIRKYPSWSHCSLDMNGKSMIIKWKGTPVKSLSVWKFCPVKNN

>IbGRAS54

MSGLNKSDVFGGAPDRNRCIDDVENNDDNMKAVKSRPPADEVEEWGDSTEIAWFSSCQVLDQDGDERPRKEAPLLPRHKHEHQLQLVVDCMSLDDLDFDEEASSVQPFSGNQELSESKRKRAENALPSSLKLLNNFQNRFRRLTGEKVNAAASCSSHDESREMNCCSSTRLWINEVLQLAAHKFIENSSHGDSELNNLFPNSCWGLHGEDYKDVELLLHLLASAERVGQKKFDSAKDFLSMCDKLSSKNGNLVQRLVYYFSEALRDKVDWQTGEKTPENFGKKQIEYLKEELTCPRKCILETHQNFPFLQVIEFASVQAVIEHVAEAKKIHIIDLEINHGMQWTILMQALAAACHAESSIENLKISALVIKSGPLIEETGKQLTSFADSLKLPFSFKTVKVQDILEVNEQSFEVEEDEALAVYGLFFFMTMISKQDRLEHLMRVMRTIRPRVMVITEVEANHNSPVFVNRFTESLFFYSAFLDSLEHFLKHDEYIRANLEREQLSRRIRNIVAAEGEERVIRHVSMNVWRAFFARFGMKEVEMSMSSVLQANLVFNNFPYGKYCTLDNDGKSMIIGWKRTPLFSVSAWKLI

>IbGRAS55

MSLVRSSTPFGNRKLYLLNGHGDSSTGLSTSMFNPEKHEIAYAAESYSSGSYDANYFLDSPSPSSDLVHPSASEALVNAFQHCPPPCPVSSGENSLSSIQSLRKCGTYQVNYDSEYISSQSPDPLDFEEGNVRLKLQELERALLDDNDDDAMFGCTQSMEVDGQWADPICSLFQNDSPKESSSSESNVSTRSSNKVDTHTHQTPKQLLFSCAAAIQHGNLEQASAMINELRQMVSIQGEPSDRIAAYMVEALAARMDTSGKGLYKALKCKAPPSNDRLSAMQVLFEVCPCFRFGFMAANGAILEALMGEKNVNIIDFDINQGSQYYTLLQTLASMPGKPPHLRLTGIDDPESVQRPTGGLRLIGLRLEKLAEDLKLPFEFRAMPAESALVAPTMLGCQPGETVIVNFAFQLHHMPDESVSTVNQRDQLLRMVKSLNPKLVTVVEQDVNTNTAPFLPRFAEAYNYYSAVFESLDATLPRDSQERMNVERHCLARDIINIIACEGEERIERYEVAGKWRARMMMAGFNSCPISRNVNDSIRKLIKQYSERYKVKEDAGSLHFGWEDKILIVASAWR

>IbGRAS56

MSLVRSSTPFGNRKLYLLNGHGDNSTGLSASMFNPEKREIAYAAESYSSGSYDANYFLDSPSPSSELVHPSASEALVNSFQHPPPPPCPVSSGENSLSSIQSLRKCDTYQSNYDSEYISSQSPDPLDFEEGNVRLKLQELERALLDDDNDDDAMFGCTQSMEVDGHNKVDTHTQQTPKQLLFSCAAAIQHGNLEQASAMINKLRQMVSIQGEPCDRIAAYMVEALAARMDTSGKGLYKALKCKAPPSNDRLSAMQVLFEVCPCFRFGFMAANGAILEALTGEKNVHIIDFDINQGSQYYTLLQTLASMPGKPPHLRVTGIDDPESVQRPTGGLRLIGLRLEKLAEDLKLPFEFRAMPAQSELVAPTMLGCQPGETVIVNFAFQLHHMPDESVSTVNQRDQLLRMVKSLNPKLRTVVEARCEHQYYPISTEIC

>IbGRAS57

MDYVNCGSPLSGSSSGVDDGNELKHVLRGLENKLLGPEPEDSCSLNDDLVVVVSKPSFSSMGLKELLLACAEAVSDADISTAEALMNLLEQRVSVSGDPIQRLGAYMLEGLRARVLASGSIIYRKLKCREPTGSELLSYMQVLYHICPYYKFAYMSANIVIQEAMVNEKSIHIIDFQIAQGSQWVFFIQALANRRGGCGSPPFVRITGVDDSQSTQARGGGLQLVGEMLAKVAASCGVGFEFHAAAISWCSEVEMEDLNIRHGEALAVNFPYMLHHTPDESVSTKNHRDRLLRLVRSLSPRVVTLVEQESNTNTAAFLPRFRETLDYYTAMFECIDASARPREDRQRVSAEEHCVARDIVNIIACEGSDRVERHELFGKWKLRLVMAGFSLCPLSPSVGLAMRDMLKEYSPNYRLAESDGALYLGWKNRALVTSSAWRC

>IbGRAS58

MQASEVSRTSGEQYYNPFHVLNNYISNDHCNSATWDPSIHTHNQHFFTLDSSPTMDYVNCGSPLSGSSSGVDDGNELRHVLRELENKLLGPEPEDSCSLNDDDLVVVVSKPSFSSMGLKELLLACAEAVSDADISTAEALMNLLEQRVSVSGDPIQRLSAYMLEGLRARVLASGSIIYRKLKCREPTGSELLSYMQVLYHICPYYKFAYMSANIVIQEAMVNEKSIHIIDFQIAQGSQWVFFIQALANRRRGCGSPPFVRITGVDDSQSAQARGRWASAGCEVEMEDLNIRHGEALAVNFPYMLHHMPDESVSTKNHRDRLLRLVRSLSPRVVTLVEQESNTNTAAFLPRFRETLDYYTAMFECIDAAARPREDRQRVSAEEHCVARDIVNIIACEGSDRVERHELFGKWKLRLVMAGFTLCPLSPSVGLAVRDMLKEYSPNYRLAESDGALYLGWKNRALVTSSAWRC

>IbGRAS59

MDHKANNPSSSNYNYFQGVLDYINQMLMEDDDLENGRFVYPDLLALQAAEKSLGDVLNAEESSGNDGGRRFPDRPGEKRHRCGEDINGVEAGRRRRSNNNNNVARDEEQVQIEMYNGIKGLCYCRELDASAKRVVQPRVRARRGRPRAGERGGNGKGEVDFAGLLNLCAQAVGGEDFRTVNELLGRIRRHCSPHGGAAERLSHYFAKALDARLAGTGAALYTANRASLADTLKAYQMYFTACPFKKLSNMFADKSIGKLTMEATKIHIIDFGISHGFQWPCLIQGLSRRPGGPPALRITGIDLNAEATATGRHLSYYSNKFNVPFQYTAVTKKWETICSEDVRIERDEVLVVNCLYRLQDVPDEIASEESPRDTVLNFIKQLNPQVFMHGVVSASYNALVFSTRFREAIFHFSSLFDMLEGTVGGGDEGRMVYEREILGREIMNVVACEGAERMKRPETYKQWQERIQRAGFEQMLPDGDIMRQVRAKVMDYSNDFFVEEDGKWMLQGWKGRVIYATSSWKPVINH

>IbGRAS60

MPSVSFPSGSAPFNHDRHLHPKGSVNDELRSSKDDNNDWFEMEGCDDFNSLFAGDKRIEHLIIEDQNQIPSPNPQVSELKPQQTPFSSACFEILKCYGRFFKDRSSLTSKTDRIDVSGAGGKLSVAEILKLAGERYIQFFTQKADGFSAFFHPYASSLSGISVDDIRDVELVHLLLAAGEEVSRRQYHLASGFISRCLWIASDSGTPVQRLAFQFSEALTERIERETGRFKGVSEERLARCRESMALSSNPAILASHQGLPFGQVIQFAGIQAIIERVKNARKIRLLDINIRTGIQWTILMQALAEQDRSSPIERIRLTAVGVVEREKMEECGKRLQSFADSLNLPFYFDTVFLSDLKDFREDLVQVEDEEVVAVMANIVLRTMIARPDCLDTLMRGIRRLRPAVMVVAEVEANHNSPSFINRFVEALFFWRADCLEDCGSRRSVRRIMEGLHFGKGSKHCGGGGEERFTRKRED

>IbGRAS61

MPGISFPSAPFNHDHHLHRKGSVNDELRSSKDDNNDWFEIEKCDDDFDSLFAGAKKIEHVIEGQNQIPSPNPQISEPKPHRTPFSSAYFEILKCYGRFFKDRSSVTSKTDHTDAGGTGGKLSVAEILKLAGERYIQFFTQKVDGFSAFSHPYASALSGISVDDVRDVELVHLLLAAGEDVSRRQYHLATGFITRCLWTASDSGTPVQRLAFHFAEALTERIERGNGKVIQFAGIQAIIERVKNARKIHLVDINIRSGIQWTILMQALAEQHDSSPIERIRLTAIGVAEREKMEECGKRLQSFADSLNLPFCFHTIFLSDLKDFREDLVQIEDEEVVAVLASTVLRTMIARPDCLDNLMRGIRGLRPAVMVVAEVEANHNSRSFINRFVEALFFYGAYFDCLDDCLDRDDPCRRIIEGLHFGEGIRNVVATEGDERFSRNVKIEVWRAYFARFPMEEMQLSDSSAYQANLVGRKFGNGNSCTLDRSGKGLIFGWKGTPMHSVTCWKFP

>IbGRAS62

MKRGNFNRSVQGSFAGAASGGGGGSGSSSGVSKGKKVWEESEQDAGMDELLAVLGYKVKSSDMAEVAQKLEQLEEVMGSVQGDDLSNFASETVHYNPSDLSSWVDSMISELNPGDPFLSQTESSAITSLPTQIFDDSSFDSDLTAIPGKAAYPQPPSKRFRTASTTSSTSSNMQLGGAAAWGSPSESISPSSESTRSVVLVDSQENGVRLVHTLMACAEAIQKENMKLAEALVKQIGFLAVSQAGAMRKVATYFAEALARRIYRLYPSNHNDSAFSDLLQMHFYESCPYLKFAHFTANQAILEAFANKNRVHVIDFSMKQGMQWPALLQALALRPGGPPSFRLTGIGPPSNDNTDHLQEVGWKLAQLAETINVEFEFRGFVANSLADLDASMFNIQDGETVAVNSIFELHQLLARPAAIEKVLSVIKDMKPEIVTVVEQEANHNGPVFMDRFTESLHYYSTLFDSLEGCAASEGGTVCDQDKVMSEVYLGRQICNVVACEGVDRVERHETLAQWRTRFKSAGFSPVHLGSNAYKQASMLLALFAGGDGYRVEENDGGLTLGWHTRPLIATSAWKLTS

>IbGRAS63

MFQDEGSSSSITSSSPLQALPVGVSVSPGSPYPWLKELKSEERGLYLIHLLLTCAGHVAGGSLENSNVALDQISHLAAPDGDTMQRIACYFAQALADRVLRTLPGVYRALHSTKLASPADEFLARKVFVEMFPFSKVAFLVANQAIIEAMEGEKMVHIIDFHAADPTQWRALLQDLSARPEGPPHLRITGVHPVKEVMEQMGRVLSEEAEKLDIPFQFNAIVSKFESLDLEKLRVKTGEALAISSPMQLHTLLAYDDDKKPSPFASKIPNRRIQITQNSLGDFIEQDVGNGYSPSTDSASSSLSNSPKIEAFLSALWGLSPKLMVVTEQDSNHNGQTIMERLSESLYFYAALFDCLESTLPRSSLERLKVEKALLGEEIKNIIACEGSERKERHEKLEKWFQRLESAGFGNVPFSYYAMLQARRFLQSFGCDGYRIKEENGCVVTCWQDRPLFSVSAWRCRR

>IbGRAS64

MQSNVSRSPPAAEEPSAAASDGGKWAERLLRECAGAISEKDSAKIHQLLWMLNELASPYGDCDQKLAAHFLQALFCKATETGPKCYKTSSRSPRRATASIPLGSSSSSSKSNTFCTQWPTLLEALATRNDETPHLKLTVVVTAATVVKSFMKEIAQRMEKFARLMGVPFEFNVVSGLTHLGEITKDALNVRDDESVAINCIGALRRVGVDERSAILRTFLTLRPKVVTVVEEHADFTHTRHDFVKCFEECLRFYTLYLEMLAESFPATSNERLMLERECSRSILRVLGCDDPSSNDGDSESRERGTQWSEKLRDAGFSPFTLNDDAVDDVKALLKRYKSGWALQPPQPQAGEDNTTGTSSSSTSSGIYLTWKDEPVVWASAWKP

>IbGRAS65

MQPSQNNMNMDGSRRFYNQPMQDLESYCLPTDNQLLAVGDNHSAEFFAQTNSQYNDVMSYGYPGHSYTADSFLSDTSGILQQDSQLYLADVHHHSPEETYHSAASKSSLSDENDLKHKLRELESAMFGHGSDALEAYGSNMGTPDQMSSEAEKLEEMMGITSRGSLKESLVACAKAIGDNDIPRAEWWMSELRTMVSVCGEPIQRLGAYMLESMVARLASSGSSIYKALRCKEPTSNELFSYMYLLYEVCPFFKFGYLSANGAIVDAMKDEDRVHIIDFQISQGTQWITLIHALAARPGGPPQICITGIDDSTSAHARGGGLEIVGKRLSTLAESCKVPFEFRPVAASCADVKIEHLNVRPGEALAVNFPLVLHHMPDEDVGTQNHRERLVRMVRSLFPKVVTLVEQESDTNKVPFFPRFLETLNYYLAVFESIDVALPRDHKERINVEQHCLAREVVNILACEGVERVERHEVLEKWRSRFSMAGFKPYPLSSSVNATIKTLLENYCKDYTLEERDQALYLGWMNRALIASCAWQ

>IbGRAS66

MRASRLRRTGMSNTLCFQQKAEAYCVPQFQILDQLSHSDNGGNYSIQAYSDHCCTRELSSKNGSHARCNSSSTISFSPYGSPMLQQQEFQSYPLDLHQSPETKYSSPISMSSSITDDMSDFRHKLKELESLETNDGKHTVGDLKQVLVTCAKAISINDLPKAECLMSQLRQMVSVSGEPIQRLGAYVLEGLVARLDASGSSIYKSLRCKEPESFELLSYMHILYEVCPYFKFGYMSANGAIAEAMKGENRVHIIDFQIGQGSQWVTLIQAFAARPGGPPHIRITGIDDSTSAYARRGGLNIVGKMLSKLAESFNVPFEFHAASMSDYEVQVADLGIHTGEALAVNFAFMLHHTPDESVSTQNHRDELLRLVKGLNPKVVTLVEQECNTNTAPFFPRFLETLDYYVAMFESIDVNLPREDKQRINVEQHCLARDVVNIIACEGTERVERHELLGKWKSRFRMAGFSPYPLSSLVNATIKTLLGNYSSKYRLEERDGALYLGWMKRDLVASCAWK

>IbGRAS67

MAAKAFPMVGEAANVSGSSGSATSSREYHHHDHNNILPLHSSSASPSHLALLCDNAKMVRKRAASEMELQIGGGGGIGEHGRFLRRAAAAGMNAPLLGDLRVCGIAPPPSSTNLSVTSTSDATHLTYMETLPLPLPLPLPLPNPPPNEAQPLPLCVFSGLPLFPAPTRARNAAGALQPPPLPAAGCGGSAIGVNSSSGMGDNGTAMAWIDGIIKDLIHSSTHVSIPQLIQNVREIIHPCNPNLAALLEYRLRSLTTADPLAANVYDDWRRKETLQPQSQDAAITHPLHLPDSMPHPWEITLPPAAATASTTRHHHHHHHQQHQLRDNSPSVTAGLPFVPAPSSDRQEQQQQQGRMDHEKQPESQSQSQSPPPSESTAAAAALIRTESLRREKDELEQQKKDEEGLHLLTLLLQCAEAVAADNLDEANRMLLQVSELSTPYGTSAQRVAAYFSEAMSARLVFNGISPFVKFSHFTANQAIQEAFEREDRVHIIDLDIMQGLQWPGLFHILASRPGGPPLVRLTGLGTSMEALEATGKRLSDFAQKLGLPFEFFPVADKVGNLDPQRLNVNKREAVAVHWLQHSLYDVTGSDTNTLWLLQRLAPKVVTVVEQDLSHAGSFLGRFVEAIHYYSALFDSLGACYGEESEERHAVEQQLLSREIRNVLAVGGPSRSSEVKFNNWREKFQQSGFRGVSLAGNAAAQATLLLGMFHSDGYTLAEDNGALKLGWKDLCLLTASAWRPPPLAQ

>IbGRAS68

MMQPQLFHPSWLSYETNYSTSPCTGSLSFFEDVFVTDNSYVSPVITADSSGLDSTLFHDDFPEFAYLPPLLEGDVSMDDIEDVCRWLNNEESEEGTNNTSSELTKDVLSPDFSVVSAEDSMAVLPGNGVEVDDSHWCLLHLLAAYAEAMGDMQRELAKEIAGCIRRKANALGETLERVAYNVVQTTEDQGGSYLRREAIKNYETAFKVLYQVLPHGRFAHFSANSAILEAIPDGAEAVRIIDFDMGDGVQWPSLIESMAQTRRALRLTSVKKGRGVYHQRSEKNKENRQKEEWLAFNCIFRLPHMTNRQQRSQAMQFLEIAKELSPYSAIQSGIVVFADGESGCWSSSFSDYSSFFNRQLVHYKSLFESMEWHFPVNLTDARIAVESLFLAPHACSDSWFHDWQENKMKAISNLRAEMGLQGRKLSIENILQAKEMVNERESPYRVRIEEENQHEMILEWRETPLVRVSTWM

>IbGRAS69

MQAAQRLRRTGMSNVLCYQPVQKADPYQLSYDNTNGASSFHASRNLYCTLESSSVSESRVLYNSPSAVCFSTDGSPMSQQDSCSYPLDNNYGSPISGSCITDDMSSFIHKLKELETVMLGPDSDILGSYDNAFPSSIASPEIDSWRQMMEARGDLKQVLIACAKAVSDNDLLTAQWLMSELRQMVSVSGEPIQRLGAYMLEGLVARLAASGSSIYKSLRCKEPTSFELLSYMHILYEVCPYFKFGYMSANGAIAEAMKDENRVHIIDFQINQGSQWITLIQAFAARPGGPPHIRITGIDDSSLCLSNAHSGGGLSLVGKRLSKLAESFNVPFEFHAAAIPGSDIHLENLRIQPGEALAVNFAYMLHHIPDESVSTQNHRDELLRLVKSLNPKVVTLVEQESNTNTSAFFPRFLETLDYYTAMFESIDVTLPRDHKERINVEQHCLARDVVNIIACEGIERVERHELLGKWKSRFRMAGFTPYPLSSVVNATIKKLLESYSDKYRLEERNGALYLGWMNRDLVASCAWK

>IbGRAS70

MTIDEPAEPRPFTDQILEWFDSSFLDCPYDSNDHFFGDSWWGDQGQNLEILHKSDDNGGGVSTSLNSFSSVTTAVEAPVVLDHPAAQPPVDKKRKGREEGEAEVEVEQAAAPVRKGGGNKKGGNKGGGNSNSNCNKDGRWAEQLLNPCAAAITAGNMNRVQHLLYVLSELASLTGDANHRLAAHGLQALTHHLGSGSSFAGVTTFASTTKKFFRESLMIFNDINPWFRIPNSFANSSILQALAEQQDRPRCLHILDIGVSHGIQWPTLLEELTHRPGGPPPLVRLTVITPTVDNQQLSCNNTPFLIPPSGYDFSPNLLGFAKAININLQINILDNLPLQNLIAHAQSIKSSQDEILIVCAQFRLHNLNHHAPDERTEFLKSLRNLAPKRVVLSENNAECSCSNCGDFAAGFSRKVEYLWRFLESTSMAFKGRWNEERRMMEGEAAKALINTGEMNERKEKWCERMRGVGFAREEFGEDAIDGARMLLRKYDNNWEMKVEEKDGCVDLWWKGQPLSFTSLWKMDPNHGFN

>IbGRAS71

MDSHQFIAYGVDLSFSSFPQTSSLPATLFDSLKFDSTSSPNSPFSNCFDPQTATTLSDSQELYSSTENLSGASPSSNSSLDYNSYPQRCSPASDCLPESLVLPSGEYTFLRNVNHNEKMKHVLWQLESALMGPDGVGATNSDPSAGENTQKQTSSQGSRSWSQEAQGSGRYESQQSSFGRSGEGIHSEKRHKTMLDFPAQGGPVNIKQLLIECARALDENKLLDFDRLIEVARCAVSITGDPIQRLGAYMIEGLVARKEASGTNIYRTLKCKEPAGDDLLSYMHILYEICPYLKFGYMAANGAIAEACRNEDRIHIIDFQICQGTQWMTLLQALAARPGGAPYVRITGIDDPLSKHARGDGLVTVGKRLAAISEKFNIPVEFNPVPVFAPQVTREMLDIRPGEALAVNFPLQLHHTPDESVDVSNPRDGLLRMVKSLSPKVVTLVEQESNTNTAPFFSRFIEALEYYSAMFESLDVVLPRDRKERINVEQHCLARDIVNVIACEGKERVERHELLGKWKSRFTMAGFQQYPLSSYVNSVIKGLLKCYSEHYTLLEKDGAMLLGWKERNLISASAWH

>IbGRAS72

MMLNTLCGSMGSLSSEVGNLKIEEEPSNSVKRENSSLLSSSSSCSTLQQHQSDKVHVPAPSDYEDHSSVVNGISFPAIKFEVPGGGDDIGIDDQSYWESFFADQLEGDFMISSPVRSNNMAASPQVSSFMNDVQMMITSPVRNCYHHHQNGITMSPPRMMMMSPLGPGHNNKGKGLSPLHRVFNSPNAQHLMQMQTHDFSHLPALDNLLDFDAHQDVLDDNHDFSSYSSTLKHLPASSSVLSESLLDCGGLQLPRLHSSSSCSASVSSIQAPPLEADDDIIYRTTTTTTAAGGSLHIAPLSHQLREERNQEQQHHHNNNNIRRSGQHLHSAPLVLPLSAPPADLQEEEEEQDSGLQLLHLLLVCAEAVSKENYMLARRYLHHLNRVVTPLGDSMQRVASCFTEALSARLAATLASASAKSITSNRPFNPPYPPNSLEILKIYQILYQACPYVKFAHFTANQAIFEAFEAEERVHVIDLDILQGYQWPAFMQALAARPGGAPFLRITGVGPSPEAVRETGRCLTELAHSLHVPFEFHPVGEELQDLKPHMFNRRVGEALAVNAVNRLHRVPPNCMGNLLGMIRDQAPNIVTIVEQEANHNGPYFLGRFLEALHYYSAIFDSLDATFPADSSQRAKVEQYIFAQEIWNIVACEGAERVYRHERLERWRRVMEGKGFKGVPLSANAVTQSNILLGLYSCDGYRLTEDKGCLLLGWQDRPILAASAWRC
