# Supplementary material for: Genome-wide survey and expression analysis of GRAS transcription factor family in sweetpotato provides insights into their potential roles in stress response
Source: BMC Plant Biol. 2022 May 6;22:232. doi: 10.1186/s12870-022-03618-5 (PMC9074257; doi:10.1186/s12870-022-03618-5)
Supplement: Supplementary file 3 — Additional file 3: Accession numbers of GRAS genes in sweetpotato and Arabidopsis. [file 12870_2022_3618_MOESM3_ESM.docx]

## Additional file 11. Specific primer sequences used for gene cloning, qRT-PCR analysis and vector construction.

| Primer code | Primer sequences (5' →3') | Application |
| --- | --- | --- |
| IbARF-Q-F | CTTTGCCAAGAAGGAGATGC | Internal standard gene for qRT-PCR analysis |
| IbARF-Q-R | CTTGTCCTGACCACCAACA |  |
| IbGRAS2-Q-F | CGACCTCTCTAAGCGACAGC | qRT-PCR analysis for *IbGRAS* genes |
| IbGRAS2-Q-R | GGAAGTAACTGCTATAATCCAGCA |  |
| IbGRAS4-Q-F | GCGTGCGGCAGTTAATATGG |  |
| IbGRAS4-Q-R | TAAACAGCGCCTGGAGAAAGTAA |  |
| IbGRAS9-Q-F | GGACTGTGGACTTTGTTGCG |  |
| IbGRAS9-Q-R | CGCCCCAACTCCATCAAC |  |
| IbGRAS16-Q-F | TGCGGATGCTCTTCAGGC |  |
| IbGRAS16-Q-R | GCTAGGCACTCGCCGTCC |  |
| IbGRAS21-Q-F | CAGCCAATCTGGGTCGGT |  |
| IbGRAS21-Q-R | ATTGATGGCGTTACTGTGAGGT |  |
| IbGRAS31-Q-F | TCATTATACAGTTTCCAATTCCGAT |  |
| IbGRAS31-Q-R | TCAGATTCACTGGGATACGGAT |  |
| IbGRAS36-Q-F | TGGTAACAATGGTGATTCTGGTT |  |
| IbGRAS36-Q-R | TGTAGGATGACTGAGAATTAGTCCG |  |
| IbGRAS51-Q-F | CGGCTTTAGCAGCAGTGAAC |  |
| IbGRAS51-Q-R | GATTAGGATTAGCCTCCATAGCG |  |
| IbGRAS58-Q-F | CTGCTGCTTGCCTGTGCTG |  |
| IbGRAS58-Q-R | AGGGTCCCCAGAAACCGATA |  |
| IbGRAS65-Q-F | TCCCGAGGAGACCTACCACT |  |
| IbGRAS65-Q-R | CAAACATCGCAGATTCCAGC |  |
| IbGRAS66-Q-F | CAAAAGGCGGAGGCGTAC |  |
| IbGRAS66-Q-R | GGCTTCCATAGGGCGAGA |  |
| IbGRAS71-Q-F | TCCCAATTCGCCGTTCAG |  |
| IbGRAS71-Q-R | CACCAGAAGGAAGAACCAAGC |  |
| IbGRAS2-Clone-F | AGAATAGCCTGTGCCTAGTGCA | Gene cloning and vector construction for *IbGRAS* genes |
| IbGRAS2-Clone-R | CAAGACATCTATGGAGGATGCCTAT |  |
| IbGRAS2-GW-F | GGGGACAAGTTTGTACAAAAAAGCAGGCTTCATGGACTCCCATCAGTTTTTTG |  |
| IbGRAS2-GW-R | GGGGACCACTTTGTACAAGAAAGCTGGGTCCAAAAAACTGATGGGAGTCCAT |  |
| IbGRAS4-Clone-F | ATGCTTATTCACTCCTGTTTTCATG |  |
| IbGRAS4-Clone-R | CTTGATCCACAAATTAACTCCAATT |  |
| IbGRAS4-GW-F | GGGGACAAGTTTGTACAAAAAAGCAGGCTTCATGGATAATAATGAAGACGACTTTTC |  |
| IbGRAS4-GW-R | GGGGACCACTTTGTACAAGAAAGCTGGGTCCCGGCCATCGCCGTCG |  |
| IbGRAS9-Clone-F | GTTTGGGTTTCTGGATTGTGC |  |
| IbGRAS9-Clone-R | ACCCTCGTAGTGACTGCGGA |  |
| IbGRAS9-GW-F | GGGGACAAGTTTGTACAAAAAAGCAGGCTTCATGGATAGGGGACCATATGAGG |  |
| IbGRAS9-GW-R | GGGGACCACTTTGTACAAGAAAGCTGGGTCCCTCATATGGTCCCCTATCCAT |  |
| IbGRAS58-Clone-F | TTCTACTTTGAGGTTGCTTGAACTAC |  |
| IbGRAS58-Clone-R | AAACAGAAGAGGTGATATCGAAATTC |  |
| IbGRAS58-GW-F | GGGGACAAGTTTGTACAAAAAAGCAGGCTTCATGCAAGCATCAGAGGTTTCCCGAA |  |
| IbGRAS58-GW-R  IbGRAS71-Clone-F  IbGRAS71-Clone-R  IbGRAS71-GW-F  IbGRAS71-GW-R | GGGGACCACTTTGTACAAGAAAGCTGGGTCTTCGGGAAACCTCTGATGCTTGCAT  CATGTTTAGAATAGTCTGTGGCTGG  CTTTGTGTCTGTGTCCATCCCA  GGGGACAAGTTTGTACAAAAAAGCAGGCTTCATGGACTCACATCAGTTTATTGCAT  GGGGACCACTTTGTACAAGAAAGCTGGGTCATGCAATAAACTGATGTGAGTCCAT |  |
