# Supplementary material for: Genome-wide survey and expression analysis of GRAS transcription factor family in sweetpotato provides insights into their potential roles in stress response
Source: BMC Plant Biol. 2022 May 6;22:232. doi: 10.1186/s12870-022-03618-5 (PMC9074257; doi:10.1186/s12870-022-03618-5)

**Additional file 2**. Schematic representations of the chromosomal distribution of the 72 *IbGRAS* genes on 15 sweetpotato chromosomes. The chromosome numbers 1-15 are indicated to the left of each chromosome as LG1-LG15, and tandem duplicated genes are marked with red arcs. The scales were indicated the genome size of sweetpotato genome (Mb).


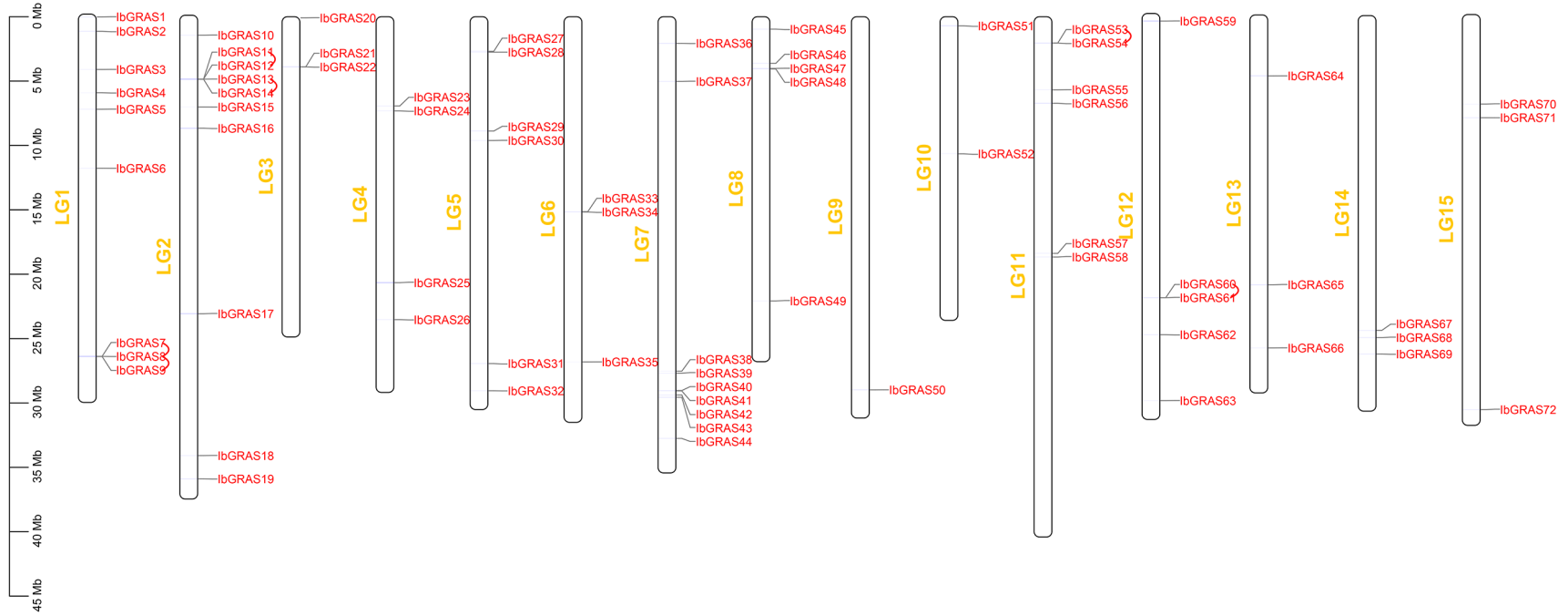

Supplement: Supplementary file 4 — Additional file 4: Chromosomal locations and segmental duplications of IbGRAS genes in sweetpotato. [file 12870_2022_3618_MOESM4_ESM.docx]
