# Supplementary material for: Genome-wide survey and expression analysis of GRAS transcription factor family in sweetpotato provides insights into their potential roles in stress response
Source: BMC Plant Biol. 2022 May 6;22:232. doi: 10.1186/s12870-022-03618-5 (PMC9074257; doi:10.1186/s12870-022-03618-5)

**Additional file 6**. Venn diagrams among the detected species with orthologous genes of sweetpotato *IbGRAS* genes.


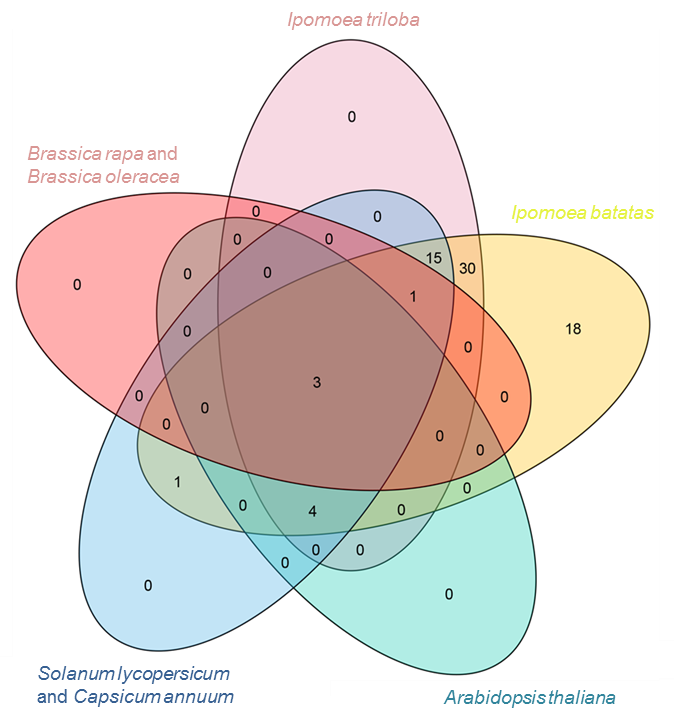

Supplement: Supplementary file 8 — Additional file 8: Differentially expressed IbGRAS genes in sweetpotato transcriptome analysis under salt stress. CR, Control roots; SR, Salt-treated roots. [file 12870_2022_3618_MOESM8_ESM.docx]
