# Supplementary material for: Genome-wide survey and expression analysis of GRAS transcription factor family in sweetpotato provides insights into their potential roles in stress response
Source: BMC Plant Biol. 2022 May 6;22:232. doi: 10.1186/s12870-022-03618-5 (PMC9074257; doi:10.1186/s12870-022-03618-5)
Supplement: Supplementary file 9 — Additional file 9: Cis-elements associated with hormone and abiotic stress within the IbGRAS gene promoters. [file 12870_2022_3618_MOESM9_ESM.docx]

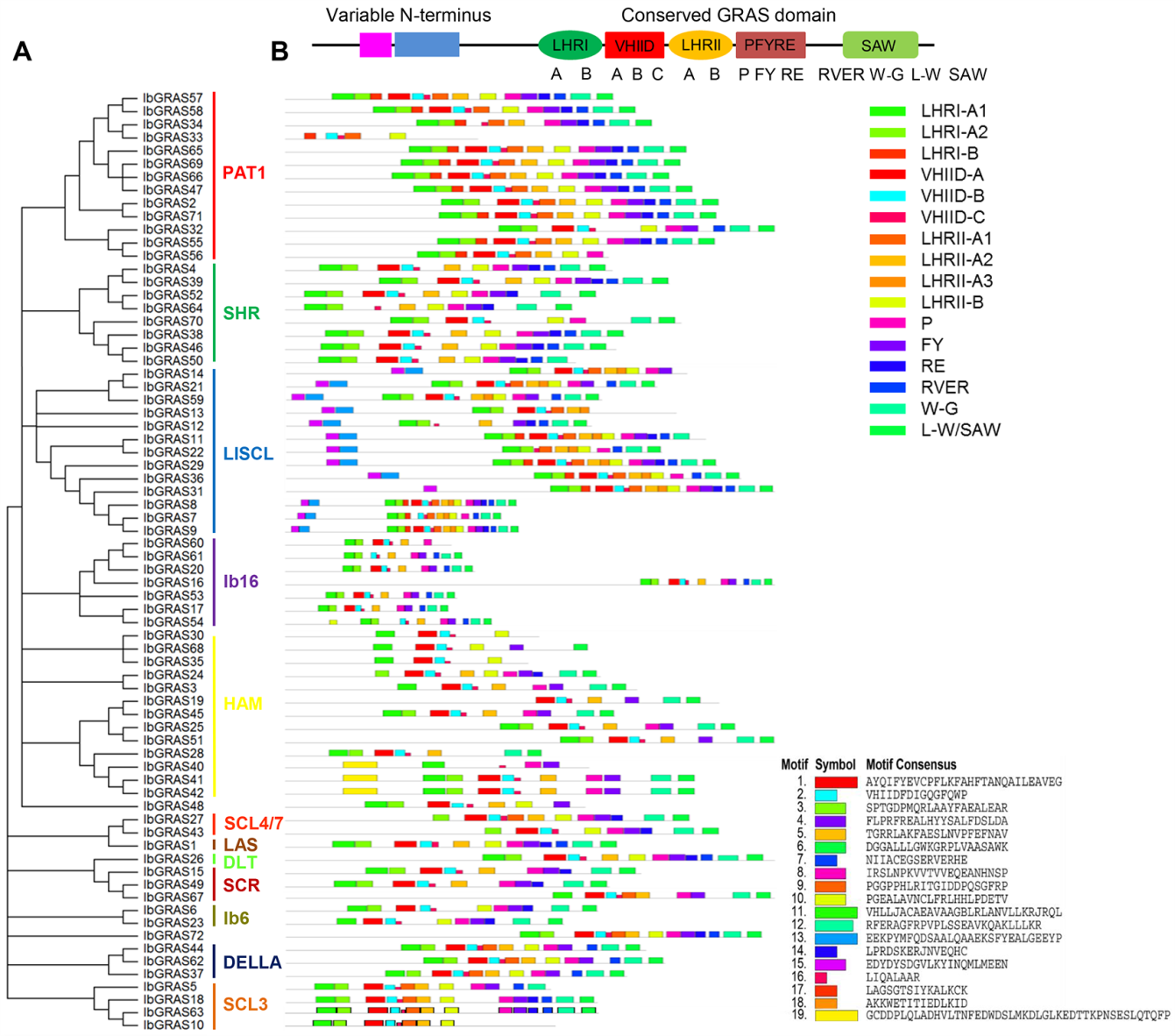
**Additional file 7**. Phylogenetic relationships and distributions of amino acid motif compositions within the IbGRAS proteins identified by MEME. **A**. The phylogenetic tree of 72 IbGRASs was constructed by MEGA X based on the consistent parameters used in Fig. 2. **B**. The distribution of 19 most significantly conserved motifs in IbGRAS proteins. The settings of conserved motifs are based on the reports in *Arabidopsis* and rice [1]. Different motifs and their positions in each IbGRAS member are represented by differently colored boxes. The black lines represent the relative length of protein.

1. Tian C, Wan P, Sun S, Li J, Chen M: **Genome-wide analysis of the GRAS gene family in rice and *Arabidopsis***. *Plant molecular biology* 2004, **54**(4):519-532.
